# Expanding Water/Base Tolerant Frustrated Lewis Pair Chemistry to Alkylamines Enables Broad Scope Reductive Aminations

**DOI:** 10.1002/chem.201605466

**Published:** 2017-01-18

**Authors:** Valerio Fasano, Michael J. Ingleson

**Affiliations:** ^1^ School of ChemistryUniversity of ManchesterManchesterM13 9PLUK

**Keywords:** frustrated Lewis pairs, boron, protodeboronation, reductive amination, water tolerance

## Abstract

Lower Lewis acidity boranes demonstrate greater tolerance to combinations of water/strong Brønsted bases than B(C_6_F_5_)_3_, this enables Si−H bond activation by a frustrated Lewis pair (FLP) mechanism to proceed in the presence of H_2_O/alkylamines. Specifically, BPh_3_ has improved water tolerance in the presence of alkylamines as the Brønsted acidic adduct H_2_O–BPh_3_ does not undergo irreversible deprotonation with aliphatic amines in contrast to H_2_O–B(C_6_F_5_)_3_. Therefore BPh_3_ is a catalyst for the reductive amination of aldehydes and ketones with alkylamines using silanes as reductants. A range of amines inaccessible using B(C_6_F_5_)_3_ as catalyst, were accessible by reductive amination catalysed by BPh_3_ via an operationally simple methodology requiring no purification of BPh_3_ or reagents/solvent. BPh_3_ has a complementary reductive amination scope to B(C_6_F_5_)_3_ with the former not an effective catalyst for the reductive amination of arylamines, while the latter is not an effective catalyst for the reductive amination of alkylamines. This disparity is due to the different p*K*_a_ values of the water–borane adducts and the greater susceptibility of BPh_3_ species towards protodeboronation. An understanding of the deactivation processes occurring using B(C_6_F_5_)_3_ and BPh_3_ as reductive amination catalysts led to the identification of a third triarylborane, B(3,5‐Cl_2_C_6_H_3_)_3_, that has a broader substrate scope being able to catalyse the reductive amination of both aryl and alkyl amines with carbonyls.

## Introduction

Considerable progress in frustrated Lewis pair (FLP) chemistry has been achieved in the last decade principally using tris(pentafluorophenyl)borane, B(C_6_F_5_)_3_.^1^ Compared to BPh_3_, the presence of fluorine atoms dramatically increases the Lewis acidity.^2^ While high Lewis acidity is essential in enabling certain FLP reactivity, it also poses challenges including the compatibility of FLPs with water (e.g. from unpurified reactants/solvents or as a reaction by‐product)/ base combinations, a topic which has attracted recent attention.^3^, ^4^, ^5^, ^6^ A fluorinated triarylborane with a high Lewis acidity towards hydride (which is desirable for H−H and Si−H bond activations) also has considerable oxophilicity, with the corresponding triarylborane–water adduct exhibiting much greater Brønsted acidity than water itself.^7^ Indeed, the Brønsted acidity of H_2_O–B(C_6_F_5_)_3_ was determined by Parkin and co‐workers (p*K*_a_=8.4 in MeCN) to be comparable to that of HCl (8.5 in MeCN).7a This poses a limit to the water tolerance of these fluorinated arylboranes in the presence of certain Brønsted bases because irreversible deprotonation of the borane–water adduct yields an inactive (for FLP chemistry) hydroxytriarylborate anion.

**Scheme 1:**
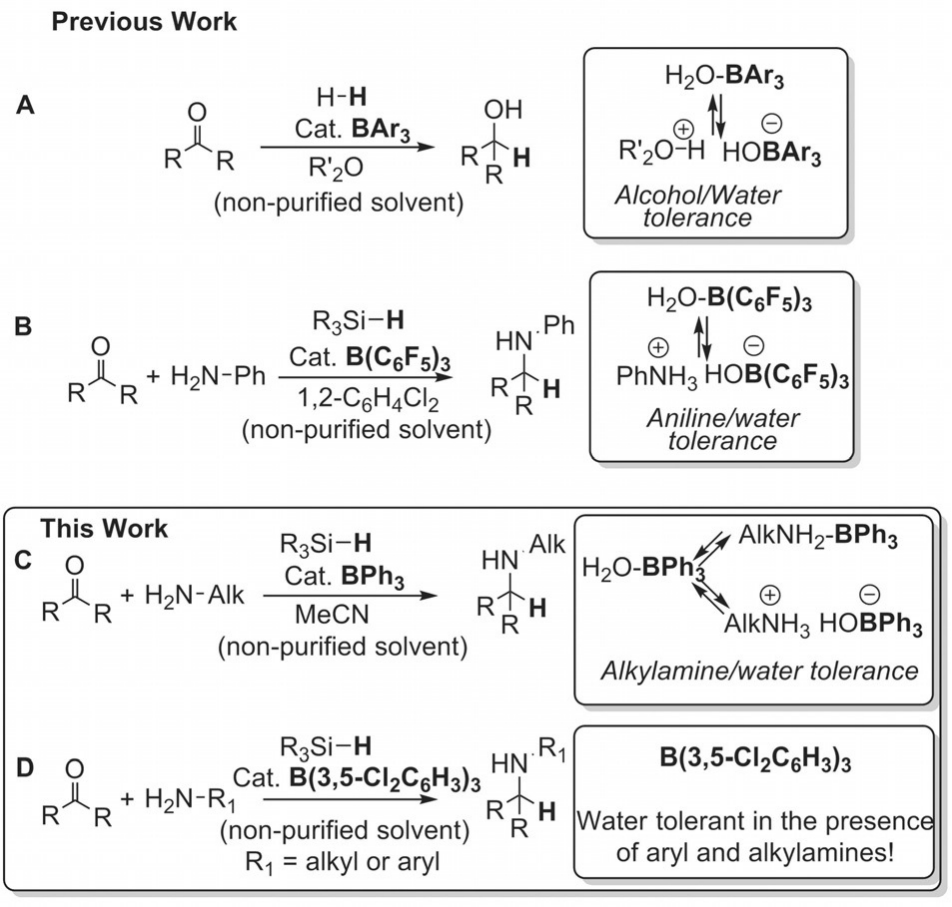
Previous work (top and middle): alcohols and anilines tolerated by fluorinated‐triarylborane–water adducts; this work (inset): alkylamines tolerated by the BPh_3_–OH_2_ adduct and both alkyl and arylamine/H_2_O combinations tolerated by B(3,5‐Cl_2_C_6_H_3_)_3_.

Ashley, Stephan, and co‐workers pioneered ROH‐tolerant FLP reactions and demonstrated that B(C_6_F_5_)_3_ could be used for the hydrogenation of carbonyls (Scheme 1 A). Importantly, the alcohol–borane adducts are not irreversibly deprotonated under these weakly basic conditions (which use ethereal solvents such as 1,4‐dioxane as Lewis bases to activate H_2_ via an FLP mechanism).^3^, ^8^ Demonstration of the water tolerance of B(C_6_F_5_)_3_ was subsequently reported proving that the hydrogenation of ketones could be performed using non‐purified, “wet” reactants and solvents (H_2_O–B(C_6_F_5_)_3_ also is not irreversibly deprotonated by ethereal solvents).^4^ Recently, we reported the water tolerance of a B(C_6_F_5_)_3_‐catalysed system involving more basic arylamines (conjugate acid p*K*_a_ ca. 11 in MeCN, Scheme 1 B).^5^ In particular we found that B(C_6_F_5_)_3_ is able to catalyse the reductive amination of aldehydes and ketones with anilines using 1.2 equivalents of silane as reductant.^9^ This proceeds in the presence of a super‐stoichiometric amount of water derived from imine formation and the use of non‐purified solvents. An elegant extension of this approach was recently reported using B(C_6_F_5_)_3_ to catalyse the tandem Meinwald rearrangement and reductive amination of epoxides with anilines and silanes.^10^ However, in the latter, as in our work, reductive amination could not be extended to alkylamines (conjugate acid p*K*_a_ ≥ 16 in MeCN) due to the irreversible deprotonation of H_2_O–B(C_6_F_5_)_3_. Thus, the compatibility of H_2_O–B(C_6_F_5_)_3_ with bases appears to be limited to those bases with conjugate acids that have p*K*_a_ values ≤12 (in MeCN). A broader amine scope catalytic reductive amination methodology using a simple triarylborane is desirable as a one‐pot method (thus preferable from an efficiency perspective) to rapidly access amines that are ubiquitous functionalities in natural products, pharmaceuticals and agrochemicals.

To circumvent the limitation of B(C_6_F_5_)_3_ towards water/strong Brønsted base combinations, Lewis acids that are less oxophilic are required. These could be “hydride selective” Lewis acids, such as Group 14 based Lewis acids (which maintain high hydridophilicity but have lower oxophilicity)^11^ or Lewis acids that are globally less Lewis acidic (e.g., less oxophilic and less hydridophilic).^12^ The latter approach was utilised by Papai, Soós and co‐workers who employed less Lewis acidic partially halogenated triarylboranes for example, (2,3,5,6‐C_6_F_4_H)_2_B(2,6‐C_6_H_4_Cl_2_), for the catalytic hydrogenation of carbonyls in ethereal solvents, with some water tolerance demonstrated.^6^ Taking this approach further, the non‐halogenated triarylborane BPh_3_ should have enhanced tolerance to water and strong base combinations due to its lower Lewis acidity. BPh_3_ does however still possess sufficient hydridophilicity to be useful as a catalyst in FLP‐type reactions as recently demonstrated.^13^, ^14^ While H_2_O–B(C_6_F_5_)_3_ is well documented,^7^ the corresponding H_2_O–BPh_3_ adduct is less studied, particularly its ability to act as a Brønsted acid.^16^, ^17^, ^18^, ^19^ Herein we report an extension to the water and base tolerance of boranes to strong amine bases, focusing, in particular on the triarylborane‐catalysed reductive amination of aldehydes/ketones with alkylamines using silanes as reducing agents. This demonstrates that BPh_3_ is an effective catalyst for the reductive amination of alkylamines and carbonyls (Scheme 1 C), including examples challenging to reduce with borohydride salts (e.g., [(OAc)_3_BH]^−^). Furthermore, B(3,5‐Cl_2_C_6_H_3_)_3_ is effective for the reductive amination of carbonyls and both aryl and alkylamines without requiring any inert atmosphere techniques or solvent/reagent purification (Scheme 1 D).

## Results and Discussion

To determine if H_2_O–BPh_3_ protonates alkylamines, BnNH_2_ (conjugate acid p*K*_a_=16.6 in MeCN)^8^ was added to a solution of H_2_O–BPh_3_ in [D_3_]‐MeCN. ^1^H NMR spectroscopy showed coordination of BnNH_2_ to BPh_3_, as indicated by a 2H integral resonance at *δ=*5.3 ppm (for BnN*H*_2_) shifted downfield from free BnN*H*_2_ in [D_3_]‐MeCN (1.5 ppm). Identical ^1^H NMR resonances are observed for Ph_3_B–N(H)_2_Bn formed under anhydrous conditions in [D_3_]‐MeCN (for both *δ*_11B_=−1.7 ppm). Coordination of BnNH_2_ to BPh_3_ is reversible at room temperature as addition of benzaldehyde led to rapid imine formation, thus the absence of any observable [HO–BPh_3_]^−^ is attributed to the lower Brønsted acidity of H_2_O–BPh_3_. In contrast, the addition of BnNH_2_ to H_2_O–B(C_6_F_5_)_3_ led to formation of [HO–B(C_6_F_5_)_3_]^−^ as the major product (by ^11^B and ^19^F NMR spectroscopy) as expected based on relative p*K*_a_ values. With no observable deprotonation of H_2_O–BPh_3_ with BnNH_2_, the utility of BPh_3_ as a catalyst was explored in the reductive amination of benzaldehyde (1.0 equiv) with benzylamine (1.2 equiv), under air using non‐purified BPh_3_, non‐purified solvents, and silane as reductant (Table 1). In this reaction, upon imine formation, water is produced as a by‐product, so both excess (relative to BPh_3_) water and a good Brønsted base (BnNH_**2**_, used in slight excess to favour imine formation) are present in the reaction mixture.

**Table 1:** Initial optimization of BPh_3_‐catalysed reductive amination. 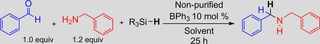

Entry	Solvent	Silane	Equiv. Silane	Temp. [°C]	Yield [%]^[a]^
1	*o*‐DCB	PhMe_2_SiH	1.2	100	<5
2	MeCN	PhMe_2_SiH	1.2	100	33
3	*o*‐DCB	PhMe_2_SiH	3.5	100	<5
4	MeCN	PhMe_2_SiH	3.5	100	87 (80)^[b]^
5^c^	MeCN	PhMe_2_SiH	3.5	100	35
6	MeCN	PhMe_2_SiH	3.5	60	6
7	MeCN	Ph_2_SiH_2_	3.5	100	86
8	MeCN	Ph_2_MeSiH	3.5	100	8
9	MeCN	PhMeSiH_2_	3.5	100	55
10	MeCN	PhSiH_3_	3.5	100	56

Reactions performed in sealed tubes. [a] Yield by ^**1**^H NMR spectroscopy versus mesitylene as internal standard. [b] Isolated yield. [c] Reaction at 5 mol % catalyst loading.

For a direct comparison with our previous work using B(C_6_F_5_)_3_,^5^ we initially performed the reaction in *ortho*‐dichlorobenzene (*o*‐DCB) using 1.2 equivalents of silane. Under these conditions imine formation proceeds but no reduction was observed using 10 % mol BPh_3_ (Table 1, entry 1). Okuda and co‐workers reported that BPh_3_ is a more effective catalyst for (de)hydrosilylation reactions in polar solvents such as MeCN or nitromethane.^13^ Changing the solvent from *o*‐DCB to MeCN now resulted in the desired product being obtained in moderate yield. On increasing the amount of silane from 1.2 to 3.5 equivalents, dibenzylamine was obtained in good yield (87 % NMR yield and 80 % isolated yield). The requirement for excess silane is due to imine reduction and H_2_O/silanol dehydrosilylation occurring concurrently. The activity of this system is not due to initial consumption of all H_2_O by excess silane and then imine reduction proceeding under anhydrous conditions as indicated by the absence of any induction period in this reductive amination. This was further confirmed by analysis of the reaction mixture after 3 hours at 100 °C, at which point considerable imine reduction had occurred (ca. 30 %) but significant water and PhMe_2_SiOH were still present.^20^ Decreasing the catalyst loading to 5 mol % resulted in a lower yield (entry 5), while 100 °C was found to be critical (entry 6). The applicability of other silanes was then investigated: while Ph_2_SiH_2_ was viable in the reductive amination (entry 7), the increase in the steric hindrance of the silane going from PhMe_2_SiH to Ph_2_MeSiH, resulted in a significant drop in imine reduction (entry 4 vs. 8). When smaller silanes were employed (entries 9 and 10), dibenzylamine was the major component among multiple products, including EtNH_2_ presumably deriving from MeCN reduction.

With the compatibility of BnNH_2_ and H_2_O–BPh_3_ mixtures confirmed by the successful reductive amination of benzaldehyde and BnNH_2_, a direct comparison between B(C_6_F_5_)_3_ and BPh_3_ was performed. In our previous work we found that B(C_6_F_5_)_3_ catalysed reductive aminations of anilines and aldehydes in *o*‐DCB at 100 °C, but not the more basic alkylamines due to irreversible deprotonation of H_2_O–B(C_6_F_5_)_3_.^5^ To avoid any disparities arising from the solvent employed, comparative reductive aminations using benzaldehyde and aniline or benzylamine with B(C_6_F_5_)_3_ or BPh_3_ as catalyst were performed in MeCN (Table 2). Although the coordination of MeCN to B(C_6_F_5_)_3_ is well documented,^21^ the reductive amination of benzaldehyde and aniline still proceeded to high yield (96 %) in 1 h at 100 °C on replacing *o*‐DCB with MeCN. As previously reported, 1.2 equivalents of silane is sufficient using anilines with imine reduction occurring preferentially to water dehydrosilylation. Interestingly, on replacing B(C_6_F_5_)_3_ with BPh_3_ under identical conditions, minimal (8 %) imine reduction and minimal water dehydrosilylation were observed after 1 h on heating at 100 °C. A similar outcome was observed using 0.1 equivalent BPh_3_ loading and 3.5 equivalents of silane (entry 2) with a low reductive amination conversion even after 25 h. In contrast, in the reductive amination of benzaldehyde/benzylamine under identical conditions the use of BPh_3_ results in an excellent conversion, whilst B(C_6_F_5_)_3_ is effectively inactive (entry 3).

**Table 2:** Reductive amination catalysed by BPh_3_ or B(C_6_F_5_)_3_. 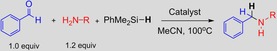

Entry	R	Mol % Catal.	Equiv. silane	Time [h]	Yield [%]^[a]^ B(C_6_F_5_)_3_ BPh_3_	
1	Ph	5	1.2	1	96	8
2	Ph	10	3.5	25	>96	35
3	Bn	10	3.5	25	<5	87

Reactions performed in sealed tubes. [a] Yield by ^1^H NMR spectroscopy versus mesitylene.

Notably, during reductive aminations using BPh_3_ as catalyst four‐coordinate boron species (such as imine→BPh_3_ and amine→BPh_3_) and ^11^B resonances consistent with Ph_2_BOH and PhB(OH)_2_ are all observed. Importantly, attempts to catalyse the reductive amination of benzaldehyde/benzylamine with PhB(OH)_2_, Ph_2_B(OH) or Ph_3_BOH^−^ (whilst not observed the latter is feasibly present in low concentration through a small degree of H_2_O–BPh_3_ deprotonation) in place of BPh_3_ led to very low conversions (e.g., ca. 10 % using Ph_2_BOH) after 25 h at 100 °C in MeCN. The use of Brønsted acids such as HCl and HNO_3_ also resulted in minimal reductive amination. Combined these control reactions indicate the importance of the triarylborane as the catalyst in this process, presumably for activation of the silane via established (for B(C_6_F_5_)_3_) mechanistic pathways.^22^

**Figure 1:**
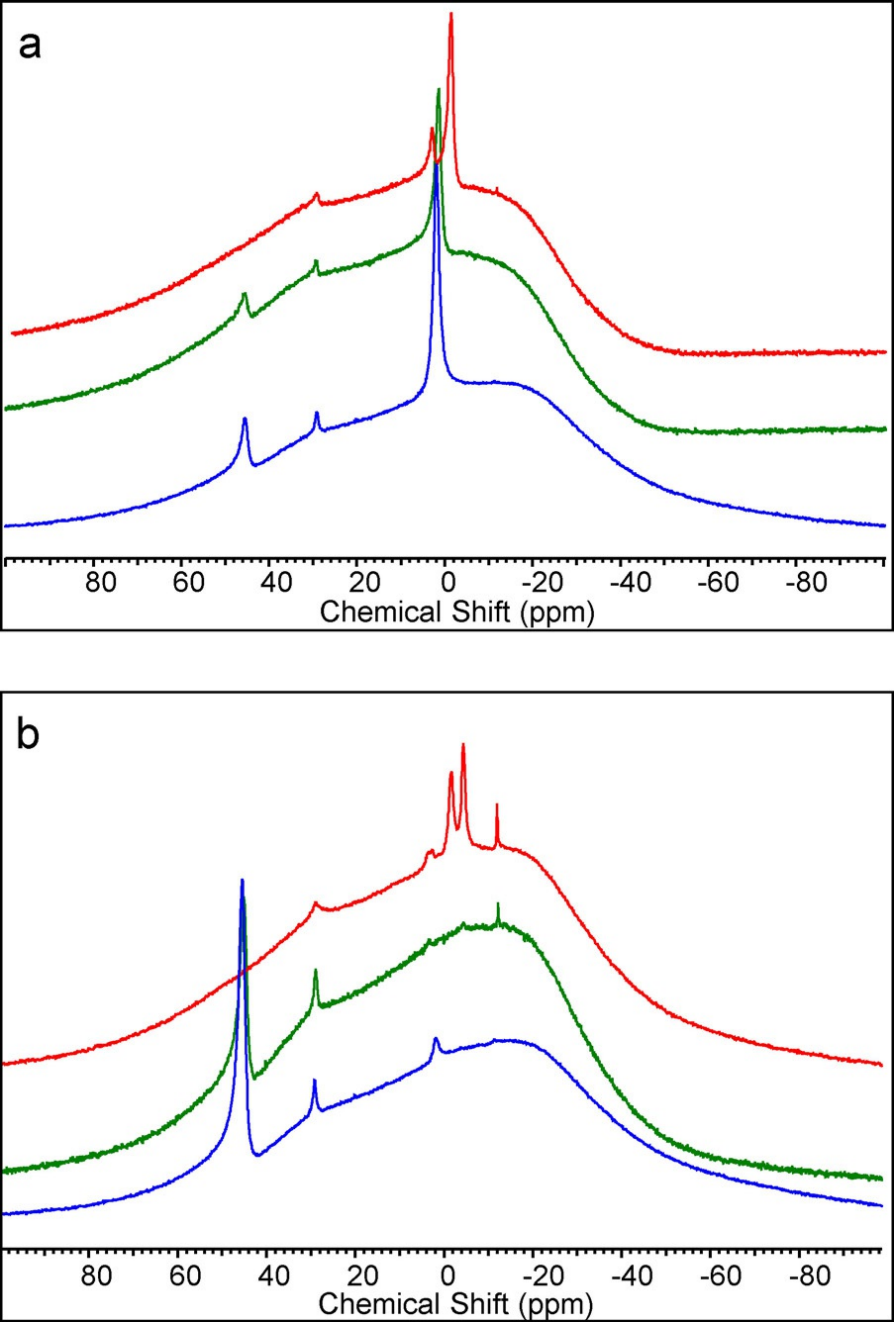
^11^B NMR spectra of H_2_O–BPh_3_ or H_2_O–BPh_3_/amine 1:10:10 immediately on mixing (top) and after heating at 100 °C for 20 h (bottom) in [D_3_]‐MeCN. Blue (no amine), green (+PhNH_2_), red (+BnNH_2_).

To better understand the disparities between PhNH_2_ and BnNH_2_ in reductive aminations catalysed by BPh_3_, a number of control reactions were performed. A solution of BPh_3_ in anhydrous MeCN was heated at 100 °C sealed under air, with no significant reaction (e.g., protodeboronation) observed. However, adding 10 equivalents of water to this solution led to significant protodeboronation after 2 hours at 100 °C (PhB(OH)_2_, Ph_2_B(OH) and PhH observed by ^1^H and ^11^B NMR spectroscopy) presumably via an intramolecular protodeboronation process from H_2_O–BPh_3_ as recently calculated for H_2_O–B(C_6_F_5_)_3_.^23^ Having identified that H_2_O–BPh_3_ can undergo protodeboronation to produce catalytically inactive products the effect of amine basicity on protodeboronation was investigated. The addition of 10 equivalents of PhNH_2_ to a solution of H_2_O–BPh_3_ (made by mixing 1 equivalents of BPh_3_ with 10 equivalents of water in MeCN to approximate the catalysis conditions) did not prevent protodeboronation on heating. Notably, when 10 equivalents of the more basic amine BnNH_2_ was added to an identical solution containing H_2_O–BPh_3_, protodeboronation proceeded to a significantly lower extent (by monitoring the appearance of benzene in the ^1^H NMR spectrum and by ^11^B NMR spectroscopy). Even upon heating at 100 °C for 20 hours (Figure 1) four‐coordinate L→BPh_3_ compounds were still the dominant species with BnNH_2_ in contrast to that with PhNH_2_.

**Scheme 2:**
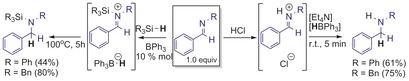
*N*‐benzylidene amines reduction.

The disparity between PhNH_2_ and BnNH_2_ in reductive amination catalyzed by BPh_3_ will be due to different amine (or imine) basicity, however this will affect a number of processes, therefore to identify the origin of this disparity a number of control reactions were performed. The disparity is not due to the less nucleophilic imine derived from aniline/benzaldehyde leading to a significantly greater barrier to an S_N_2 type reaction with the R_3_Si–H–BPh_3_ species. This was confirmed by the fact that under anhydrous conditions using catalytic BPh_3_ and stoichiometric PhMe_2_SiH, *N*‐benzylidene aniline and *N*‐benzylidene benzylamine were both reduced (Scheme 2, left). However, under catalytic reductive amination conditions the key electrophile could be the silylated iminium cation (if the BPh_3_ activated silane is directly attacked by the imine) or the protonated iminium cation (via imine protonation by [R_3_Si–OH_2_][HBPh_3_] formed from initial attack by H_2_O on R_3_Si–H–BPh_3_). Although no silylated amine was observed during reductive amination, the exact nature of the iminium cation could not be unambiguously defined in this process due to the fast hydrolysis of silylated amine under these conditions. Nevertheless, further control reactions showed that both protonated *N*‐benzylidene aniline and *N*‐benzylidene benzylamine were reduced by [HBPh_3_]^−^ (consistent with Okuda and co‐workers report on imine hydroboration catalyzed by [HBPh_3_]^−^ salts)_._ 24 There was no evidence for differing degrees of side reactions (such as evolution of PhH (by protodeboronation)) or significant differences in the rate of reduction during the control reactions with the iminium cations (Scheme 2, right). Whilst the iminium cations derived from *N*‐benzylidene aniline do undergo slower reductions (than those derived from *N*‐benzylidene benzylamine) this should only result in longer reaction times being required for complete reductive amination using PhNH_2_/benzaldehyde under BPh_3_ catalysis. However, this is not observed, as no further increase in conversion is observed on longer reaction times in reductive aminations. Combined these observations indicate that the difference in reactivity is due to more rapid catalyst decomposition in the presence of PhNH_2_ relative to BnNH_2_ and not any intrinsic barrier to *N*‐benzylidene aniline reduction.

As BPh_3_ decomposition most probably proceeds via H_2_O–BPh_3_ (based on its fast protodeboronation), reducing the concentration of this species in solution should be key to provide enhanced catalytic activity. At least two scenarios are feasible for achieving this: i) the more basic species (BnNH_2_ or its derived imine) retards protodeboronation by deprotonating H_2_O–BPh_3_ resulting in a different catalyst resting state, [HO–BPh_3_]^−^_,_ that is more stable to protodeboronation; ii) the more nucleophilic amine/imine (e.g., BnNH_2_ or its derived imine) forms a Lewis adduct L→BPh_3_, which is more stable to protodeboronation than Ph_3_B–OH_2_. Based on the in situ NMR data for H_2_O–BPh_3_/BnNH_2_ the latter is more probable as only Bn(H)_2_ n–BPh_3_ is observed with no [Ph_3_B–OH]^−^ detectable. In contrast, with the less basic/nucleophilic aniline, the adduct Ph(H)_2_ n–BPh_3_ (which when formed under anhydrous conditions has a characteristic integral 2H singlet in the ^1^H NMR spectrum at *δ*=5.7 ppm for the NH_2_ group) reacts with equimolar water as indicated by a drastic shift in the ^1^H NMR spectrum to a broad resonance at *δ*=2.1 ppm (integral four for the combined NH_2_/OH_2_ resonance). This suggests an equilibrium between Ph(H)_2_ n–BPh_3_ and H_2_O–BPh_3_ consistent with the more rapid protodeboronation observed. The ^11^B NMR spectra are inconclusive for this system as H_2_O–BPh_3_ and Ph_3_B–N(H)_2_Ph have extremely similar chemical shifts, whilst the slow exchange regime is not reached even at −38 °C in [D_3_]‐MeCN.

**Scheme 3:**
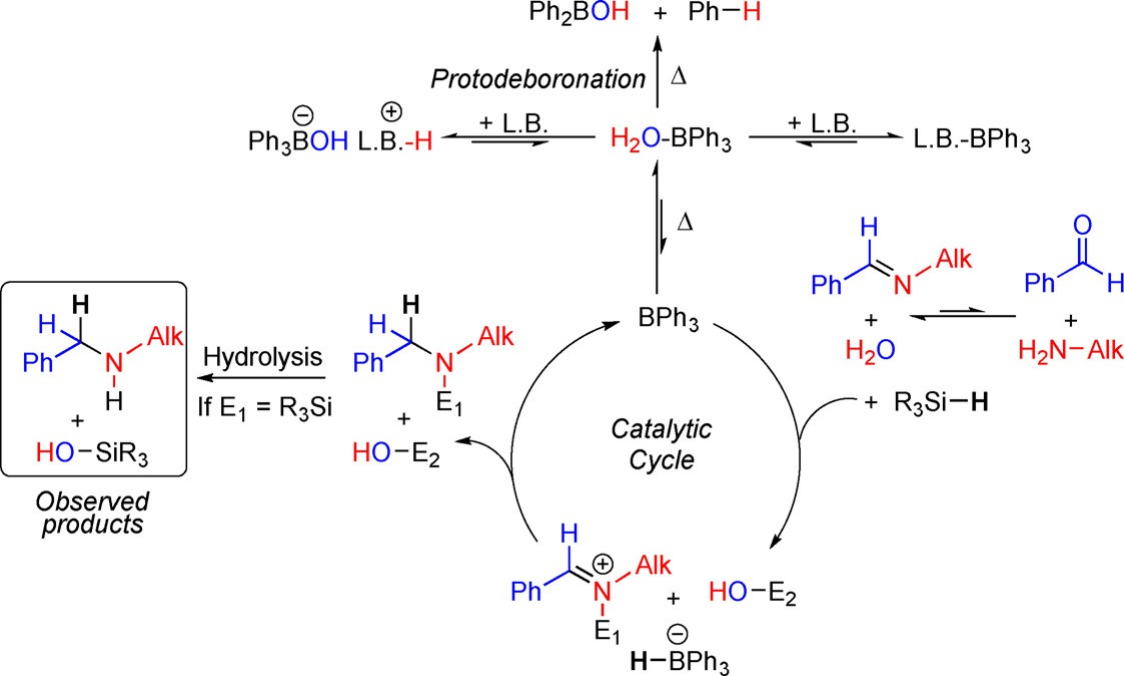
Feasible key reactions in reductive amination reaction mixtures. L.B.=Lewis bases. E_1_=H, E_2_=R_3_Si or E_1_=R_3_Si, E_2_=H.

With the disparity between BnNH_2_ and PhNH_2_ in reductive aminations catalyzed with BPh_3_ clarified, we next investigated the highly Brønsted basic but less nucleophilic amine *t*BuNH_2_. Significantly, *t*BuNH_2_ and PhNH_2_ have similar Mayr nucleophilicity values in MeCN (*N*=12.35 and 12.64, respectively),^25^ but the conjugate acid of *t*BuNH_2_ has a p*K*_a_ of 18.4. Under standard conditions (3.5 equiv. silane, 10 mol % BPh_3_, MeCN), the reductive amination of *t*BuNH_2_ and benzaldehyde proceeded to a 93 % conversion after 25 h at 100 °C. Again the ^11^B NMR spectrum after 25 h was dominated by four‐coordinate boron species with minimal PhB(OH)_2_ and Ph_2_B(OH) observed. To investigate the origin of the enhanced stability of BPh_3_ in the presence of *t*BuNH_2_, the ^1^H and ^11^B NMR spectra of BPh_3_/*t*BuNH_2_/H_2_O mixtures was examined, which revealed broad resonances at 25 °C, (e.g., a ^1^H resonance at *δ*=3.7 ppm) shifted downfield with respect to *t*BuN*H*_2_ and *H*_2_O–BPh_3_ (*δ*=1.3 and 2.6 ppm, respectively). Cooling this solution to below −10 °C resulted in the appearance of *t*BuN(H)_2_–BPh_3_, however, this was a minor component (ca. 10 %). The major resonance in the ^1^H NMR spectrum was still broad with a chemical shift not consistent with H_2_O–BPh_3_ or free *t*BuNH_2_, instead it is assigned as H_2_O–BPh_3_ and [HOBPh_3_][H_3_N*t*Bu] in fast exchange, a process which was not frozen out at −38 °C in [D_3_]‐MeCN. Based on these observations feasible key processes occurring in situ in the reductive amination reactions are summarised in Scheme 3.

Upon heating, enough BPh_3_ is generated from a Lewis adduct or the hydroxyborate to activate the silane to nucleophilic attack. Nucleophilic attack leads to the formation of [HBPh_3_]^−^ that in turn would reduce the iminium cation (either silylated or protonated) by hydride transfer thus regenerating the catalyst. The protodeboronation pathway deactivates the catalyst, and is a process which most probably proceeds from H_2_O–BPh_3_. The concentration of this species can be minimized in solution by using stronger bases/nucleophiles which lead to formation of LB→BPh_3_ or [LB–H][HOBPh_3_] (LB=amine or imine). Notably, in the presence of both BnNH_2_ and *N*‐benzylidene benzylamine, BPh_3_ binds the former preferentially. As the optimal catalysis conditions uses a slight excess of amine, the continued presence of free amine presumably helps reduce the quantity of H_2_O–BPh_3_ present and thus limit protodeboronation.

With an understanding of the limitations of using BPh_3_ for catalytic reductive amination, the substrate scope was then explored with the reactions performed under air, using non‐purified solvent and reactants with everything combined at the start in an operationally simple process (Table 3).

**Table 3:** Substrates screening of the reductive amination.

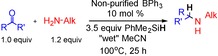
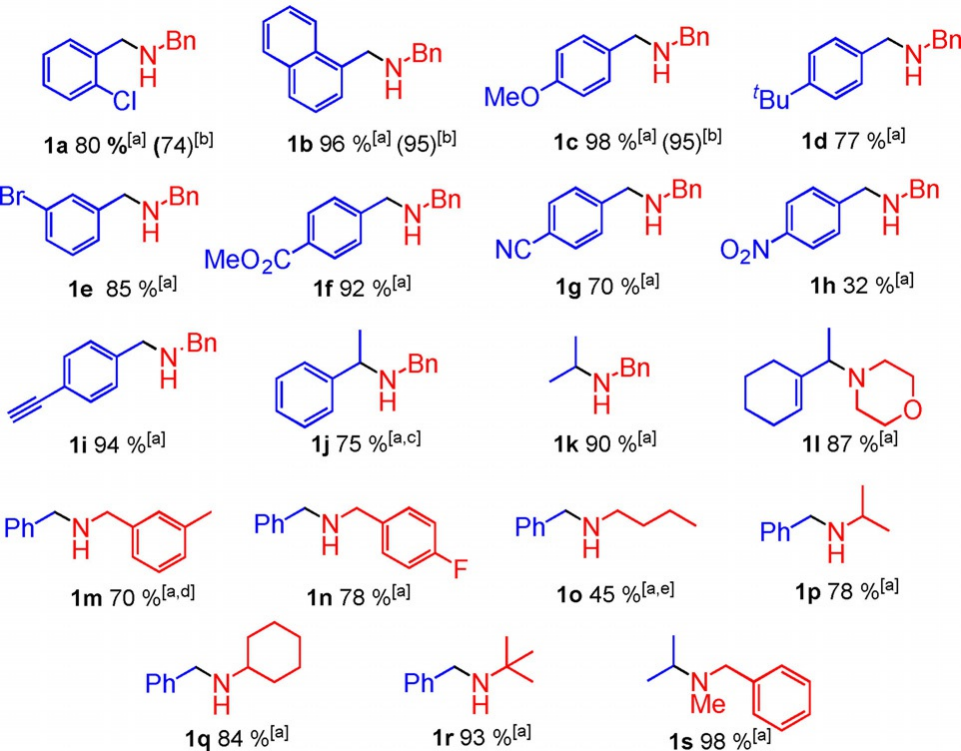

Reactions performed in sealed tubes. [a] ^1^H NMR yields versus mesitylene. [b] Isolated yield. [c] 48 h. [d] 40 h. [e] 30 h.

**Scheme 4:**
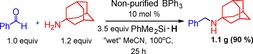
Gram‐scale synthesis of *N*‐benzyl‐1‐adamantylamine.

A range of functionalised benzaldehydes were amenable in the reductive amination with benzylamine, with good in situ conversions and isolated yields (**1 a**–**e**). It is noteworthy that ester and cyano substituents were compatible, with no evidence for their reduction under these conditions (**1 f**, **g**). However, the reaction was less tolerant to nitro substituents (due to *trans*‐imination and formation of dibenzylamine observed as the major by‐product). It is noteworthy that when electron‐withdrawing groups are present in the *para* position of benzaldehyde (e.g. ‐CO_2_Me or ‐CN), minimal siloxane (and silanol) were observed after 25 h (by ^1^H and ^29^Si NMR spectroscopy), with significant reduction of the imine still occurring. Furthermore >50 % imine reduction to **1 f** was observed with only 1.2 equivalents of silane after 25 h. This indicates that more electrophilic imines effectively out compete H_2_O for reaction with the borohydride, whereas with less electrophilic imines the rates of water/silanol dehydrosilylation and iminium cation reduction are comparable hence excess silane is required. Reductive amination also proceeded in the presence of a terminal C−C triple bond without significant reduction of the latter (**1 i**), or any observable side reactivity, for example, dehydroboration.1d When aliphatic aldehydes (n‐butyraldehyde and propionaldehyde) were used, full consumption of the in situ formed imine was observed, but the desired product was only a minor component due to over‐alkylation to the tertiary amine or enamine isomerization reactions, as reported for B(C_6_F_5_)_3_.^5^ However, when ketones were utilised, the reaction was successful, allowing a secondary carbon centre to be attached to the nitrogen (**1 j**,**k**). Notably, the reductive amination of acetophenone and benzylamine is challenging with widely used reducing agents such as Na[triacetoxyborohydride] (Na[(OAc)_3_BH], 55 % yield after 10 days),^26^ in contrast using BPh_3_/silane **1 j** is produced in higher yield in shorter reaction times. The reductive amination of 1‐acetyl‐1‐cyclohexene and morpholine to yield **1 l** is also challenging using [(OAc)_3_BH]^−^ (only 10 % yield after 4 days),^26^ but it proceeds to 87 % yield using BPh_3_/silane. This demonstrates that the BPh_3_‐catalysed process is applicable to systems where established borohydride reductive amination approaches struggle. Furthermore, the formation of **1 l** shows the compatibility of this methodology with C−C double bonds. The inclusion of substituents on benzylamine, as well as the use of *n*BuNH_2_ as another C‐primary amine, was also realized (e.g. **1 m**–**o**), although using the latter amine over‐alkylation also occurred to some extent (e.g. forming *n*Bu_2_NBn). C‐secondary amines, such as *cyclo*‐hexylamine and isopropylamine, or a C‐tertiary amine *t*BuNH_2_, gave good conversions to the desired products (**1 p**–**r**). It is noteworthy that a common product could be formed from a different combination of aldehyde/amine (e.g. **1 k** and **1 p**), offering two retrosynthetic strategies. Finally, when a secondary amine such as BnN(H)Me was used in combination with an enolizable ketone the reaction still proceeds successfully to form **1 s** in excellent yield. It should be emphasized that these amines are not accessible by reductive amination using B(C_6_F_5_)_3_ as catalyst due to it being limited to aniline derivatives. To demonstrate scalability the reductive amination of benzaldehyde and 1‐adamantylamine was performed on gram‐scale under air, using 10 mol % of unpurified BPh_3_ in non‐purified acetonitrile and using PhMe_2_SiH as reductant (Scheme 4). Combining all the reactants at the start and heating the reaction mixture at 100 °C for 25 hours enabled the desired product to be isolated in a 90 % isolated yield (1.1 g).

**Figure 2:**
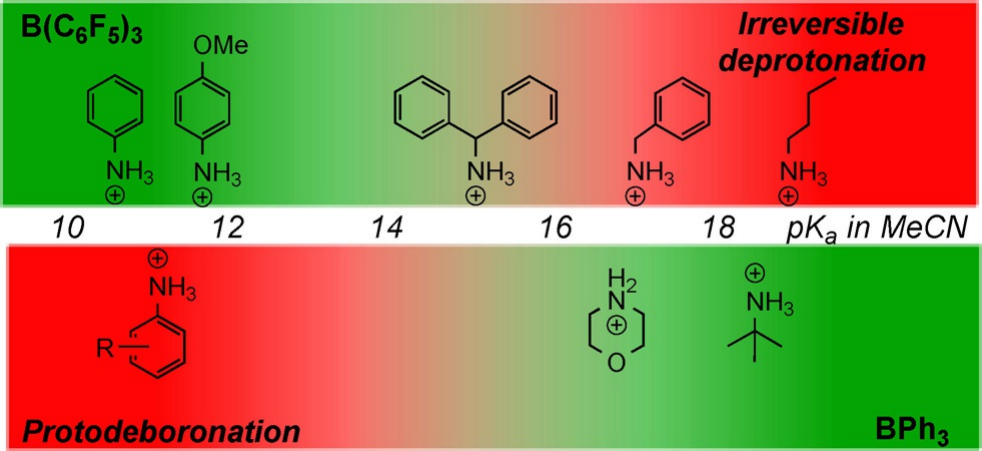
Water/amine tolerance of B(C_6_F_5_)_3_ and BPh_3_ under the reductive amination reaction conditions.

The results discussed above indicate that B(C_6_F_5_)_3_ and BPh_3_ have complementary tolerance to water/amine combinations in reductive aminations (Figure 2). B(C_6_F_5_)_3_ is a viable catalyst for aryl amines (conjugate acids p*K*_a_<12 in MeCN) but not alkylamines (conjugate acids p*K*_a_>16 in MeCN) due to irreversible deprotonation of H_2_O–B(C_6_F_5_)_3_ with the latter. In contrast, BPh_3_ is a viable reductive amination catalyst for alkylamines but not arylamines due to more rapid protodeboronation in the presence of the latter. We were thus interested in exploring an amine with an intermediate p*K*_a_, specifically the reductive amination of benzaldehyde and benzhydrylamine (conjugate acid p*K*_a_ 15 in MeCN)^27^ was performed with both these boranes using 10 mol % catalyst loading. In all cases the in situ conversions were only moderate at best (less than 30 %) under a range of conditions with both boranes (e.g., in MeCN or *o*‐DCB at 100 °C), indicating that an amine whose conjugate acid has a p*K*_a_ between 12–16 is particularly challenging for both boranes. Again in situ analysis revealed that with BPh_3_ significant protodeboronation proceeded upon heating (by ^11^B NMR spectroscopy), whilst with B(C_6_F_5_)_3_ the deactivation was due to the effectively irreversible deprotonation of H_2_O–B(C_6_F_5_)_3_ (by ^11^B/^19^F NMR spectroscopy).

**Scheme 5:**
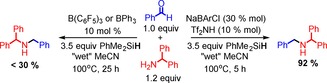
Reductive amination with benzaldehyde and benzylhydrylamine using B(C_6_F_5_)_3,_ BPh_3_ or B(3,5‐C_6_H_3_Cl_2_)_3_ (generated in situ) as catalyst.

Given the respective limitations of B(C_6_F_5_)_3_ and BPh_3_, a single triarylborane that is a viable catalyst for the reductive amination of both aryl and alkyl amines (including benzhydrylamine) was targeted. To have a broad amine scope, the triarylborane must form a H_2_O–BAryl_3_ adduct that is both more resistant to protodeboronation than H_2_O–BPh_3_ and less Brønsted acidic than H_2_O–B(C_6_F_5_)_3_. Furthermore, a triarylborane that does not contain *ortho*‐halogen aryl substituents is desirable, as *ortho* substituents increase the steric bulk around boron and thus can significantly hinder amine/imine coordination to boron.^12^ The latter is actually desired in this process as it reduces the concentration of H_2_O–BAryl_3_ in solution, thus also helping to limit protodeboronation. Given these requisites B(3,5‐Cl_2_C_6_H_3_)_3_ was selected and its synthesis via the protolytic decomposition of its tetraarylborate salt was utilised as the borate salt is air and moisture stable as a solid in contrast to the free triarylboranes (see subsequent discussion). Tetraarylborate anion decomposition has significant precedence for [BPh_4_]^−^ salts which react with Brønsted acids to release BPh_3_ compounds.^28^ Furthermore, we recently observed decomposition of Na[B(3,5‐Cl_2_C_6_H_3_)_4_] (termed Na[BArCl] herein) in wet solvents on heating. To confirm that Na[BArCl] decomposition by protonolysis generates B(3,5‐Cl_2_C_6_H_3_)_3_ species, the strong Brønsted acid HNTf_2_ was added to NaBArCl. This resulted in the appearance of a major new resonance at *δ*=67 ppm in the ^11^B NMR spectrum assigned as B(3,5‐Cl_2_C_6_H_3_)_3_, with this chemical shift consistent with other reported tri(chloroaryl)boranes.^29^ Applying this in situ B(3,5‐Cl_2_C_6_H_3_)_3_ generation procedure (using an excess of Na[BArCl] relative to HNTf_2_ to preclude any trace Brønsted acid remaining as strong Brønsted acids can also activate Si−H bonds),^30^ B(3,5‐Cl_2_C_6_H_3_)_3_ catalyzed the reductive amination of benzaldehyde and benzhydrylamine to give the desired product in good yield (Scheme 5). The use of both B(C_6_F_5_)_3_ and BPh_3_ as catalysts under these conditions gave low conversions.

**Scheme 6:**
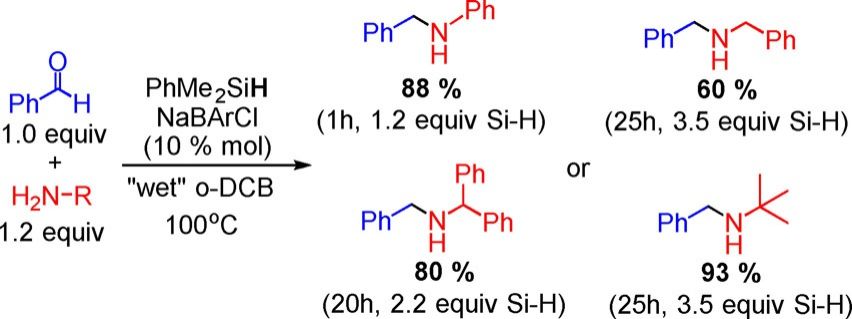
Reductive aminations under air employing Na[BArCl] as precursor catalyst.

Seeking an operationally simpler process, the decomposition of Na[BArCl] by action of H_2_O was investigated as a route to generate B(3,5‐Cl_2_C_6_H_3_)_3_ in situ.^31^, ^32^ This approach was successful for the catalytic reductive amination of benzhydrylamine and benzaldehyde using 10 mol % Na[BArCl] in *o*‐DCB (Scheme 6), with all manipulations performed in air using non‐purified solvent/reagents. Weakly coordinating solvents are essential as attempts using MeCN as solvent led to no reductive amination. The solvent dependency is attributed to the formation of [(H_2_O)_*x*_Na]^+^ species in *o*‐DCB that have enhanced Brønsted acidity (relative to H_2_O) and are thus key to effecting anion protodeboronation and generation of the triarylborane, as previously discussed for NaBPh_4_.^28^ In contrast in MeCN, the solvent is presumably solvating the Na cations, resulting in a less Brønsted acidic solution and no anion protodeboronation.

With an in situ catalyst generation protocol in hand, a brief amine substrate scope exploration was undertaken. Most notably, the triarylborane derived in situ from Na[BArCl] was able to catalyse the reductive amination of benzaldehyde with PhNH_2_, BnNH_2_, and *t*BuNH_2_ amines whose conjugate acids span the p*K*_a_ range from 10.6 to 18.4 in MeCN. This indicates a reduced acidity of the corresponding H_2_O–B(3,5‐Cl_2_C_6_H_3_)_3_ adduct (relative to that of H_2_O–B(C_6_F_5_)_3_) and an improved stability of B(3,5‐Cl_2_C_6_H_3_)_3_ species to protodeboronation (relative to BPh_3_). The amount of silane required for good conversion to the reductive amination product was explored and again found to depend on the imine electrophilicity, with the more electrophilic imine (derived from aniline) reduced using only 1.2 equivalents of silane, whilst the less electrophilic imines again required an excess of silane due to competitive dehydrosilylation reactions.

**Scheme 7:**
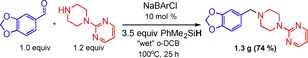
Synthesis of Piridebil by reductive amination.

The ability to use Na[BArCl] as a precursor to the active triarylborane catalyst has practical advantages since it is readily synthesized and is bench stable for at least 6 months. In contrast, whilst BPh_3_ is commercially available its storage as a solid under ambient atmosphere leads to gradual decomposition (even after only 14 days significant PhB(OH)_2_ and Ph_2_B(OH) are observed by ^11^B NMR spectroscopy). This negatively impacts conversion; for example using pristine BPh_3_ gives 87 % conversion of benzaldehyde and benzylamine to the reductive amination product whereas the same batch of BPh_3_ stored as a solid in air for 2 weeks results in only 52 % conversion when used as the catalyst under otherwise identical conditions. In contrast, Na[BArCl] stored as a solid for 6 months in air shows no deterioration in reductive amination catalytic activity. Thus Na[BArCl] is a useful bench‐stable catalyst precursor for reductive aminations, with its utility further demonstrated in the rapid synthesis of the more complex drug molecule Piribedil (used in the treatment of Parkinson's disease)^33^ in good yield (Scheme 7) under air using non‐purified reagents/solvents.

## Conclusions

In summary, BPh_3_ has a higher tolerance to H_2_O and alkylamine combinations than B(C_6_F_5_)_3_, due to the lower Brønsted acidity of H_2_O–BPh_3_. This extends the water/base tolerance of FLP systems to strong bases (conjugate acid p*K*_a_=18.5). This enables the utilisation of BPh_3_ as a catalyst for the reductive amination of aldehydes and ketones with many different aliphatic amines, ranging from C‐primary to C‐tertiary. This system is even effective for the reductive amination of substrates that are challenging with conventional borohydrides (e.g., [(OAc)_3_BH]^−^). BPh_3_ and B(C_6_F_5_)_3_ exhibit complementary amine scope in reductive aminations, with the former limited by the protodeboronation of H_2_O–BPh_3_ in the presence of weaker amine Brønsted bases/nucleophiles, while the latter is limited by H_2_O–B(C_6_F_5_)_3_ undergoing irreversible deprotonation by stronger Brønsted basic amines such as alkylamines. Finally, a third triarylborane, B(3,5‐Cl_2_C_6_H_3_)_3_, of intermediate Lewis acidity, was shown to be effective for the reductive amination of a range of amines whose conjugate acids span p*K*_a_ values of 10.6 to 18.5 in MeCN. Furthermore, in situ tetraarylborate anion decomposition by H_2_O in non‐coordinating solvents represents a simple route to generate the active triarylborane catalyst from a readily accessible bench‐stable precursor. The reductive amination methodologies presented herein are operationally simple (e.g. no purification of any materials/solvent is required and the reactions are performed under air) and are applicable to gram‐scale and complex molecule synthesis.

## Supporting information

As a service to our authors and readers, this journal provides supporting information supplied by the authors. Such materials are peer reviewed and may be re‐organized for online delivery, but are not copy‐edited or typeset. Technical support issues arising from supporting information (other than missing files) should be addressed to the authors.

Supplementary
