# Supplementary material for: Expanding Water/Base Tolerant Frustrated Lewis Pair Chemistry to Alkylamines Enables Broad Scope Reductive Aminations
Source: Chemistry. 2017 Jan 18;23(9):2217–24. doi: 10.1002/chem.201605466 (PMC5396349; doi:10.1002/chem.201605466)

CHEMISTRY
A European Journal

Supporting Information

Expanding Water/Base Tolerant Frustrated Lewis Pair Chemistry to Alkylamines Enables Broad Scope Reductive Aminations

Valerio Fasano and Michael J. Ingleson*[a]

chem_201605466_sm_miscellaneous_information.pdf

Supporting Information for:

Expanding Water/Base Tolerant FLP Chemistry to Alkylamines Enables Broad Scope Reductive Aminations

*Valerio Fasano and Michael J. Ingleson**

Michael.ingleson@manchester.ac.uk

School of Chemistry, University of Manchester, Manchester, M13 9PL, U.K.

Table of Contents

1. General Remarks 2

2. Borane adducts with water and amines/imines 3

3. Decomposition of $Ph_3B\text{-}OH_2$ in the presence of amines 14

4. Initial screening of reaction conditions 17

5. Screening of the hydrosilanes as reductant 21

6. Imine consumption *vs* water dehydrosilylation 26

7. Reductive amination benzaldehyde/aniline with $B(C_6F_5)_3$ and BPh_3 27

8. Hydrosilylation of *N*-benzylidene aniline and *N*-benzylidene benzylamine 29

9. Reduction of protonated imine with $[Et_4N][HBPh_3]$ 31

10. Attempted imine reduction with Ph_2BOH, $PhB(OH)_2$ and Ph_3BOH^- as catalyst 33

11. Attempted reductive amination using HNO_3 and HCl as catalysts 36

12. Carbonyl substrate scope 38

13. Amine substrate scope 43

14. Synthesis of *N*-benzyl-1-adamantylamine 46

15. Reductive amination benzaldehyde/benzhydrylamine with $B(C_6F_5)_3$ and BPh_3 47

16. Reductive aminations using NaBArCl as catalyst precursor 49

17. Synthesis of Piribedil 56

18. BPh_3 decomposition sealed under air 57

19. References 58

20. NMR and GC-MS data of all compounds 59

1. General Remarks

Unless otherwise indicated all manipulations were conducted under ambient conditions. Unless otherwise indicated, all compounds and solvents were purchased from commercial sources and used as received unless otherwise stated. BPh_3 was bought and kept under argon to prevent decomposition. NaBArcl was produced using the previously published procedure.[1] $[Et_4N][HBPh_3]$ was synthesised by Mr John McGough from $Na[HBPh_3]$ and $[Et_4N]I$ in accordance with the literature.[2] Solvents for column chromatography were of technical grade and used without further purification. Column chromatography was performed on silica gel (230-400 mesh). NMR spectra were recorded with a Bruker AV-400 spectrometer (400 MHz ^{1}H; 100 MHz ^{13}C; 128 MHz ^{11}B; 376 MHz ^{19}F; 79 MHz ^{29}Si). ^{1}H NMR chemical shifts are reported in ppm relative to *protio* impurities in the deuterated solvents and ^{13}C NMR using the solvent resonances unless otherwise stated. ^{11}B NMR spectra were referenced to external $BF_3{:}Et_2O$, ^{19}F to Cl_3CF and ^{29}Si to $Si(CH_3)_4$. Coupling constants J are given in Hertz (Hz), while the multiplicity of the signals are indicated as "s", "d", "t" "pent", "sept" or "m" for singlet, doublet, triplet, pentet, septet or multiplet, respectively. Mesitylene (distilled from K) was used as an internal standard to determine the NMR yields. GC-MS analysis was performed on an Agilent Technologies 7890A GC system equipped with an Agilent Technologies 5975C inert XL EI/CI MSD with triple axis detector. The column employed was an Agilent J&W HP-5ms ((5%-Phenyl)-methylpolysiloxane) of dimensions: length, 30 m; internal diameter, 0.250 mm; film, 0.25 μm. Mass spectra were recorded on a Waters QTOF mass spectrometer.

2. Boranes adducts with water and amines

BPh_3 in MeCN: A J. Youngs NMR tube equipped with d_6-DMSO capillary was charged with BPh_3 (7.0 mg, 0.028 mmol, 1.0 eq.) in distilled acetonitrile (0.5 mL). Upon heating at 100°C for 20 hours, minimal decomposition was observed, with Ph_3B-MeCN adduct observed as the main species.

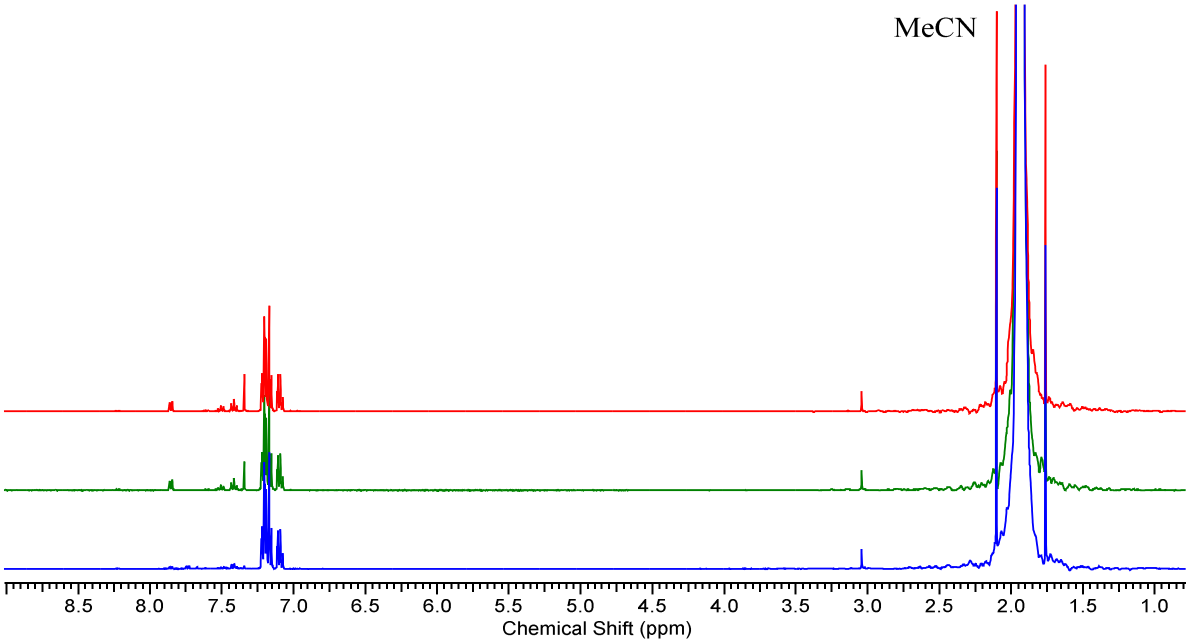

Figure S1. *In situ* ^{1}H-NMR spectra of BPh_3 in acetonitrile. Blue (t = 5 min, r.t.), green (after t = 3 hours at 100°C), red (after 20 hours at 100°C).

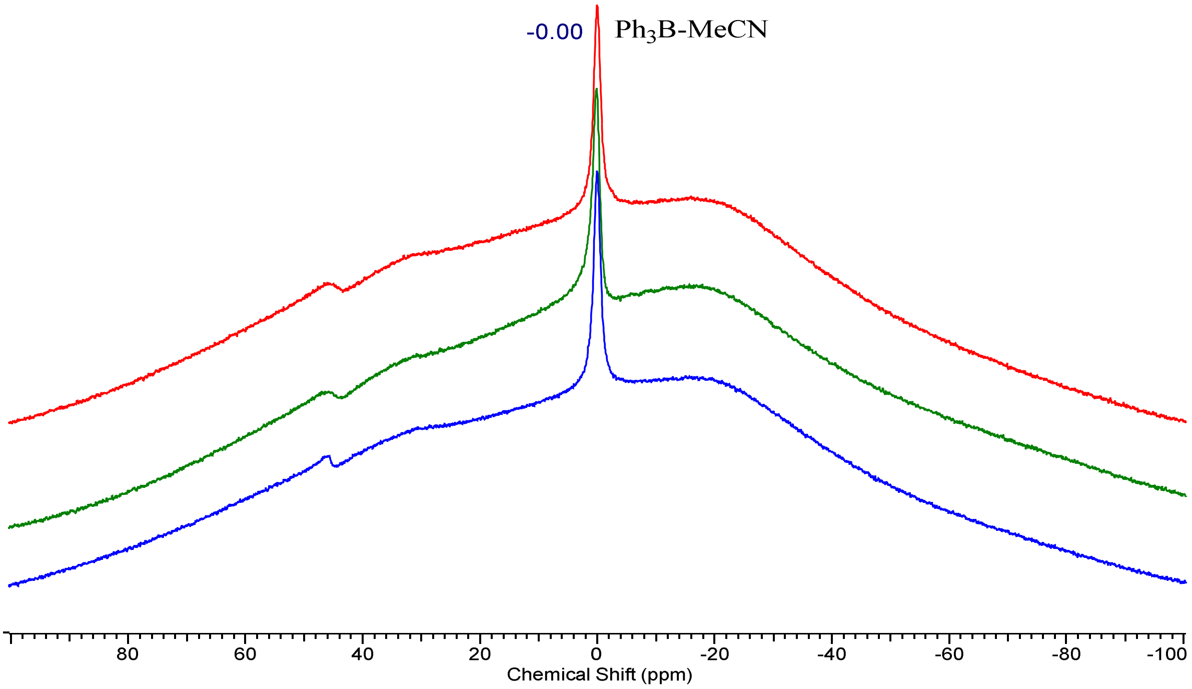

Figure S2. *In situ* ^{11}B-NMR spectra of BPh_3 in acetonitrile. Blue (t = 5 min, r.t.), green (after t = 3 hours at 100°C), red (after 20 hours at 100°C). Impurity at 47 ppm is Ph_2BOH present in commercial material which does not increase on prolonged heating in the absence of H_2O.

BPh_3/H_2O (1:10): A J. Youngs NMR tube equipped with d_6-DMSO capillary was charged with BPh_3 (11.3 mg, 0.044 mmol, 1.0 eq.) and H_2O (8 µL, 0.445 mmol, 10.0 eq.) in distilled acetonitrile (0.5 mL). Upon heating at 100°C for 3 hours, BPh_3 decomposition was observed.

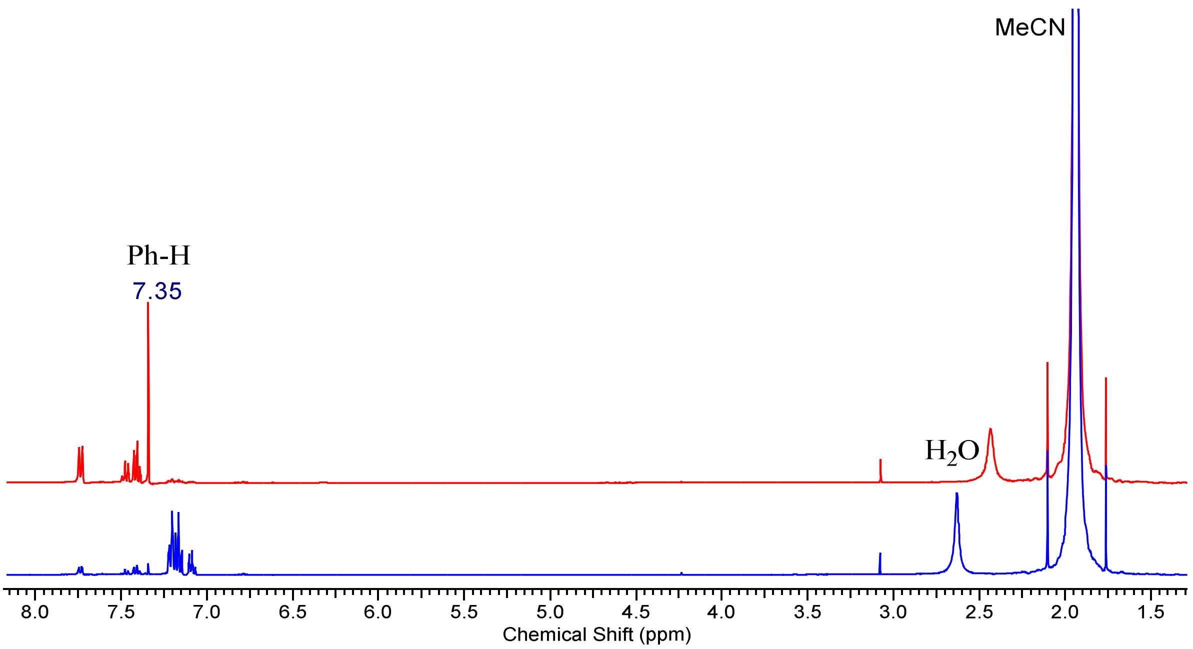

Figure S3. *In situ* ^{1}H-NMR spectra of BPh_3/H_2O (1:10) in acetonitrile. Blue (t = 5 min, r.t.), red (after 3 hours at 100°C).

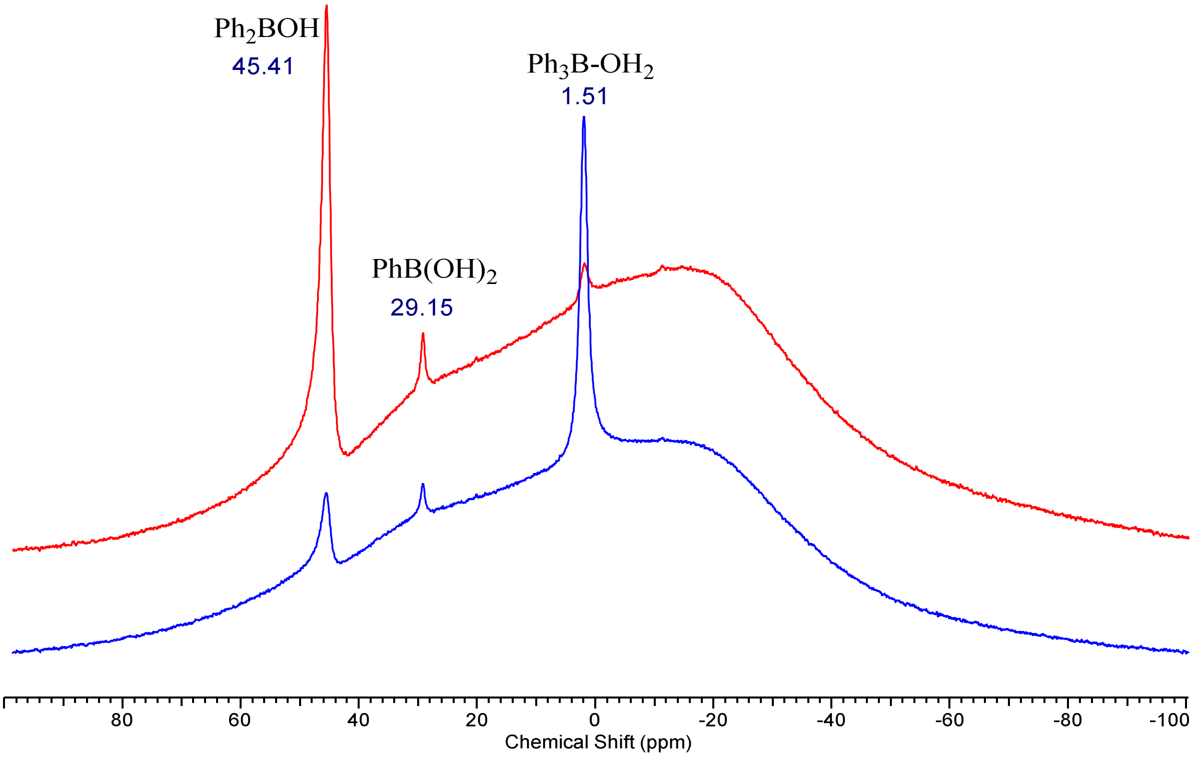

Figure S4. *In situ* ^{11}B-NMR spectra of BPh_3/H_2O (1:10) in acetonitrile. Blue (t = 5 min, r.t.), red (after 3 hours at 100°C).

$BPh_3/H_2O/PhNH_2$ (1:1:1): A J. Youngs NMR tube was charged with BPh_3 (14.0 mg, 0.055 mmol, 1.0 eq.), H_2O (1 µL, 0.055 mmol, 1.0 eq.) and $PhNH_2$ (5 µL, 0.055 mmol, 1.0 eq.) in d_3-acetonitrile (0.5 mL). Both $-NH_2$ and H_2O protons were observed as a broad feature centered at 2.3 ppm.

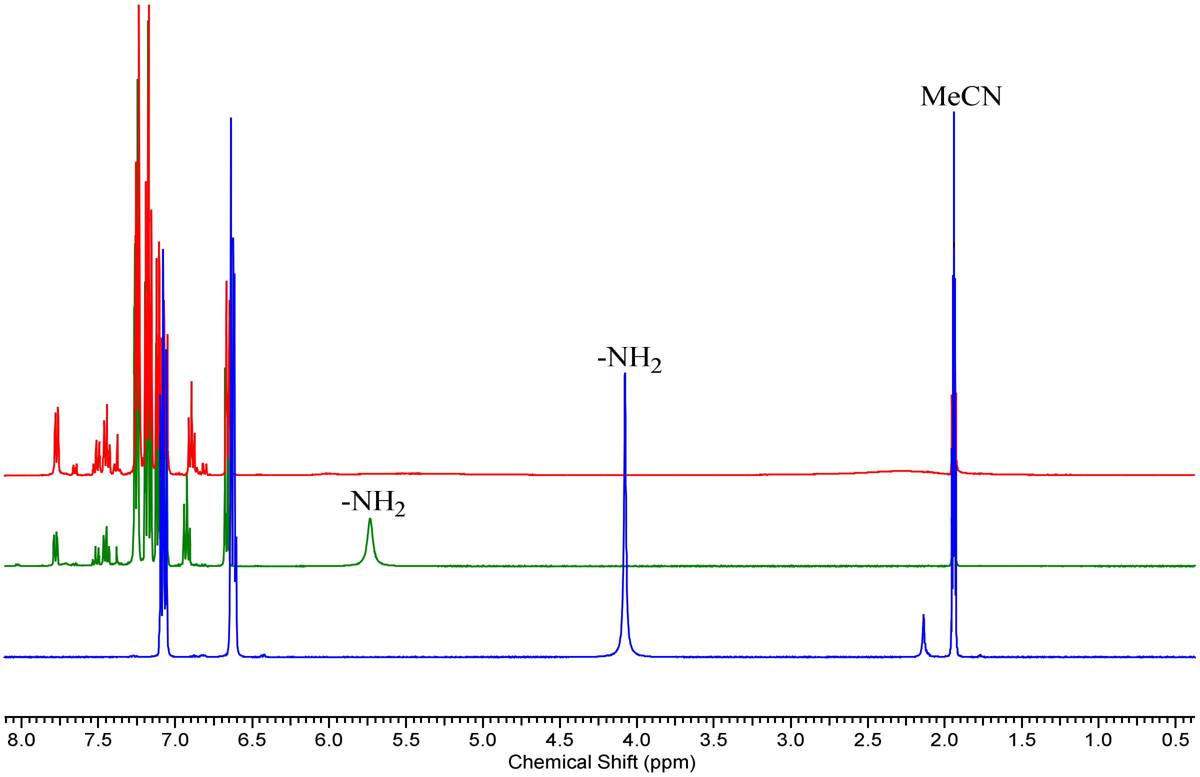

Figure S5. *In situ* ^{1}H-NMR spectra of $PhNH_2$ (blue), $BPh_3/PhNH_2$ 1:1 (green) and $BPh_3/H_2O/PhNH_2$ 1:1:1 (red) in d_3-acetonitrile.

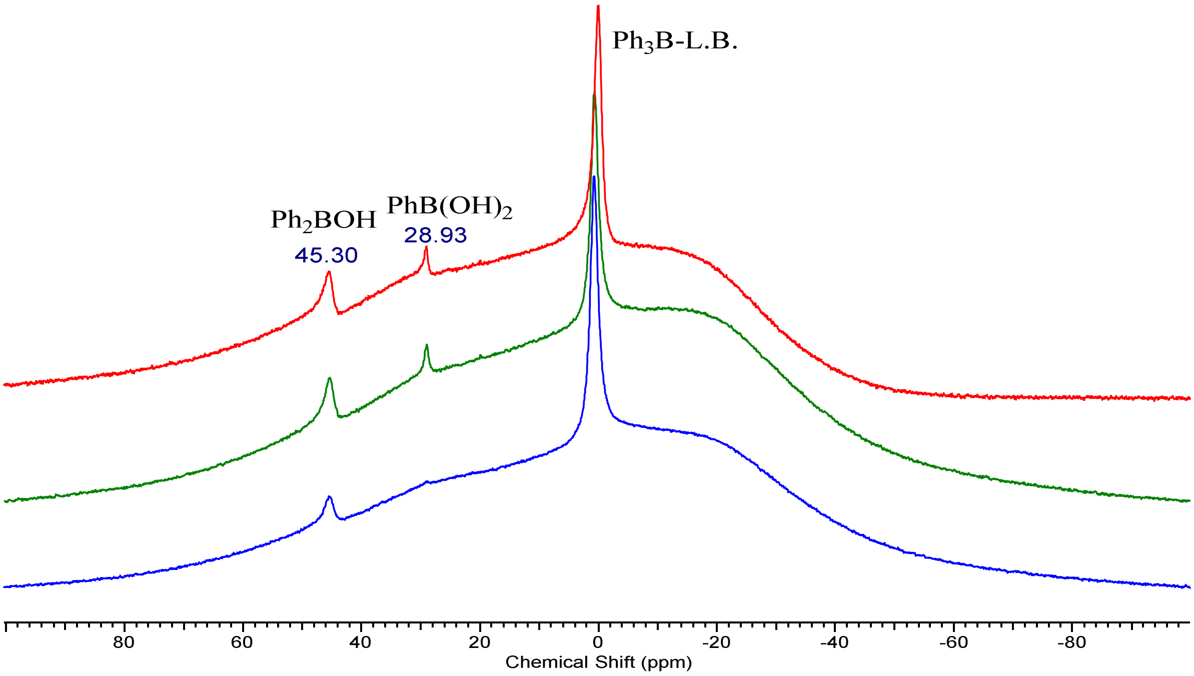

Figure S6. *In situ* ^{11}B-NMR spectra of $BPh_3/PhNH_2$ 1:1 (blue), $BPh_3/H_2O/PhNH_2$ 1:1:1 (green) and BPh_3/H_2O 1:1 (red) d_3-acetonitrile.

In an attempt to reach the slow exchange regime, a VT-NMR analysis was performed, but the equilibrium still persisted at -40°C in d_3-MeCN.

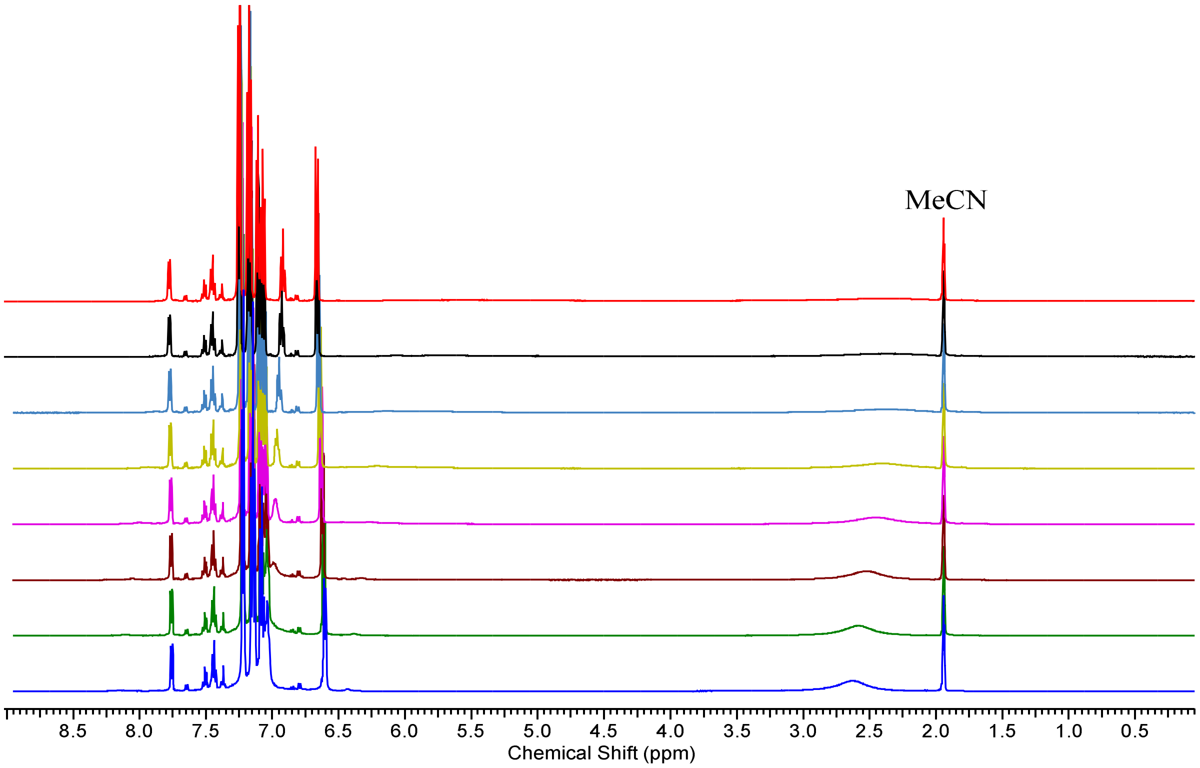

Figure S7. *In situ* ^{1}H-NMR spectra of $BPh_3/H_2O/PhNH_2$ 1:1:1 in d_3-acetonitrile. Dark blue (-40°C), green (-30°C), brown (-20°C), pink (-10°C), yellow (0°C), light blue (10°C), black 20°C), red (25°C).

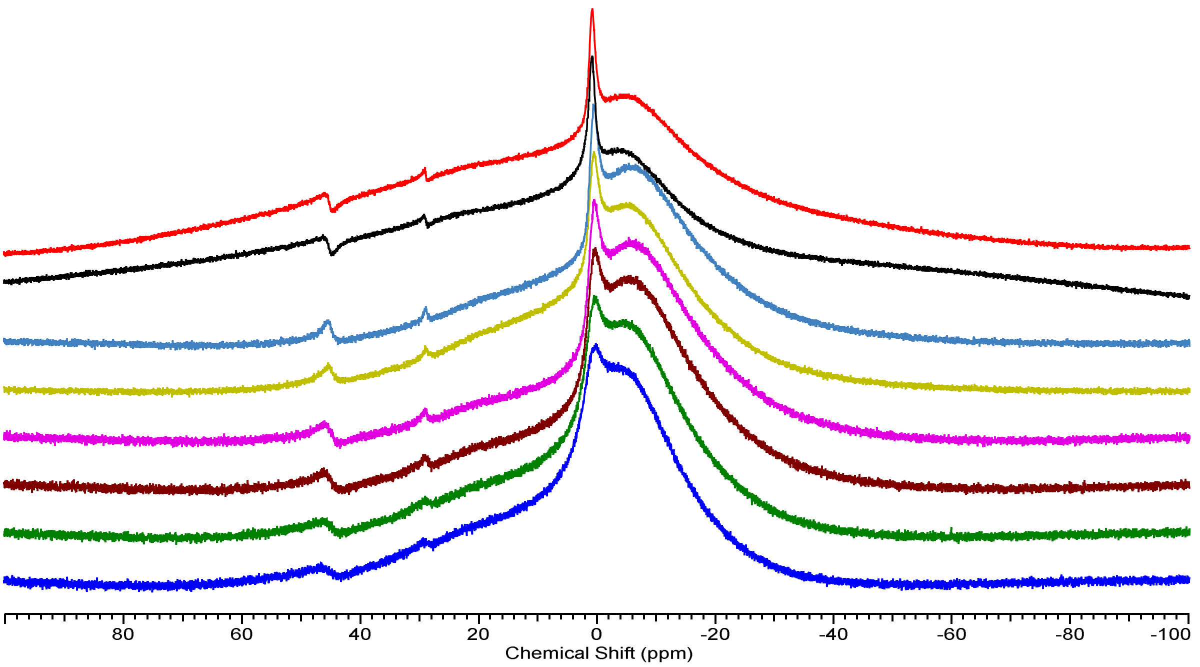

Figure S8. *In situ* ^{11}B-NMR spectra of $BPh_3/H_2O/PhNH_2$ 1:1:1 in d_3-acetonitrile. Dark blue (-40°C), green (-30°C), brown (-20°C), pink (-10°C), yellow (0°C), light blue (10°C), black 20°C), red (25°C).

$BPh_3/H_2O/BnNH_2$ (1:1:1): A J. Youngs NMR tube was charged with BPh_3 (14.0 mg, 0.055 mmol, 1.0 eq.), H_2O (1 µL, 0.055 mmol, 1.0 eq.) and $BnNH_2$ (6 µL, 0.055 mmol, 1.0 eq.) in *d_3*-acetonitrile (0.5 mL). Ph_3B-H_2NBn adduct was detected as the main species.

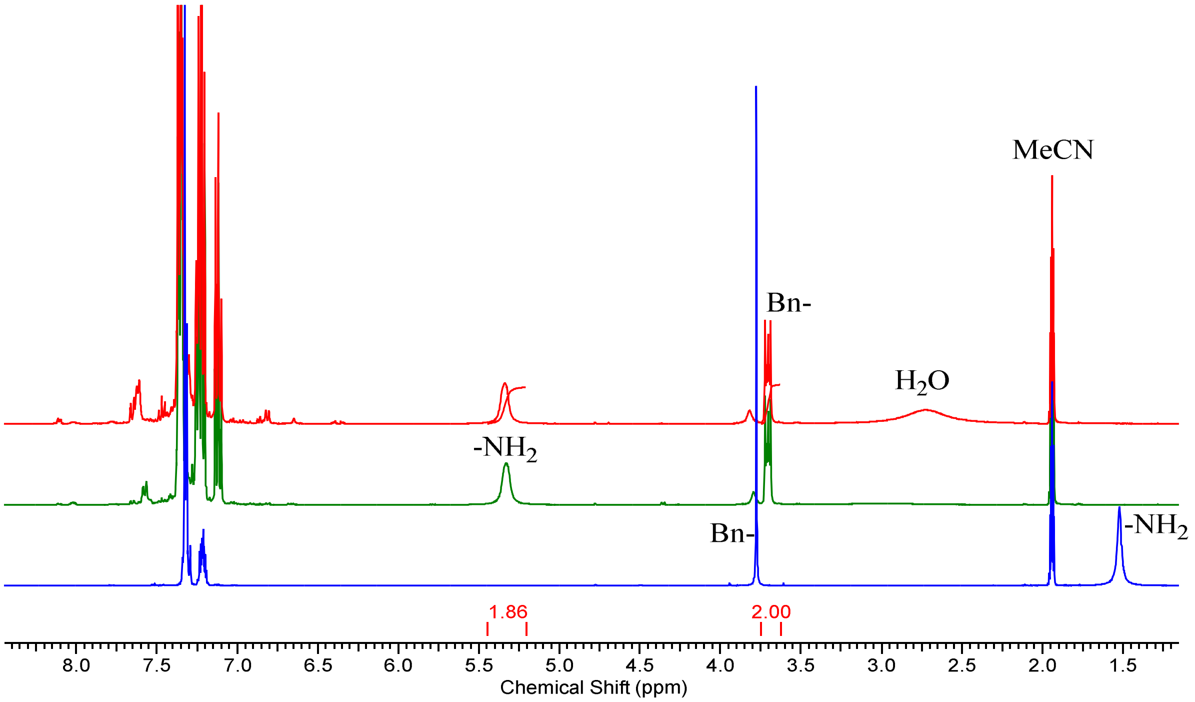

Figure S9. *In situ* ^{1}H-NMR spectra of $BnNH_2$ (blue), $BPh_3/BnNH_2$ 1:1 (green) and $BPh_3/H_2O/BnNH_2$ 1:1:1 (red) in d_3-acetonitrile.

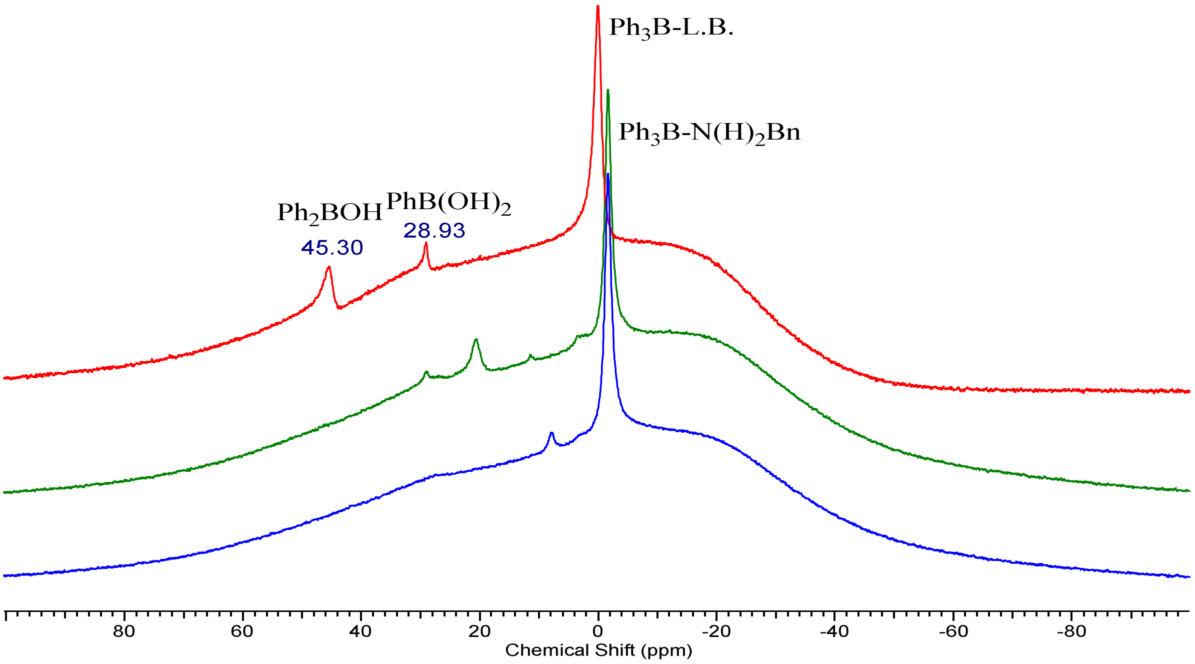

Figure S10. *In situ* ^{11}B-NMR spectra of $BPh_3/BnNH_2$ 1:1 (blue), $BPh_3/H_2O/BnNH_2$ 1:1:1 (green) and BPh_3/H_2O 1:1 (red) in d_3-acetonitrile.

$B(C_6F_5)_3/H_2O/BnNH_2$ (1:1:1): A J. Youngs NMR tube was charged with $B(C_6F_5)_3$ (30.0 mg, 0.055 mmol, 1.0 eq.), H_2O (1 µL, 0.055 mmol, 1.0 eq.) and $BnNH_2$ (6 µL, 0.055 mmol, 1.0 eq.) in *d_3*-acetonitrile (0.5 mL). $(F_5C_6)_3BOH^-$ was detected as the main species (at -4.4 ppm in the ^{11}B-NMR). The peaks observed for $B(C_6F_5)_3/BnNH_2$ 1:1 could not be assigned unambiguously (we tentatively assign them to the adducts of $B(C_6F_5)_3$ with the amine and MeCN).

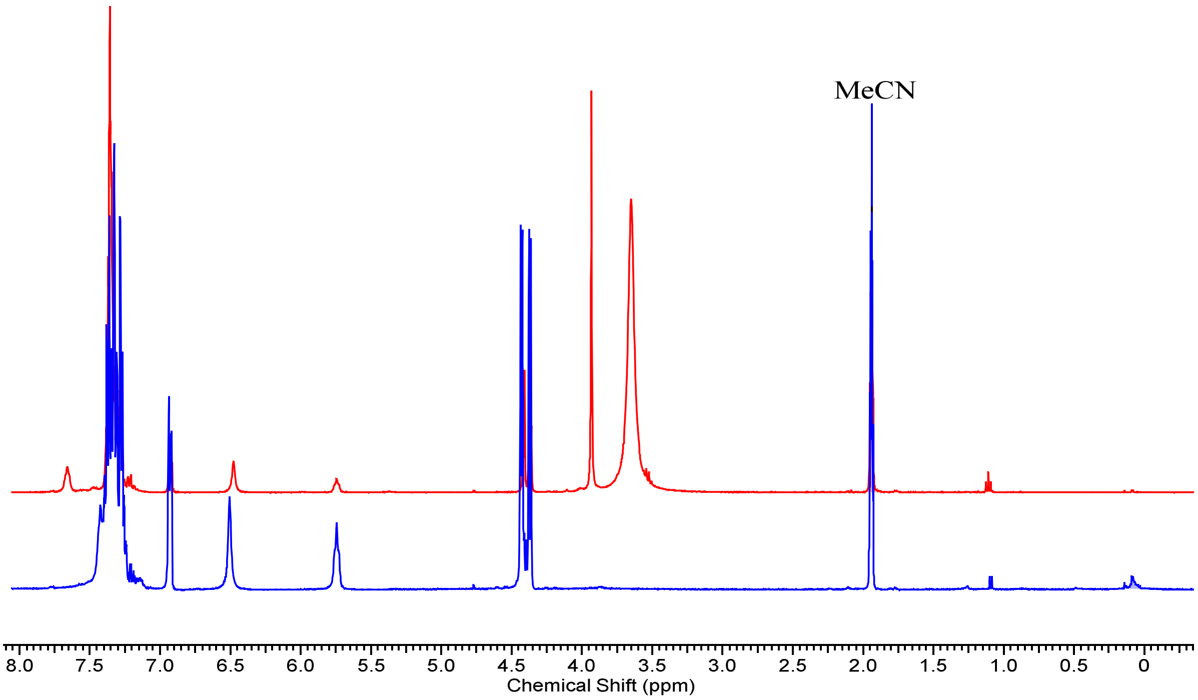

Figure S11. *In situ* ^{1}H-NMR spectra of $B(C_6F_5)_3/BnNH_2$ 1:1 (blue) and $B(C_6F_5)_3/H_2O/BnNH_2$ 1:1:1 (red) in d_3-acetonitrile.

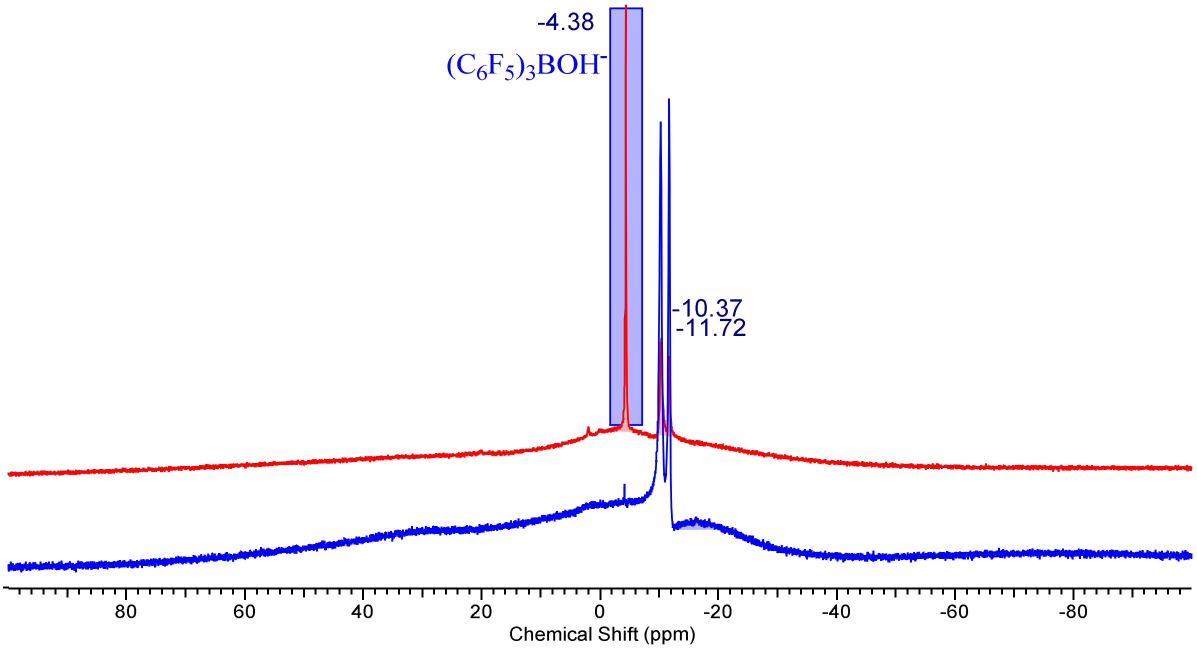

Figure S12. *In situ* ^{11}B-NMR spectra of $B(C_6F_5)_3/BnNH_2$ 1:1 (blue) and $B(C_6F_5)_3/H_2O/BnNH_2$ 1:1:1 (red) in d_3-acetonitrile.

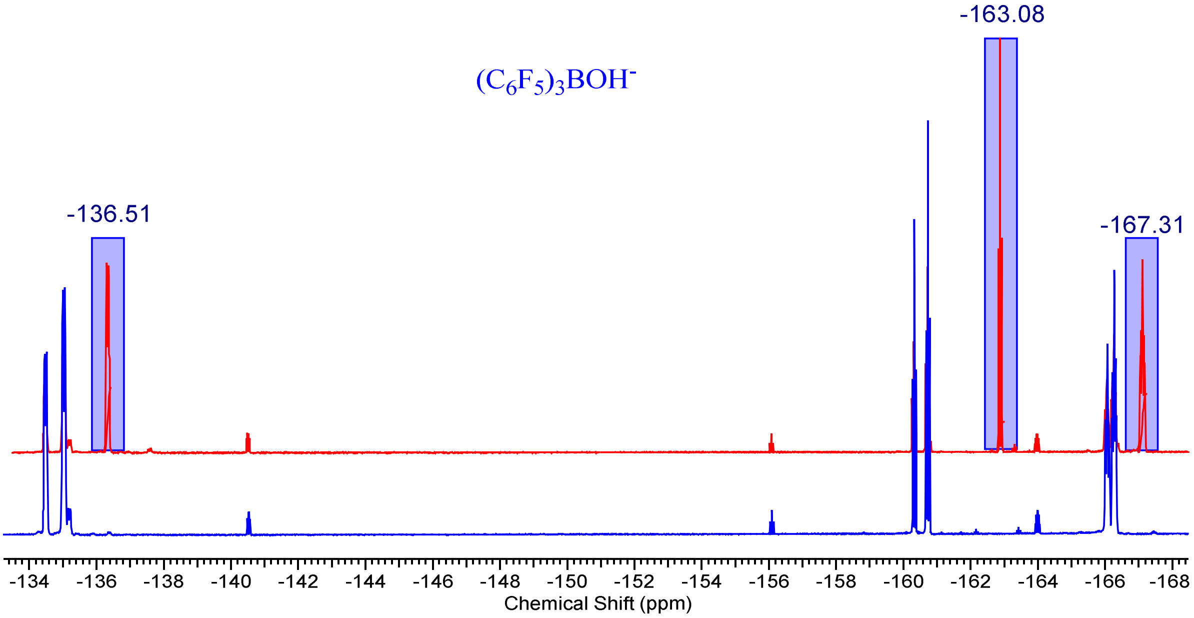

Figure S13. *In situ* ^{19}F-NMR spectra of $B(C_6F_5)_3/BnNH_2$ 1:1 (blue) and $B(C_6F_5)_3/H_2O/BnNH_2$ 1:1:1 (red) in d_3-acetonitrile.

To confirm the $(F_5C_6)_3BOH^-$ formation, an equimolar mixture of $B(C_6F_5)_3/H_2O/^tBuNH_2$ was dissolved in DCM, observing quantitative formation of $(F_5C_6)_3BOH^-$. Then, the system was dried in vacuo and the solid obtained was dissolved in d_3-acetonitrile, obtaining ^{11}B and ^{19}F-NMR resonances for $(F_5C_6)_3BOH^-$ comparable to those observed for $B(C_6F_5)_3/H_2O/BnNH_2$ mixture.

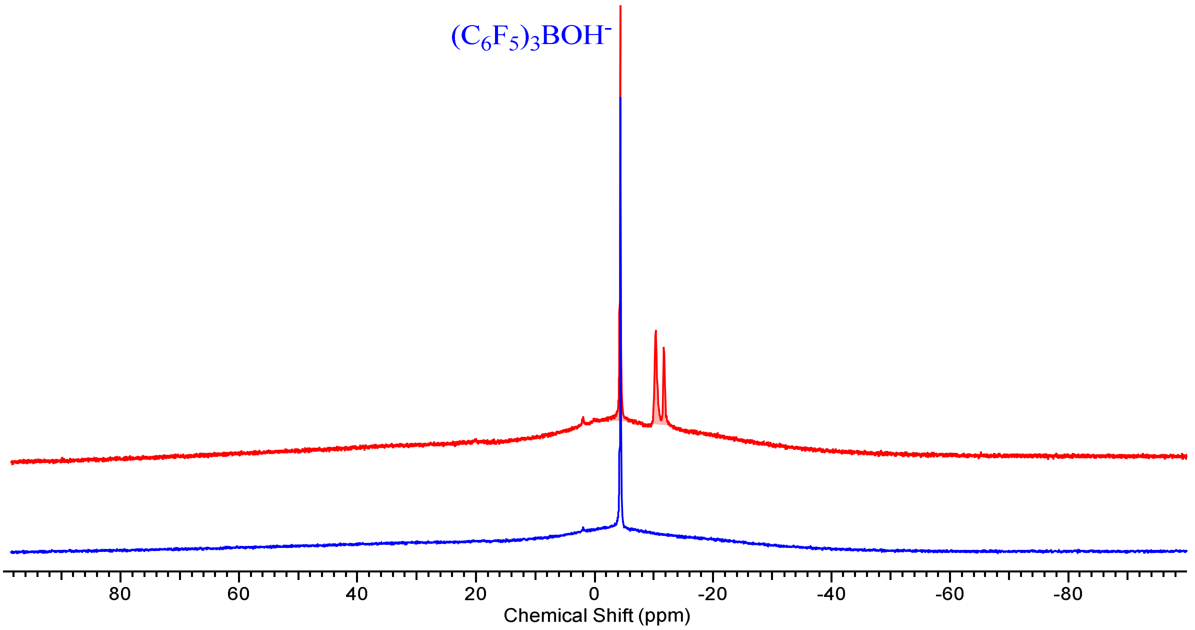

Figure S14. ^{11}B-NMR spectra of $(F_5C_6)_3BOH^-$ (blue) and $B(C_6F_5)_3/H_2O/BnNH_2$ 1:1:1 (red) in d_3-acetonitrile.

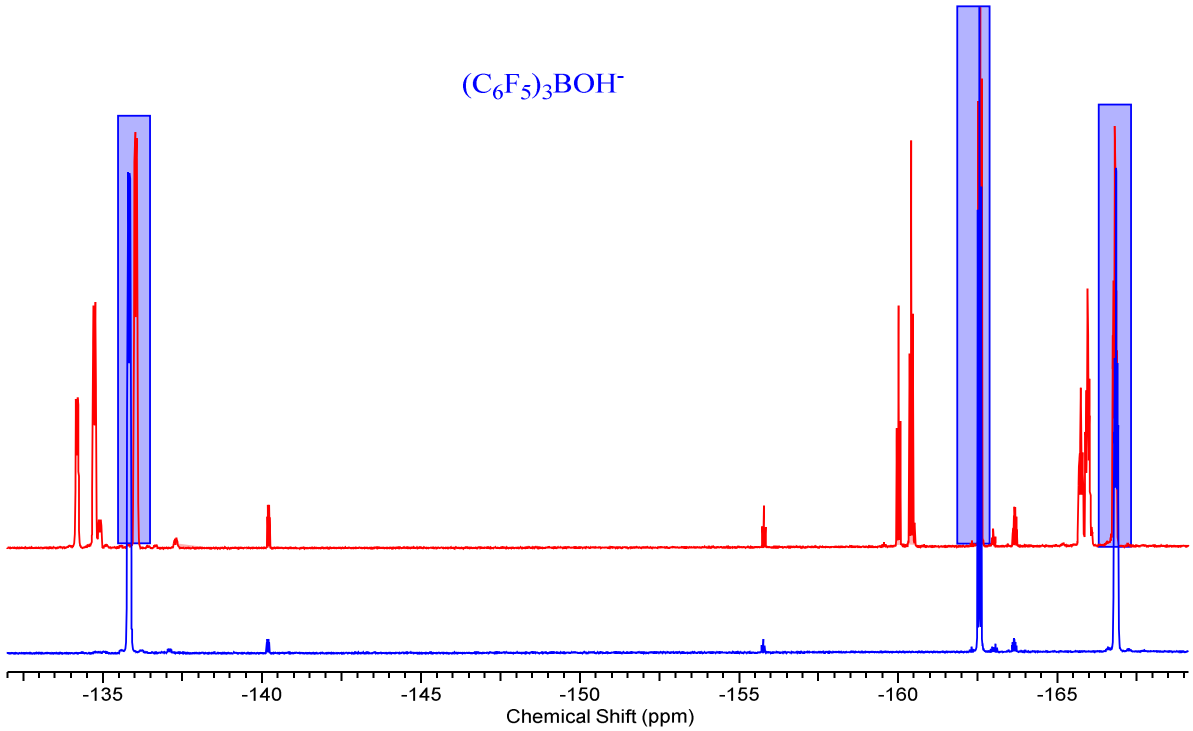

Figure S15. *In situ* ^{19}F-NMR spectra of $(F_5C_6)_3BOH^-$ (blue) and $B(C_6F_5)_3/H_2O/BnNH_2$ 1:1:1 (red) in d_3-acetonitrile.

$BPh_3/H_2O/^tBuNH_2$ (1:1:1): A J. Youngs NMR tube was charged with BPh_3 (14.0 mg, 0.055 mmol, 1.0 eq.), H_2O (1 µL, 0.055 mmol, 1.0 eq.) and $^tBuNH_2$ (6 µL, 0.055 mmol, 1.0 eq.) in *d*$_3$-acetonitrile (0.5 mL).

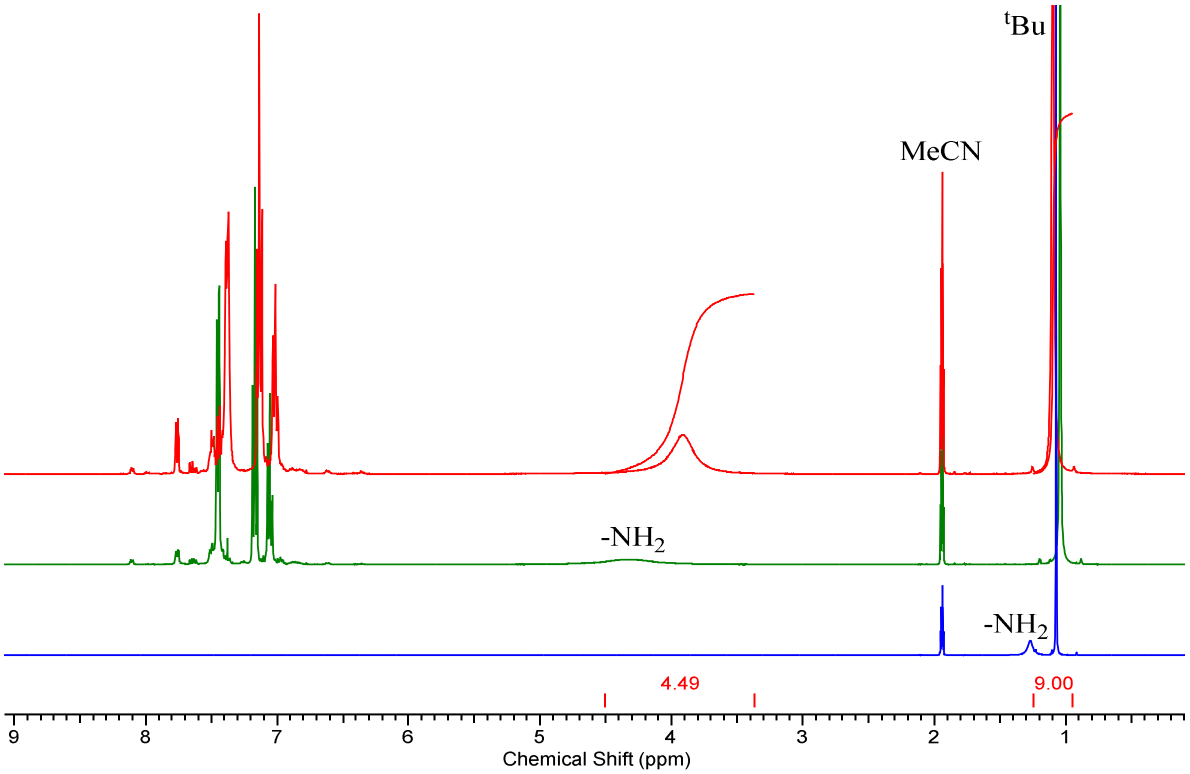

Figure S16. *In situ* ^{1}H-NMR spectra of $^tBuNH_2$ (blue), $BPh_3/^tBuNH_2$ 1:1 (green) and $BPh_3/H_2O/^tBuNH_2$ 1:1:1 (red) in d_3-acetonitrile.

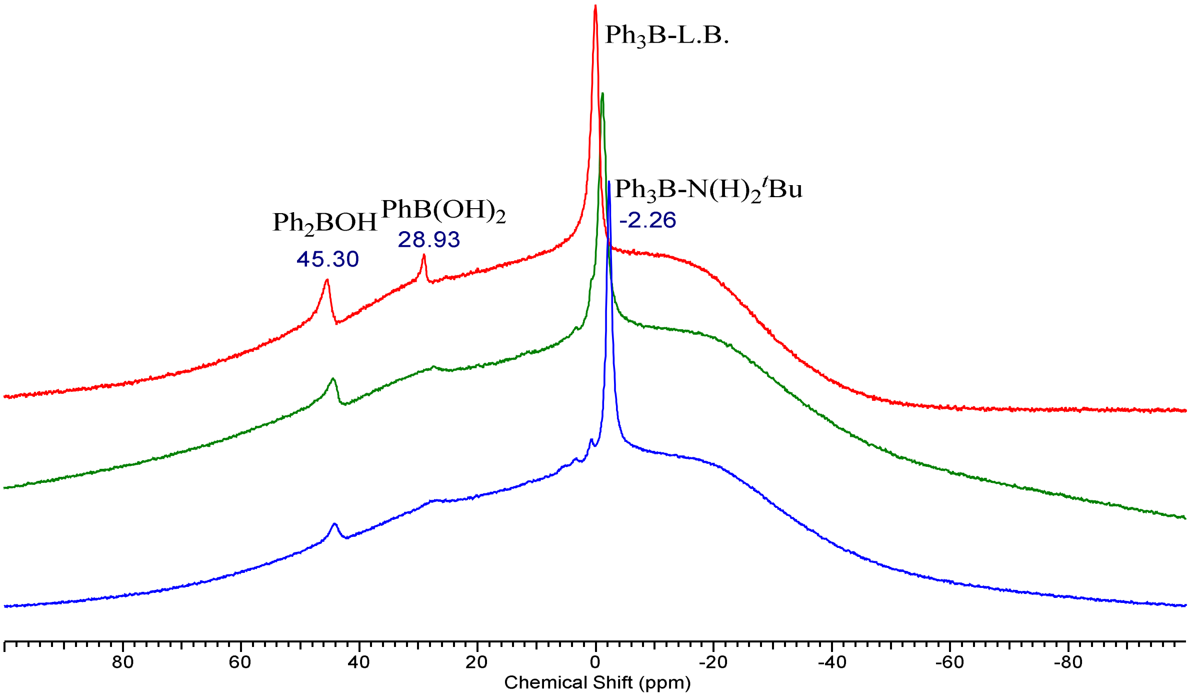

Figure S17. *In situ* ^{11}B-NMR spectra of $BPh_3/^tBuNH_2$ 1:1 (blue), $BPh_3/H_2O/^tBuNH_2$ 1:1:1 (green) and BPh_3/H_2O 1:1 (red) d_3-acetonitrile.

The VT-NMR analysis revealed that $^{t}BuN(H_2)N\text{-}BPh_3$ is a minor component and not observable at temperature higher than 10°C, however an additional dynamic process is not frozen out at -40°C in d_3-MeCN.

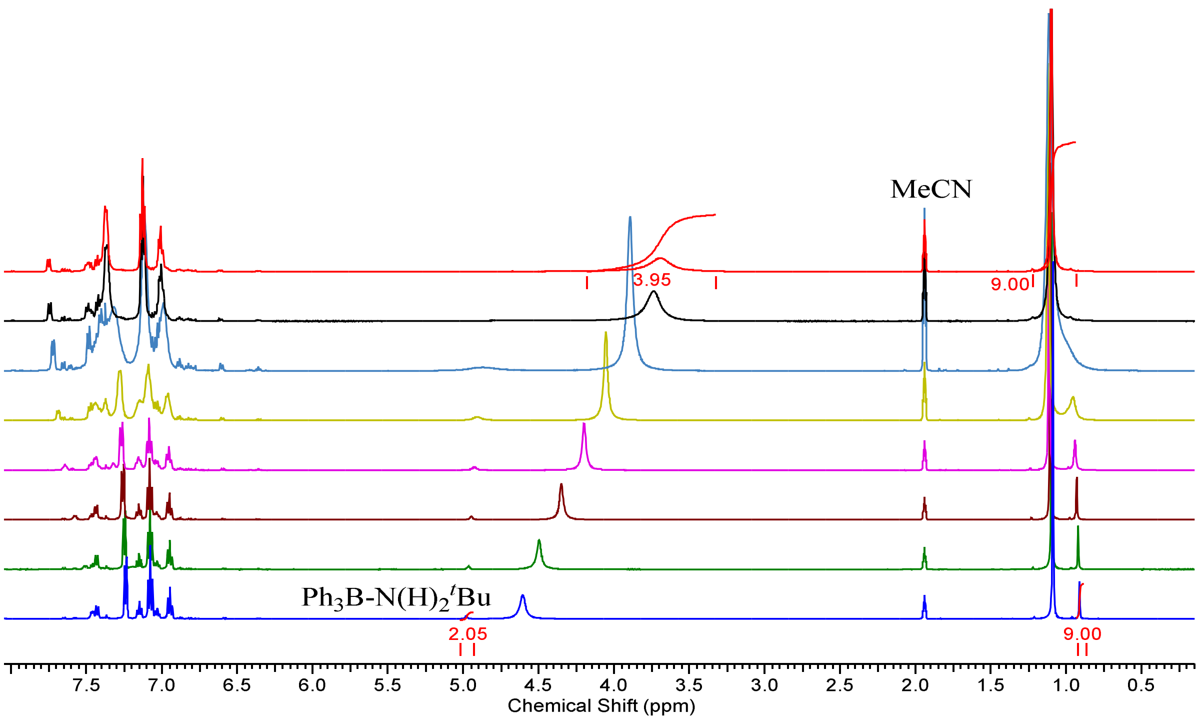

Figure S18. *In situ* ^{1}H-NMR spectra of $BPh_3/H_2O/^{t}BuNH_2$ 1:1:1 in d_3-acetonitrile. Dark blue (-40°C), green (-30°C), brown (-20°C), pink (-10°C), yellow (0°C), light blue (10°C), black 20°C), red (25°C).

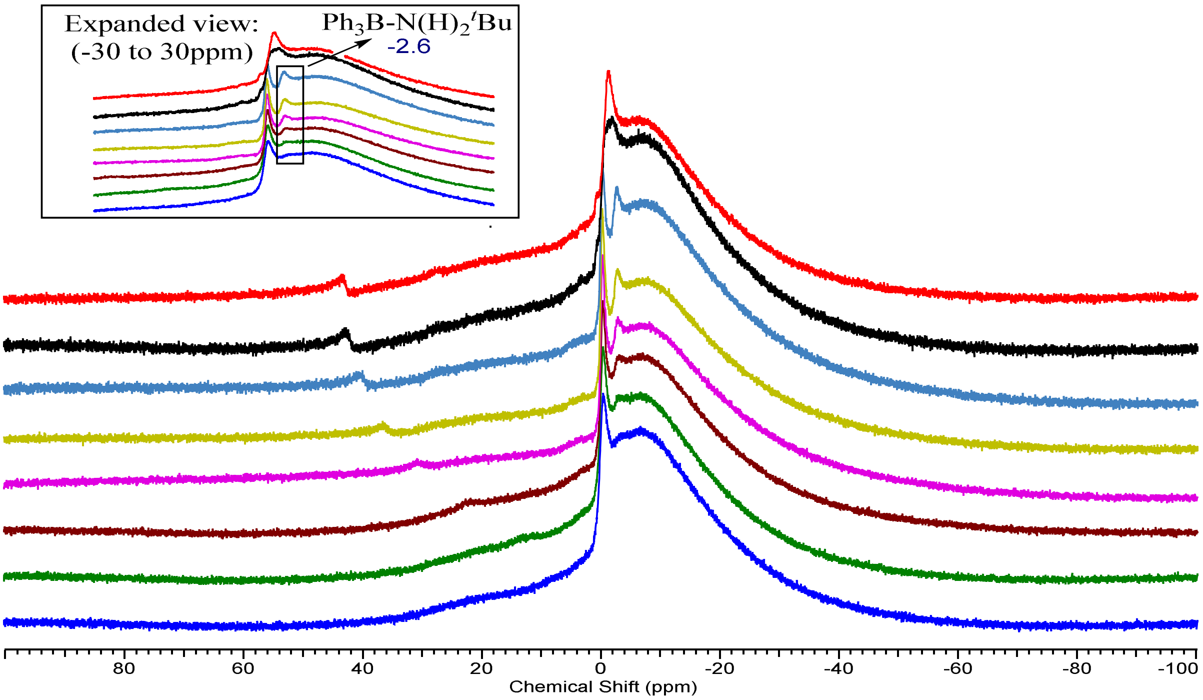

Figure S19. *In situ* ^{11}B-NMR spectra of $BPh_3/H_2O/^{t}BuNH_2$ 1:1:1 in d_3-acetonitrile. Dark blue (-40°C), green (-30°C), brown (-20°C), pink (-10°C), yellow (0°C), light blue (10°C), black 20°C), red (25°C).

BPh_3/Ph(H)C=NBn (1:1): A J. Youngs NMR tube was charged with BPh_3 (14.0 mg, 0.055 mmol, 1.0 eq.) and *N*-benzylidene benzylamine (11 µL, 0.055 mmol, 1.0 eq.) in *d_3*-acetonitrile (0.5 mL). Water traces induced a small degree of imine hydrolysis. Adding $BnNH_2$ (6 µL, 0.055 mmol, 1.0 eq.), $BnNH_2$-BPh_3 was observed as the main species. The unchanged chemical shift of the aldimine protons upon $BnNH_2$ addition may indicate weak imine coordination to BPh_3.

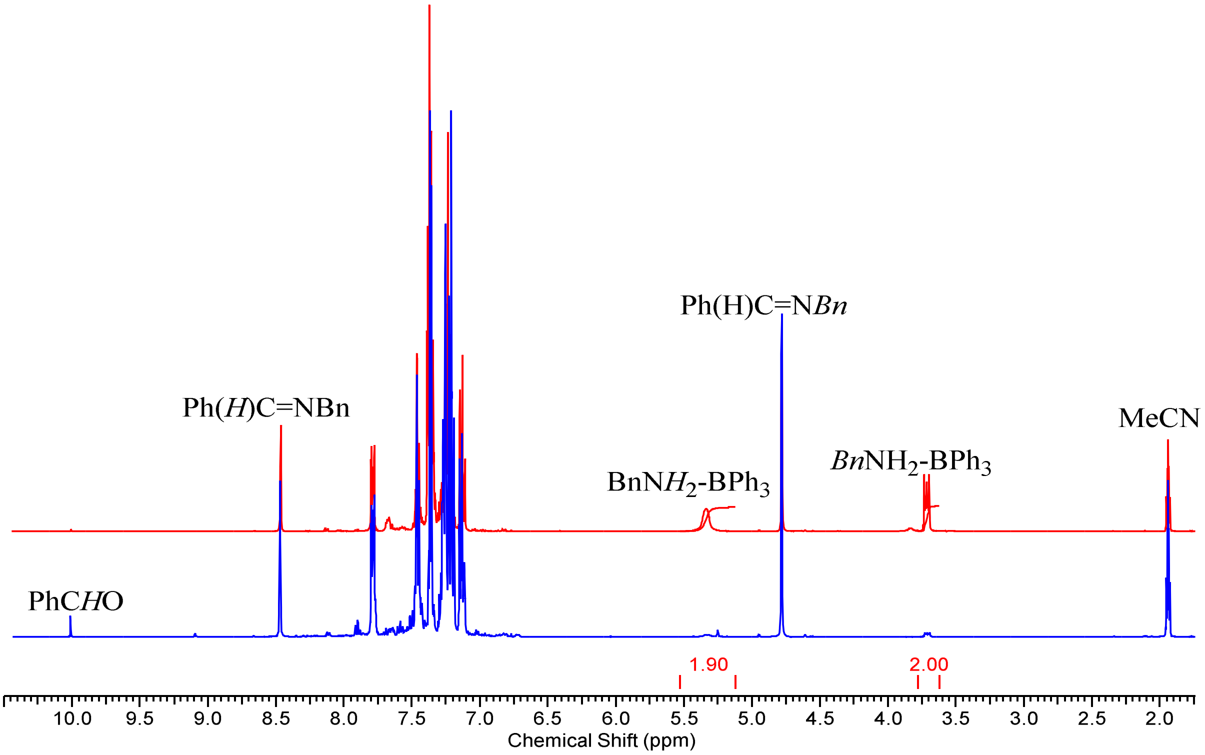

Figure S20. *In situ* ^{1}H-NMR spectra of BPh_3/Ph(H)C=NBn 1:1 in d_3-acetonitrile. Blue (after 5 min at r.t.), red (after $BnNH_2$ addition).

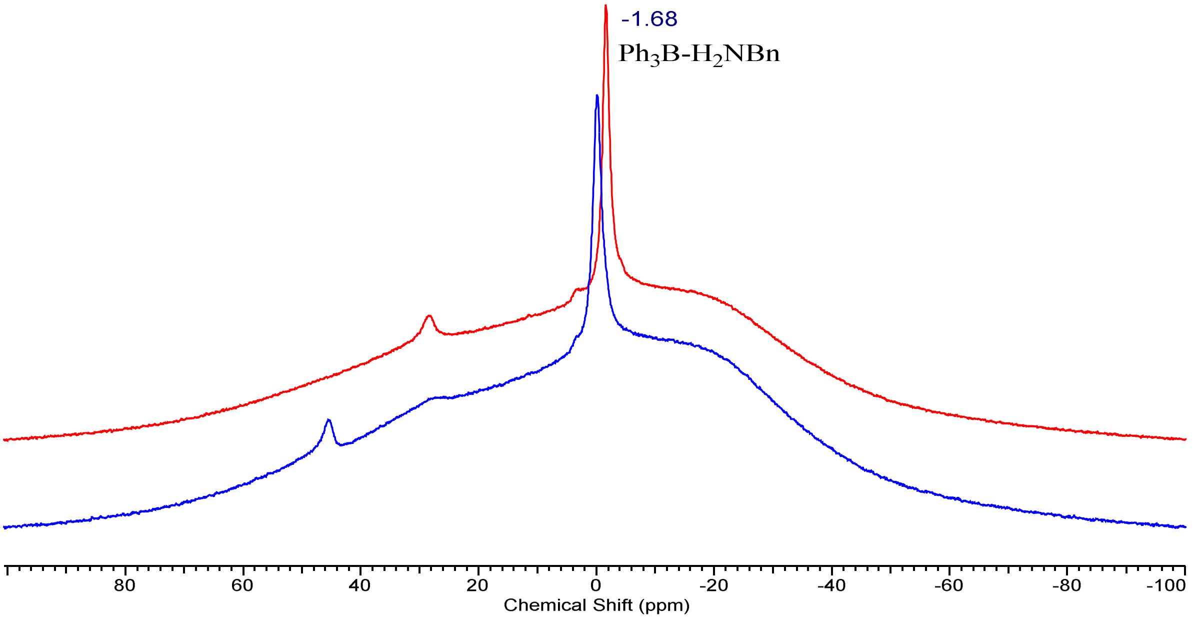

Figure S21. *In situ* ^{11}B-NMR spectra of BPh_3/Ph(H)C=NBn 1:1 in d_3-acetonitrile. Blue (after 5 min at r.t.), red (after $BnNH_2$ addition).

3. Decomposition of Ph_3B-OH_2 in the presence of amines

BPh_3/H_2O/$PhNH_2$ (1:10:10): A J. Youngs NMR tube equipped with d_6-DMSO capillary was charged with BPh_3 (7.0 mg, 0.028 mmol, 1.0 eq.), H_2O (5 μL, 0.275 mmol, 10.0 eq.) and $PhNH_2$ (25 μL, 0.275 mmol, 10.0 eq.) in acetonitrile (0.5 mL). Upon heating at 100°C for 20 hours, full BPh_3 decomposition was observed.

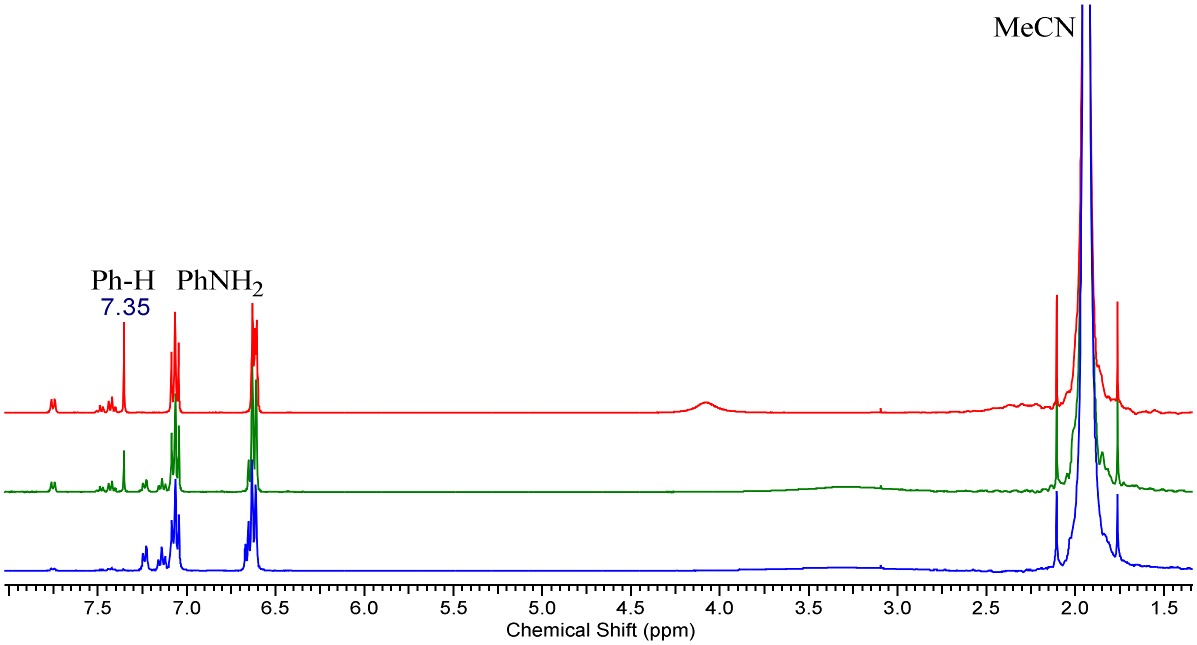

Figure S22. *In situ* ^{1}H-NMR spectra of BPh_3/H_2O/$PhNH_2$ (1:10:10) in acetonitrile. Blue (t = 5 min, r.t.), green (after t = 3 hours at 100°C), red (after 20 hours at 100°C).

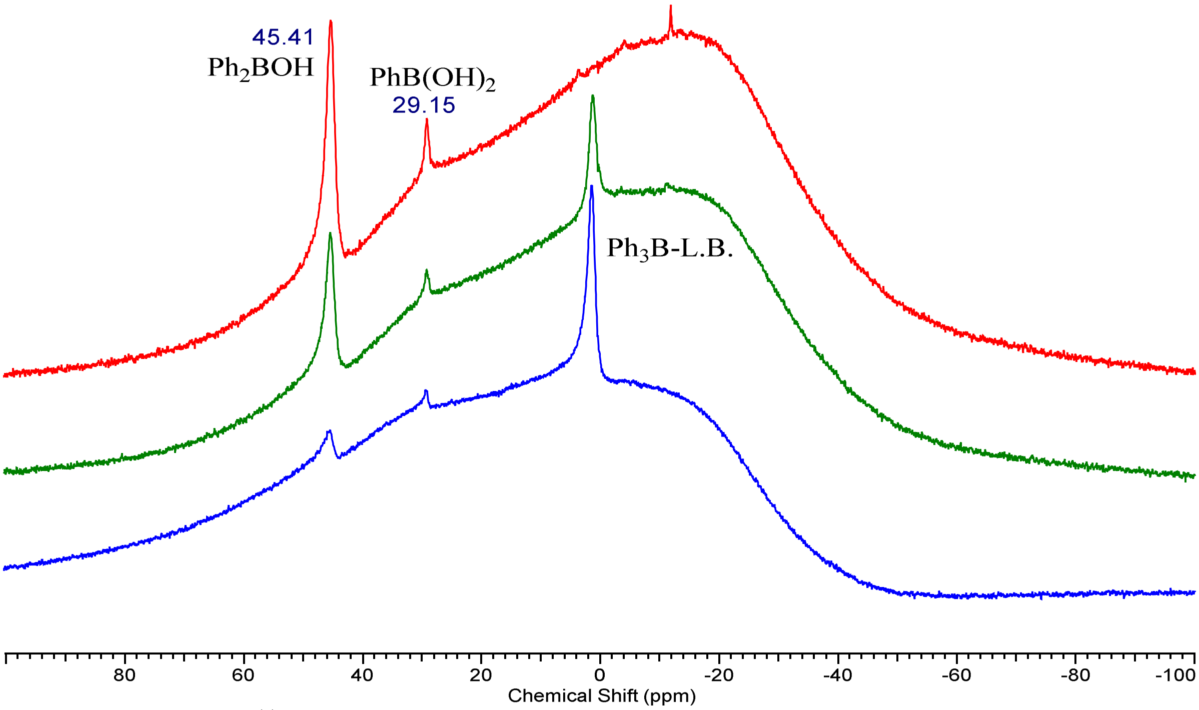

Figure S23. *In situ* ^{11}B-NMR spectra of BPh_3/H_2O/$PhNH_2$ (1:10:10) in acetonitrile. Blue (t = 5 min, r.t.), green (after t = 3 hours at 100°C), red (after 20 hours at 100°C).

$BPh_3/H_2O/BnNH_2$ (1:10:10): A J. Youngs NMR tube equipped with d_6-DMSO capillary was charged with BPh_3 (7.0 mg, 0.028 mmol, 1.0 eq.), H_2O (5 µL, 0.275 mmol, 10.0 eq.) and $BnNH_2$ (30 µL, 0.275 mmol, 10.0 eq.) in acetonitrile (0.5 mL). Upon heating at 100°C for 20 hours, minimal decomposition was observed as based on the absence of significant PhH.

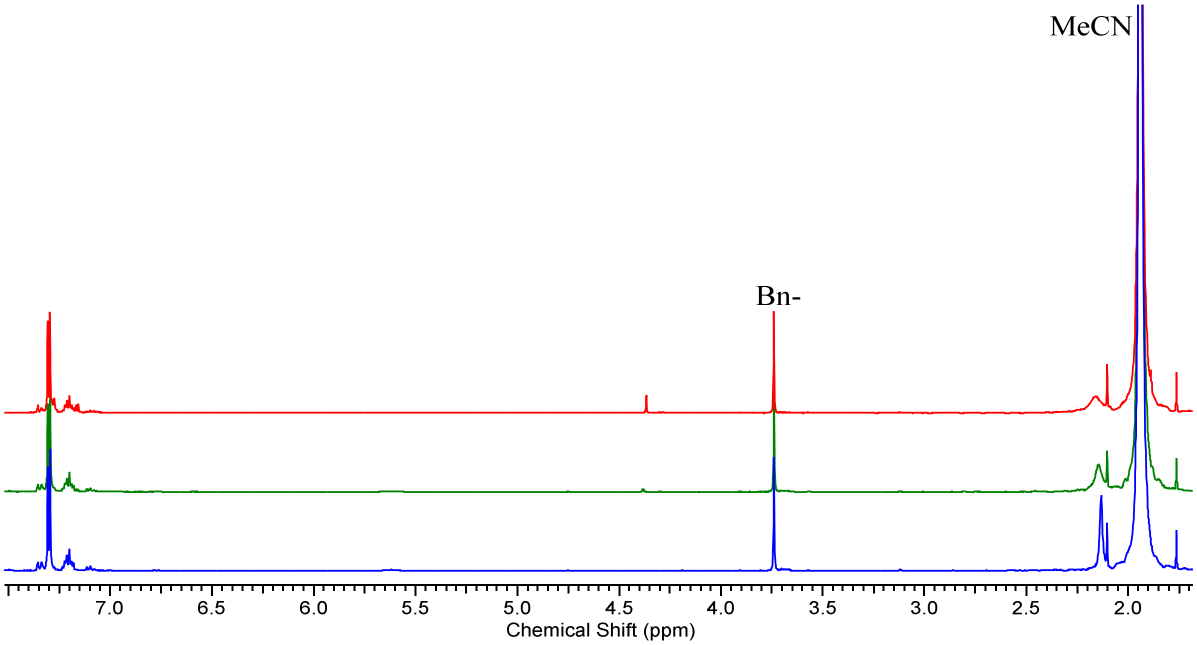

Figure S24. *In situ* ^{1}H-NMR spectra of $BPh_3/H_2O/BnNH_2$ (1:10:10) in acetonitrile. Blue (t = 5 min, r.t.), green (after t = 3 hours at 100°C), red (after 20 hours at 100°C).

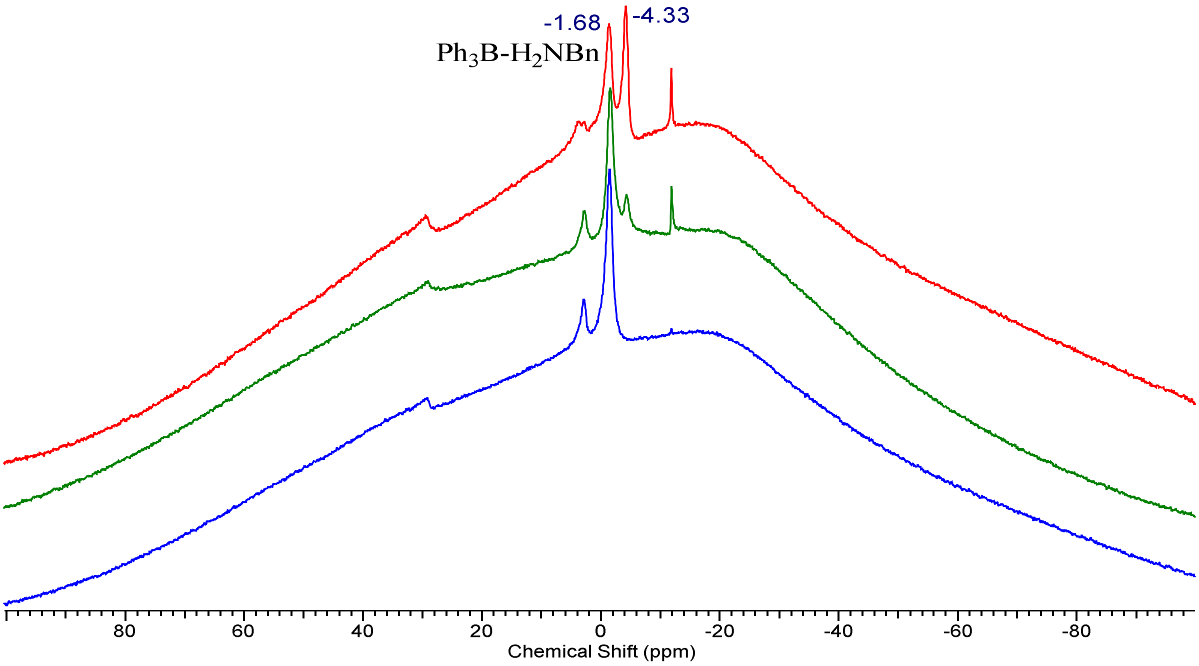

Figure S25. *In situ* ^{11}B-NMR spectra of $BPh_3/H_2O/BnNH_2$ (1:10:10) in acetonitrile. Blue (t = 5 min, r.t.), green (after t = 3 hours at 100°C), red (after 20 hours at 100°C).

$BPh_3/H_2O/^tBuNH_2$ (1:10:10): A J. Youngs NMR tube equipped with *d_6*-DMSO capillary was charged with BPh_3 (7.0 mg, 0.028 mmol, 1.0 eq.), H_2O (5 µL, 0.275 mmol, 10.0 eq.) and $^tBuNH_2$ (30 µL, 0.275 mmol, 10.0 eq.) in acetonitrile (0.5 mL). Upon heating at 100°C for 20 hours, partial BPh_3 decomposition was observed (PhH present in the ^1H NMR spectrum), but tetracoordinated L.B.-BPh_3 species were still the main species.

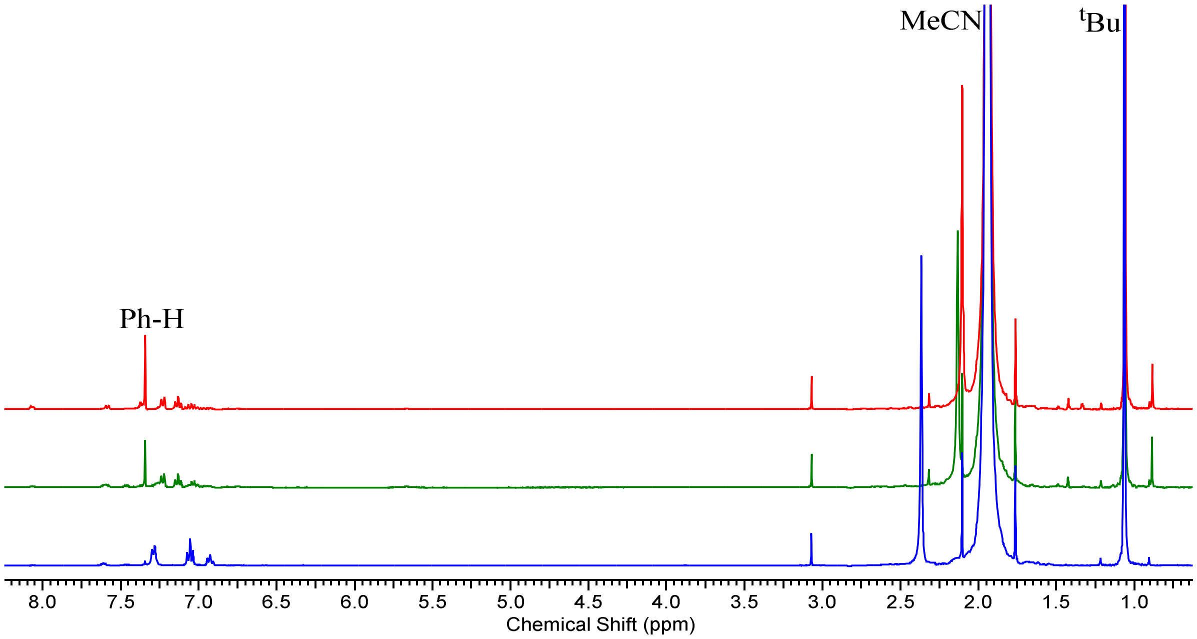

Figure S26. *In situ* ^1H-NMR spectra of $BPh_3/H_2O/^tBuNH_2$ (1:10:10) in acetonitrile. Blue (t = 5 min, r.t.), green (after t = 3 hours at 100°C), red (after 20 hours at 100°C).

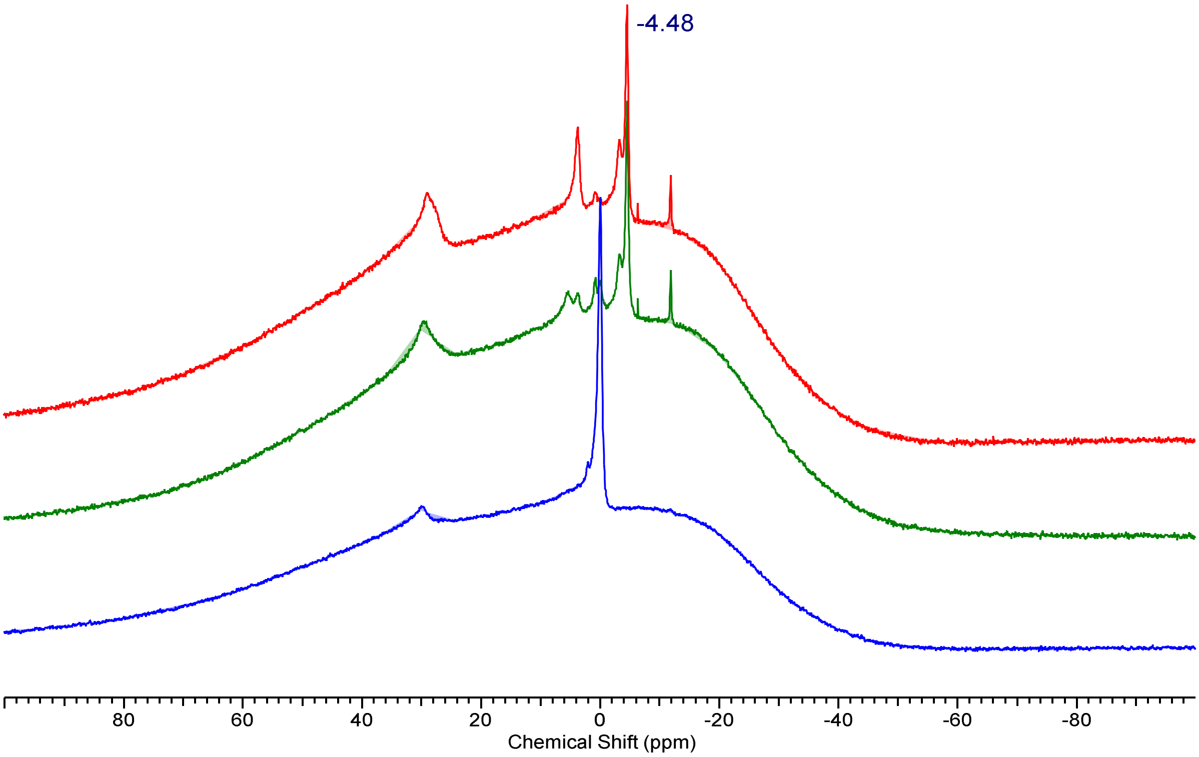

Figure S27. *In situ* ^{11}B-NMR spectra of $BPh_3/H_2O/^tBuNH_2$ (1:10:10) in acetonitrile. Blue (t = 5 min, r.t.), green (after t = 3 hours at 100°C), red (after 20 hours at 100°C).

4. Initial screening of reaction conditions

General Procedure: A J. Youngs NMR tube equipped with a DMSO-d_6 capillary was loaded with the catalyst in the appropriate solvent. Then, benzaldehyde (1.00 eq.), benzylamine (1.20 eq.) and dimethylphenylsilane (3.50 eq.) were added. After the monitoring of the initial reaction mixture by multinuclear NMR spectroscopy, the J. Youngs NMR tube was heated up at 60 or 100°C for 25 hours and then monitored by ^{1}H, ^{11}B and ^{29}Si-NMR spectroscopy. Subsequent addition of mesitylene (25 μL) to the reaction mixture allowed the determination of the NMR yields based on the protons attached to the α-carbons. The reaction mixture was then concentrated under reduced pressure and the crude analysed by multinuclear NMR spectroscopy and by GC-MS analysis. Both analysis revealed the main species present were $PhMe_2SiOH$, $(PhMe_2Si)_2O$ and the desired product. Purification by flash column chromatography on silica gel gave isolated yields which were in good agreement with the NMR yields.

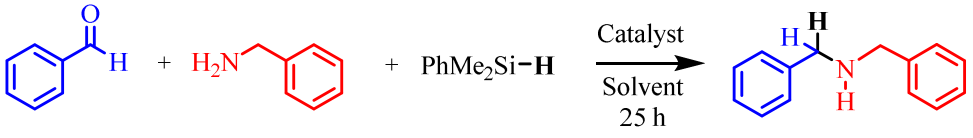

Entry	Solvent	Eq. silane	Eq. Cat.	Temp.	Yield (%)[a]	
					$B(C_6F_5)_3$	BPh_3
**1**	*o*-DCB	1.2	10 % mol	100 °C	< 5	< 5
**2**	MeCN	1.2	10 % mol	100 °C	< 5	33
**3**	*o*-DCB	3.5	10 % mol	100 °C	< 5	< 5
**4**	MeCN	3.5	10 % mol	100 °C	< 5	87 (80)[b]
**5**	MeCN	3.5	5 % mol	100 °C	< 5	35
**6**	MeCN	3.5	10 % mol	60 °C	< 5	6

[a] NMR yield using mesitylene as standard. [b] Isolated yield.

The reaction in entry 4 was repeated with the same batch of BPh_3 obtaining similar NMR conversion (82% *vs* 87%). A new batch of BPh_3 was opened and left under ambient conditions for two weeks: repeating the standard reaction with this batch of BPh_3 revealed slower activity (52% NMR yield).

Reductive amination with $B(C_6F_5)_3$ in o-DCB (Entry 3): Following the general procedure, a J. Youngs NMR tube was loaded with $B(C_6F_5)_3$ (12.3 mg, 0.024 mmol, 0.10 eq.) in o-dichlorobenzene (0.5 mL), followed by the addition of benzaldehyde (25 µL, 0.241 mmol, 1.00 eq.), benzylamine (32 µL, 0.289 mmol, 1.20 eq.) and dimethylphenylsilane (132 µL, 0.843 mmol, 3.50 eq.). After heating at 100°C for 25 hours, multinuclear NMR spectroscopy revealed minimal reduction of the imine.

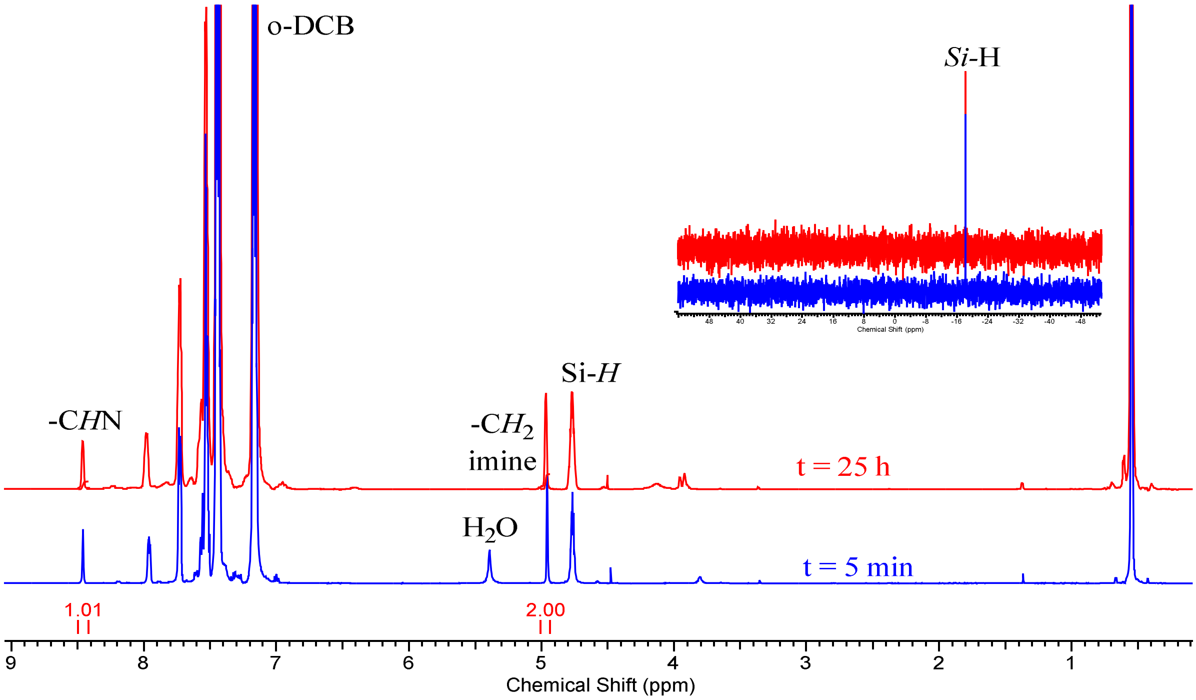

Figure S28. *In situ* ^{1}H and ^{29}Si-NMR spectra of the reductive amination of benzaldehyde/ benzylamine with $B(C_6F_5)_3$ in o-dichlorobenzene, using $PhMe_2SiH$ (3.50 eq.) as reductant. Blue (t = 5 min, r.t.), red (after 25 hours at 100°C).

Reductive amination with BPh_3 in MeCN (Entry 4): Following the general procedure, a J. Youngs NMR tube was loaded with BPh_3 (5.8 mg, 0.024 mmol, 0.10 eq.) in acetonitrile (0.5 mL), followed by the addition of benzaldehyde (25 µL, 0.241 mmol, 1.00 eq.), benzylamine (32 µL, 0.289 mmol, 1.20 eq.) and dimethylphenylsilane (132 µL, 0.843 mmol, 3.50 eq.). After heating at 100°C for 25 hours, in the ^{1}H NMR spectrum the signal of the protons attached to α-carbons appeared at δ = 3.77 ppm (NMR yield = 87%).

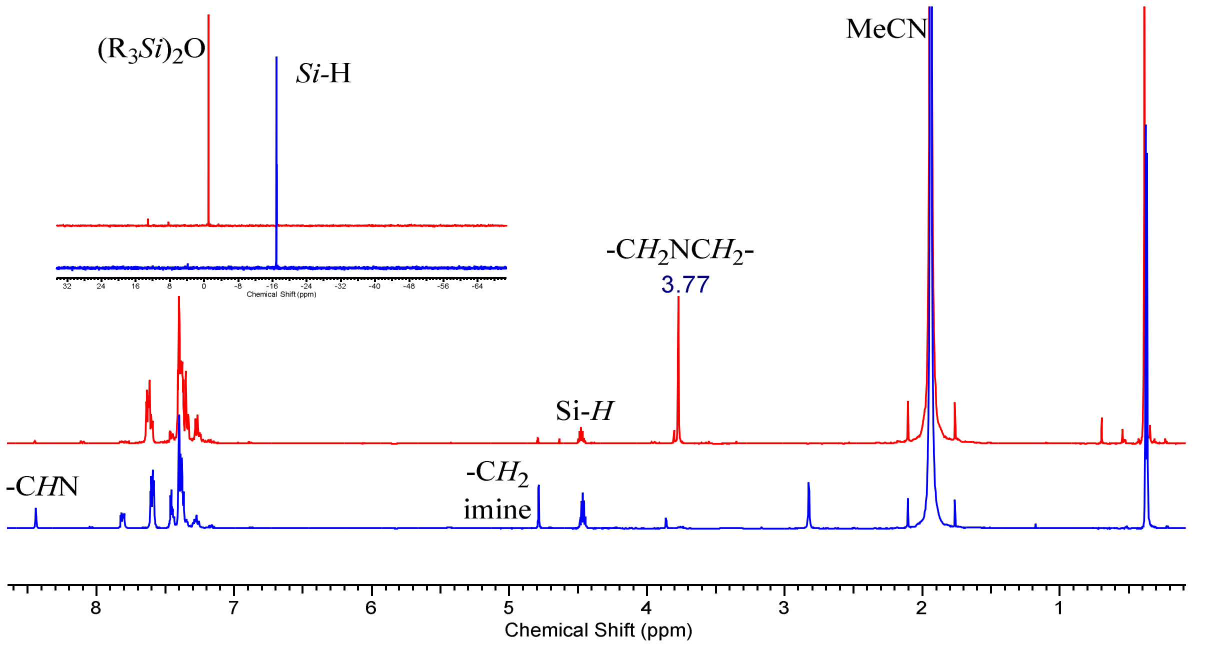

Figure S29. *In situ* ^{1}H and ^{29}Si-NMR spectra of the reductive amination of benzaldehyde/ benzylamine with BPh_3 in acetonitrile, using $PhMe_2SiH$ (3.50 eq.) as reductant. Blue (t = 5 min, r.t.), red (after 25 hours at 100°C).

After concentration under reduced pressure, in the crude the main species detected were $(PhMe_2Si)_2O$ and dibenzylamine. The crude was purified by flash column chromatography (Pet. Et. : EtOAc 9:1) obtaining dibenzylamine as yellow oil (38 mg, 0.193 mmol, 80%). R_f = 0.30. ^{1}H NMR ($CDCl_3$, 400 MHz, 298 K): δ 7.28-7.21 (m, 8H), 7.20-7.13 (m, 2H), 3.72 (s, 4H), 1.79 (br, 1H) ppm. ^{13}C{^{1}H} NMR ($CDCl_3$, 100 MHz, 298 K): 140.2, 128.4, 128.1, 126.9, 53.1 ppm. GC-MS: *m/z* calculated for $C_{14}H_{15}N$, 197.1; found 197.1. GC-MS retention times of analyte: 10.43 minutes (Dibenzylamine).The data were in agreement with those reported in the literature.[3]

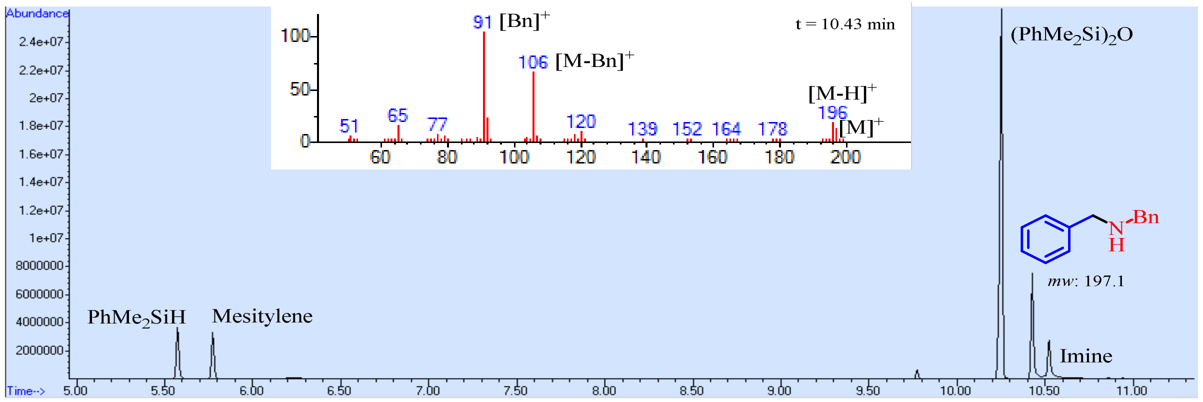

Figure S30. GC-MS of reaction crude.

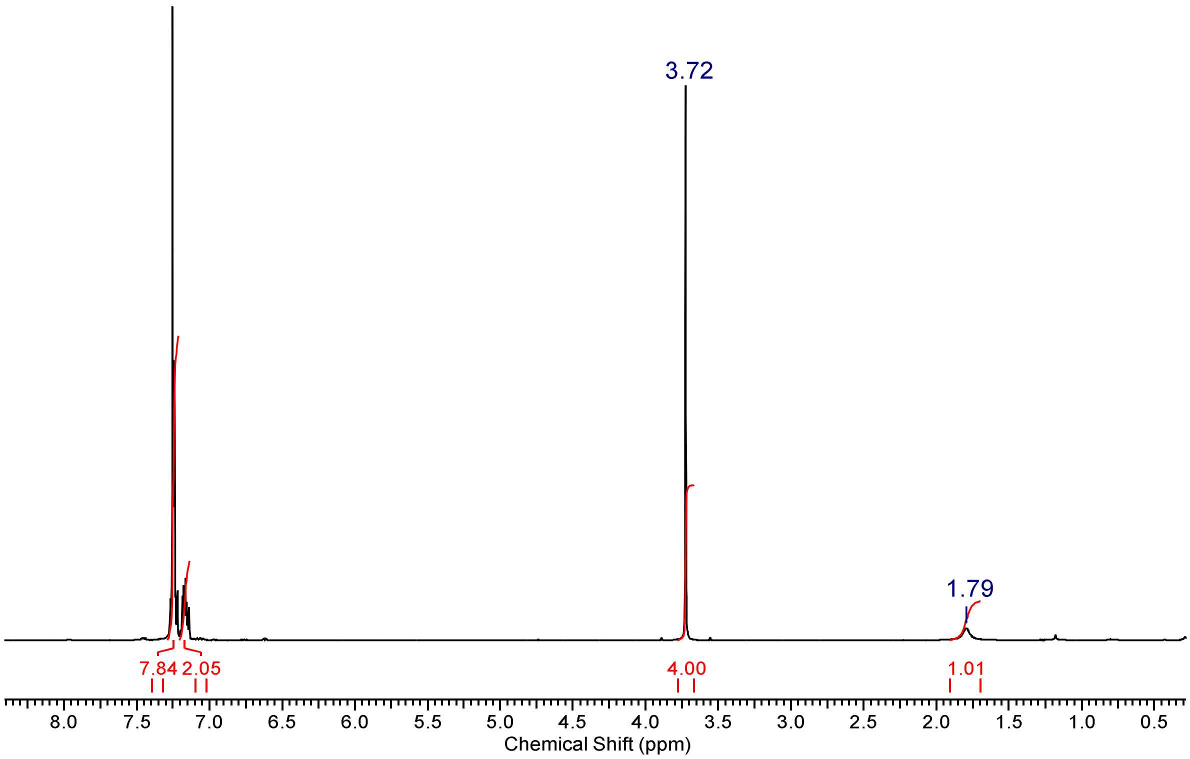

Figure S31. ^{1}H-NMR spectrum of the isolated dibenzylamine ($CDCl_3$).

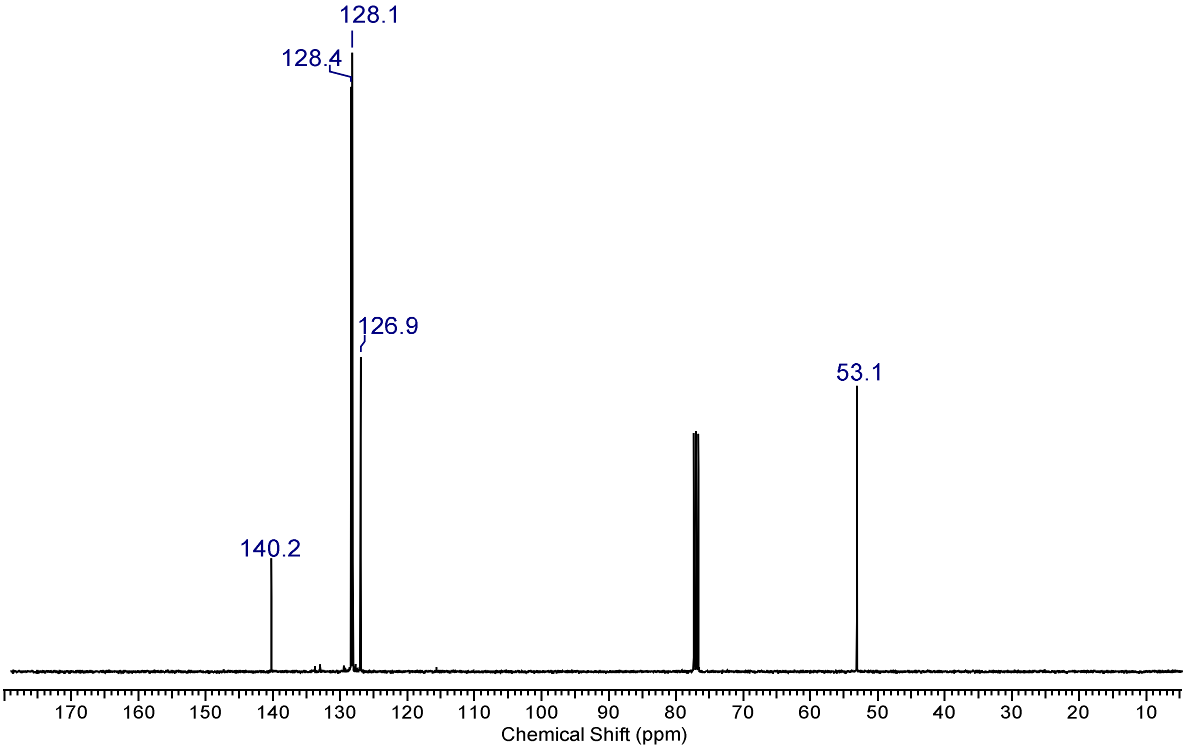

Figure S32. ^{13}C-NMR spectrum of the isolated dibenzylamine ($CDCl_3$).

5. Screening of the hydrosilanes as reductant

General Procedure: A J. Youngs NMR tube equipped with a DMSO-d_6 capillary was loaded with the BPh_3 (5.8 mg, 0.024 mmol, 0.10 eq.) in acetonitrile (0.5 mL), followed by the addition of benzaldehyde (25 μL, 0.241 mmol, 1.00 eq.), benzylamine (32 μL, 0.289 mmol, 1.20 eq.) and the appropriate silane (3.50 eq.). The reaction mixture was then heated at 100°C and monitored periodically by multinuclear spectroscopy. Subsequent addition of mesitylene (25 μL) to the reaction mixture allowed the determination of the NMR yields based on the protons attached to the α-carbons (δ = 3.8 ppm). The reaction mixture was then concentrated under reduced pressure and analysed by GC-MS analysis.

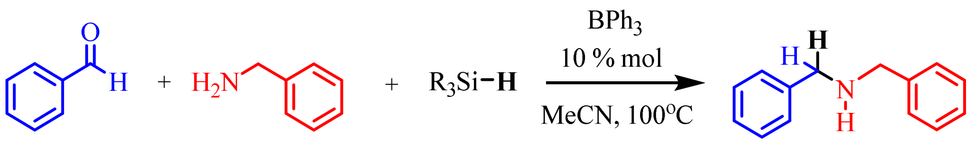

Entry	Silane	Time (h)	Yield (%)[a]
**1**	$PhMe_2SiH$	25	87
**2**	Ph_2MeSiH	25	8
**3**	$PhMeSiH_2$	25	55
**4**	$PhSiH_3$	20	56
**5**	Ph_2SiH_2	20	86

[a] NMR yield using mesitylene as standard.

Reductive amination using Ph_2MeSiH (Entry 2): Following the general procedure, a J. Youngs NMR tube was loaded with BPh_3 (5.8 mg, 0.024 mmol, 0.10 eq.) in acetonitrile (0.5 mL), followed by the addition of benzaldehyde (25 µL, 0.241 mmol, 1.00 eq.), benzylamine (32 µL, 0.289 mmol, 1.20 eq.) and methyldiphenylsilane (173 µL, 0.843 mmol, 3.50 eq.). After heating at 100°C for 25 hours, the NMR yield was 8%. GC-MS: *m/z* calculated for $C_{14}H_{15}N$, 197.1; found 197.1. GC-MS retention times of analyte: 10.42 minutes (Dibenzylamine).The data were in agreement with those reported in the literature.[3]

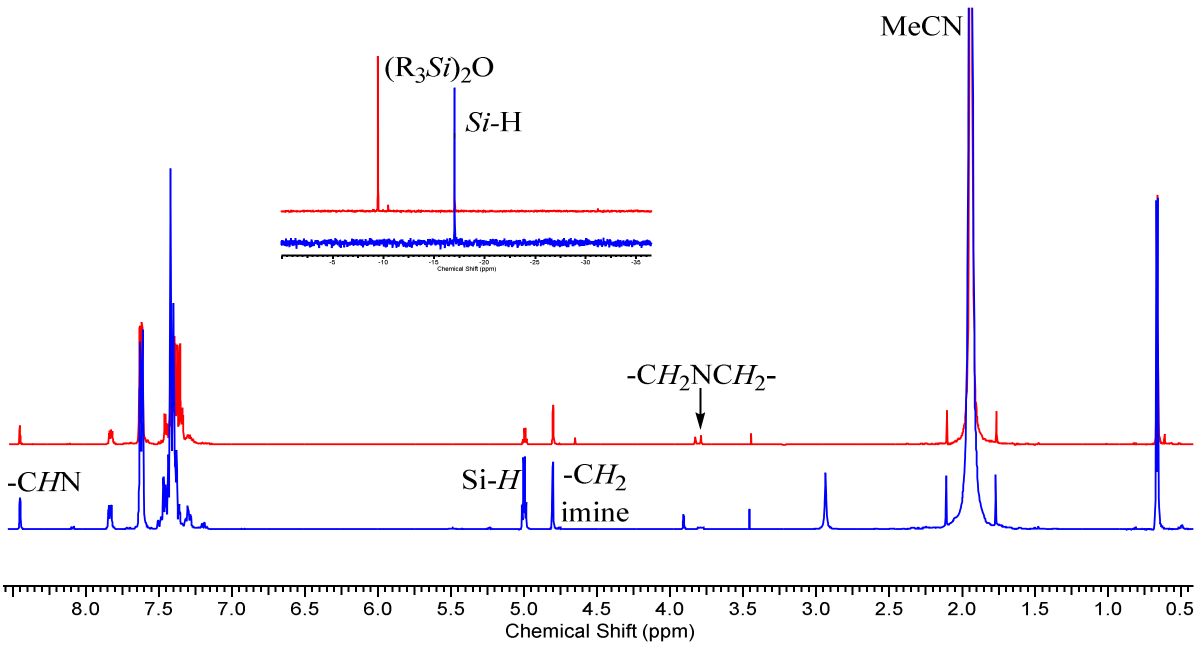

Figure S33. *In situ* ^{1}H and ^{29}Si-NMR spectra of the reductive amination of benzaldehyde/ benzylamine with BPh_3 in acetonitrile, using Ph_2MeSiH (3.50 eq.) as reductant. Blue (t = 5 min, r.t.), red (after 25 hours at 100°C).

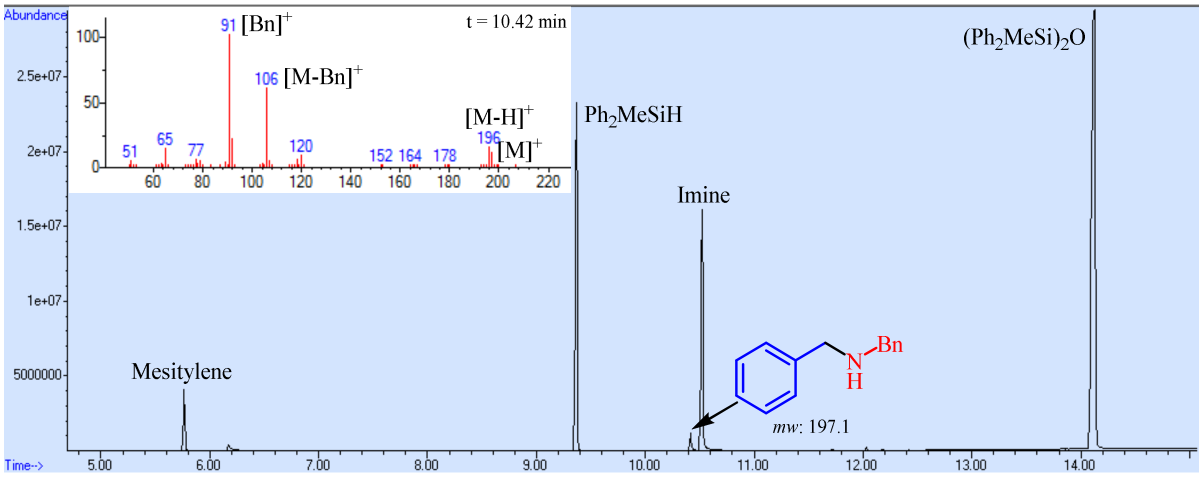

Figure S34. GC-MS of crude reaction.

Reductive amination using $PhMeSiH_2$ (Entry 3): Following the general procedure, a J. Youngs NMR tube was loaded with BPh_3 (5.8 mg, 0.024 mmol, 0.10 eq.) in acetonitrile (0.5 mL), followed by the addition of benzaldehyde (25 µL, 0.241 mmol, 1.00 eq.), benzylamine (32 µL, 0.289 mmol, 1.20 eq.) and methylphenylsilane (118 µL, 0.843 mmol, 3.50 eq.). After heating at 100°C for 25 hours, the NMR yield was 55%. GC-MS: *m/z* calculated for $C_{14}H_{15}N$, 197.1; found 197.1. GC-MS retention times of analyte: 10.42 minutes (Dibenzylamine).The data were in agreement with those reported in the literature.[3]

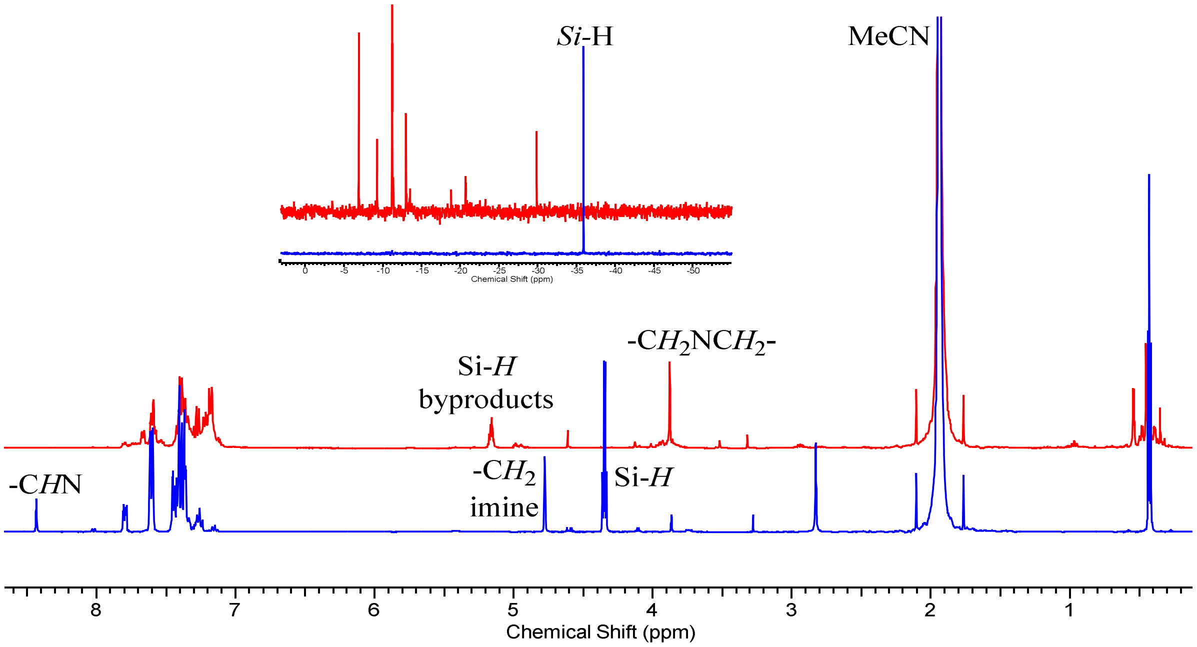

Figure S35. *In situ* ^{1}H and ^{29}Si-NMR spectra of the reductive amination of benzaldehyde/ benzylamine with BPh_3 in acetonitrile, using $PhMeSiH_2$ (3.50 eq.) as reductant. Blue (t = 5 min, r.t.), red (after 25 hours at 100°C).

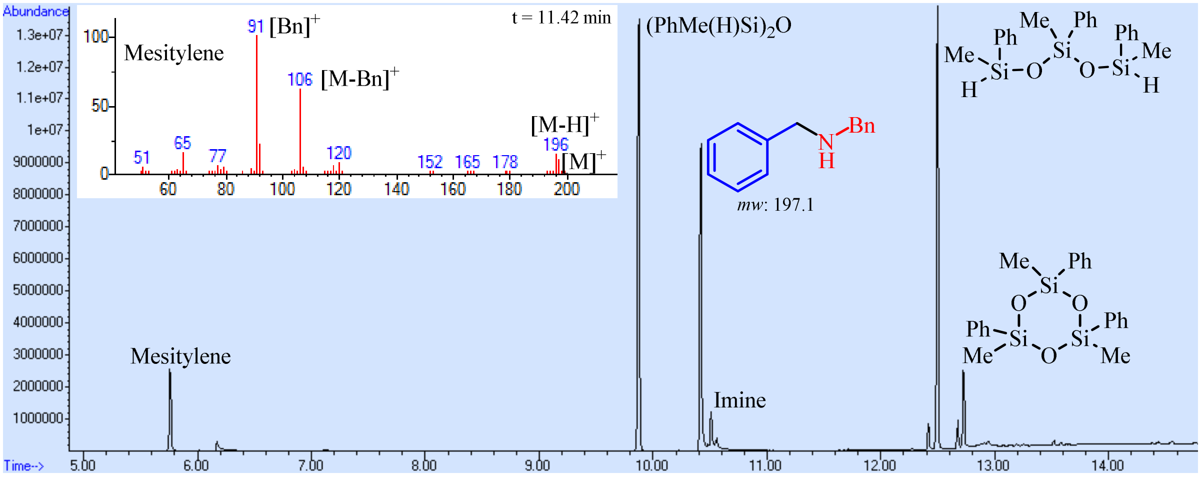

Figure S36. GC-MS of crude reaction.

Reductive amination using $PhSiH_3$ (Entry 4): Following the general procedure, a J. Youngs NMR tube was loaded with BPh_3 (5.8 mg, 0.024 mmol, 0.10 eq.) in acetonitrile (0.5 mL), followed by the addition of benzaldehyde (25 µL, 0.241 mmol, 1.00 eq.), benzylamine (32 µL, 0.289 mmol, 1.20 eq.) and phenylsilane (107 µL, 0.843 mmol, 3.50 eq.). After heating at 100°C for 20 hours, the NMR yield was 56%. GC-MS: *m/z* calculated for $C_{14}H_{15}N$, 197.1; found 197.1. GC-MS retention times of analyte: 10.42 minutes (Dibenzylamine).The data were in agreement with those reported in the literature.[3] Upon heating, a dark orange solution was obtained. $EtNH_2$ (from the reduction of MeCN) was also present in the reaction mixture, due to the high excess of H equivalent.

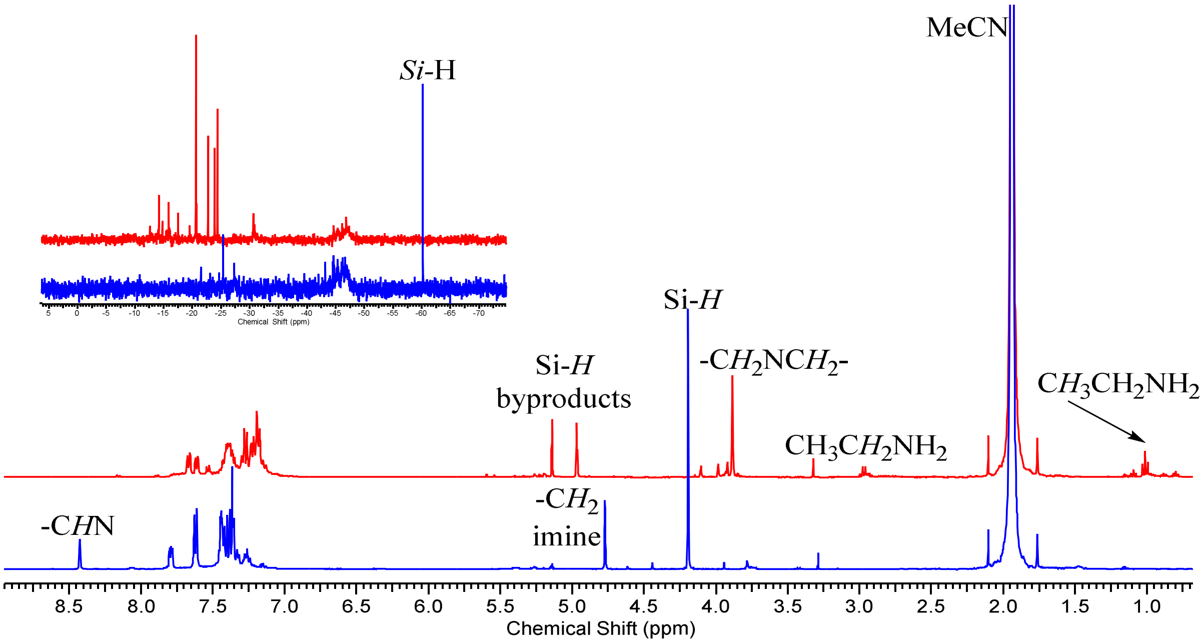

Figure S37. *In situ* ^{1}H and ^{29}Si-NMR spectra of the reductive amination of benzaldehyde/ benzylamine with BPh_3 in acetonitrile, using $PhSiH_3$ (3.50 eq.) as reductant. Blue (t = 5 min, r.t.), red (after 20 hours at 100°C).

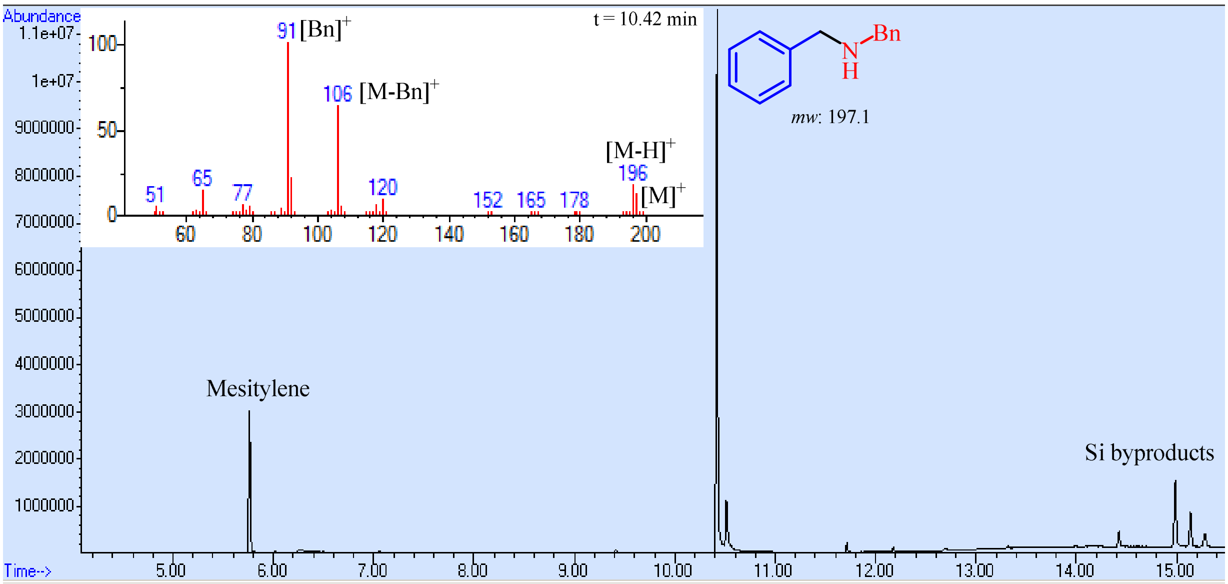

Figure S38. GC-MS of crude reaction.

Reductive amination using Ph_2SiH_2 (Entry 5): Following the general procedure, a J. Youngs NMR tube was loaded with BPh_3 (5.8 mg, 0.024 mmol, 0.10 eq.) in acetonitrile (0.5 mL), followed by the addition of benzaldehyde (25 µL, 0.241 mmol, 1.00 eq.), benzylamine (32 µL, 0.289 mmol, 1.20 eq.) and diphenylsilane (161 µL, 0.843 mmol, 3.50 eq.). After heating at 100°C for 20 hours, the NMR yield was 86%. GC-MS: *m/z* calculated for $C_{14}H_{15}N$, 197.1; found 197.1. GC-MS retention times of analyte: 10.42 minutes (Dibenzylamine).The data were in agreement with those reported in the literature.[3]

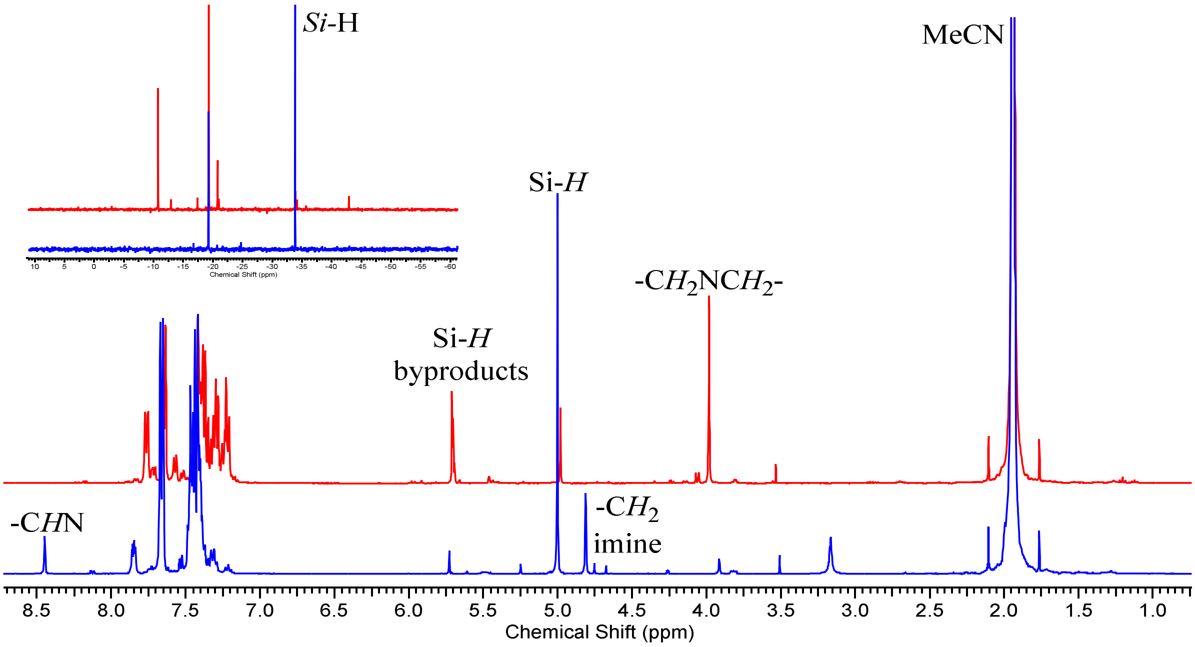

Figure S39. *In situ* ^{1}H and ^{29}Si-NMR spectra of the reductive amination of benzaldehyde/ benzylamine with BPh_3 in acetonitrile, using Ph_2SiH_2 (3.50 eq.) as reductant. Blue (t = 5 min, r.t.), red (after 20 hours at 100°C).

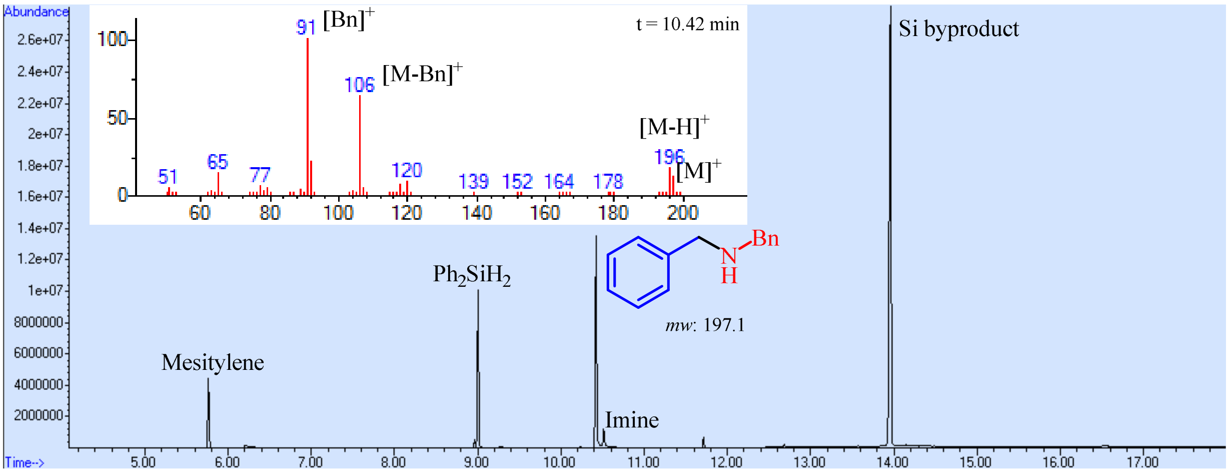

Figure S40. GC-MS of crude reaction.

6. Imine consumption *vs* water dehydrosilylation

Imine consumption: Following the general procedure, a J. Youngs NMR tube was loaded with BPh_3 (5.8 mg, 0.024 mmol, 0.10 eq.) in acetonitrile (0.5 mL), followed by the addition of benzaldehyde (25 μL, 0.241 mmol, 1.00 eq.), benzylamine (32 μL, 0.289 mmol, 1.20 eq.) and dimethylphenylsilane (132 μL, 0.843 mmol, 3.50 eq.). Then, mesitylene (25 μL, 0.179 mmol, 0.74 eq.) was added as internal standard to quantify the imine present. The reaction mixture was periodically monitored by ^{1}H-NMR spectroscopy after each 2 hours upon heating at 100°C (no induction period was observed).

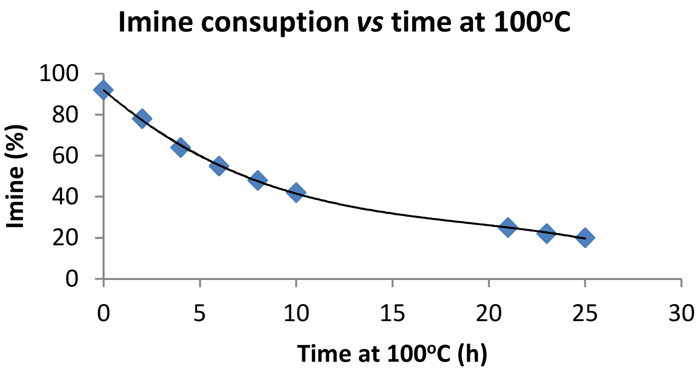

Figure S41. Imine consumption vs time at 100°C.

Water dehydrosilylation: A J. Youngs NMR tube equipped with a DMSO-d_6 capillary was loaded with BPh_3 (5.8 mg, 0.024 mmol, 0.10 eq.), water (5 μL, 0.241 mmol, 1.00 eq.) and dimethylphenylsilane (132 μL, 0.843 mmol, 3.50 eq.) in acetonitrile (0.5 mL). The reaction mixture was periodically monitored by ^{29}Si-NMR spectroscopy after each hour upon heating at 100°C (after 2 hours upon heating, siloxane formation was still in progress).

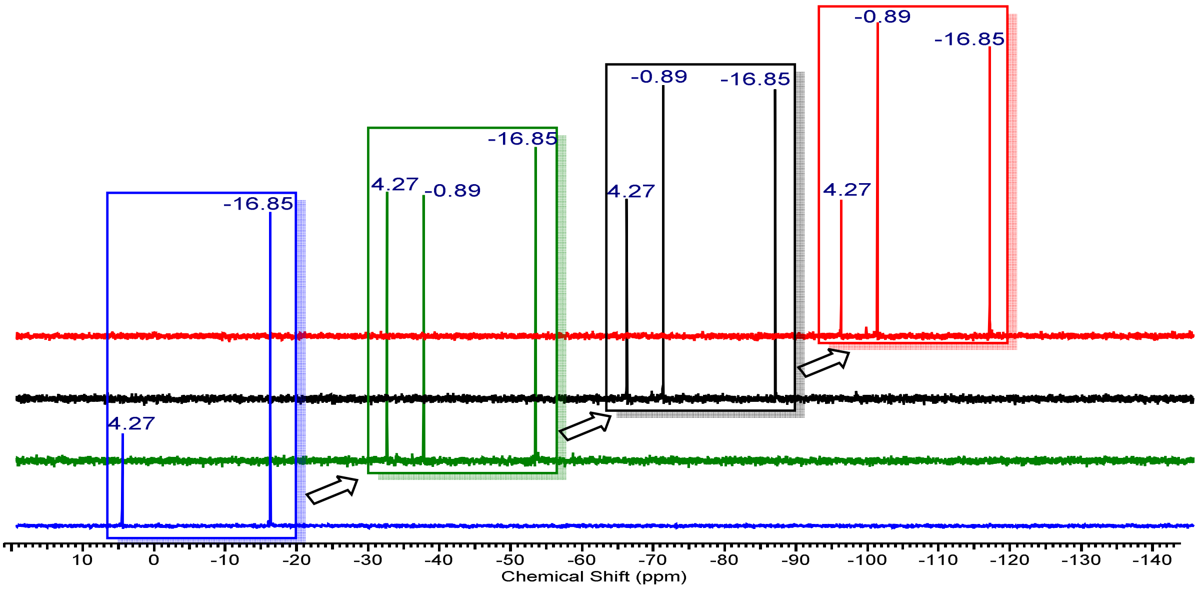

Figure S42. *In situ* ^{29}Si-NMR spectra of water dehydrosilylation in acetonitrile: -16.85 ppm = $PhMe_2SiH$, -0.89 ppm = $(PhMe_2Si)_2O$, 4.27 ppm = $PhMe_2SiOH$. Blue (t = 5 min, r.t.), green (after 1 hour at 100°C), black (after 2 hours at 100°C), red (after 3 hours at 100°C).

7. Reductive amination benzaldehyde/aniline with $B(C_6F_5)_3$ and BPh_3

$B(C_6F_5)_3$ as catalyst: Following the general procedure, a J. Youngs NMR tube was loaded with $B(C_6F_5)_3$ (6.5 mg, 0.012 mmol, 0.05 eq.) in acetonitrile (0.5 mL), followed by the addition of benzaldehyde (25 μL, 0.241 mmol, 1.00 eq.), aniline (27 μL, 0.289 mmol, 1.20 eq.) and dimethylphenylsilane (45 μL, 0.289 mmol, 1.20 eq.). After heating at 100°C for 1 hour, in the ^{1}H-NMR the signal of the protons attached to α-carbons appeared at δ = 4.31 ppm (NMR yield = 96%).. GC-MS: *m/z* calculated for $C_{13}H_{13}N$, 183.1; found 183.1. GC-MS retention times of analyte: 10.39 minutes (*N*-benzylaniline).The data were in agreement with those reported in the literature.[4]

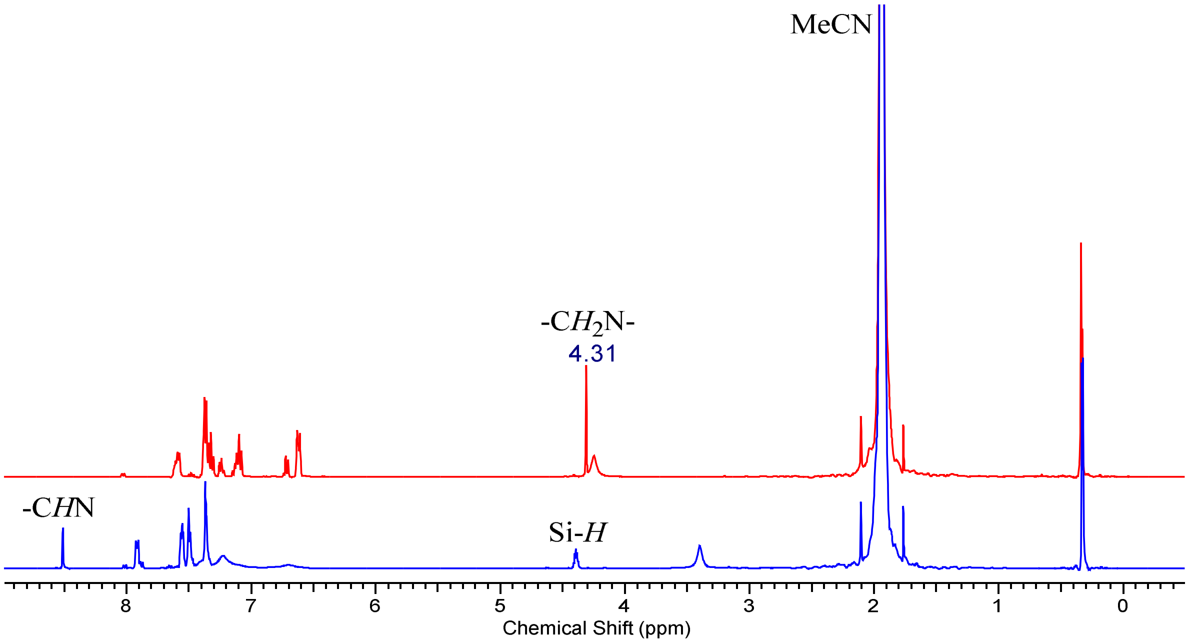

Figure S43. *In situ* ^{1}H-NMR spectra of the reductive amination of benzaldehyde/ aniline with $B(C_6F_5)_3$ in acetonitrile, using $PhMe_2SiH$ (1.20 eq.) as reductant. Blue (t = 5 min, r.t.), red (after 1 hour at 100°C).

BPh_3 as catalyst: Following the general procedure, a J. Youngs NMR tube was loaded with BPh_3 (3.1 mg, 0.012 mmol, 0.05 eq.) in acetonitrile (0.5 mL), followed by the addition of benzaldehyde (25 µL, 0.241 mmol, 1.00 eq.), aniline (27 µL, 0.289 mmol, 1.20 eq.) and dimethylphenylsilane (45 µL, 0.289 mmol, 1.20 eq.). After heating at 100°C for 1 hour, no significant reduction of the imine was observed by ^{1}H-NMR spectroscopy, while complete degradation of BPh_3 was noted in the ^{11}B-NMR spectra.

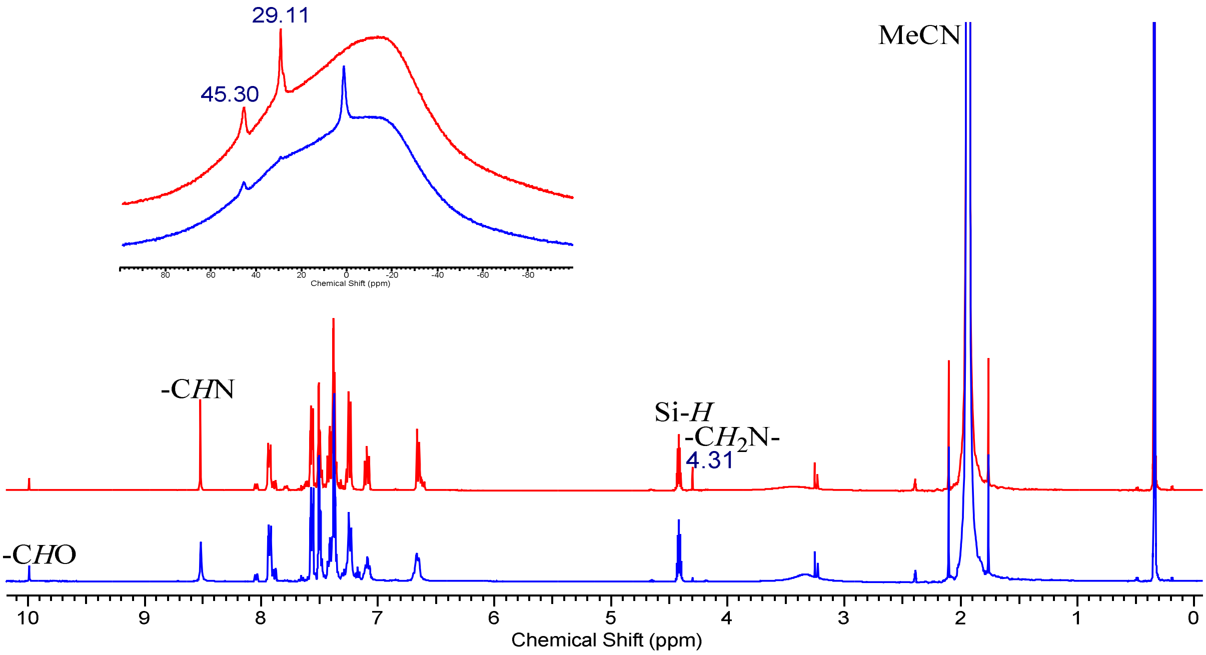

Figure S44. *In situ* ^{1}H and ^{11}B-NMR spectra of the reductive amination of benzaldehyde/ aniline with BPh_3 in acetonitrile, using $PhMe_2SiH$ (1.20 eq.) as reductant. Blue (t = 5 min, r.t.), red (after 1 hour at 100°C).

8. Hydrosilylation of *N*-benzylidene aniline and *N*-benzylidene benzylamine under anhydrous conditions.

General procedure: The reactions were carried out under nitrogen. Both acetonitrile and dimethylphenylsilane were distilled over CaH_2 for these reactions. A J. Youngs NMR tube equipped with a DMSO-d_6 capillary was loaded with BPh_3 (0.10 eq.) in acetonitrile (0.5 mL), followed by addition of the appropriate *N*-benzylidene amine (1.00 eq.) and dimethylphenylsilane (1.20 eq.). After the monitoring of the initial reaction mixture by multinuclear NMR spectroscopy, the reaction mixture was then heated at 100°C and periodically monitored by multinuclear spectroscopy. Upon heating at 100°C for 3 hours, the addition of mesitylene (25 μL) to the reaction mixture allowed the determination of the NMR conversion based on the relative integral of the benzylic protons attached to the aminated carbon. Due to the use of unpurified imine substrates, trace of siloxane could be detected in the ^{29}Si-NMR spectra.

***N*-benzylidene aniline**: A J. Youngs NMR tube equipped with a DMSO-d_6 capillary was loaded with BPh_3 (7.0 mg, 0.027 mmol, 0.10 eq.) in acetonitrile (0.5 mL), followed by addition of *N*-benzylidene aniline (50 mg, 0.273 mmol, 1.00 eq.) and dimethylphenylsilane (51 μL, 0.328 mmol, 1.20 eq.). NMR yield = 44%. Diagnostic peaks of BnN(Ph)$SiMe_2Ph$: ^{1}H-NMR (400 MHz, MeCN): δ 4.64 (s, 2H, -CH_2-), 0.51 (s, 6H, SiMe_2) ppm. ^{29}Si-NMR (81 MHz, MeCN): δ 1.26 ppm. The data were in accordance with those reported in the literature.[5] Adding 2.5 μL of water to the reaction mixture, almost quantitative hydrolysis of the product was observed after few minutes at r.t.

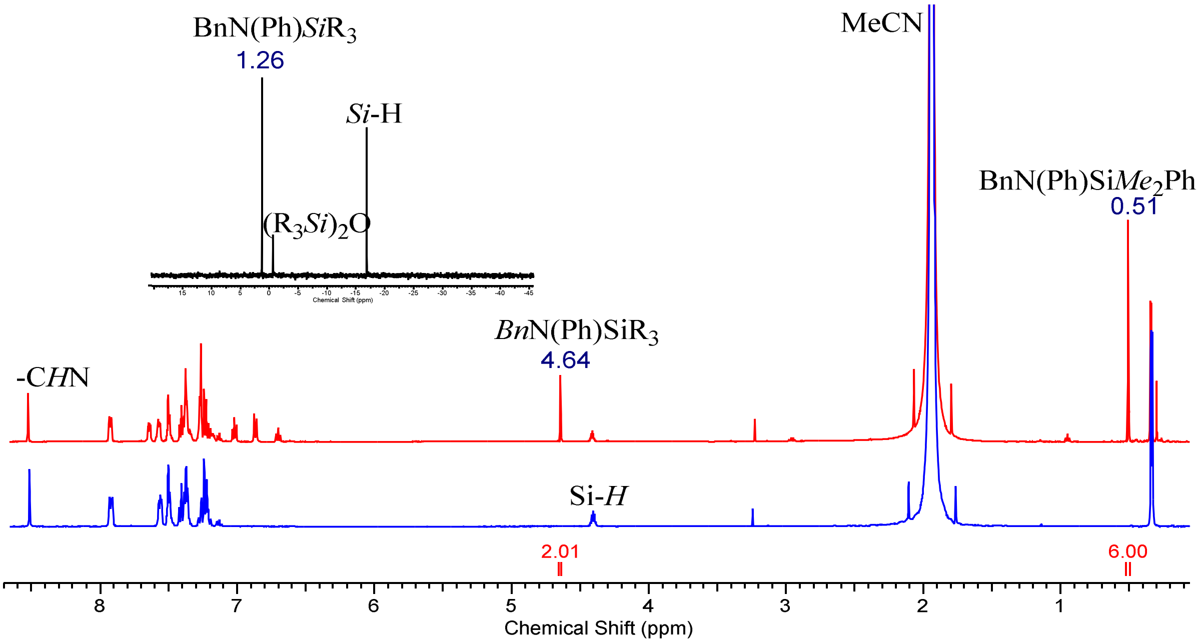

Figure S45. *In situ* ^{1}H-NMR spectra of the hydrosilylation of *N*-benzylidene aniline with $PhMe_2SiH$ using BPh_3 as catalyst in acetonitrile. Blue (t = 5 min, r.t.), red (after 5 hours at 100°C). In black, the ^{29}Si-NMR spectrum after 3 hours at 100°C.

***N*-benzylidene benzylamine**: A J. Youngs NMR tube equipped with a DMSO-d_6 capillary was loaded with BPh_3 (6.7 mg, 0.026 mmol, 0.10 eq.) in acetonitrile (0.5 mL), followed by addition of *N*-benzylidene benzylamine (50 µL, 0.263 mmol, 1.00 eq.) and dimethylphenylsilane (50 µL, 0.316 mmol, 1.20 eq.). NMR yield = 80%. Diagnostic peaks of Bn_2NSiMe_2Ph: ^{1}H-NMR (400 MHz, MeCN): δ 3.87 (s, 4H, -CH_2-), 0.49 (s, 6H, SiMe_2) ppm. ^{29}Si-NMR (81 MHz, MeCN): δ 0.55 ppm. The data were in accordance with those reported in the literature.[5] Adding 2.5 µL of water to the reaction mixture led to almost quantitative hydrolysis of the product was observed after few minutes at r.t.

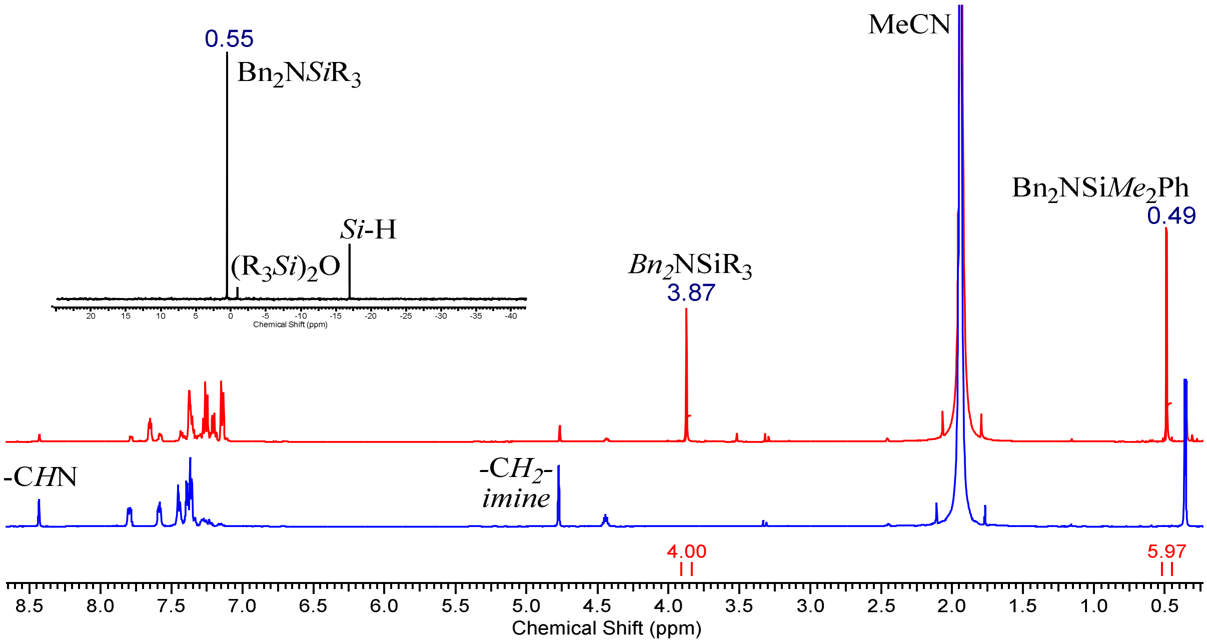

Figure S46. *In situ* ^{1}H-NMR spectra of the hydrosilylation of *N*-benzylidene benzylamine with $PhMe_2SiH$ using BPh_3 as catalyst in acetonitrile. Blue (t = 5 min, r.t.), red (after 3 hours at 100°C). In black, the ^{29}Si-NMR spectrum after 3 hours at 100°C.

9. Reduction of protonated imine with [Et_4N][$HBPh_3$] under anhydrous conditions.

***N*-benzylidene aniline**: A J. Youngs NMR tube equipped with a DMSO-d_6 capillary was loaded with *N*-benzylidene aniline (20 mg, 0.109 mmol, 1.00 eq.) and a 1M solution of hydrogen chloride in Et_2O (109 µL, 0.109 mmol, 1.00 eq.). Et_2O was then removed, leaving a yellow solid that partially redissolved in freshly distilled MeCN (0.5 mL). ^{1}H-NMR spectroscopy confirmed the formation of protonated imine (downfield of the aldimine proton). To the obtained system, [Et_4N][$HBPh_3$] (43 mg, 0.109 mmol, 1.00 eq.) was added. After few minutes at r.t., the reaction mixture became completely homogeneous. ^{1}H-NMR spectroscopy revealed partially reduction of the protonated imine to form the corresponding BnN(H)Ph (-CH_2- peak at 4.30 ppm). The formation of the product was also confirmed by GC-MS analysis (GC-MS: m/z calculated for $C_{13}H_{13}N$, 183.1; found 183.1. GC-MS retention times of analyte: 10.39 minutes (*N*-benzylaniline).

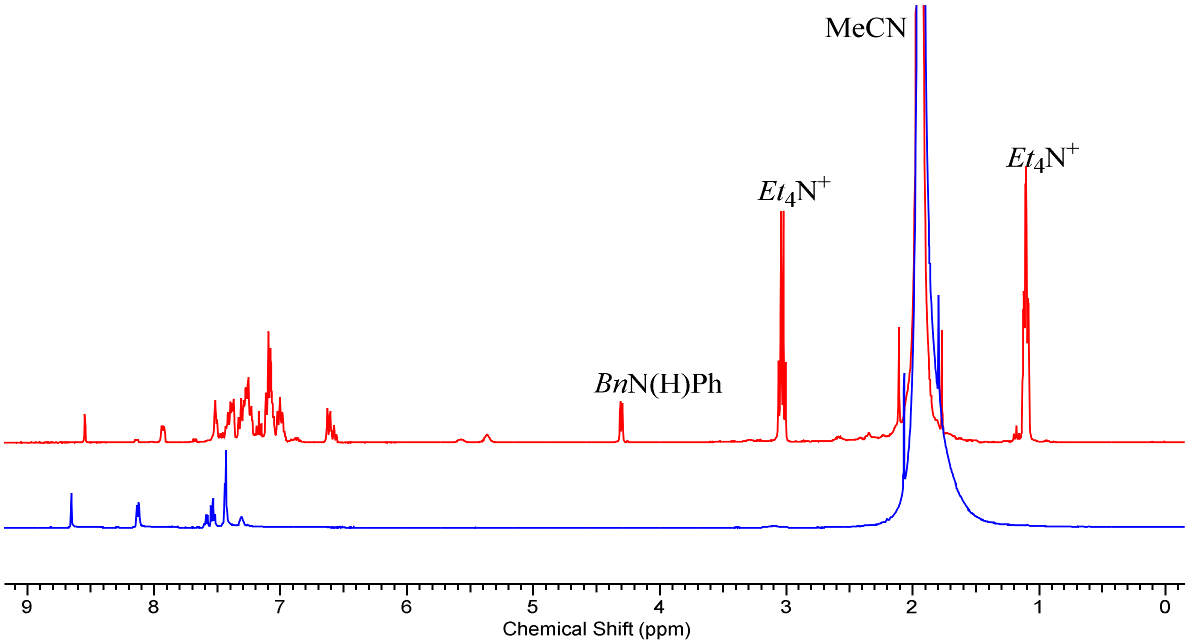

Figure S47. *In situ* ^{1}H-NMR spectra of the reduction of protonated *N*-benzylidene aniline with [Et_4N][$HBPh_3$] in acetonitrile. Blue (*N*-benzylidene aniline and HCl), red (few minutes after the addition of [Et_4N][$HBPh_3$]).

***N*-benzylidene benzylamine**: A J. Youngs NMR tube equipped with a DMSO-d_6 capillary was loaded with *N*-benzylidene benzylamine (21 μL, 0.110 mmol, 1.00 eq.) and a 1M solution of hydrogen chloride in Et_2O (110 μL, 0.110 mmol, 1.00 eq.). Et_2O was then removed, leaving a white solid that partially redissolved in freshly distilled MeCN (0.5 mL). ^1H-NMR spectroscopy confirmed the formation of protonated imine (downfield of the aldimine proton). To the obtained system, $[Et_4N][HBPh_3]$ (44 mg, 0.110 mmol, 1.00 eq.) was added. After few minutes at r.t., the reaction mixture became completely homogeneous. ^1H-NMR spectroscopy revealed almost quantitative reduction of the protonated imine to form the corresponding Bn_2NH (-CH_2- peak at 3.80 ppm). The formation of the product was also confirmed by GC-MS analysis (GC-MS: m/z calculated for $C_{14}H_{15}N$, 197.1; found 197.1. GC-MS retention times of analyte: 10.42 minutes (dibenzylamine).

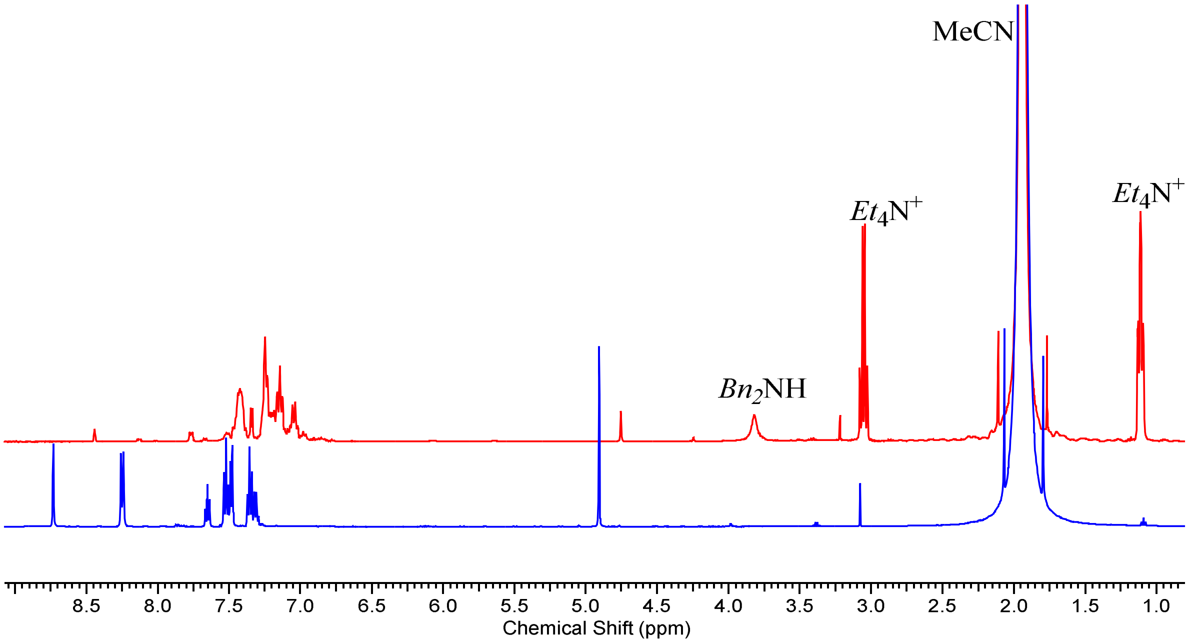

Figure S48. *In situ* ^1H-NMR spectra of the reduction of protonated *N*-benzylidene benzylamine with $[Et_4N][HBPh_3]$ in acetonitrile. Blue (*N*-benzylidene benzylamine and HCl), red (few minutes after the addition of $[Et_4N][HBPh_3]$).

10. Attempted imine reduction with Ph_2BOH, $PhB(OH)_2$ and Ph_3BOH^-

Ph_2BOH as catalyst: A J. Youngs NMR tube equipped with a DMSO-d_6 capillary was loaded with BPh_3 (5.3 mg, 0.022 mmol, 0.10 eq.) in acetonitrile, followed by addition of H_2O (4 µL, 0.221 mmol, 1.00 eq.). The system was heated at 100°C for 3 hours until full degradation of BPh_3. Then, *N*-benzylidene benzylamine (42 µL, 0.221 mmol, 1.20 eq.) and dimethylphenylsilane (121 µL, 0.774 mmol, 3.50 eq.) were added. The reaction mixture was heated at 100°C for 25 hours, but no significant reduction of the imine was observed by ^1H-NMR spectroscopy (imine left > 85%).

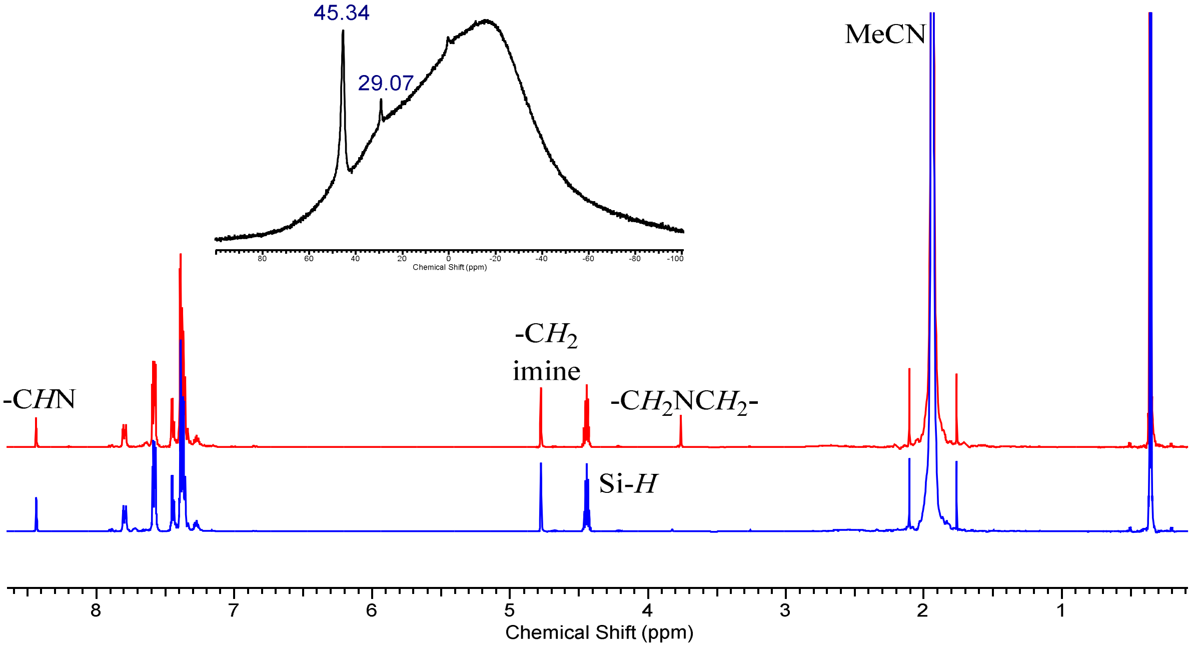

Figure S49. *In situ* ^1H-NMR spectra of *N*-benzylidene benzylamine reduction after BPh_3-OH_2 degradation in acetonitrile, using $PhMe_2SiH$ (3.50 eq.) as reductant. Blue (t = 5 min, r.t.), red (after 25 hours at 100°C). Inset, ^{11}B-NMR spectrum before imine/silane addition.

$PhB(OH)_2$ as catalyst: A J. Youngs NMR tube equipped with a DMSO-d_6 capillary was loaded with phenylboronic acid (2.9 mg, 0.024 mmol, 0.10 eq.) in acetonitrile, followed by addition of benzaldehyde (25 μL, 0.241 mmol, 1.00 eq.), benzylamine (32 μL, 0.289 mmol, 1.20 eq.) and dimethylphenylsilane (132 μL, 0.843 mmol, 3.50 eq.). The reaction mixture was then heated at 100°C for 25 hours, but no significant reduction of the imine was observed by ^{1}H-NMR spectroscopy (imine left > 85%).

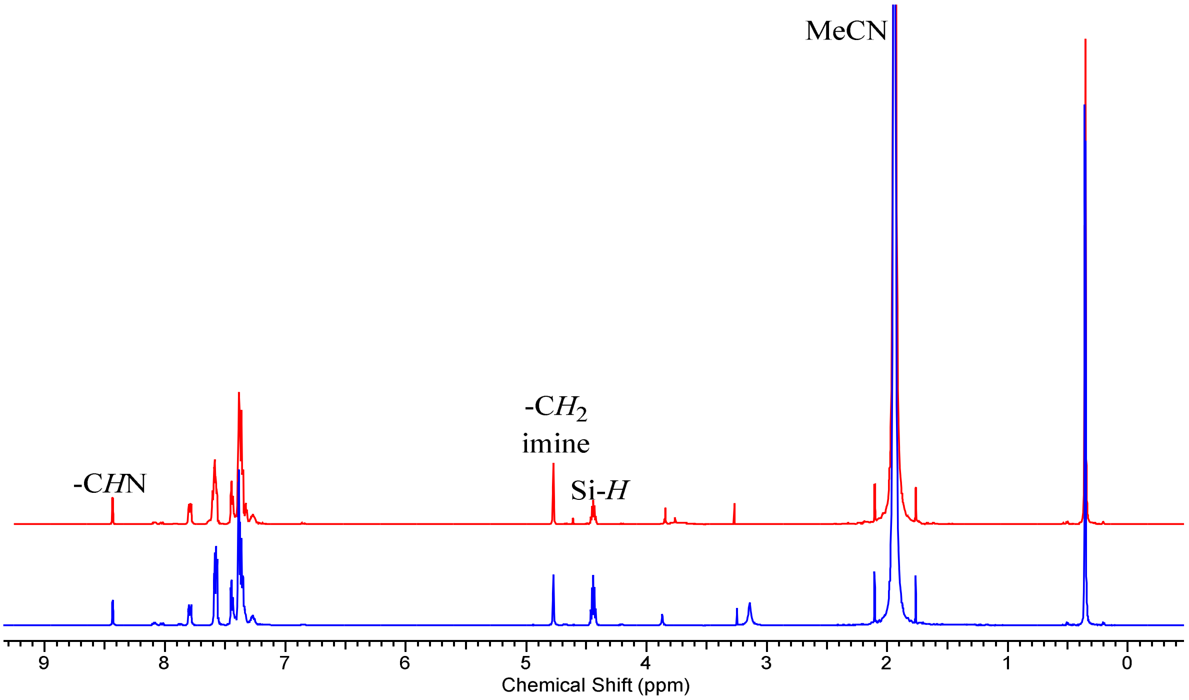

Figure S50. *In situ* ^{1}H-NMR spectra of the attempted reductive amination of benzaldehyde/ benzylamine with $PhB(OH)_2$ in acetonitrile, using $PhMe_2SiH$ (3.50 eq.) as reductant. Blue (t = 5 min, r.t.), red (after 25 hours at 100°C).

Ph_3BOH^- as catalyst: A J. Youngs NMR tube equipped with a DMSO-d_6 capillary was loaded with BPh_3 (8.9 mg, 0.035 mmol, 0.10 eq.) and $[Bu_4N][OH]\cdot 30H_2O$ (30 mg, 0.037 mmol, 0.11 eq.) in THF (0.5 mL). After initial monitoring by multinuclear spectroscopy, the system was dried in-vacuo and then redissolved in MeCN (0.5 mL). Then, benzaldehyde (36 μL, 0.350 mmol, 1.00 eq.), benzylamine (46 μL, 0.420 mmol, 1.20 eq.) and dimethylphenylsilane (192 μL, 1.225 mmol, 3.50 eq.) were added. The reaction mixture was heated at 100°C for 25 hours, but no significant reduction of the imine was observed by ^{1}H-NMR spectroscopy (imine left > 75%).

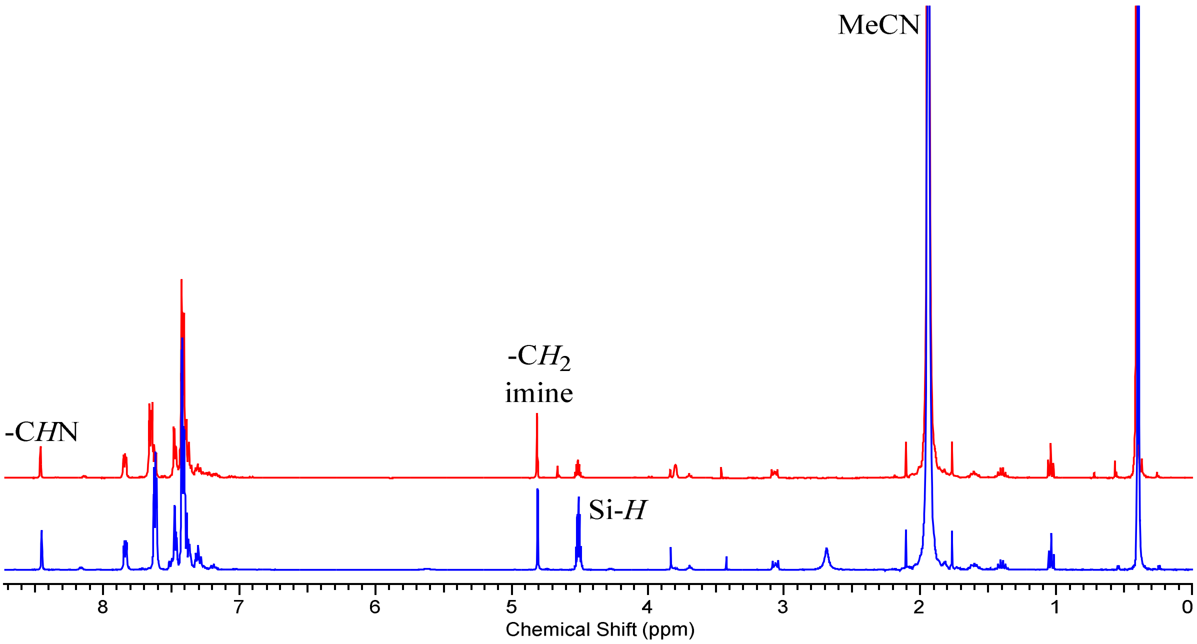

Figure S51. *In situ* ^{1}H-NMR spectra of *N*-benzylidene benzylamine reduction after BPh_3-OH_2 degradation in acetonitrile, using $PhMe_2SiH$ (3.50 eq.) as reductant. Blue (t = 5 min, r.t.), red (after 25 hours at 100°C). Inset, ^{11}B-NMR spectrum before imine/silane addition.

11. Attempted reductive amination using HNO_3 and HCl as catalysts

HNO_3 as catalyst: A J. Youngs NMR tube equipped with a DMSO-d_6 capillary was loaded with benzaldehyde (25 μL, 0.241 mmol, 1.00 eq.), benzylamine (32 μL, 0.289 mmol, 1.20 eq.) and dimethylphenylsilane (132 μL, 0.843 mmol, 3.50 eq.) in acetonitrile (0.5 mL), followed by the addition of a solution (70% wt./wt.) of HNO_3 (1.5 μL, 0.024 mmol, 0.10 eq.). The reaction mixture was then heated at 100°C for 25 hours, but no significant reduction of the imine was observed by ^{1}H-NMR spectroscopy.

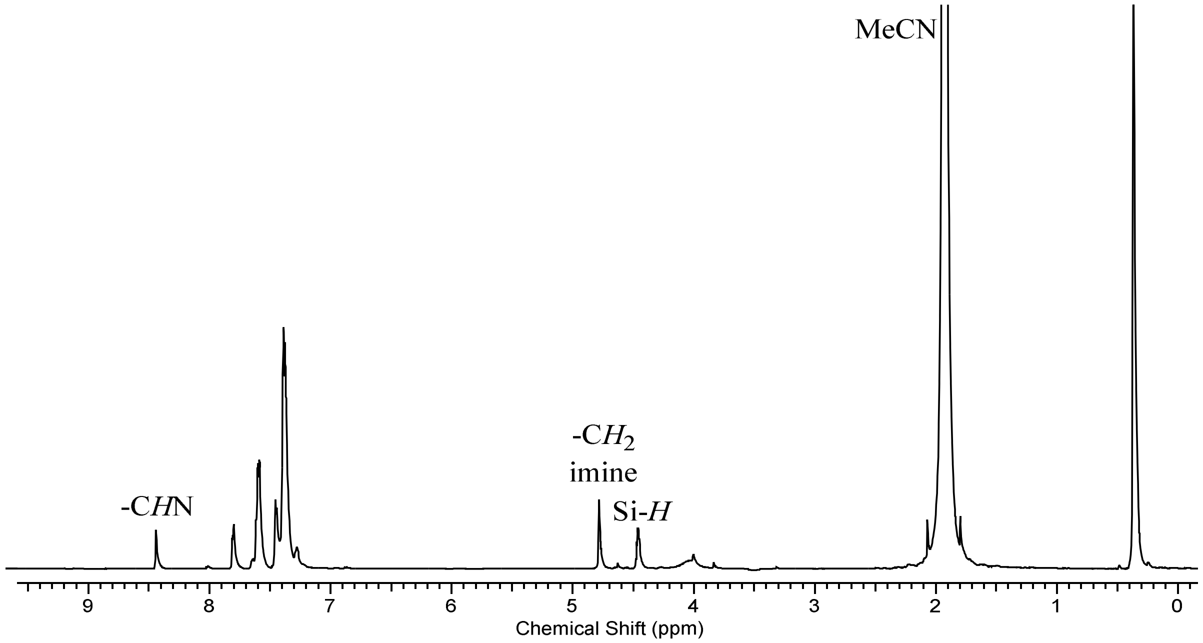

Figure S52. *In situ* ^{1}H-NMR spectrum of the attempted reductive amination of benzaldehyde/ benzylamine with HNO_3 in acetonitrile, using $PhMe_2SiH$ (3.50 eq.) as reductant, after 25 hours at 100°C.

HCl as catalyst: A J. Youngs NMR tube equipped with a DMSO-d_6 capillary was loaded with benzaldehyde (25 μL, 0.241 mmol, 1.00 eq.), benzylamine (32 μL, 0.289 mmol, 1.20 eq.) and dimethylphenylsilane (132 μL, 0.843 mmol, 3.50 eq.) in acetonitrile (0.5 mL), followed by the addition of a 4M solution of hydrogen chloride in 1,4-dioxane (6 μL, 0.024 mmol, 0.10 eq.). The reaction mixture was then heated at 100°C for 25 hours, but no significant reduction of the imine was observed by ^{1}H-NMR spectroscopy.

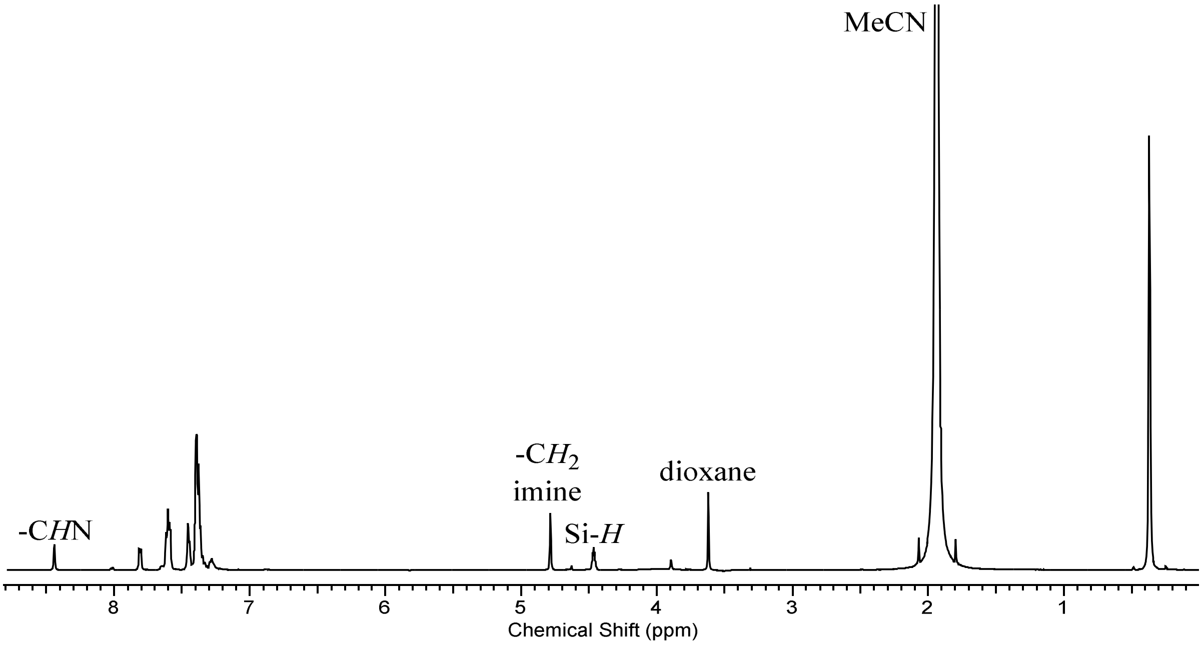

Figure S53. *In situ* ^{1}H-NMR spectrum of the attempted reductive amination of benzaldehyde/ benzylamine with HCl in acetonitrile, using $PhMe_2SiH$ (3.50 eq.) as reductant, after 25 hours at 100°C.

12. Carbonyl substrate scope

General Procedure: All reagents/solvent used without any purification. A J. Youngs NMR tube equipped with a DMSO-d_6 capillary was loaded with triphenylborane (0.10 eq.) in acetonitrile (0.5 mL). Then, the appropriate carbonyl compound (1.00 eq.), benzylamine (1.20 eq.) and dimethylphenylsilane (3.50 eq.) were added and the tube sealed. After the monitoring of the initial reaction mixture by multinuclear NMR spectroscopy, the J. Youngs NMR tube was heated up at 100°C for 25 hours (unless otherwise indicated) and then monitored by ^{1}H, ^{11}B and ^{29}Si-NMR spectroscopy. Subsequent addition of mesitylene (25 μL) to the reaction mixture allowed the determination of the NMR yields based on the protons attached to the α-carbons. The reaction mixture was then concentrated under reduced pressure and the crude analysed by multinuclear NMR spectroscopy and by GC-MS analysis. Both analysis revealed the main present species were the $PhMe_2SiOH$ (^{29}Si NMR, δ ~ 3.5 ppm), $(PhMe_2Si)_2O$ (^{29}Si NMR δ ~ -1.0 ppm) and the desired product. Some reactions were purified by flash column chromatography on silica gel, obtaining isolated yields in good agreement with the NMR yields. Due to the overlap with siloxane/silanol aromatic resonances, the *in-situ* data reported are referred to the diagnostic peaks of the aliphatic region.

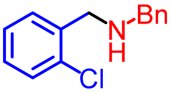

***N*-benzyl-1-(2-chlorophenyl)methanamine (1a):** Following the general procedure, a J. Youngs NMR tube was loaded with triphenylborane (6.4 mg, 0.026 mmol, 0.10 eq.) in acetonitrile (0.5 mL), followed by the addition of 2-chlorobenzaldehyde (30 μL, 0.264 mmol, 1.00 eq.), benzylamine (35 μL, 0.316 mmol, 1.20 eq.) and dimethylphenylsilane (145 μL, 0.923 mmol, 3.50 eq.). After heating at 100°C for 25 hours, in the ^{1}H NMR spectrum the signals of the protons attached to α-carbons appeared at δ = 3.86 and 3.79 ppm (NMR yield = 80%). After concentration under reduced pressure, in the crude the main species detected were $(PhMe_2Si)_2O$ and *N*-benzyl-1-(2-chlorophenyl)methanamine. The crude was purified by flash column chromatography (Pet. Et. : EtOAc 9:1) obtaining **1a** as yellow oil (45 mg, 0.194 mmol, 74%). R_f = 0.32. ^{1}H NMR ($CDCl_3$, 400 MHz, 298 K): δ 7.39-7.26 (m, 6H), 7.24-7.13 (m, 3H), 3.87 (s, 2H), 3.77 (s, 2H), 1.73 (br, 1H) ppm. ^{13}C{^{1}H} NMR ($CDCl_3$, 100 MHz, 298 K): 140.1, 137.6, 133.8, 130.2, 129.5, 128.4, 128.3, 128.2, 127.0, 126.8, 53.1, 50.7 ppm. GC-MS: *m/z* calculated for $C_{14}H_{14}ClN$, 231.1; found 231.1. GC-MS retention times of analyte: 11.20 minutes (*N*-benzyl-1-(2-chlorophenyl)methanamine). The data were in agreement with those reported in the literature.[6]

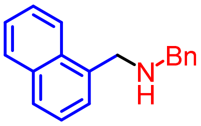

***N*-benzyl-1-(naphthalen-1-yl)methanamine (1b):** Following the general procedure, a J. Youngs NMR tube was loaded with triphenylborane (6.2 mg, 0.025 mmol, 0.10 eq.) in acetonitrile (0.5 mL), followed by the addition of 1-naphthaldehyde (35 μL, 0.245 mmol, 1.00 eq.), benzylamine (32 μL, 0.294 mmol, 1.20 eq.) and dimethylphenylsilane (134 μL, 0.857 mmol, 3.50 eq.). After heating at 100°C for 25 hours, in the ^{1}H NMR spectrum the signals of the protons attached to α-carbons appeared at δ = 4.19 and 3.87 ppm (NMR yield = 96%). After concentration under reduced pressure, in the crude the main species detected were

($(PhMe_2Si)_2O$ and *N*-benzyl-1-(naphthalen-1-yl)methanamine. The crude was purified by flash column chromatography (Pet. Et. : EtOAc 9:1) obtaining **1b** as yellow oil (58 mg, 0.234 mmol, 95%). R_f = 0.30. ^{1}H NMR ($CDCl_3$, 400 MHz, 298 K): δ 8.12 (d, *J* = 8.0 Hz, 1H), 7.90 (d, *J* = 7.3 Hz, 1H), 7.81 (d, *J* = 8.0 Hz, 1H), 7.58-7.49 (m, 3H), 7.47 (d, *J* = 7.0 Hz, 1H), 7.42-7.36 (m, 4H), 7.33-7.28 (m, 1H), 4.29 (s, 2H), 3.96 (s, 2H), 1.88 (br, 1H) ppm. ^{13}C{^{1}H} NMR ($CDCl_3$, 100 MHz, 298 K): 140.2, 135.7, 133.9, 131.8, 128.7, 128.4, 128.2, 127.8, 127.0, 126.1, 126.0, 125.6, 125.3, 123.7, 53.6, 50.8 ppm. GC-MS: *m/z* calculated for $C_{18}H_{17}N$, 247.1; found 247.1. GC-MS retention times of analyte: 12.73 minutes (*N*-benzyl-1-(naphthalen-1-yl)methanamine). The data were in agreement with those reported in the literature.[7]

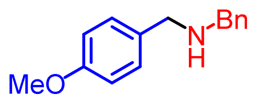

***N*-benzyl-1-(4-methoxyphenyl)methanamine (1c):** Following the general procedure, a J. Youngs NMR tube was loaded with triphenylborane (5.8 mg, 0.024 mmol, 0.10 eq.) in acetonitrile (0.5 mL), followed by the addition of 4-methoxybenzaldehyde (30 μL, 0.242 mmol, 1.00 eq.), benzylamine (32 μL, 0.290 mmol, 1.20 eq.) and dimethylphenylsilane (132 μL, 0.846 mmol, 3.50 eq.). After heating at 100°C for 25 hours, in the ^{1}H NMR spectrum the signals of the protons attached to α-carbons appeared at δ = 3.76 and 3.71 ppm (NMR yield = 98%). After concentration under reduced pressure, in the crude the main species detected were $(PhMe_2Si)_2O$ and *N*-benzyl-1-(4-methoxyphenyl)methanamine. The crude was purified by flash column chromatography (Pet. Et. : EtOAc 9:1) obtaining **1c** as yellow oil (52 mg, 0.229 mmol, 95%). R_f = 0.25. ^{1}H NMR ($CDCl_3$, 400 MHz, 298 K): δ 7.36-7.29 (m, 4H), 7.28-7.22 (m, 1H), 7.26 (d, *J* = 8.5 Hz, 2H), 6.87 (d, *J* = 8.5 Hz, 2H), 3.80 (s, 2H + 3H), 3.75 (s, 2H), 1.67 (br, 1H) ppm. ^{13}C{^{1}H} NMR ($CDCl_3$, 100 MHz, 298 K): 158.6, 140.3, 132.4, 129.3, 128.3, 128.1, 126.9, 113.7, 55.2, 53.0, 52.5 ppm. GC-MS: *m/z* calculated for $C_{15}H_{17}NO$, 227.1; found 227.1. GC-MS retention times of analyte: 11.56 minutes (*N*-benzyl-1-(4-methoxyphenyl)methanamine). The data were in agreement with those reported in the literature.[8]

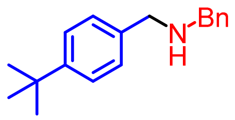

***N*-benzyl-1-(4-(tert-butyl)phenyl)methanamine (1d):** Following the general procedure, a J. Youngs NMR tube was loaded with triphenylborane (6.3 mg, 0.026 mmol, 0.10 eq.) in acetonitrile (0.5 mL), followed by the addition of 4-tert-butylbenzaldehyde (45 μL, 0.261 mmol, 1.00 eq.), benzylamine (35 μL, 0.313 mmol, 1.20 eq.) and dimethylphenylsilane (143 μL, 0.913 mmol, 3.50 eq.). After heating at 100°C for 25 hours, in the ^{1}H NMR spectrum the signals of the protons attached to α-carbons appeared at δ = 3.78 and 3.75 ppm (NMR yield = 77%). After concentration under reduced pressure, in the crude the main species detected were $(PhMe_2Si)_2O$ and *N*-benzyl-1-(4-(tert-butyl)phenyl)methanamine. ^{1}H NMR ($CDCl_3$, 400 MHz, 298 K): δ 3.85 (s, 2H, C*H*$_2$), 3.83 (s, 2H, C*H*$_2$), 1.40 (s, 9H, *tBu*) ppm. ^{13}C{^{1}H} NMR ($CDCl_3$, 100 MHz, 298 K): 52.9 (1C, *C*H$_2$), 52.5 (1C, *C*H$_2$), 31.3 (3C, *tBu*) ppm. GC-MS: *m/z* calculated for $C_{18}H_{23}N$, 253.1; found 253.1. GC-MS retention times of analyte: 11.81 minutes (*N*-benzyl-1-(4-(tert-butyl)phenyl)methanamine). The data were in agreement with those reported in the literature.[9]

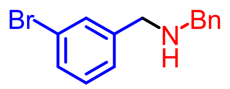

***N*-benzyl-1-(3-bromophenyl)methanamine (1e):** Following the general procedure, a J. Youngs NMR tube was loaded with triphenylborane (6.0 mg, 0.025 mmol, 0.10 eq.) in acetonitrile (0.5 mL), followed by the addition of 3-bromobenzaldehyde (30 µL, 0.250 mmol, 1.00 eq.), benzylamine (33 µL, 0.299 mmol, 1.20 eq.) and dimethylphenylsilane (137 µL, 0.874 mmol, 3.50 eq.). After heating at 100°C for 25 hours, in the ^{1}H NMR spectrum the signals of the protons attached to α-carbons appeared at δ = 3.75 and 3.73 ppm (NMR yield = 85%). After concentration under reduced pressure, in the crude the main species detected were $(PhMe_2Si)_2O$ and *N*-benzyl-1-(3-bromophenyl)methanamine. ^{1}H NMR ($CDCl_3$, 400 MHz, 298 K): δ 3.91 (s, 2H, C*H₂*), 3.88 (s, 2H, C*H₂*) 2.25 (br, 1H) ppm. ^{13}C{^{1}H} NMR ($CDCl_3$, 100 MHz, 298 K): 53.0 (1C, *C*H₂), 52.4 (1C, *C*H₂) ppm. GC-MS: *m/z* calculated for $C_{14}H_{14}BrN$, 275.0; found 275.0. GC-MS retention times of analyte: 11.72 minutes (*N*-benzyl-1-(3-bromophenyl)methanamine). The data were in agreement with those reported in the literature.[8]

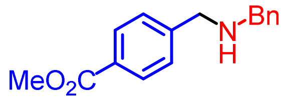

Methyl 4-((benzylamino)methyl)benzoate (1f): Following the general procedure, a J. Youngs NMR tube was loaded with triphenylborane (5.8 mg, 0.024 mmol, 0.10 eq.) in acetonitrile (0.5 mL), followed by the addition of methyl 4-formylbenzoate (40 mg, 0.239 mmol, 1.00 eq.), benzylamine (32 µL, 0.287 mmol, 1.20 eq.) and dimethylphenylsilane (131 µL, 0.836 mmol, 3.50 eq.). After heating at 100°C for 25 hours, in the ^{1}H NMR spectrum the signals of the protons attached to α-carbons appeared at δ = 3.81 and 3.75 ppm (NMR yield = 92%). After concentration under reduced pressure, in the crude the main species detected were $(PhMe_2Si)_2O$ and methyl 4-((benzylamino)methyl)benzoate. ^{1}H NMR ($CDCl_3$, 400 MHz, 298 K): δ 3.98 (s, 3H, -OCH_3), 3.92 (s, 2H, CH_2), 3.87 (s, 2H, CH_2) ppm. ^{13}C{^{1}H} NMR ($CDCl_3$, 100 MHz, 298 K): 53.0 (1C, -O*C*H₃), 52.5 (1C, *C*H₂), 51.9 (1C, *C*H₂) ppm. GC-MS: *m/z* calculated for $C_{16}H_{17}NO_2$, 255.1; found 255.1. GC-MS retention times of analyte: 12.38 minutes (methyl 4-((benzylamino)methyl)benzoate). The data were in agreement with those reported in the literature.[9]

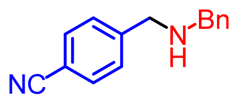

4-((benzylamino)methyl)benzonitrile (1g): Following the general procedure, a J. Youngs NMR tube was loaded with triphenylborane (6.2 mg, 0.025 mmol, 0.10 eq.) in acetonitrile (0.5 mL), followed by the addition of 4-formylbenzonitrile (34 mg, 0.254 mmol, 1.00 eq.), benzylamine (34 µL, 0.305 mmol, 1.20 eq.) and dimethylphenylsilane (139 µL, 0.889 mmol, 3.50 eq.). After heating at 100°C for 25 hours, in the ^{1}H NMR spectrum the signals of the protons attached to α-carbons appeared at δ = 3.79 and 3.73 ppm (NMR yield = 70%). After concentration under reduced pressure, in the crude the main species detected were $(PhMe_2Si)_2O$ and 4-((benzylamino)methyl)benzonitrile. ^{1}H NMR ($CDCl_3$, 400 MHz, 298 K): δ 3.69 (s, 2H, CH_2), 3.64 (s, 2H, CH_2) ppm. ^{13}C{^{1}H} NMR ($CDCl_3$, 100 MHz, 298 K): 53.0 (1C, *C*H₂), 52.3 (1C, *C*H₂) ppm. GC-MS: *m/z* calculated for $C_{15}H_{14}N_2$, 222.1; found 222.1. GC-MS retention times of analyte: 11.97 minutes (4-((benzylamino)methyl)benzonitrile). The data were in agreement with those reported in the literature.[10]

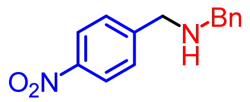

***N*-benzyl-1-(4-nitrophenyl)methanamine (1h):** Following the general procedure, a J. Youngs NMR tube was loaded with triphenylborane (6.3 mg, 0.026 mmol, 0.10 eq.) in acetonitrile (0.5 mL), followed by the addition of 4-nitrobenzaldehyde (40 mg, 0.259 mmol, 1.00 eq.), benzylamine (34 µL, 0.311 mmol, 1.20 eq.) and dimethylphenylsilane (142 µL, 0.908 mmol, 3.50 eq.). After heating at 100°C for 25 hours, in the ^{1}H NMR spectrum the signals of the protons attached to α-carbons appeared at δ = 3.84 and 3.75 ppm (NMR yield = 32%). After concentration under reduced pressure, in the crude the main species detected were $(PhMe_2Si)_2O$, dibenzylamine and *N*-benzyl-1-(4-nitrophenyl)methanamine. The formation of dibenzylamine was not prevented neither using 1.20 eq. of silane or reducing the reaction time and its formation is attributed to trans-imination during the reaction. ^{1}H NMR ($CDCl_3$, 400 MHz, 298 K): δ 3.73 (s, 2H, -CH_2-), 3.66 (s, 2H, -CH_2-) ppm. ^{13}C{^{1}H} NMR ($CDCl_3$, 100 MHz, 298 K): 53.0 (1C, *C*H_2), 52.0 (1C, *C*H_2) ppm. GC-MS: *m/z* calculated for $C_{14}H_{14}N_2O_2$, 242.1; found 242.1. GC-MS retention times of analyte: 12.48 minutes (*N*-benzyl-1-(4-nitrophenyl)methanamine). The data were in agreement with those reported in the literature.[11]

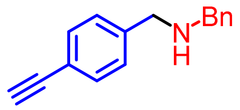

***N*-benzyl-1-(4-ethynylphenyl)methanamine (1i):** Following the general procedure, a J. Youngs NMR tube was loaded with triphenylborane (5.8 mg, 0.024 mmol, 0.10 eq.) in acetonitrile (0.5 mL), followed by the addition of methyl 4-ethynylbenzaldehyde (32 mg, 0.241 mmol, 1.00 eq.), benzylamine (32 µL, 0.289 mmol, 1.20 eq.) and dimethylphenylsilane (132 µL, 0.843 mmol, 3.50 eq.). After heating at 100°C for 25 hours, in the ^{1}H NMR spectrum the signals of the protons attached to α-carbons appeared at δ = 3.76 and 3.74 ppm (NMR yield = 94%). After concentration under reduced pressure, in the crude the main species detected were $(PhMe_2Si)_2O$ and *N*-benzyl-1-(4-ethynylphenyl)methanamine. ^{1}H NMR ($CDCl_3$, 400 MHz, 298 K): δ 3.90 (s, 2H, CH_2), 3.89 (s, 2H, CH_2) ppm. ^{13}C{^{1}H} NMR ($CDCl_3$, 100 MHz, 298 K): 83.6 (1C, -CCH), 76.9 (1C, -CCH), 53.0 (1C, *C*H_2), 52.6 (1C, *C*H_2) ppm. GC-MS: *m/z* calculated for $C_{16}H_{15}N$, 221.1; found 221.1. GC-MS retention times of analyte: 11.39 minutes (*N*-benzyl-1-(4-ethynylphenyl)methanamine). The data were in agreement with those reported in the literature.[12]

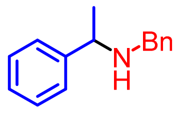

***N*-benzyl-1-phenylethan-1-amine (1j):** Following the general procedure, a J. Youngs NMR tube was loaded with triphenylborane (5.3 mg, 0.021 mmol, 0.10 eq.) in acetonitrile (0.5 mL), followed by the addition of acetophenone (25 µL, 0.210 mmol, 1.00 eq.), benzylamine (28 µL, 0.252 mmol, 1.20 eq.) and dimethylphenylsilane (115 µL, 0.735 mmol, 3.50 eq.). After heating at 100°C for 48 hours, in the ^{1}H NMR spectrum the signals of the protons attached to α-carbons appeared at δ = 3.78, 3.60, and 3.58 ppm (NMR yield = 75%). After concentration under reduced pressure, in the crude the main species detected were $(PhMe_2Si)_2O$ and *N*-benzyl-1-phenylethan-1-amine. ^{1}H NMR ($CDCl_3$, 400 MHz, 298 K): δ 3.73 (q, *J* = 6.6 Hz, 1H, C*H*), 3.57 (d, *J* = 13.1 Hz, 1H, NCH_aH_b), 3.51 (d, *J* = 13.1 Hz, 1H, NCH_aH_b), 1.28 (d, *J* = 6.6 Hz, 3H, -CH_3) ppm. Note that the benzylic protons are diastereotopic as reported in the literature. ^{13}C{^{1}H} NMR ($CDCl_3$, 100 MHz, 298 K): 57.4 (1C, *C*H_2), 51.5 (1C, *C*H), 24.4 (1C, -*C*H_3) ppm. GC-MS: *m/z* calculated for $C_{15}H_{17}N$, 211.1; found 211.1. GC-MS retention times of analyte: 10.39 minutes

(*N*-benzyl-1-phenylethan-1-amine). The data were in agreement with those reported in the literature.[13]

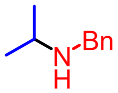

N-benzylpropan-2-amine (1k): Following the general procedure, a J. Youngs NMR tube was loaded with triphenylborane (6.9 mg, 0.027 mmol, 0.10 eq.) in acetonitrile (0.5 mL), followed by the addition acetone (20 µL, 0.270 mmol, 1.00 eq.), benzylamine (36 µL, 0.324 mmol, 1.20 eq.) and dimethylphenylsilane (148 µL, 0.944 mmol, 3.50 eq.). After heating at 100°C for 25 hours, in the ^{1}H NMR spectrum the signals of the protons attached to α-carbons appeared at δ = 3.79 and 2.83 ppm (NMR yield = 92%). After concentration under reduced pressure, in the crude the main species detected were $(PhMe_2Si)_2O$ and *N*-benzylpropan-2-amine. ^{1}H NMR ($CDCl_3$, 400 MHz, 298 K): δ 3.80 (s, 2H, CH_2), 2.88 (sept, J = 6.3 Hz, 1H, C*H*), 1.13 (d, J = 6.3 Hz, 6H, CH_3) ppm. ^{13}C{^{1}H} NMR ($CDCl_3$, 100 MHz, 298 K): 51.5 (1C, *C*H$_2$), 48.0 (1C, *C*H), 22.8 (2C, *C*H$_3$) ppm. GC-MS: *m/z* calculated for $C_{10}H_{15}N$, 149.1; found 149.1. GC-MS retention times of analyte: 7.40 minutes (*N*-benzylpropan-2-amine). The data were in agreement with those reported in the literature.[14]

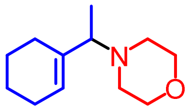

4-(1-(cyclohex-1-en-1-yl)ethyl)morpholine (1l): Following the general procedure, a J. Youngs NMR tube was loaded with triphenylborane (5.8 mg, 0.024 mmol, 0.10 eq.) in acetonitrile (0.5 mL), followed by the addition of 1-acetyl-1-cyclohexene (32 µL, 0.241 mmol, 1.00 eq.), morpholine (26 µL, 0.289 mmol, 1.20 eq.) and dimethylphenylsilane (132 µL, 0.845 mmol, 3.50 eq.). After heating at 100°C for 25 hours, in the ^{1}H NMR spectrum the signals of the proton attached to aminated carbons appeared at δ = 2.55 ppm (NMR yield = 87%, with anisole as standard). After concentration under reduced pressure, in the crude the main species detected were $(PhMe_2Si)_2O$ and 4-(1-(cyclohex-1-en-1-yl)ethyl)morpholine. ^{1}H NMR ($CDCl_3$, 400 MHz, 298 K): δ 5.66 (s, 1H, -C=C*H*), 3.79 (t, J = 4.8 Hz, 4H, -CH_2OCH_2-), 2.68 (q, J = 6.5 Hz, 1H, C*H*), 2.56-2.39 (m, 4H, -CH_2N(R)CH_2-), 2.18-2.03 (m, 4H), 1.81-1.58 (m, 4H), 1.22 (d, J = 6.5 Hz, 3H, -CH_3) ppm. ^{13}C{^{1}H} NMR ($CDCl_3$, 100 MHz, 298 K): 67.3 (2C, *C*-O) 67.2 (1C, -C*H*Me), 51.2 (broad, 2C, *C*-N), 25.1 (1C), 24.0 (1C), 22.8 (1C), 22.8 (1C), 16.5 (1C, -*C*H$_3$) ppm. GC-MS: *m/z* calculated for $C_{12}H_{21}NO$, 195.2; found 195.2. GC-MS retention times of analyte: 9.23 minutes (4-(1-(cyclohex-1-en-1-yl)ethyl)morpholine). The data were in agreement with those reported in the literature.[15]

13. Amine Substrate Scope

General Procedure: All reagents/solvent are used without any purification. A J. Youngs NMR tube equipped with a DMSO-d_6 capillary was loaded with triphenylborane (0.10 eq.) in acetonitrile (0.5 mL). Then, benzaldehyde (1.00 eq.), the appropriate amine (1.20 eq.) and dimethylphenylsilane (3.50 eq.) were added. After the monitoring of the initial reaction mixture by multinuclear NMR spectroscopy, the J. Youngs NMR tube was heated up at 100°C for 25 hours (unless otherwise indicated) and then monitored by ^{1}H, ^{11}B and ^{29}Si-NMR spectroscopy. Subsequent addition of mesitylene (25 μL) to the reaction mixture allowed the determination of the NMR yields based on the protons attached to the α-carbons. The reaction mixture was then concentrated under reduced pressure and the crude analysed by multinuclear NMR spectroscopy and by GC-MS analysis. Both analysis revealed the main present species were the $PhMe_2SiOH$ (^{29}Si NMR, δ ~ 3.5 ppm), $(PhMe_2Si)_2O$ (^{29}Si NMR δ ~ -1.0 ppm) and the desired product.

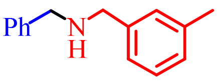

***N*-benzyl-1-(m-tolyl)methanamine (1m):** Following the general procedure, a J. Youngs NMR tube was loaded with triphenylborane (5.8 mg, 0.024 mmol, 0.10 eq.) in acetonitrile (0.5 mL), followed by the addition of benzaldehyde (25 μL, 0.241 mmol, 1.00 eq.), 3-methylbenzylamine (37 μL, 0.289 mmol, 1.20 eq.) and dimethylphenylsilane (132 μL, 0.843 mmol, 3.50 eq.). After heating at 100°C for 40 hours, in the ^{1}H NMR spectrum the signals of the protons attached to α-carbons appeared at δ = 3.76 and 3.72 ppm (NMR yield = 70%). After concentration under reduced pressure, in the crude the main species detected were $(PhMe_2Si)_2O$ and *N*-benzyl-1-(m-tolyl)methanamine. ^{1}H NMR ($CDCl_3$, 400 MHz, 298 K): δ 3.96 (s, 2H, -NCH_2), 3.93 (s, 2H, -NCH_2), 2.50 (s, 3H, CH_3) ppm. ^{13}C{^{1}H} NMR ($CDCl_3$, 100 MHz, 298 K): 53.0 (1C, NCH_2), 52.9 (1C, NCH_2), 21.3 (1C, CH_3) ppm. GC-MS: *m/z* calculated for $C_{15}H_{17}N$, 211.1; found 211.1. GC-MS retention times of analyte: 10.83 minutes (*N*-benzyl-1-(m-tolyl)methanamine). The data were in agreement with those reported in the literature.[16]

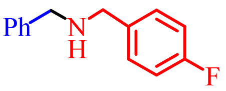

***N*-benzyl-1-(4-fluorophenyl)methanamine (1n):** Following the general procedure, a J. Youngs NMR tube was loaded with triphenylborane (5.8 mg, 0.024 mmol, 0.10 eq.) in acetonitrile (0.5 mL), followed by the addition of benzaldehyde (25 μL, 0.241 mmol, 1.00 eq.), 4-fluorobenzylamine (34 μL, 0.289 mmol, 1.20 eq.) and dimethylphenylsilane (132 μL, 0.843 mmol, 3.50 eq.). After heating at 100°C for 25 hours, in the ^{1}H NMR spectrum the signals of the protons attached to α-carbons appeared at δ = 3.75 and 3.73 ppm (NMR yield = 78%). After concentration under reduced pressure, in the crude the main species detected were $(PhMe_2Si)_2O$ and *N*-benzyl-1-(4-fluorophenyl)methanamine. ^{1}H NMR ($CDCl_3$, 400 MHz, 298 K): δ 3.93 (s, 2H, -NCH_2), 3.90 (s, 2H, -NCH_2) ppm. ^{13}C{^{1}H} NMR ($CDCl_3$, 100 MHz, 298 K): 52.9 (1C, NCH_2), 52.1 (1C, NCH_2) ppm. ^{19}F{^{1}H} NMR ($CDCl_3$, 100 MHz, 298 K): 115.9 ppm. GC-MS: *m/z* calculated for $C_{14}H_{14}NF$, 215.1; found 215.1. GC-MS retention times of analyte: 10.43 minutes (*N*-benzyl-1-(4-fluorophenyl)methanamine). The data were in agreement with those reported in the literature.[9]

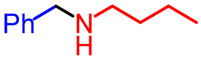

***N*-benzylbutan-1-amine (1o):** Following the general procedure, a J. Youngs NMR tube was loaded with triphenylborane (5.8 mg, 0.024 mmol, 0.10 eq.) in acetonitrile (0.5 mL), followed by the addition of benzaldehyde (25 µL, 0.241 mmol, 1.00 eq.), *n*-butylamine (29 µL, 0.289 mmol, 1.20 eq.) and dimethylphenylsilane (132 µL, 0.843 mmol, 3.50 eq.). After heating at 100°C for 30 hours, in the ^{1}H NMR spectrum the signals of the protons attached to α-carbons appeared at δ = 3.75 and 2.57 ppm (NMR yield = 45%). The other major products observed were derived from overalkylation or enamine isomerization. After concentration under reduced pressure, in the crude the main species detected were $(PhMe_2Si)_2O$ and *N*-benzylbutan-1-amine. ^{1}H NMR ($CDCl_3$, 400 MHz, 298 K): δ 3.91 (s, 2H, C*H_2*), 2.75 (t, *J* = 7.5 Hz, 2H, C*H_2*) ppm. $^{13}C\{^1H\}$ NMR ($CDCl_3$, 100 MHz, 298 K): 53.7 (1C, -N*C*H_2Ph), 48.8 (1C, -*C*H_2), 51.9 (1C, -N*C*$H_2$$CH_2$) ppm. GC-MS: *m/z* calculated for $C_{11}H_{17}N$, 163.1; found 163.1. GC-MS retention times of analyte: 8.36 minutes (*N*-benzylbutan-1-amine). The data were in agreement with those reported in the literature.[17]

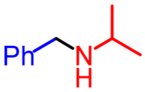

***N*-benzylpropan-2-amine (1p):** Following the general procedure, a J. Youngs NMR tube was loaded with triphenylborane (5.8 mg, 0.024 mmol, 0.10 eq.) in acetonitrile (0.5 mL), followed by the addition of benzaldehyde (25 µL, 0.241 mmol, 1.00 eq.), isopropylamine (25 µL, 0.289 mmol, 1.20 eq.) and dimethylphenylsilane (132 µL, 0.843 mmol, 3.50 eq.). After heating at 100°C for 25 hours, in the ^{1}H NMR spectrum the signals of the protons attached to α-carbons appeared at δ = 3.76 and 2.80 ppm (NMR yield = 78%). After concentration under reduced pressure, in the crude the main species detected were $(PhMe_2Si)_2O$ and *N*-benzyl-2-methylpropan-2-amine. ^{1}H NMR ($CDCl_3$, 400 MHz, 298 K): δ 3.78 (s, 2H, C*H_2*), 2.86 (sept, *J* = 6.3 Hz, 1H, C*H*), 1.11 (d, *J* = 6.3 Hz, 6H, C*H_3*) ppm. $^{13}C\{^1H\}$ NMR ($CDCl_3$, 100 MHz, 298 K): 51.1 (1C, *C*H_2), 48.0 (1C, *C*H), 22.4 (2C, *C*H_3) ppm. GC-MS: *m/z* calculated for $C_{10}H_{15}N$, 149.1; found 149.1. GC-MS retention times of analyte: 7.40 minutes (*N*-benzylpropan-2-amine). The data were in agreement with those reported in the literature.[14]

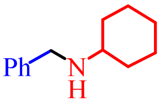

***N*-benzylcyclohexanamine (1q):** Following the general procedure, a J. Youngs NMR tube was loaded with triphenylborane (5.8 mg, 0.024 mmol, 0.10 eq.) in acetonitrile (0.5 mL), followed by the addition of benzaldehyde (25 µL, 0.241 mmol, 1.00 eq.), cyclohexylamine (34 µL, 0.289 mmol, 1.20 eq.) and dimethylphenylsilane (132 µL, 0.843 mmol, 3.50 eq.). After heating at 100°C for 25 hours, in the ^{1}H NMR spectrum the signals of the protons attached to α-carbons appeared at δ = 3.79 and 2.45 ppm (NMR yield = 84%). After concentration under reduced pressure, in the crude the main species detected were $(PhMe_2Si)_2O$ and *N*-benzylcyclohexanamine. ^{1}H NMR ($CDCl_3$, 400 MHz, 298 K): δ 3.73 (s, 2H, -NC*H_2*), 2.47-2.36 (m, 1H) ppm. $^{13}C\{^1H\}$ NMR ($CDCl_3$, 100 MHz, 298 K): 56.0 (1C, N*C*H_2), 50.7 (1C, N*C*H) ppm. GC-MS: *m/z* calculated for $C_{13}H_{19}N$, 189.1; found 189.1. GC-MS retention times of analyte: 9.73 minutes (*N*-benzylcyclohexanamine). The data were in agreement with those reported in the literature.[18]

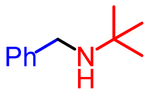

***N*-benzyl-2-methylpropan-2-amine (1r):** Following the general procedure, a J. Youngs NMR tube was loaded with triphenylborane (5.8 mg, 0.024 mmol, 0.10 eq.) in acetonitrile (0.5 mL), followed by the addition of benzaldehyde (25 µL, 0.241 mmol, 1.00 eq.), *t*-butylamine (31 µL, 0.289 mmol, 1.20 eq.) and dimethylphenylsilane (132 µL, 0.843 mmol, 3.50 eq.). After heating at 100°C for 25 hours, in the ^{1}H NMR spectrum the signal of the protons attached to α-carbon appeared at δ = 3.74 ppm (NMR yield = 93%). After concentration under reduced pressure, in the crude the main species detected were $(PhMe_2Si)_2O$ and *N*-benzyl-2-methylpropan-2-amine. ^{1}H NMR ($CDCl_3$, 400 MHz, 298 K): δ 3.88 (s, 2H, C*H*$_2$), 1.33 (s, 9H, *tBu*) ppm. ^{13}C{^{1}H} NMR ($CDCl_3$, 100 MHz, 298 K): 50.8 (1C, *C*H$_2$), 47.1 (1C, -*C*(CH_3)$_3$), 28.9 (3C, -*C*H$_3$)ppm. GC-MS: *m/z* calculated for $C_{11}H_{17}N$, 163.1; found 163.1. GC-MS retention times of analyte: 7.77 minutes (*N*-benzyl-2-methylpropan-2-amine). The data were in agreement with those reported in the literature.[17]

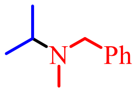

***N*-benzyl-*N*-methylpropan-2-amine (1s):** Following the general procedure, a J. Youngs NMR tube was loaded with triphenylborane (6.9 mg, 0.027 mmol, 0.10 eq.) in acetonitrile (0.5 mL), followed by the addition of acetone (20 µL, 0.270 mmol, 1.00 eq.), *N*-methylbenzylamine (43 µL, 0.324 mmol, 1.20 eq.) and dimethylphenylsilane (148 µL, 0.944 mmol, 3.50 eq.). After heating at 100°C for 25 hours, in the ^{1}H NMR spectrum the signals of the protons attached to α-carbons appeared at δ = 3.52 and 2.87 ppm (NMR yield = 98%). After concentration under reduced pressure, in the crude the main species detected were $(PhMe_2Si)_2O$ and *N*-benzyl-*N*-methylpropan-2-amine. ^{1}H NMR ($CDCl_3$, 400 MHz, 298 K): δ 3.42 (s, 2H, -NC*H*$_2$), 2.81 (sept, *J* = 6.5 Hz, 1H, -NC*H*-), 2.06 (s, 3H, N*Me*), 1.00 (d, *J* = 6.5 Hz, 6H, C(C*H*$_3$)$_2$) ppm. ^{13}C{^{1}H} NMR ($CDCl_3$, 100 MHz, 298 K): 57.5 + 52.9 (2C, *C*H$_2$N*C*H), 36.7 (1C, N*C*H$_3$), 17.8 (2C, C(*C*H$_3$)$_2$) ppm. GC-MS: *m/z* calculated for $C_{11}H_{17}N$, 163.1; found 163.1. GC-MS retention times of analyte: 7.69 minutes (*N*-benzyl-*N*-methylpropan-2-amine). The data were in agreement with those reported in the literature.[19]

14. Synthesis of *N*-benzyl-1-adamantaylamine on gram-scale

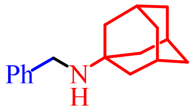

Control experiment in small scale: Following the general procedure, a J. Youngs NMR tube was loaded with triphenylborane (5.8 mg, 0.024 mmol, 0.10 eq.) in acetonitrile (0.5 mL), followed by the addition of benzaldehyde (25 μL, 0.241 mmol, 1.00 eq.), 1-adamantylamine (45 mg, 0.289 mmol, 1.20 eq.) and dimethylphenylsilane (132 μL, 0.843 mmol, 3.50 eq.). After heating at 100°C for 25 hours, in the ^{1}H NMR spectrum the signal of the protons attached to α-carbon appeared at δ = 3.73 ppm (NMR yield = 96%). After concentration under reduced pressure, in the crude the main species detected were $(PhMe_2Si)_2O$ and *N*-benzyl-1-adamantylamine. ^{1}H NMR ($CDCl_3$, 400 MHz, 298 K): δ 3.77 (s, 2H, -NC*H*$_2$), 2.14-2.06 (m, 3H, -$(CH_2)_3$C*H*) ppm. ^{13}C{^{1}H} NMR ($CDCl_3$, 100 MHz, 298 K): 50.8 (1C, N*C*H_2), 45.0 (1C, N*C*$(CH_2)_3$), 42.8 (3C, *C*H_2), 36.7 (3C, *C*H_2), 29.6 (3C, *C*H) ppm. GC-MS: *m/z* calculated for $C_{17}H_{23}N$, 241.2; found 241.2. GC-MS retention times of analyte: 11.80 minutes (*N*-benzyl-1-adamantylamine). The data were in agreement with those reported in the literature.[20]

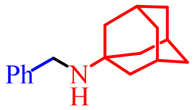

Gram-scale synthesis (performed under ambient atmosphere with no purification of reagents or solvent): A 2-necked round bottomed flask was loaded with triphenylborane (122 mg, 0.5 mmol, 0.10 eq.) in acetonitrile (10 mL), followed by the addition of benzaldehyde (520 μL, 5.0 mmol, 1.00 eq.), 1-adamantylamine (938 mg, 6.0 mmol, 1.20 eq.) and dimethylphenylsilane (2.7 mL, 17.5 mmol, 3.50 eq.). The flask was then equipped with a condenser and heated at 100°C for 25 hours (MeCN reflux). The reaction mixture was cooled down to r.t. and then diluted in more MeCN (10 ml), observing a white solid precipitate. After filtration, the clear orange solution left was dried and purified by flash-chromatography (Pet. Et. : EtOAc 9:1) obtaining *N*-benzyl-1-adamantylamine as yellow oil (1.1 g, 4.556 mmol, 90%). R_f = 0.23. ^{1}H NMR ($CDCl_3$, 400 MHz, 298 K): δ 7.38-7.28 (m, 4H), 7.23 (t, *J* = 6.8 Hz, 1H), 3.77 (s, 2H), 2.14-2.06 (m, 3H), 1.75-1.61 (m, 12H), 1.23-0.67 (brs, 1H, N*H*) ppm. ^{13}C{^{1}H} NMR ($CDCl_3$, 100 MHz, 298 K): 141.8, 128.4, 128.2, 126.6, 50.8, 45.1, 42.9, 36.8, 29.6. The data were in agreement with those reported in the literature.[20]

15. Reductive amination benzaldehyde/benzhydrylamine with $B(C_6F_5)_3$ and BPh_3

$B(C_6F_5)_3$ as catalyst: Following the general procedure, a J. Youngs NMR tube was loaded with $B(C_6F_5)_3$ (12.3 mg, 0.024 mmol, 0.10 eq.) in acetonitrile (0.5 mL), followed by the addition of benzaldehyde (25 µL, 0.241 mmol, 1.00 eq.), benzhydrylamine (52 µL, 0.289 mmol, 1.20 eq.) and dimethylphenylsilane (132 µL, 0.843 mmol, 3.50 eq.). After heating at 100°C for 25 hour, low imine reduction was observed (NMR conversion = 30%).

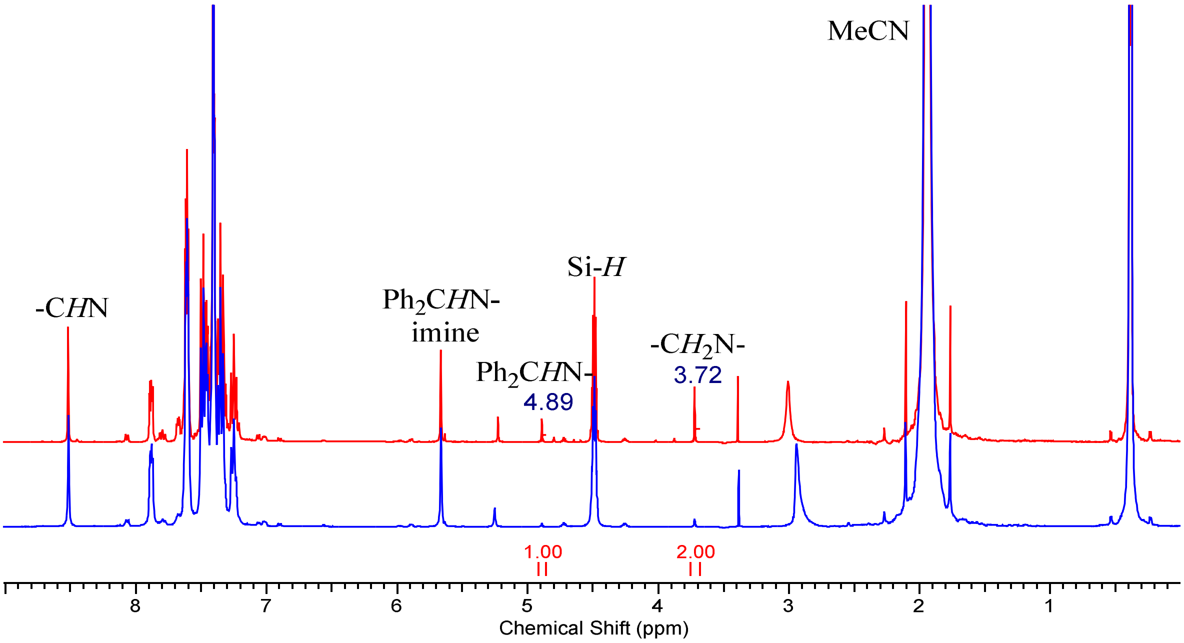

Figure S54. *In situ* ^{1}H-NMR spectra of the reductive amination of benzaldehyde/ benzhydrylamine with $B(C_6F_5)_3$ in acetonitrile, using $PhMe_2SiH$ (3.50 eq.) as reductant. Blue (t = 5 min, r.t.), red (after 25 hour at 100°C).

BPh_3 as catalyst: Following the general procedure, a J. Youngs NMR tube was loaded with BPh_3 (5.8 mg, 0.024 mmol, 0.05 eq.) in acetonitrile (0.5 mL), followed by the addition of benzaldehyde (25 µL, 0.241 mmol, 1.00 eq.), benzhydrylamine (52 µL, 0.289 mmol, 1.20 eq.) and dimethylphenylsilane (132 µL, 0.843 mmol, 3.50 eq.). After heating at 100°C for 25 hour, low imine reduction was observed (NMR conversion = 30%).

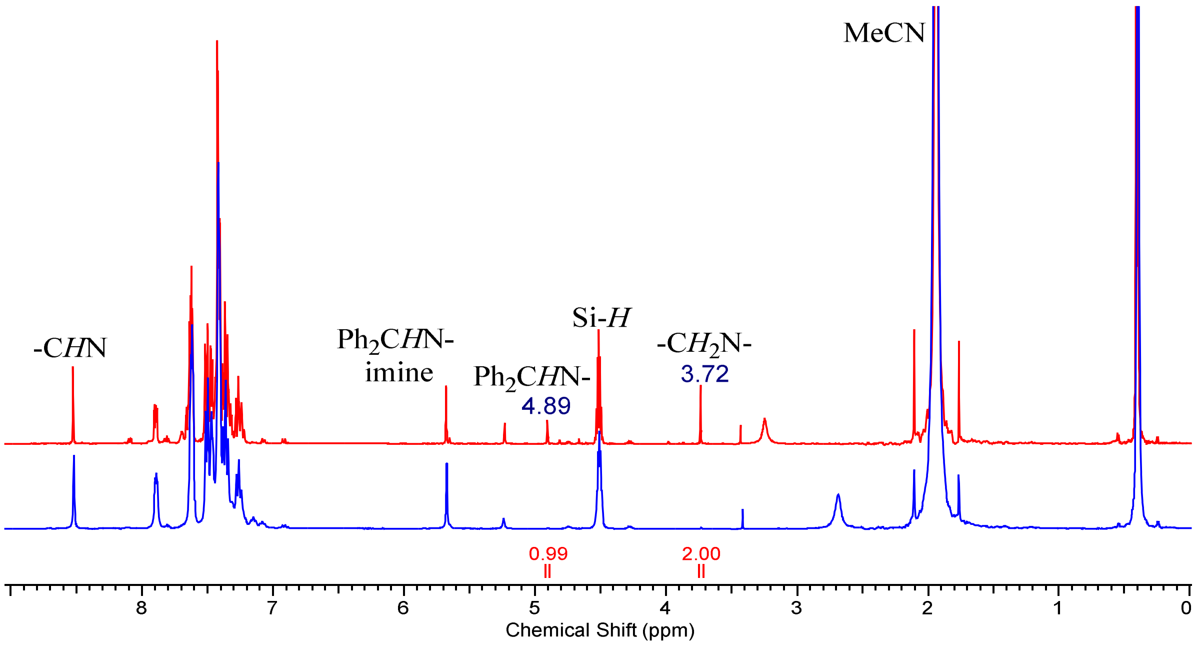

Figure S55. *In situ* ^{1}H-NMR spectra of the reductive amination of benzaldehyde/ benzhydrylamine with BPh_3 in acetonitrile, using $PhMe_2SiH$ (3.50 eq.) as reductant. Blue (t = 5 min, r.t.), red (after 25 hour at 100°C).

16. Reductive aminations using NaBArCl as catalyst precursor

Acid induced degradation of NaBArCl: A J. Youngs NMR tube was loaded with sodium tetra(3,5-dichlorophenyl)borate (45.6 mg, 0.072 mmol, 0.30 eq.) in dichloromethane (0.5 mL) in which it is only partially soluble at this concentration, followed by the addition of trifluoromethanesulfonimide (7.1 mg, 0.024 mmol, 0.10 eq.). After few seconds from the addition, most of the insoluble white solid disappeared, obtaining a yellow heterogeneous solution. The ^{11}B-NMR spectrum of the reaction mixture revealed the decomposition of the BArCl anion with the concomitant appearance of a new peaks (the peak at 66.8 ppm is consistent with (3,5-$C_6H_3Cl_2$)$_3$B). The degradation was performed in a non-coordinating solvent using a weakly coordinating anion (Tf_2N^-) in order to clearly detect the formation of borane. The impurities maybe caused by further decomposition to $Ar_{3-x}B(OH)_x$ (peaks at 43.9 and 28.2 ppm) by trace water or are possibly species such as $Aryl_2BNTf_2$ (which on exposure to moisture e.g., during catalysis will form the borinic acid anyway).

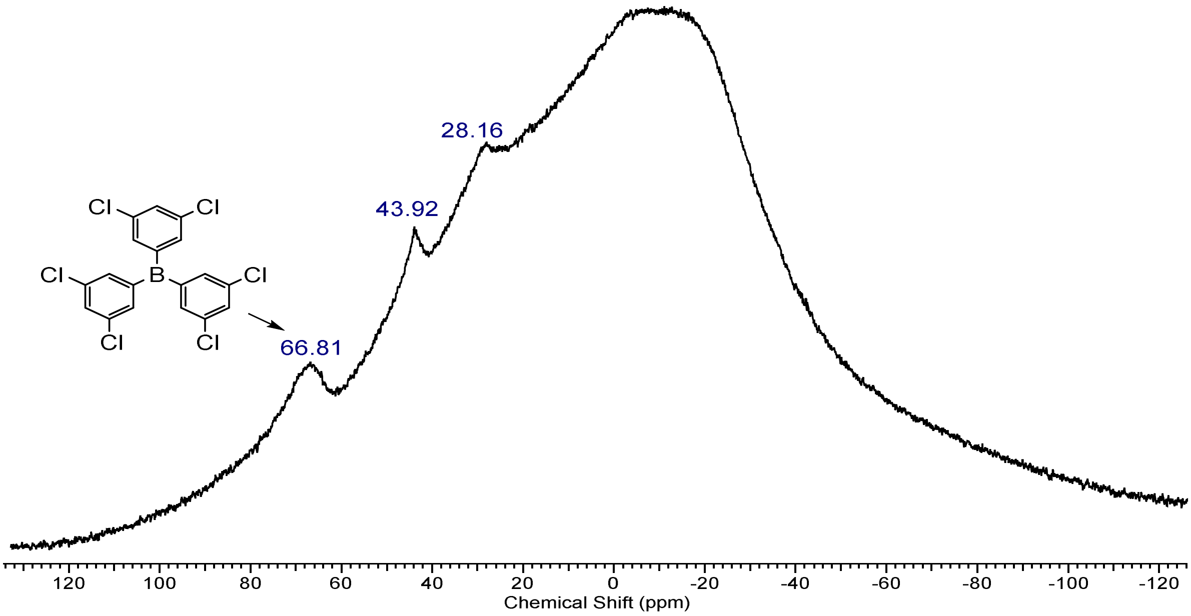

Figure S56. In situ ^{11}B-NMR spectrum of NaBArCl and Tf_2NH (3:1) in DCM (with capillary inserted containing d$_6$-DMSO).

The reaction mixture was then concentrated, obtaining a white solid which was dissolved in acetonitrile, followed by the addition of benzaldehyde (25 μL, 0.241 mmol, 1.00 eq.), benzhydrylamine (52 μL, 0.289 mmol, 1.20 eq.) and dimethylphenylsilane (132 μL, 0.843 mmol, 3.50 eq.). After heating at 100°C for 5 hours, in the ^{1}H NMR the signal of the protons attached to α-carbons appeared at δ = 4.88 and 3.71 ppm (NMR yield = 92%). After concentration under reduced pressure, in the crude the main species detected were $(PhMe_2Si)_2O$ and *N*-benzyl-1,1-diphenylmethanamine. ^{1}H NMR ($CDCl_3$, 400 MHz, 298 K): δ 5.06 (s, 1H, -NC*H*), 3.94 (s, 2H, -NC*H₂*) ppm. ^{13}C{^{1}H} NMR ($CDCl_3$, 100 MHz, 298 K): 66.4 (1C, N*C*H), 51.8 (1C, N*C*H$_2$) ppm. GC-MS: *m/z* calculated for $C_{20}H_{19}N$, 273.1; found 273.1. GC-MS retention times of analyte: 12.61 minutes (*N*-benzyl-1,1-diphenylmethanamine). The data were in agreement with those reported in the literature.[21]

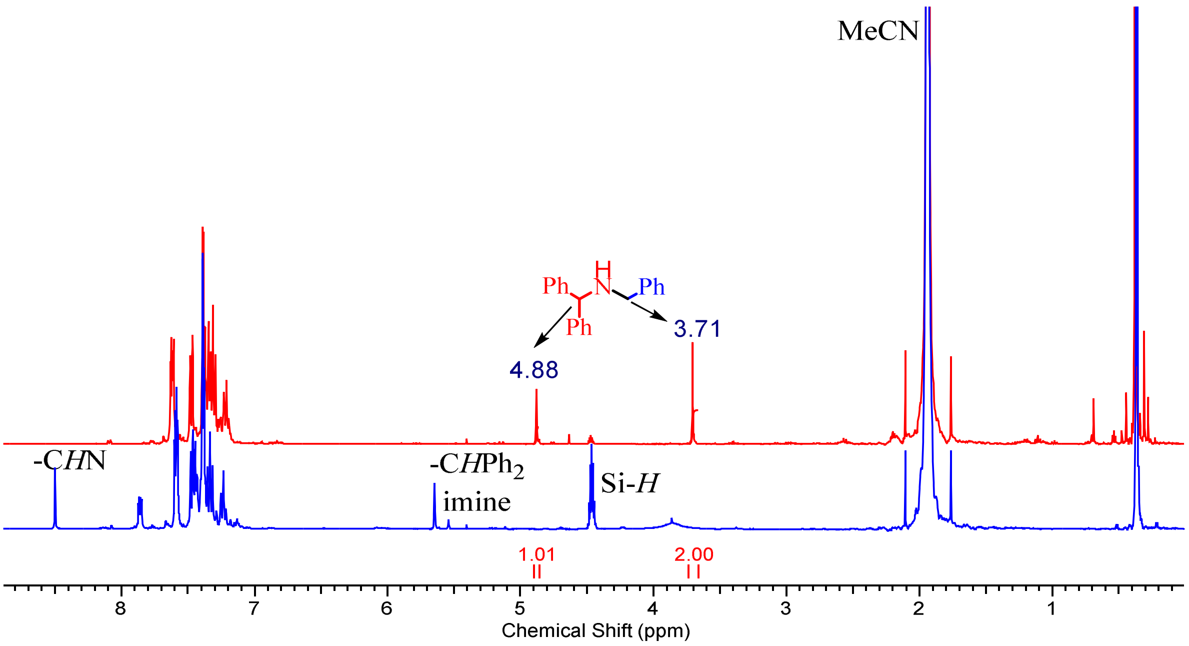

Figure S57. *In situ* ^{1}H-NMR spectra of the reductive amination of benzaldehyde/ benzhydrylamine with NaBArCl and Tf_2NH (3:1) in acetonitrile, using $PhMe_2SiH$ (3.50 eq.) as reductant. Blue (t = 5 min, r.t.), red (after 5 hours at 100°C).

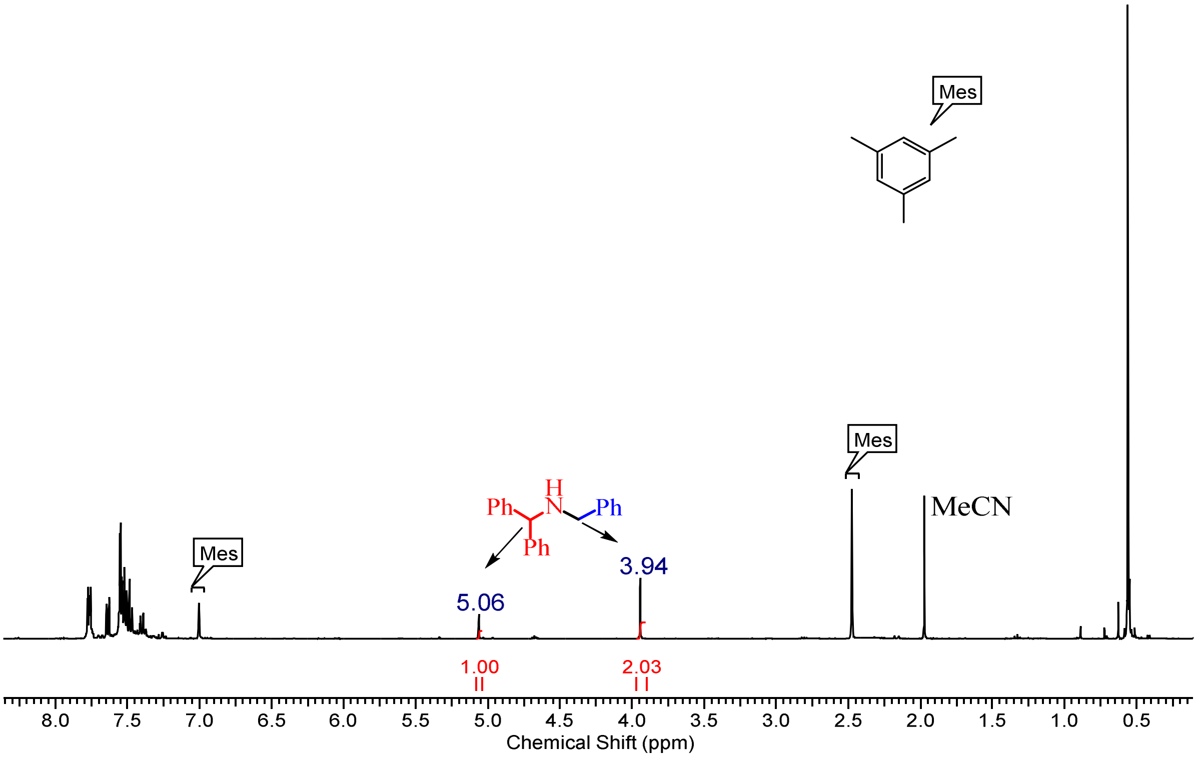

Figure S58. ^{1}H-NMR spectrum of the reaction crude.

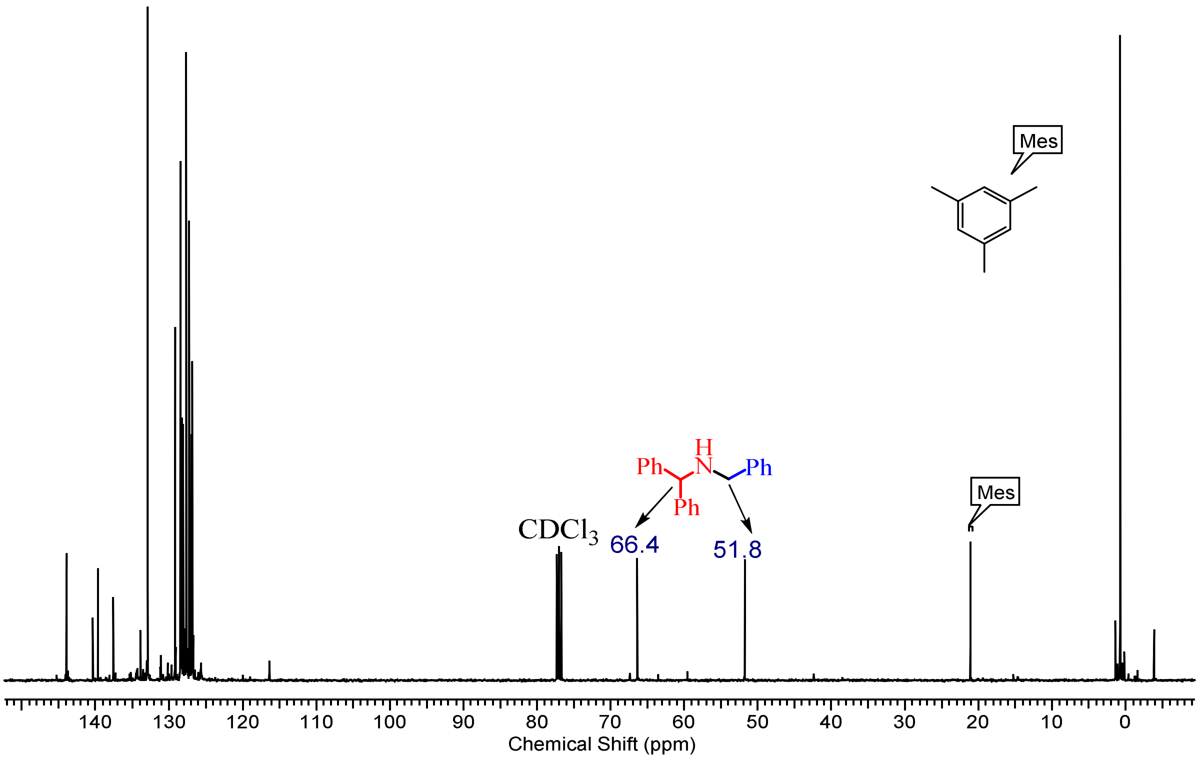

Figure S59. ^{13}C-NMR spectrum of the reaction crude.

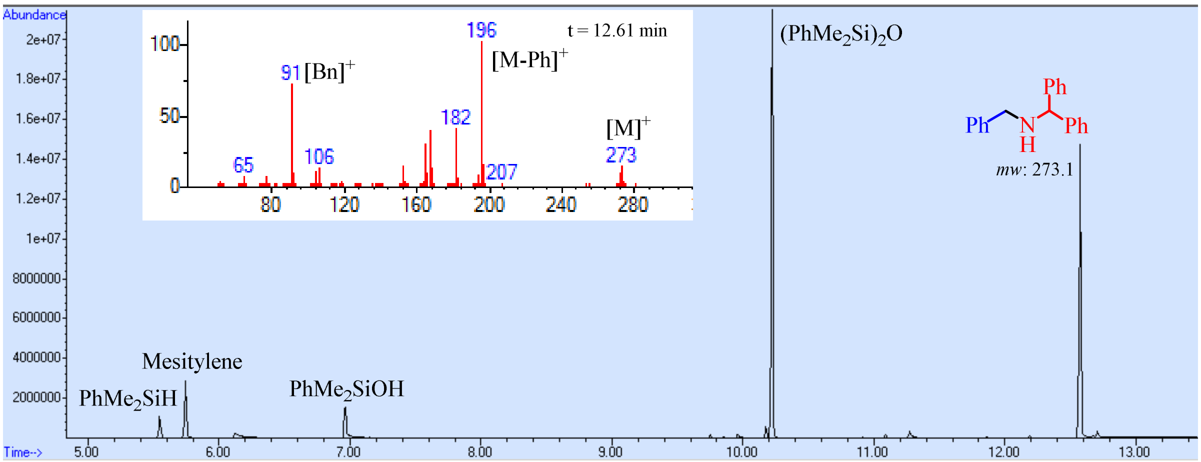

Figure S60. GC-MS of crude reaction.

Reductive amination of benzaldehyde/aniline using NaBArCl as catalyst precursor: A J. Youngs NMR tube was loaded with sodium tetra(3,5-dichlorophenyl)borate (15.2 mg, 0.024 mmol, 0.10 eq.) in o-dichlorobenzene (0.5 mL), followed by the addition of benzaldehyde (25 μL, 0.241 mmol, 1.00 eq.), aniline (26 μL, 0.289 mmol, 1.20 eq.) and dimethylphenylsilane (45 μL, 0.289 mmol, 1.20 eq.). After heating at 100°C for 1 hour, in the ^{1}H NMR spectrum the signal of the protons attached to α-carbon appeared at δ = 4.37 ppm (NMR yield = 88%). The NMR yield is an average of two independent reactions. The data were in agreement with those previously reported.

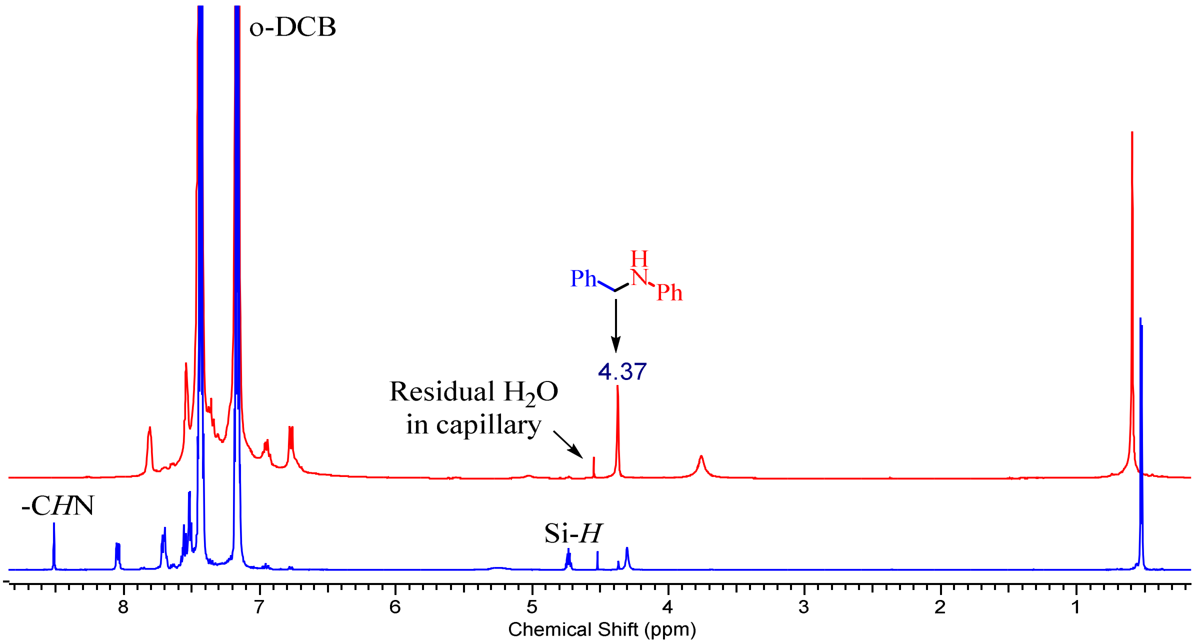

Figure S61. *In situ* ^{1}H-NMR spectra, with wet d_6-DMSO capillary inserted, of the reductive amination of benzaldehyde/aniline with NaBArCl in o-DCB, using $PhMe_2SiH$ (1.20 eq.) as reductant. Blue (t = 5 min, r.t.), red (after 1 hour at 100°C).

Reductive amination of benzaldehyde/benzhydrylamine using NaBArCl as catalyst precursor: A J. Youngs NMR tube was loaded with sodium tetra(3,5-dichlorophenyl)borate (15.2 mg, 0.024 mmol, 0.10 eq.) in o-dichlorobenzene (0.5 mL), followed by the addition of benzaldehyde (25 μL, 0.241 mmol, 1.00 eq.), benzhydrylamine (52 μL, 0.289 mmol, 1.20 eq.) and dimethylphenylsilane (83 μL, 0.530 mmol, 2.20 eq.). After heating at 100°C for 20 hours, in the ^{1}H NMR spectrum the signals of the protons attached to α-carbons appeared at δ = 5.08 and 3.93 ppm (NMR yield = 80%). The NMR yield is an average of two independent reactions. The data were in agreement with those previously reported.

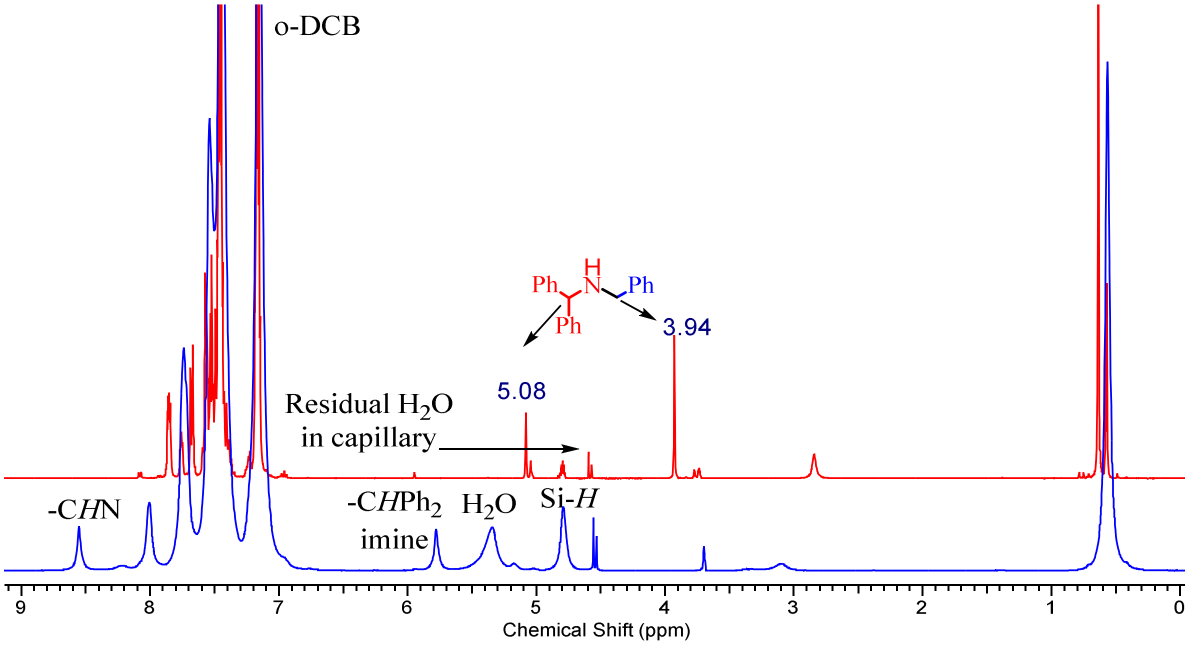

Figure S62. *In situ* ^{1}H-NMR spectra, with wet d_6-DMSO capillary inserted, of the reductive amination of benzaldehyde/benzhydrylamine with NaBArCl in o-DCB, using $PhMe_2SiH$ (2.20 eq.) as reductant. Blue (t = 5 min, r.t.), red (after 20 hours at 100°C).

Reductive amination of benzaldehyde/benzylamine using NaBArCl as catalyst precursor: A J. Youngs NMR tube was loaded with sodium tetra(3,5-dichlorophenyl)borate (15.2 mg, 0.024 mmol, 0.10 eq.) in o-dichlorobenzene (0.5 mL), followed by the addition of benzaldehyde (25 µL, 0.241 mmol, 1.00 eq.), benzylamine (32 µL, 0.289 mmol, 1.20 eq.) and dimethylphenylsilane (132 µL, 0.843 mmol, 3.50 eq.). After heating at 100°C for 25 hours, in the ^{1}H NMR spectrum the signals of the protons attached to α-carbons appeared at δ = 3.90 ppm (NMR yield = 60%). In this case, overalkylation was observed (Bn_3N: GC-MS: *m/z* calculated for $C_{21}H_{21}N$, 287.1; found 287.1. GC-MS retention times of analyte: 12.66 minutes). The NMR yield is an average of two independent reactions. The data were in agreement with those previously reported.

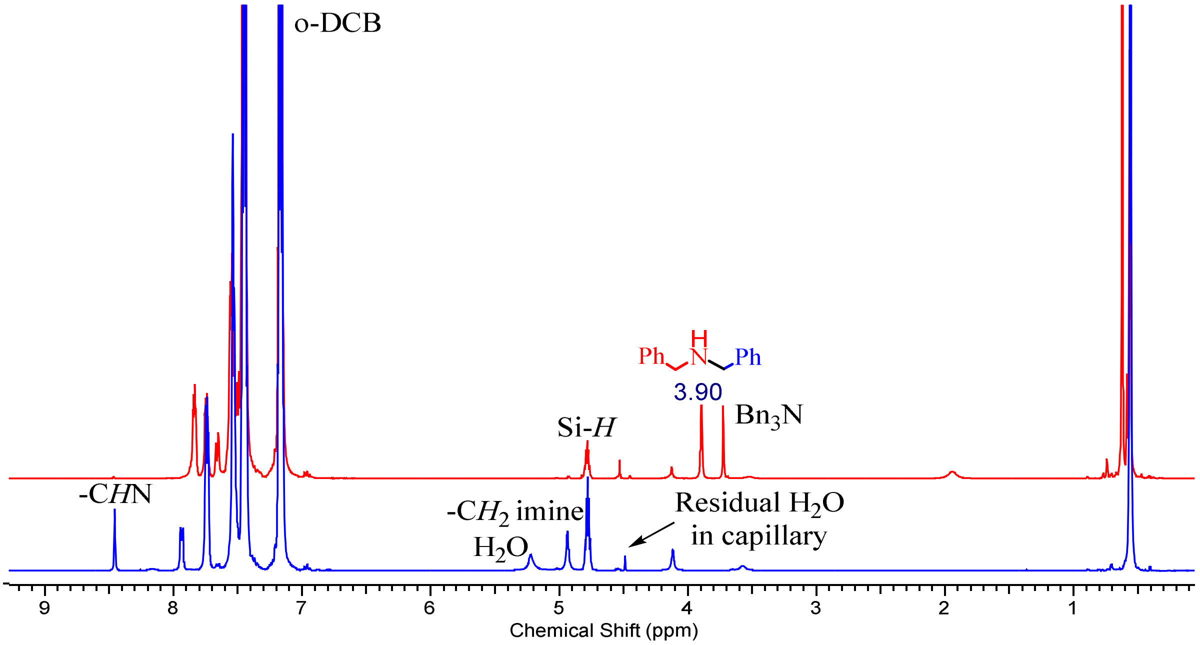

Figure S63. *In situ* ^{1}H-NMR spectra, with wet d_6-DMSO capillary inserted, of the reductive amination of benzaldehyde/benzylamine with NaBArCl in o-DCB, using $PhMe_2SiH$ (3.50 eq.) as reductant. Blue (t = 5 min, r.t.), red (after 25 hours at 100°C).

Reductive amination of benzaldehyde/t-butylamine using NaBArCl as catalyst precursor: A J. Youngs NMR tube was loaded with sodium tetra(3,5-dichlorophenyl)borate (15.2 mg, 0.024 mmol, 0.10 eq.) in o-dichlorobenzene (0.5 mL), followed by the addition of benzaldehyde (25 µL, 0.241 mmol, 1.00 eq.), *t*-butylamine (31 µL, 0.289 mmol, 1.20 eq.) and dimethylphenylsilane (132 µL, 0.843 mmol, 3.50 eq.). After heating at 100°C for 25 hours, in the ^{1}H NMR spectrum the signals of the protons attached to α-carbons appeared at δ = 3.87 ppm (NMR yield = 93%). The NMR yield is an average of two independent reactions. The data were in agreement with those previously reported.

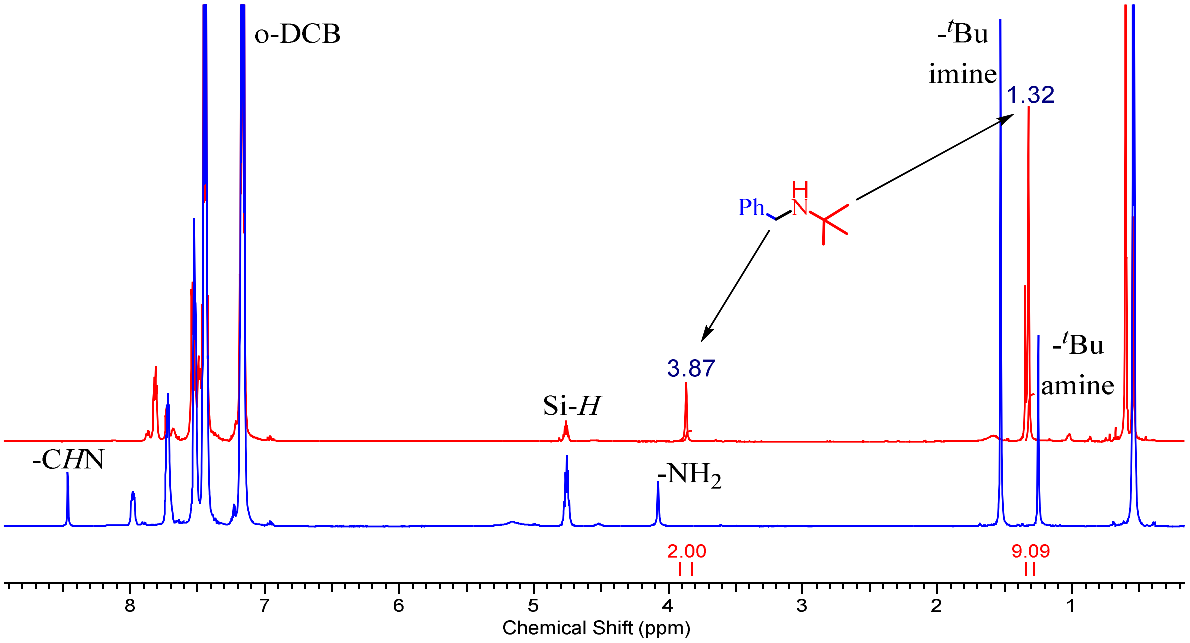

Figure S64. *In situ* ^{1}H-NMR spectra, with wet d_6-DMSO capillary inserted, of the reductive amination of benzaldehyde/ *t*-butylamine with NaBArCl in o-DCB, using $PhMe_2SiH$ (3.50 eq.) as reductant. Blue (t = 5 min, r.t.), red (after 25 hours at 100°C).

17. Synthesis of Piribedil

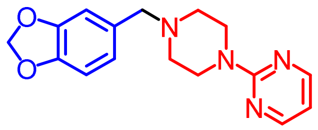

Reaction performed under ambient atmosphere with no purification of reagents or solvent. A 2-necked round bottomed flask was loaded with sodium tetra(3,5-dichlorophenyl)borate (390 mg, 0.6 mmol, 0.10 eq.) in o-dichlorobenzene (10 mL), followed by the addition of piperonal (910 mg, 6.0 mmol, 1.00 eq.), 2-(piperazin-1-yl)pyrimidine (1.0 mL, 7.2 mmol, 1.20 eq.) and dimethylphenylsilane (3.3 mL, 21.0 mmol, 3.50 eq.). The flask was then equipped with a condenser and heated at 100°C for 25 hours. The reaction mixture was cooled down to r.t. and then purified by flash-chromatography (Pet. Et. : EtOAc 1:0 to 2:1) obtaining *Piribedil* as yellow solid (1.3 g, 4.4 mmol, 74%). R_f = 0.20. ^{1}H NMR ($CDCl_3$, 400 MHz, 298 K): δ 8.30 (d, J = 4.8 Hz, 2H), 6.89 (s, 1H), 6.76 (s, 2H), 6.46 (t, J = 4.8 Hz, 1H), 5.95 (s, 2H), 3.82 (t, J = 5.3 Hz, 4H), 3.45 (s, 2H), 2.48 (t, J = 5.3 Hz, 4H) ppm. ^{13}C{^{1}H} NMR ($CDCl_3$, 100 MHz, 298 K): 161.6, 157.6, 147.6, 146.6, 131.8, 122.2, 109.7, 109.4, 107.8, 100.9, 62.8, 52.8, 43.6 ppm. GC-MS: m/z calculated for C16H18N4O2, 298.1; found 298.1. GC-MS retention times of analyte: 13.77 minutes. The data were in agreement with those reported in the literature.[22]

18. BPh_3 decomposition sealed under air

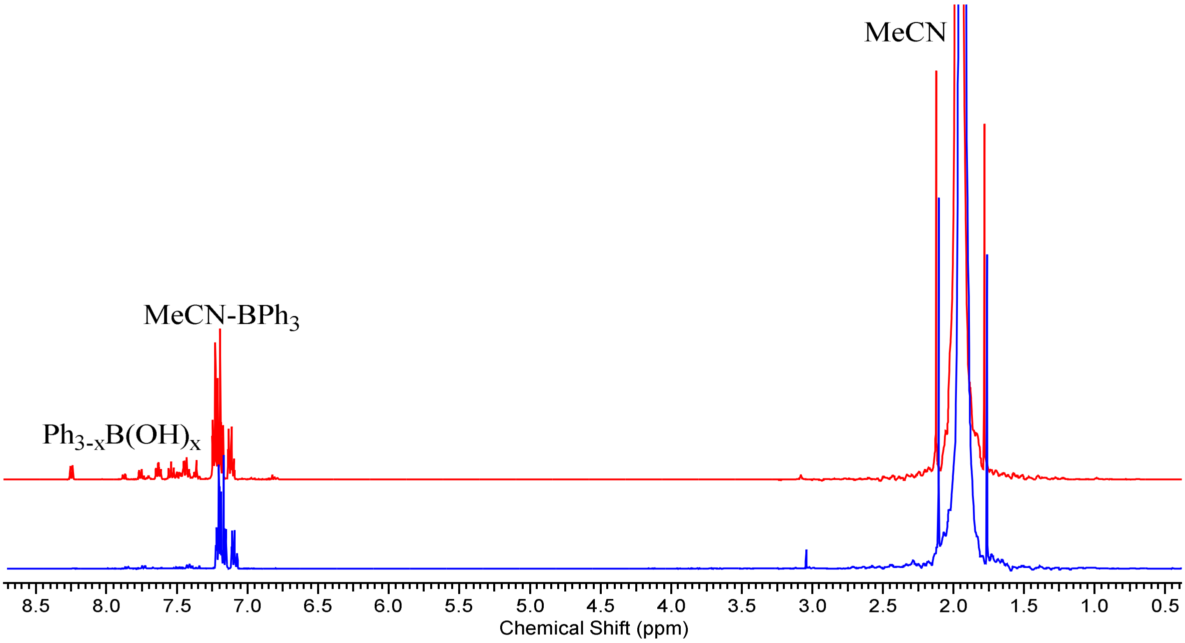

Figure S65. ^{1}H-NMR spectra of BPh_3 in distilled acetonitrile (with d_6-DMSO capillary inserted). Blue (as received), red (kept under ambient atmosphere as a solid for 15 days).

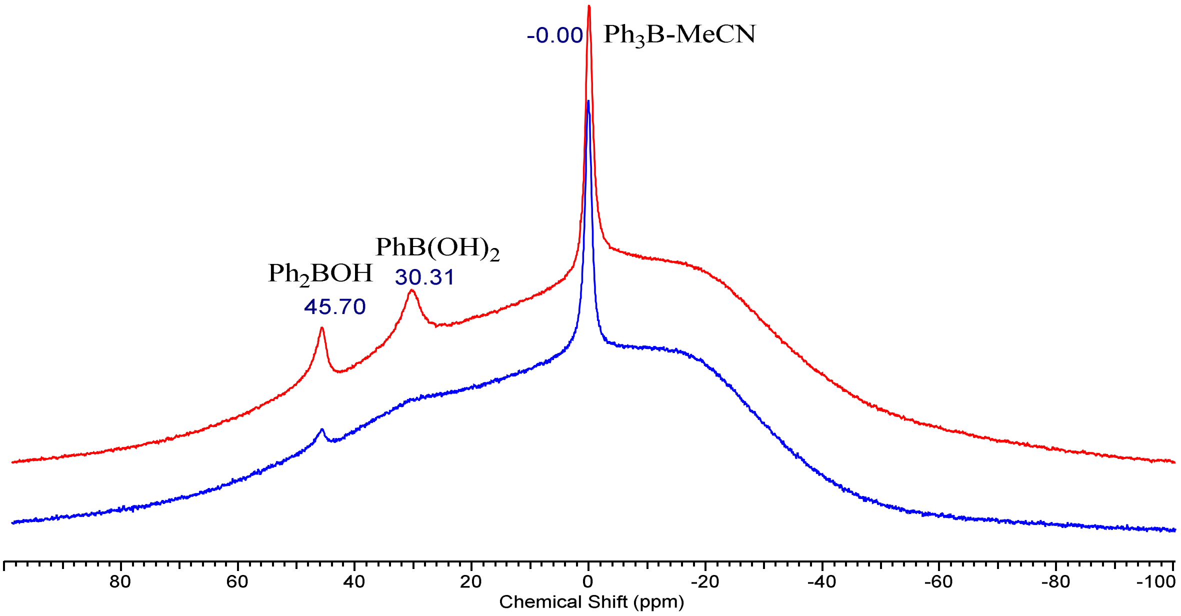

Figure S66. ^{11}B-NMR spectra of BPh_3 in distilled acetonitrile (with d_6-DMSO capillary inserted). Blue (as received), red (kept under ambient atmosphere as a solid for 15 days).

19. References

1 R. Anulewicz-Ostrowska, T. Kliś, D. Krajewski, Ba. Lewandowski and J. Serwatowski, *Tetrahedron Letters*, **2003**, *44*, 7329–7331.

2 D. Mukherjee, H. Osseili, T. P. Spaniol and J. Okuda, *J. Am. Chem. Soc.*, **2016**, *138*, 10790-10793.

3 K. G. Andrews, D. M. Summers, L. J. Donnelly and R. M. Denton, *Chem. Commun.*, **2016**, *52*, 1855-1858.

4 Y. Minghua and F. Liu, *J. Org. Chem.*, **2007**, *72*, 8969-8971.

5 E. R. Clark and M. J. Ingleson, *Angew. Chem. Int. Ed.*, **2014**, *53*, 11306 –11309.

6 P. R. Likhar,R. Arundhathi,M. L. Kantam and P. S. Prathima, *Eur. J. Org. Chem.*, **2009**, 5383–5389.

7 S. Zhang, Y. Wang, X. Feng and M.Bao, *J. Am. Chem. Soc.*, **2012**, *134*, 5492-5495.

8 C. J. Smith, C. D. Smith, N. Nikbin, S. V. Ley and I. R. Baxendale, *Org. Biomol. Chem.*, **2011**, *9*, 1927-1937.

9 S. Das, B. Join, K. Junge and M. Beller, *Chem. Commun.*, **2012**, *48*, 2683-2685.

10 P. V. Ramachandran, P. D. Gagare, Ka. Sakavuyi and P. Clark, *Tetrahedron Letters*, **2010**, *51*, 3167–3169.

11 M. Lohse, K. Nowosinski, N. L. Traulsen, A. J. Achazi, L. K. S. von Krbek, B. Paulus, C. A. Schalley and S. Hecht, *Chem. Commun.*, **2015**, *51*, 9777-9780.

12 X. Wang, V. Ervithayasuporn, Y. Zhang and Y. Kawakami, *Chem. Commun.*, **2011**, *47*, 1282-1284.

13 C. Wang, A. Pettman, J. Bacsa and J. Xiao, *Angew. Chem. Int. Ed.*, **2010**, *49*, 7548-7552.

14 L. M. Martínez-Prieto, A. Ferry, L. Rakers, C. Richter, P. Lecante, K. Philippot, B. Chaudret and F. Glorius, *Chem. Commun.*, **2016**, *52*, 4768-4771.

15 A. F. Abdel-Magid, K. G. Carson, B. D. Harris, C. A. Maryanoff and R. D. Shah, *J. Org. Chem.*, **1996**, *61*, 3849–3862.

16 W. Chen, J. Ren, M. Wang, L. Dang, X. Shen, X. Yang and H. Zhang, *Chem. Commun.*, **2014**, *50*, 6259-6262.

17 P. Huang, Q. Lang and Y. Wang, *J. Org. Chem.*, **2016** , *81*, 4235 – 4243.

18 Q. Lei, Y. Wei, D. Talwar, C. Wang, D. Xue and J. Xiao, *Chem. Eur. J.*, **2013**, *19*, 4021-4029.

19 H. Ahlbrecht, J. Harbagh, R. W. Hoffmann and T. Ruhland, *Liebigs Ann*, **1995**, 211–216.

20 S. Bähn, D. Hollmann, A. Tillack, M. Beller, *Adv. Synth. Catal.*, **2008**, *350*, 2099-2103.

21 I. Chatterjee and M. Oestreich, *Angew. Chem. Int. Ed.*, **2015**, *54*, 1965 –1968.

22 M.-C. Fu, R. Shang, W.-M. Cheng and Y. Fu, *Angew. Chem. Int. Ed.*, **2015**, *54*, 9042–9046.

20. NMR and GC-MS Data for All Compounds

N-benzyl-1-(2-chlorophenyl)methanamine (1a)

In situ – ^{1}H-NMR spectra

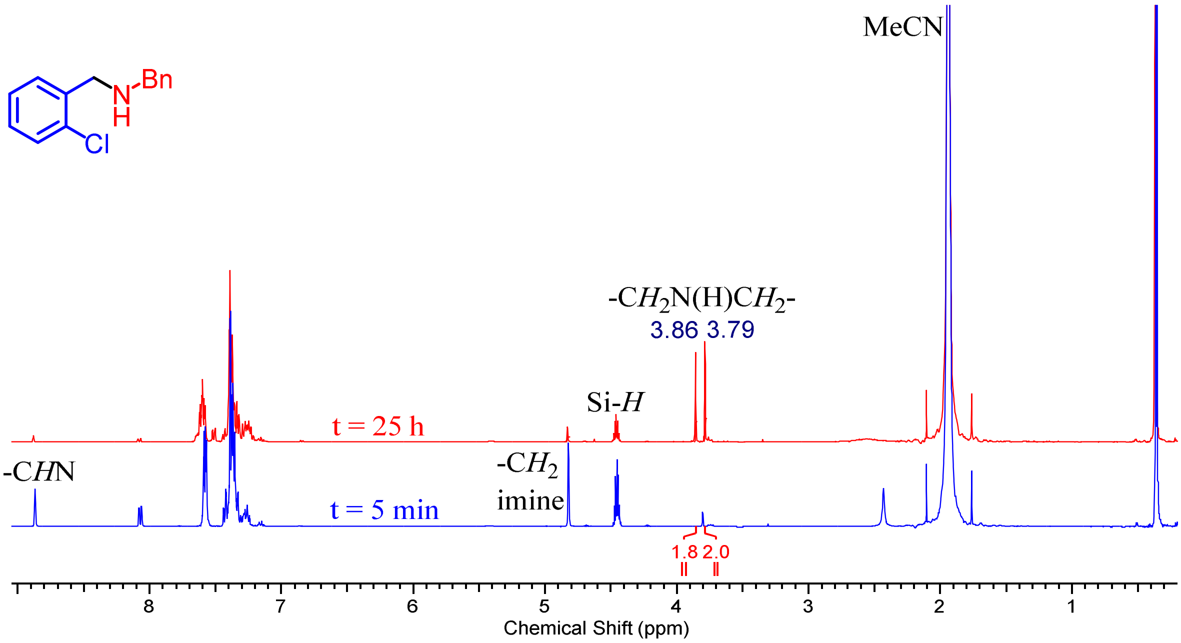

Crude - GC-MS analysis

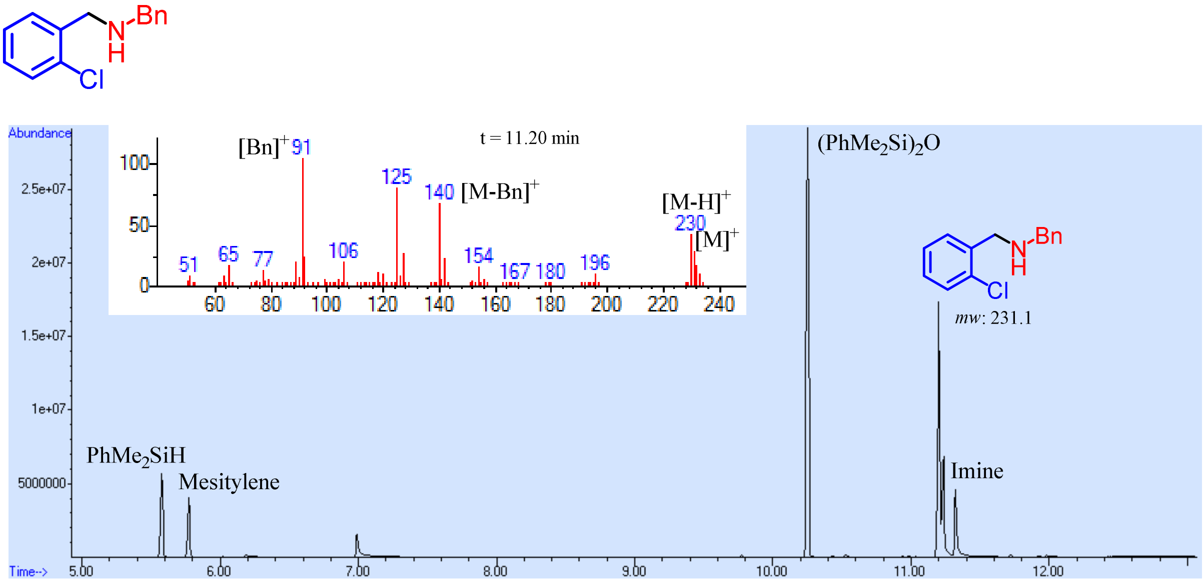

Isolated product by flash chromatography - ^{1}H and ^{13}C{^{1}H}-NMR spectra

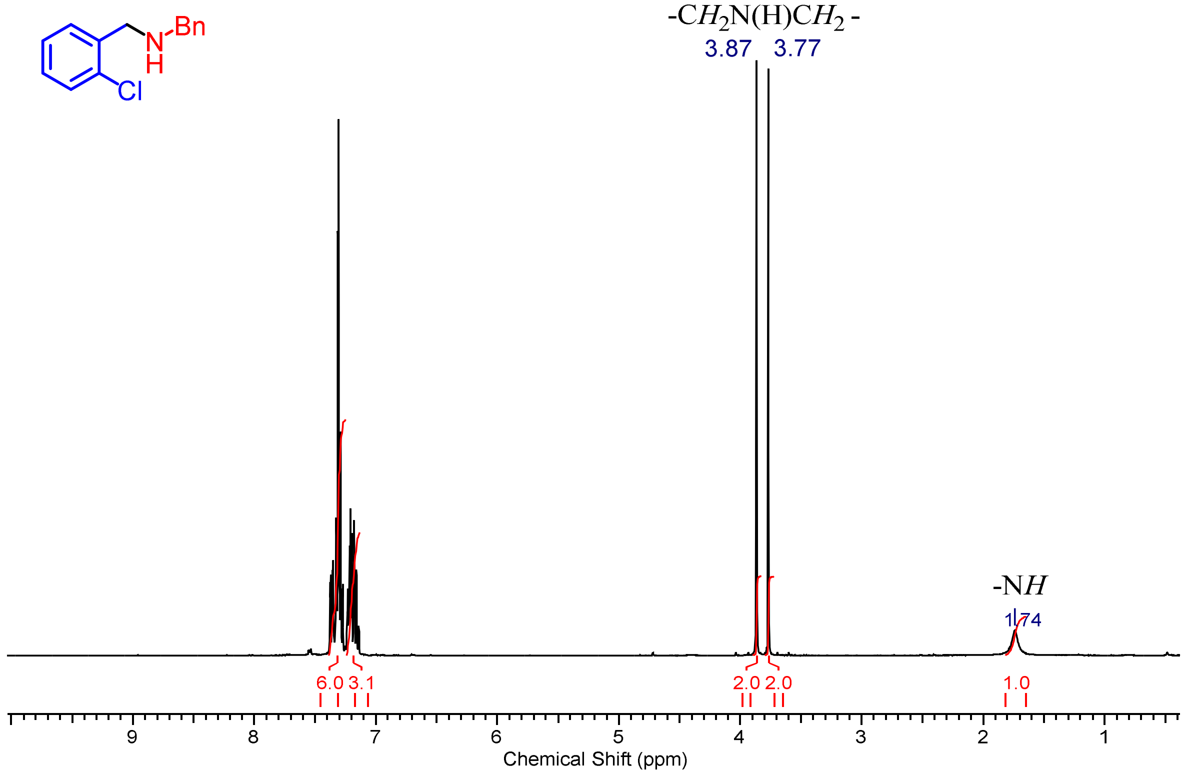

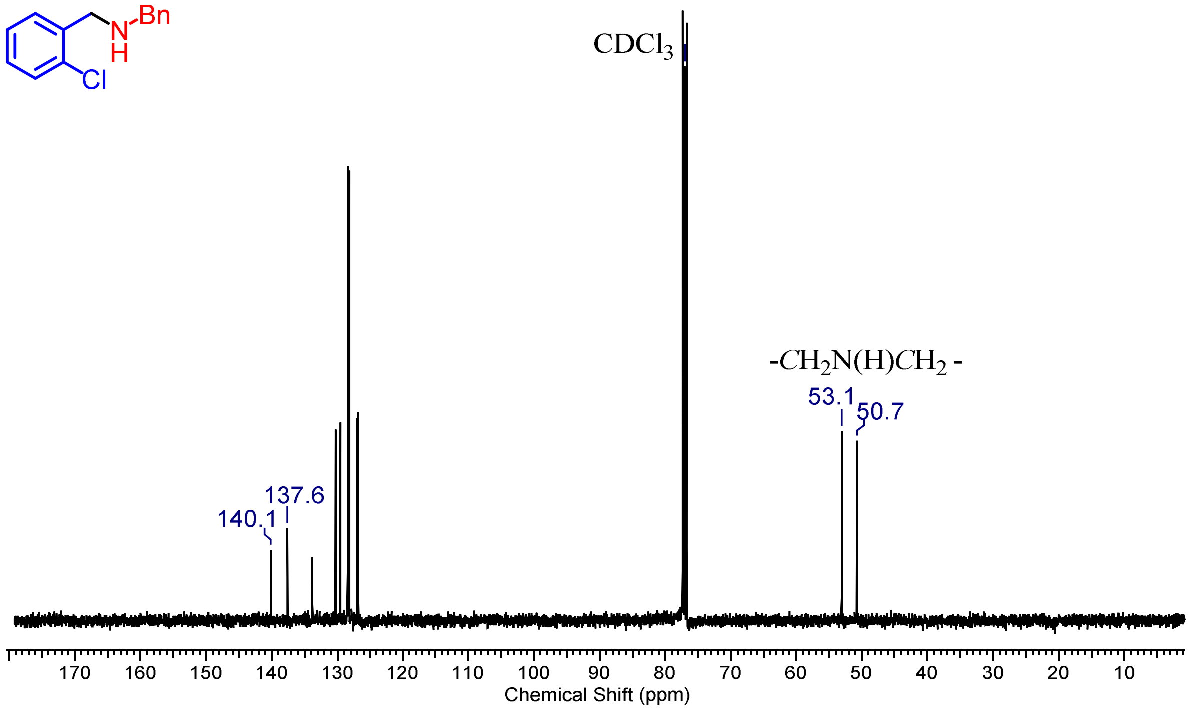

N-benzyl-1-(naphthalen-1-yl)methanamine (1b)

In situ – ^{1}H-NMR spectra

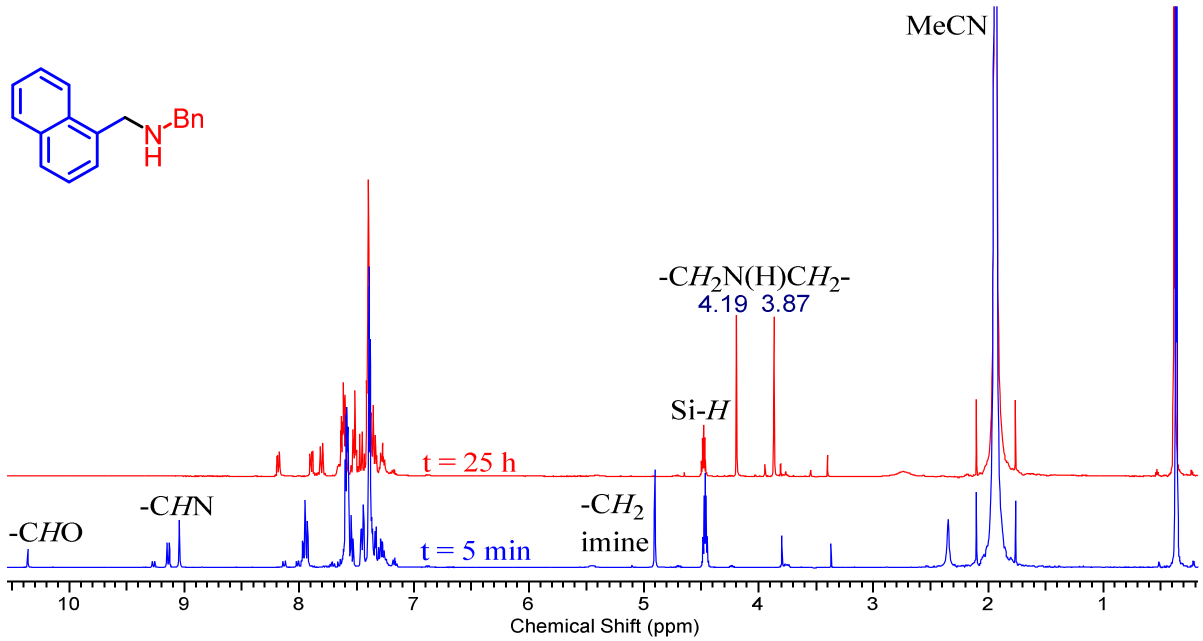

Crude - GC-MS analysis

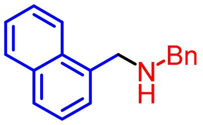

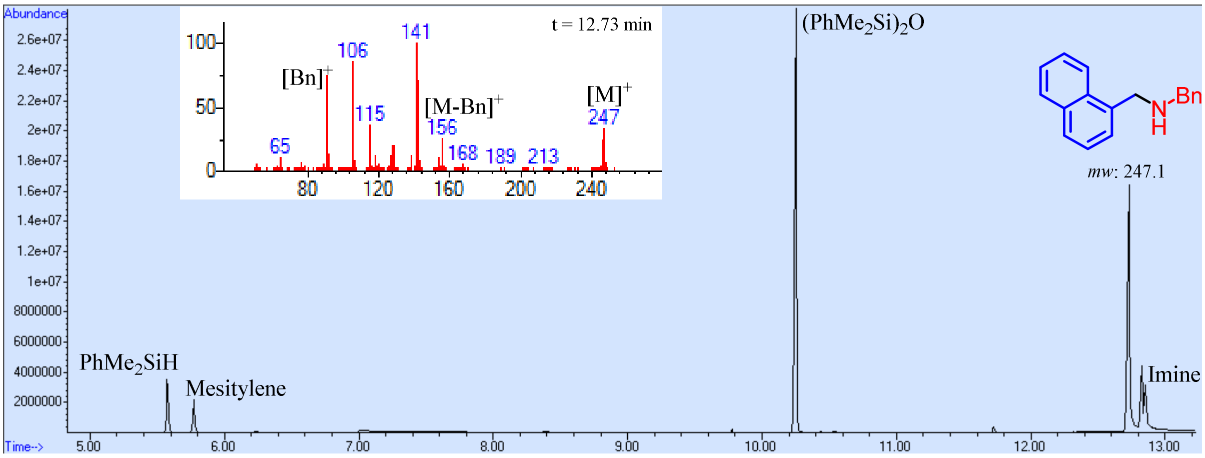

Isolated product by flash chromatography - ^{1}H and ^{13}C{^{1}H}-NMR spectra

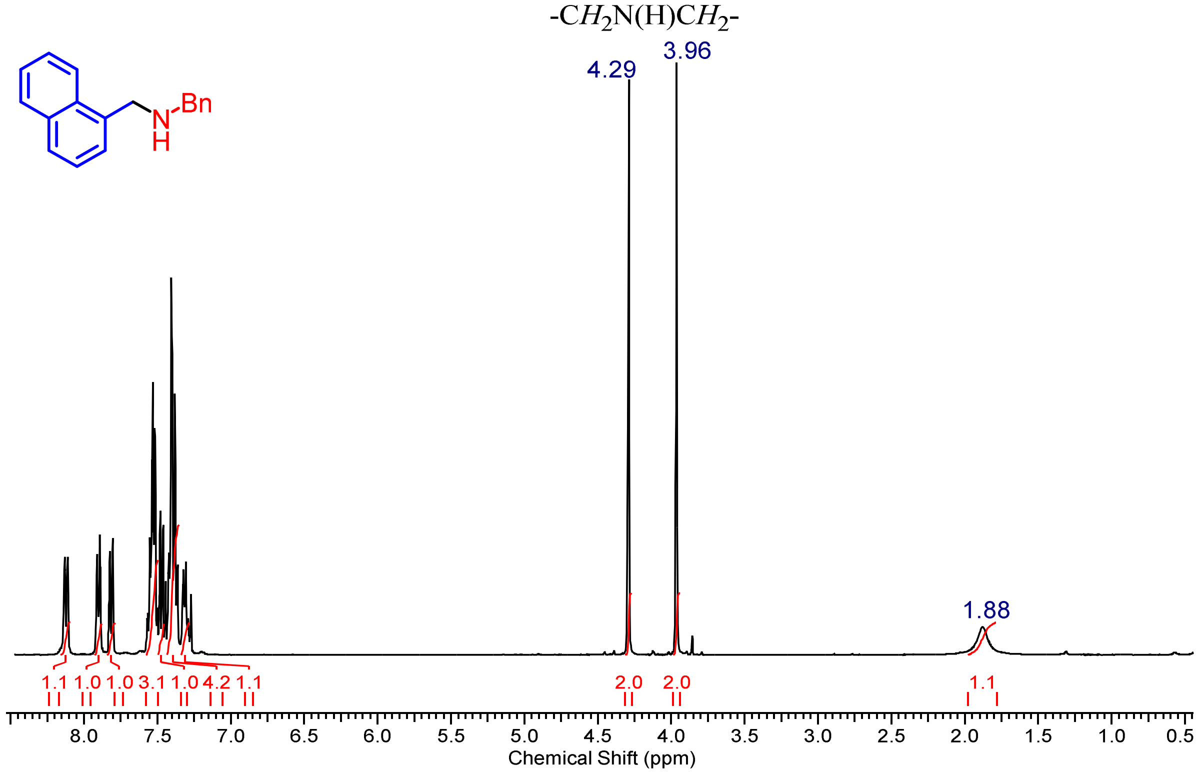

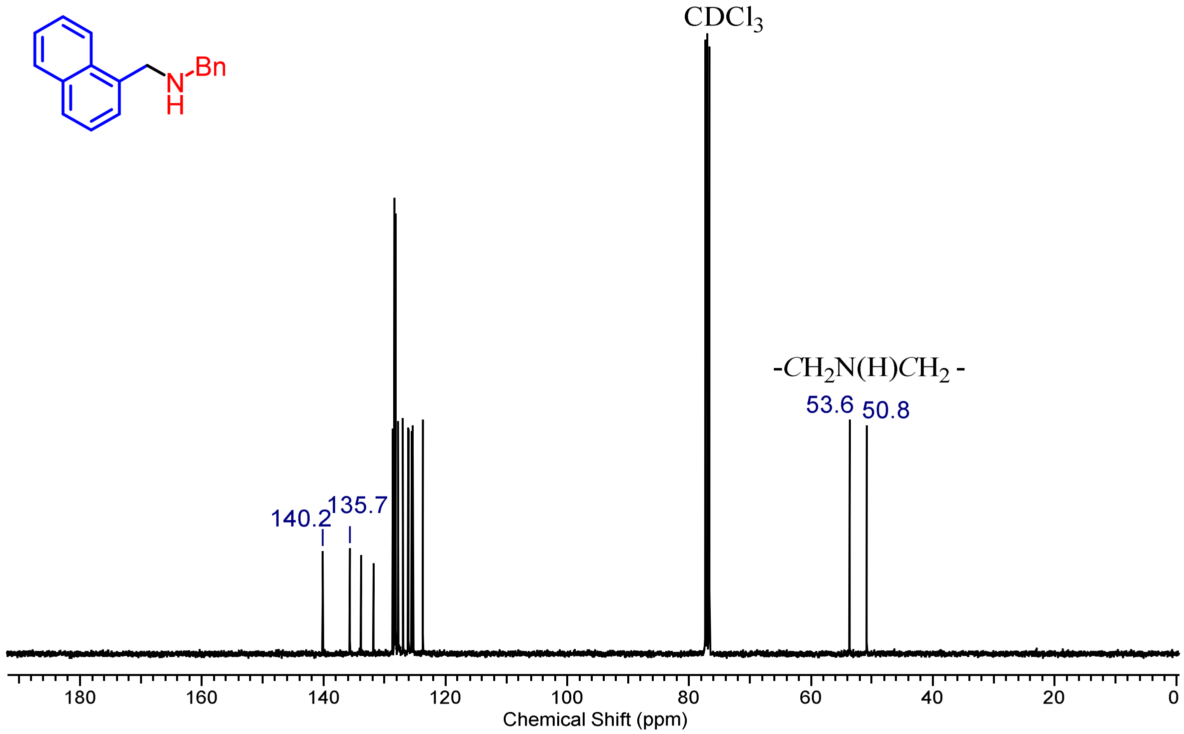

N-benzyl-1-(4-methoxyphenyl)methanamine (1c)

In situ – ^{1}H-NMR spectra

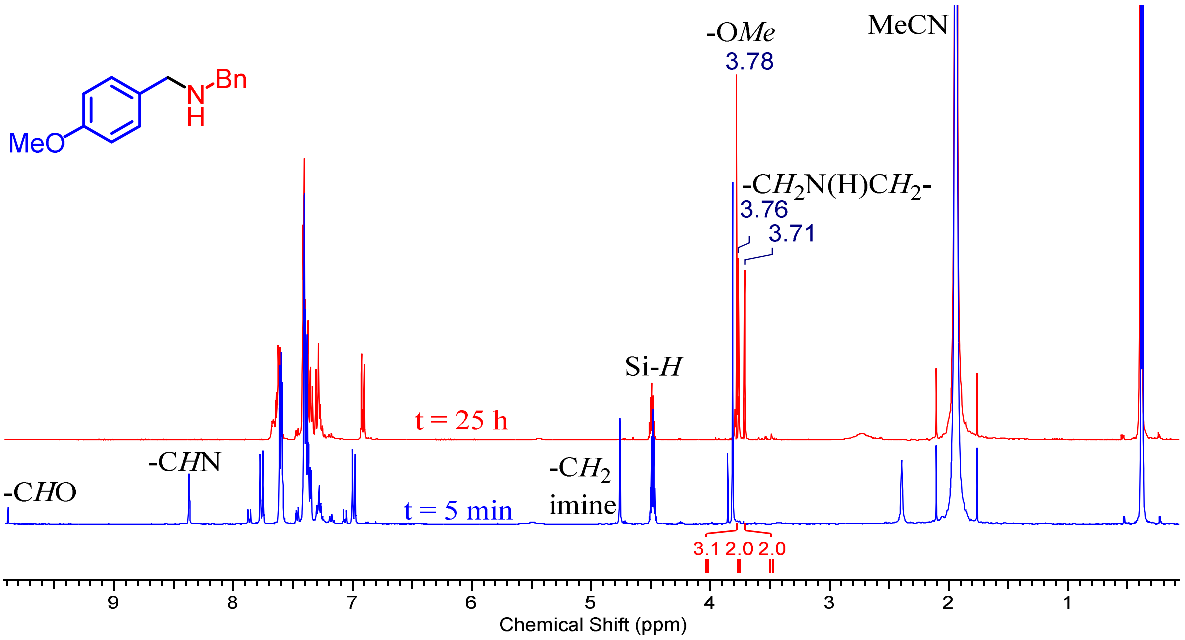

Crude - GC-MS analysis

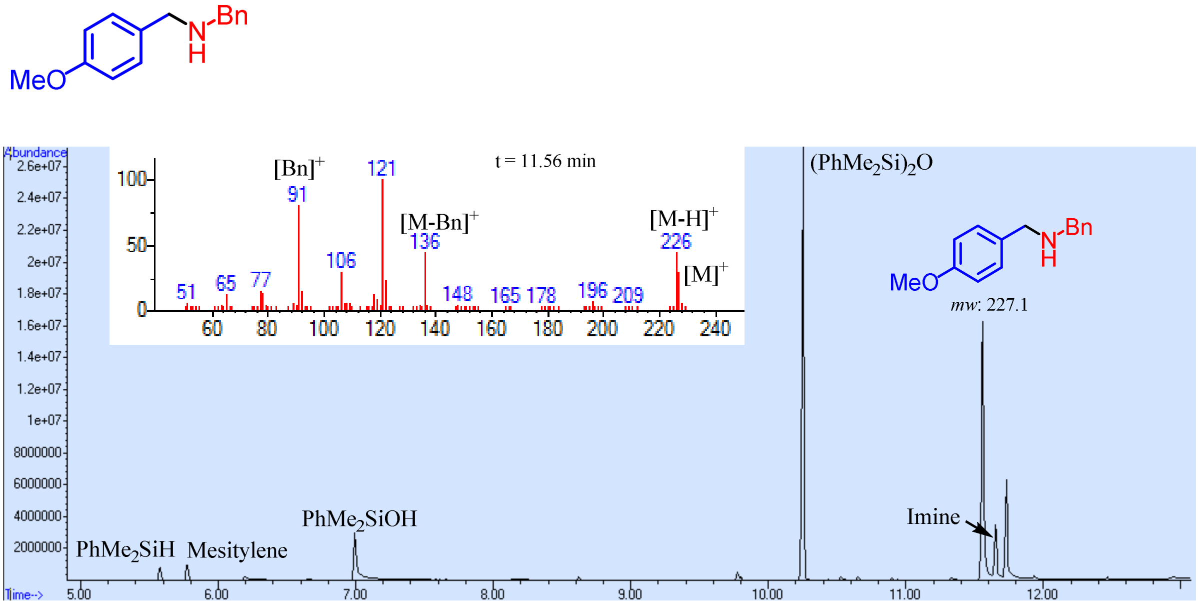

Isolated product by flash chromatography - ¹H and ¹³C{¹H}-NMR spectra

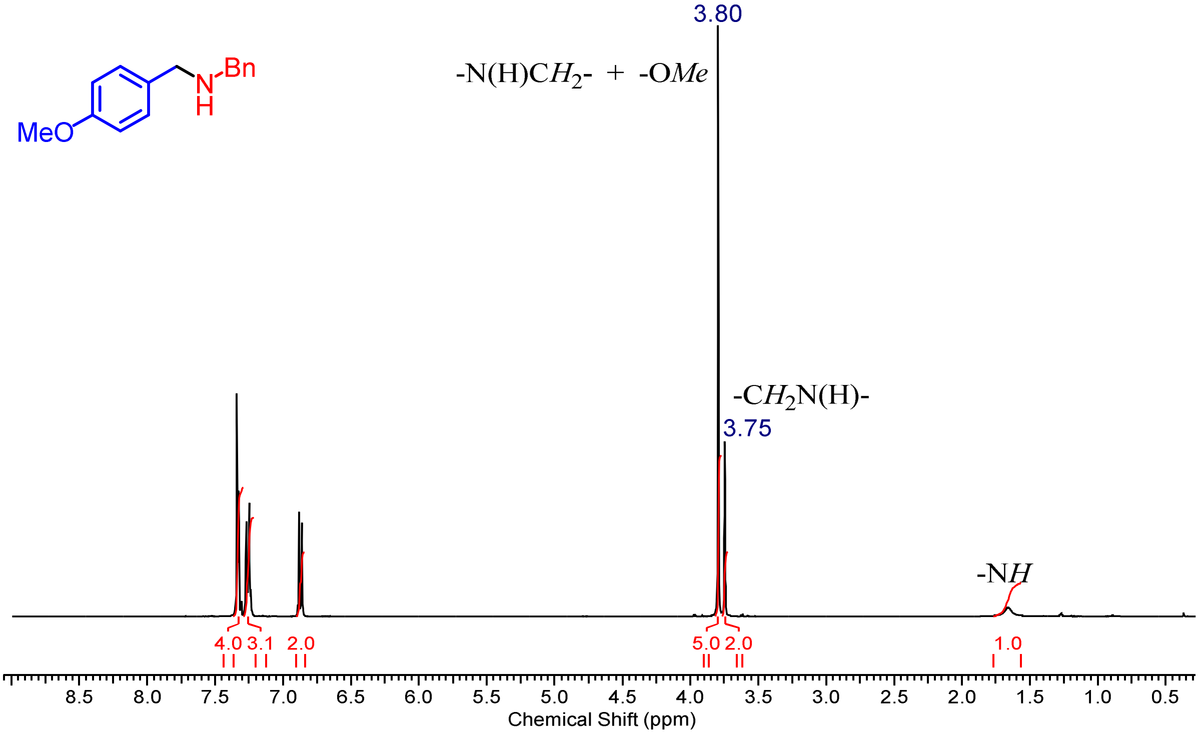

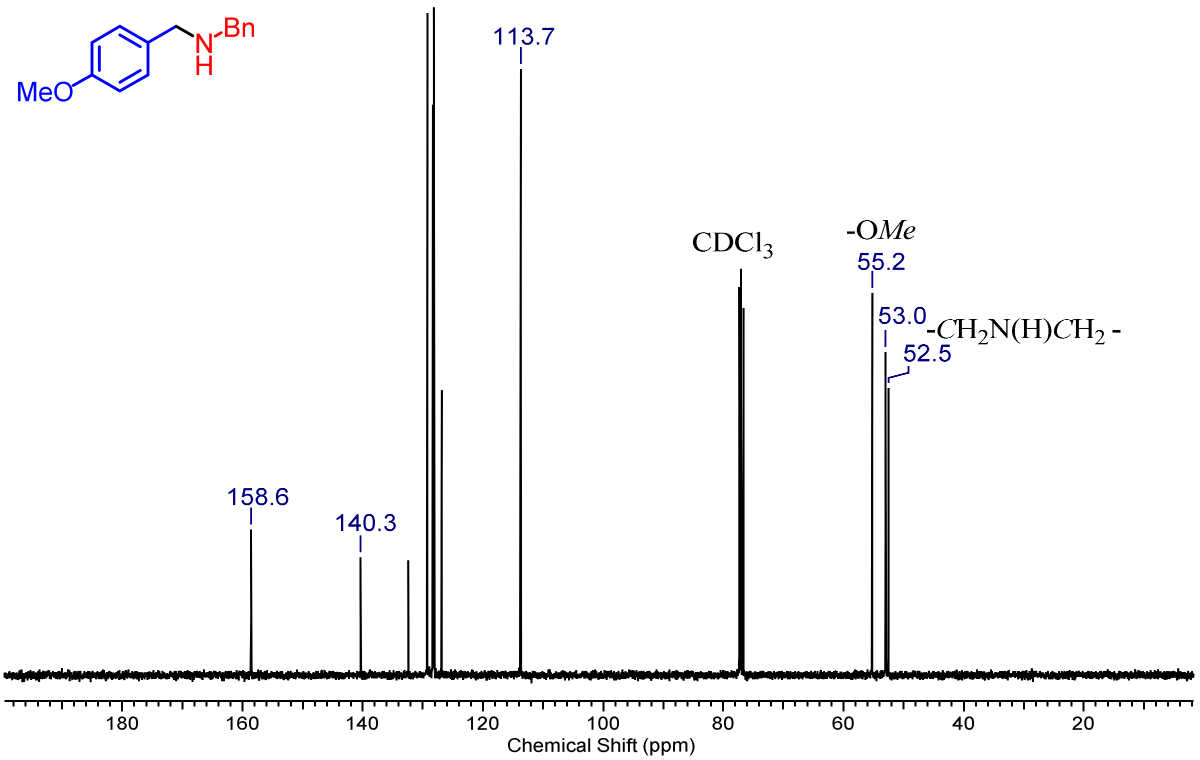

***N*-benzyl-1-(4-(tert-butyl)phenyl)methanamine (1d)**

***In situ* – ¹H-NMR spectra**

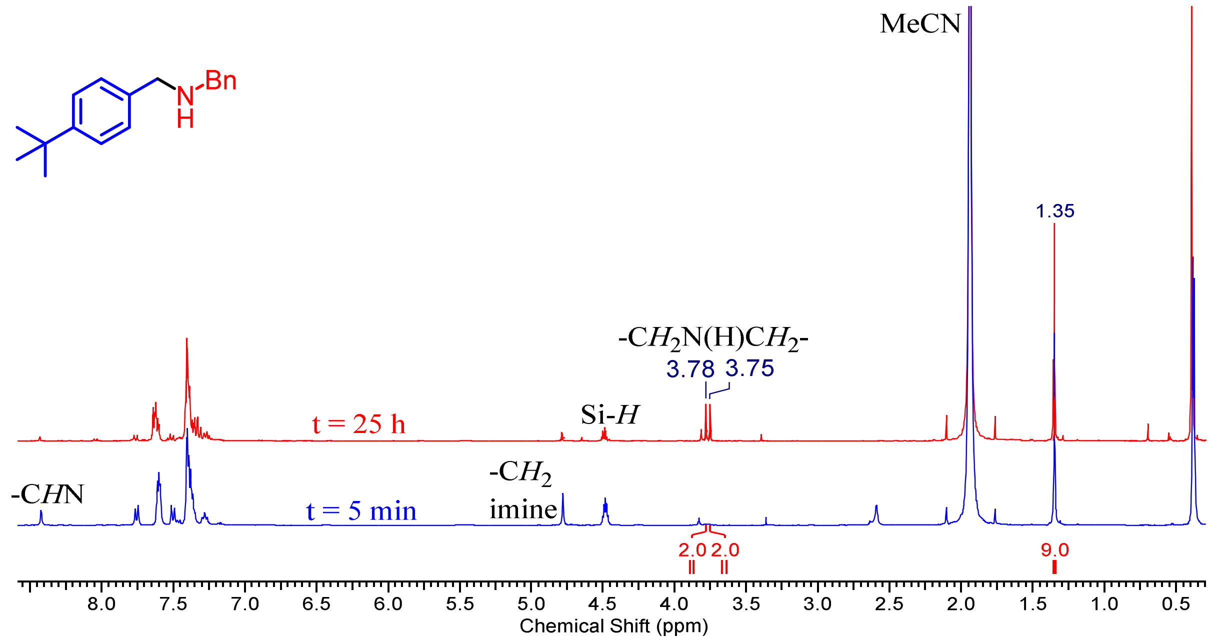

Crude - ¹H and ¹³C{¹H}-NMR spectra and GC-MS analysis

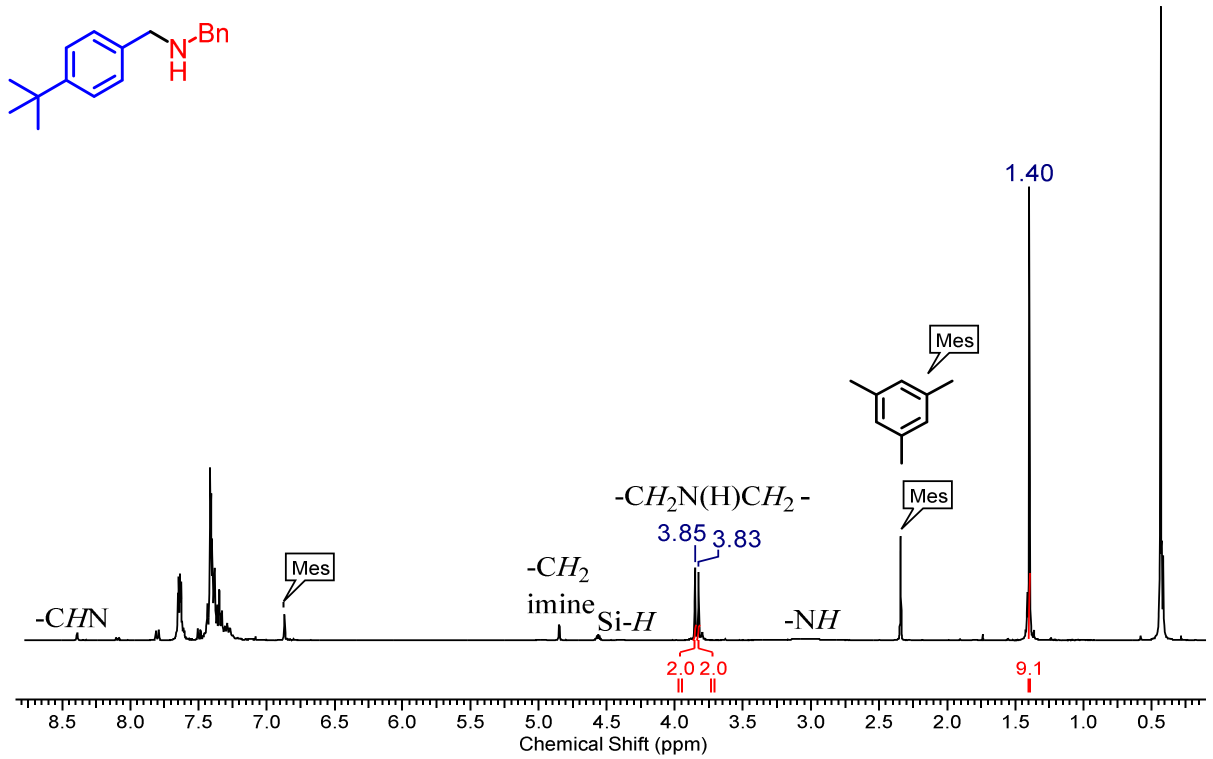

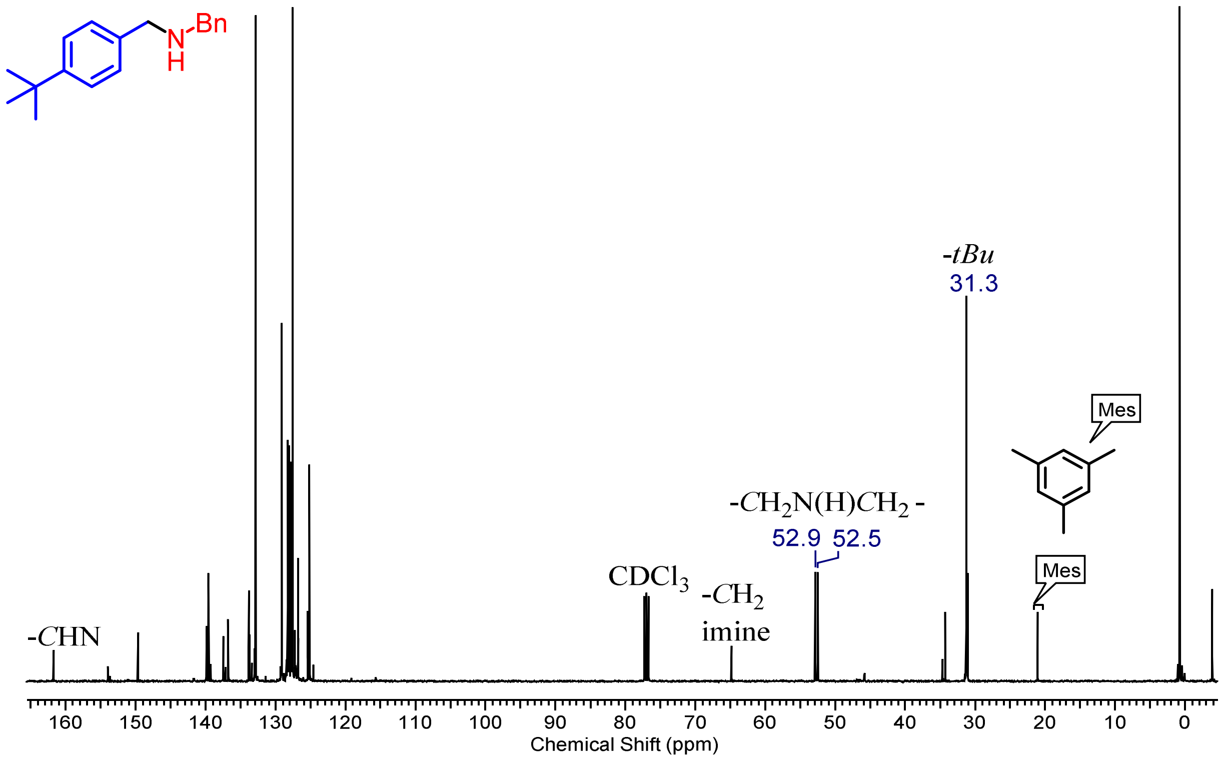
N-Bn
H
-CHN
CDCl3
-CH2
imine
-CH2N(H)CH2 -
52.9 52.5
-tBu
31.3
Mes
Mes
160 150 140 130 120 110 100 90 80 70 60 50 40 30 20 10 0
Chemical Shift (ppm)

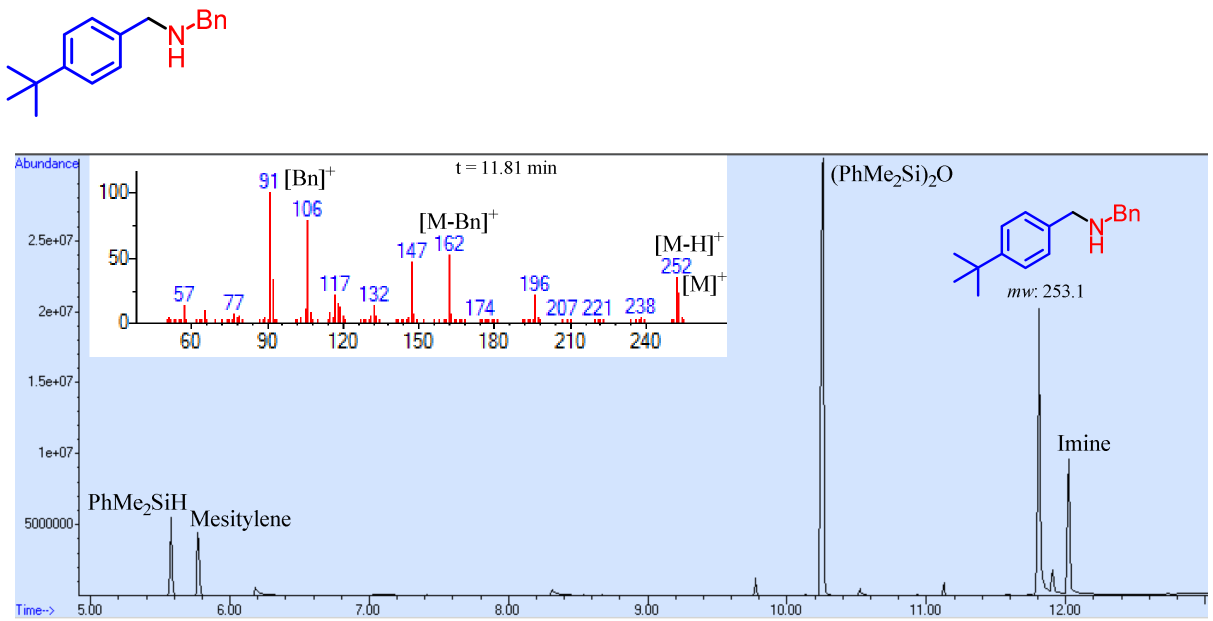
N-Bn
H
Abundance
t = 11.81 min
91 [Bn]+
106
[M-Bn]+
147 162
[M-H]+
252
[M]+
57 77 117 132 174 196 207 221 238
(PhMe2Si)2O
mw: 253.1
Imine
PhMe2SiH
Mesitylene
Time-->
5.00 6.00 7.00 8.00 9.00 10.00 11.00 12.00

N-benzyl-1-(3-bromophenyl)methanamine (1e)

***In situ* – ^{1}H-NMR spectra**

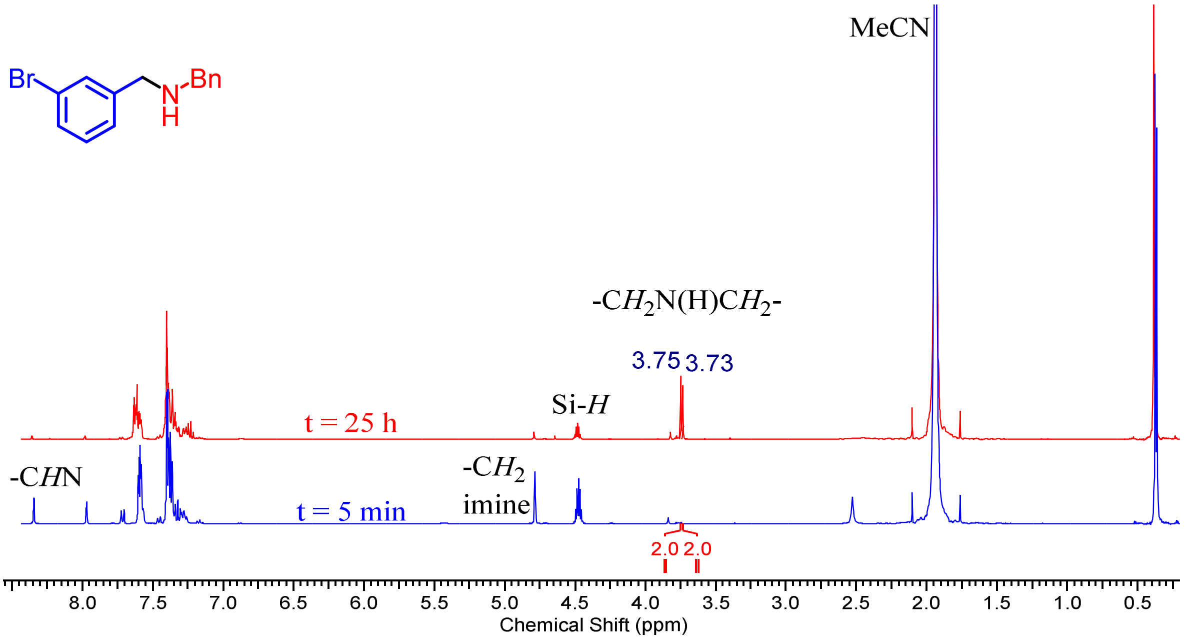

Crude - ^{1}H and ^{13}C{^{1}H}-NMR spectra and GC-MS analysis

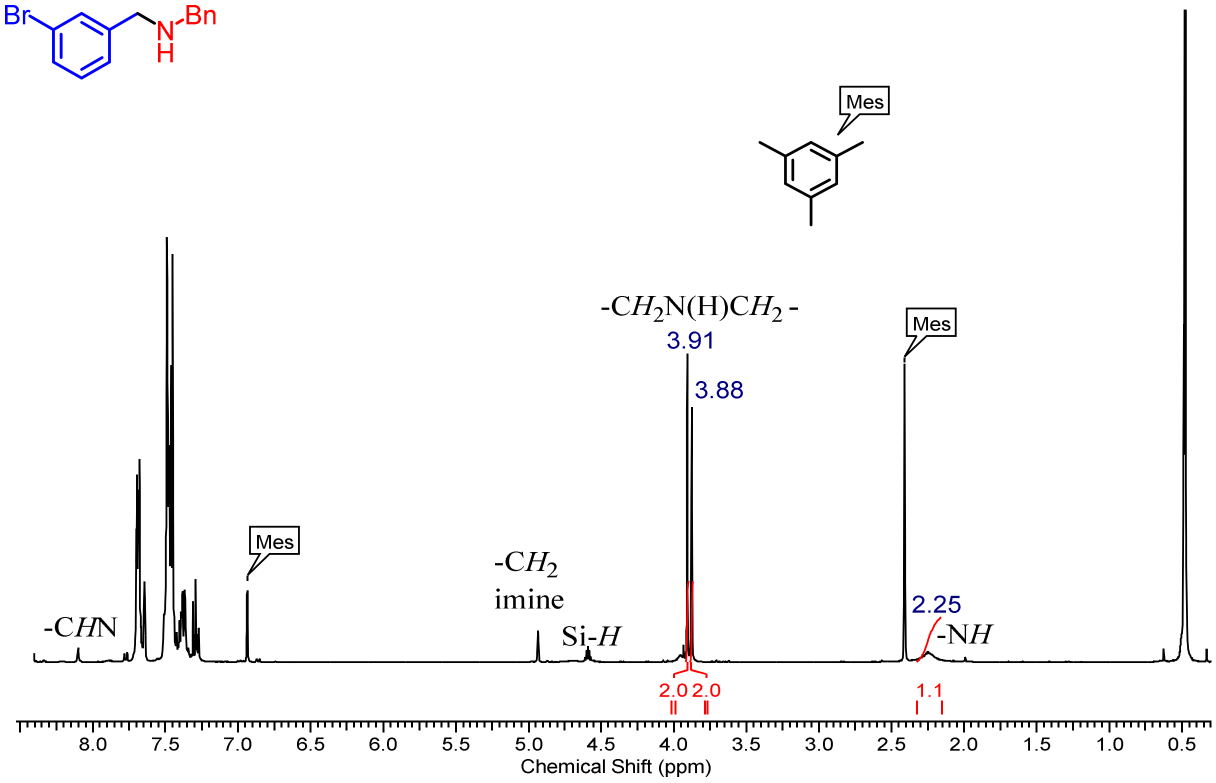

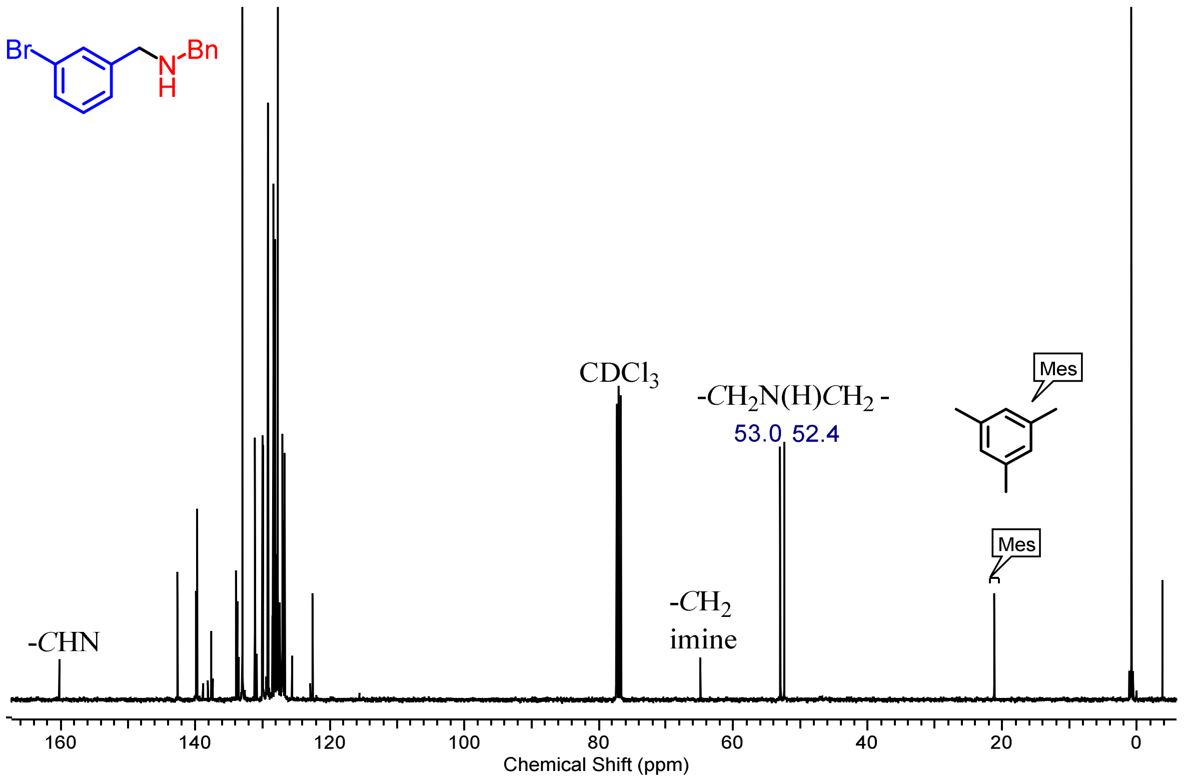
Br
N
H
Bn
CDCl3
-CH2N(H)CH2 -
53.0 52.4
Mes
Mes
-CH2
imine
-CHN
160
140
120
100
80
60
40
20
0
Chemical Shift (ppm)

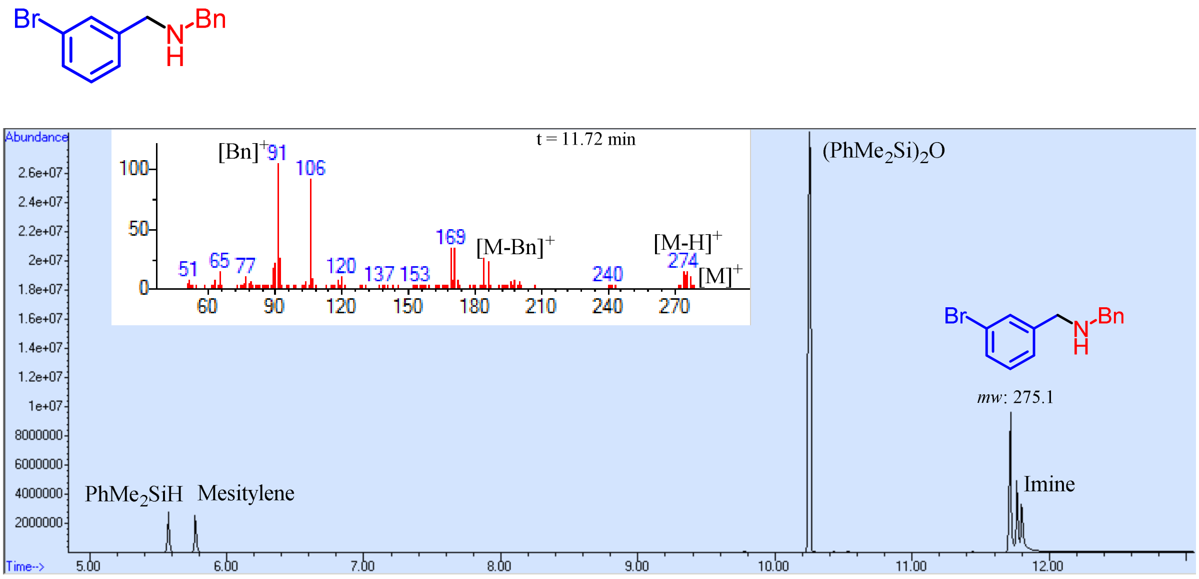
Br
N
H
Bn
Abundance
t = 11.72 min
[Bn]+ 91
106
169
[M-Bn]+
[M-H]+
274
[M]+
51
65
77
120
137
153
240
(PhMe2Si)2O
mw: 275.1
Imine
PhMe2SiH
Mesitylene
Time-->
5.00
6.00
7.00
8.00
9.00
10.00
11.00
12.00

Methyl 4-((benzylamino)methyl)benzoate (1f)

In situ – ^{1}H-NMR spectra

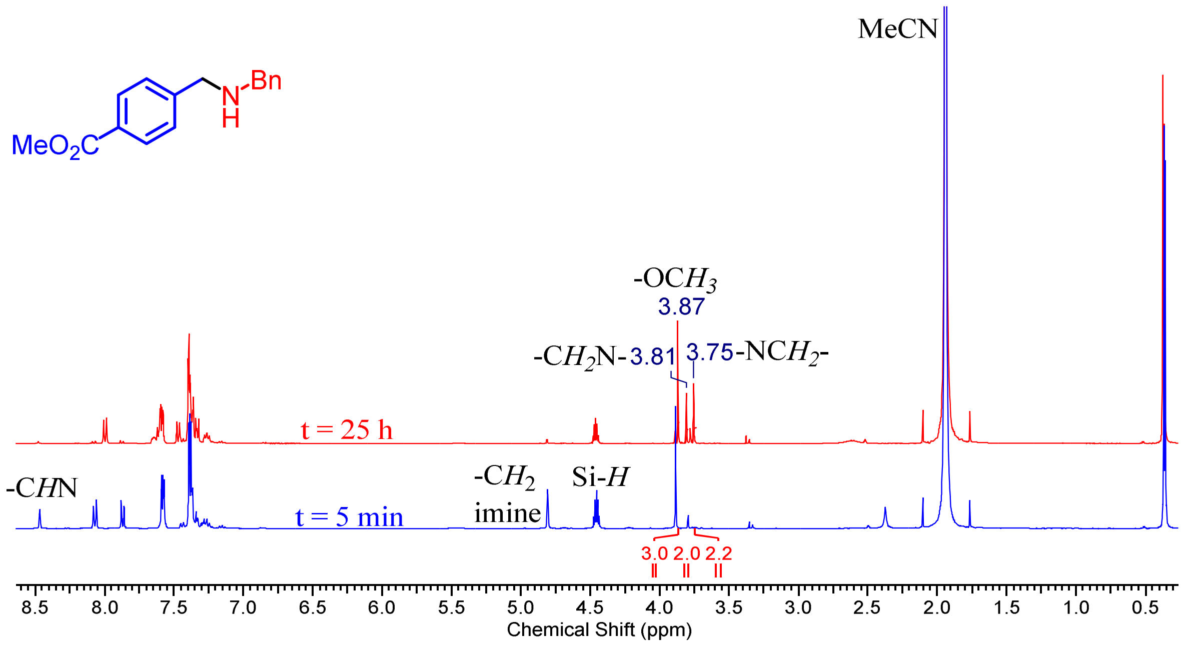

Crude - ^{1}H and ^{13}C{^{1}H}-NMR spectra and GC-MS analysis

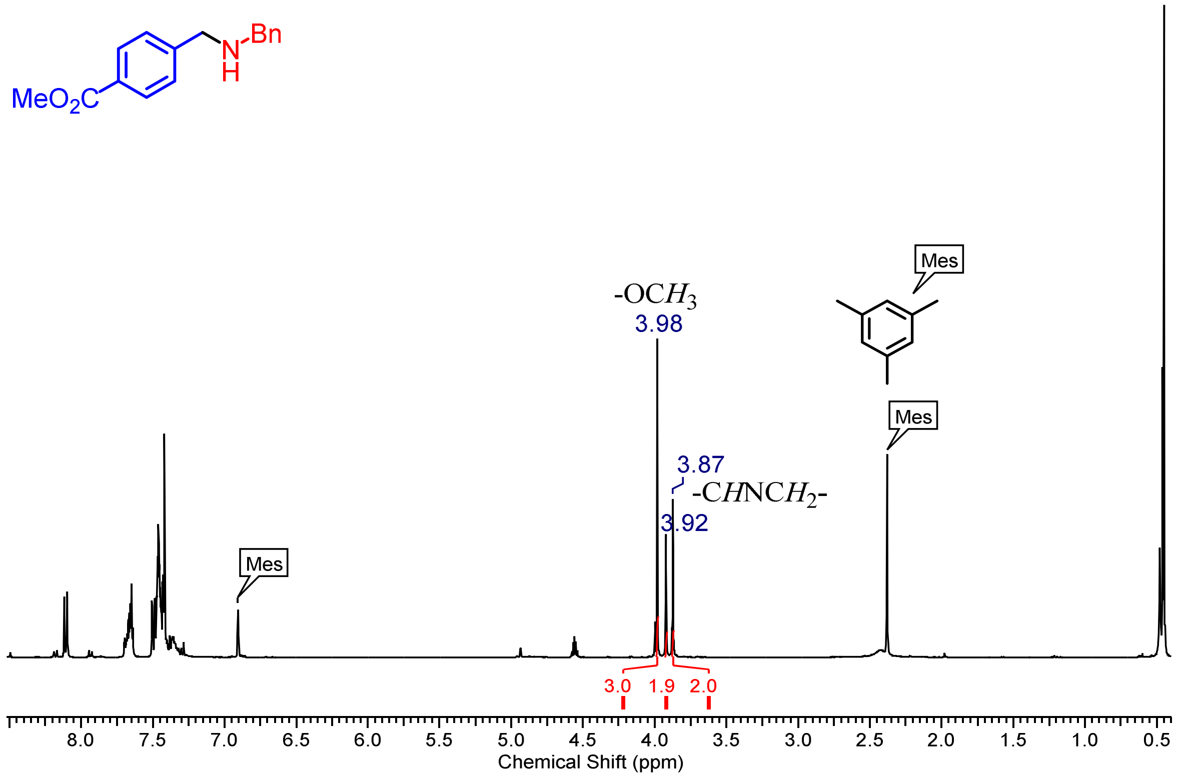

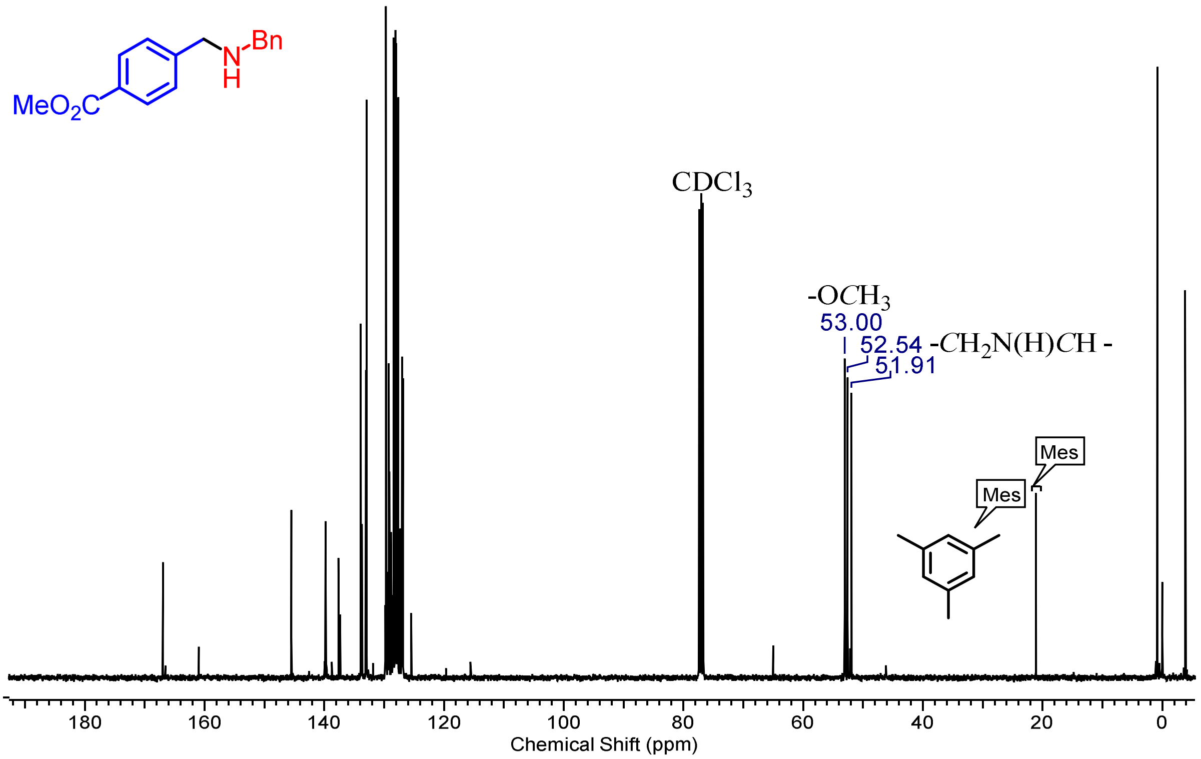
MeO2C
N
H
Bn
CDCl3
-OCH3
53.00
52.54 -CH2N(H)CH -
51.91
Mes
Mes
180
160
140
120
100
80
60
40
20
0
Chemical Shift (ppm)

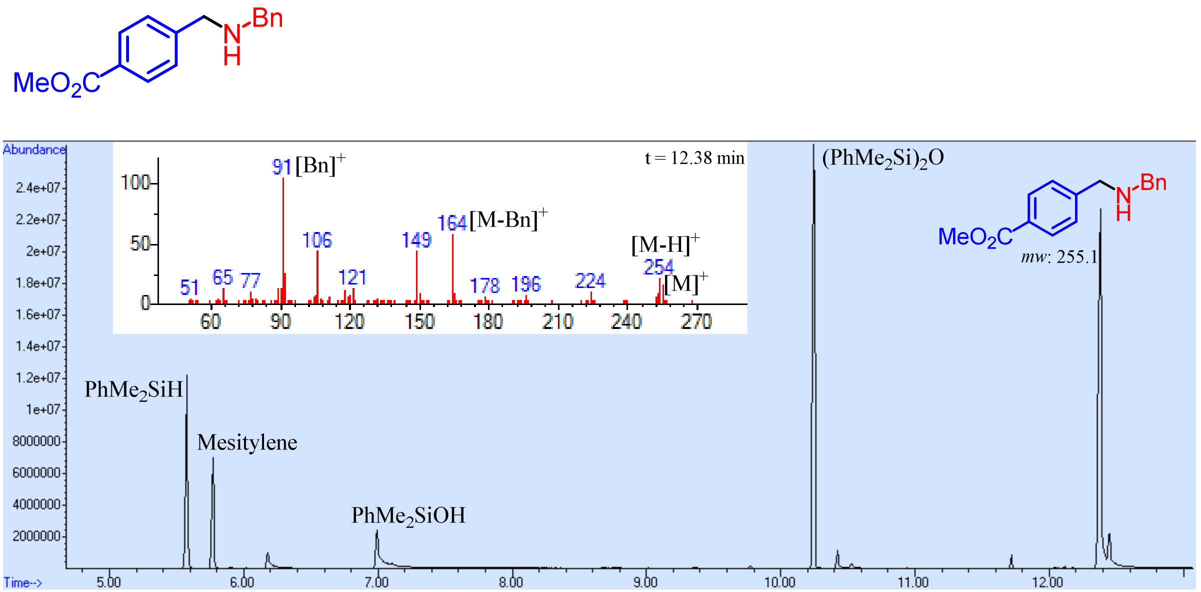
MeO2C
N
H
Bn
Abundance
t = 12.38 min
91 [Bn]+
164 [M-Bn]+
[M-H]+
254
[M]+
51
65
77
106
121
149
178
196
224
(PhMe2Si)2O
mw: 255.1
PhMe2SiH
Mesitylene
PhMe2SiOH
Time-->
5.00
6.00
7.00
8.00
9.00
10.00
11.00
12.00

4-((benzylamino)methyl)benzonitrile (1g)

In situ – ^{1}H-NMR spectra

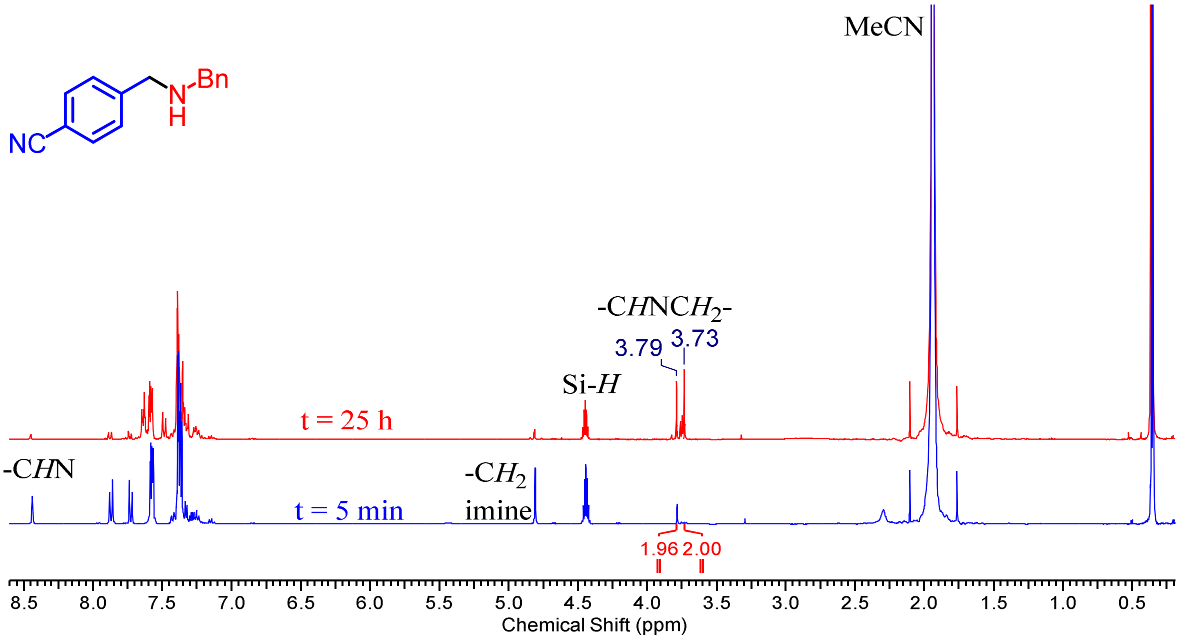

Crude - ^{1}H and ^{13}C{^{1}H}-NMR spectra and GC-MS analysis

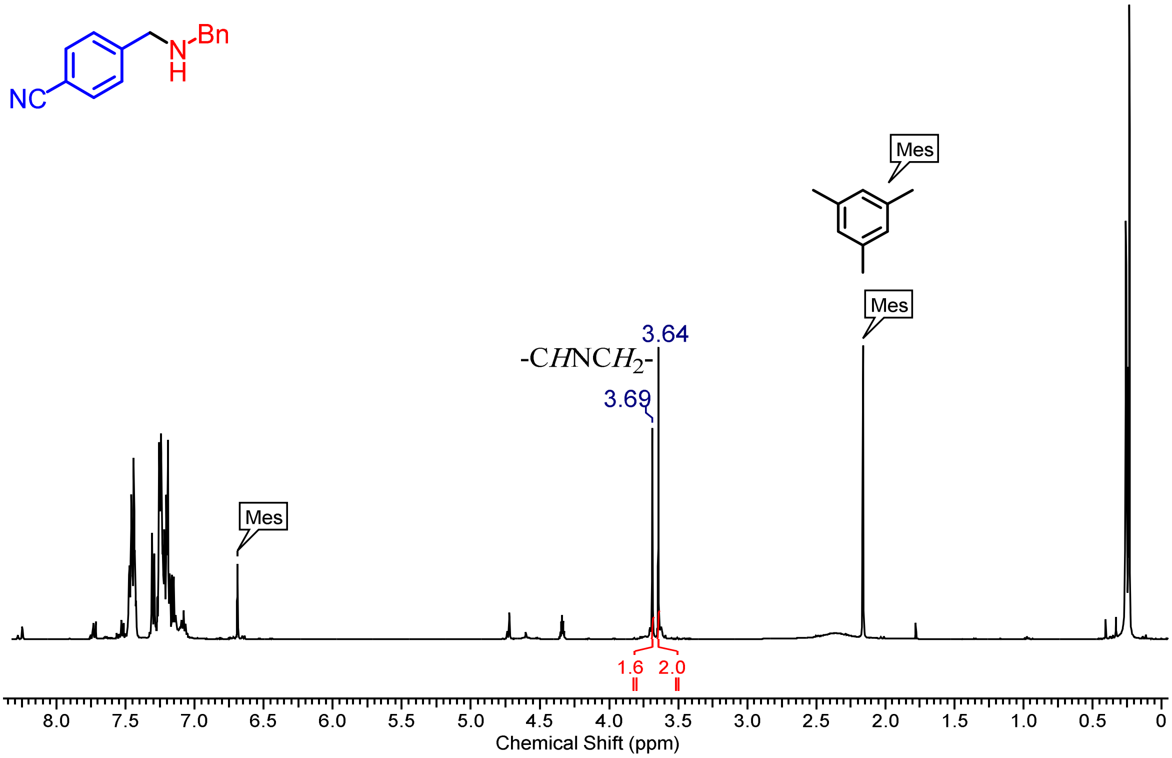

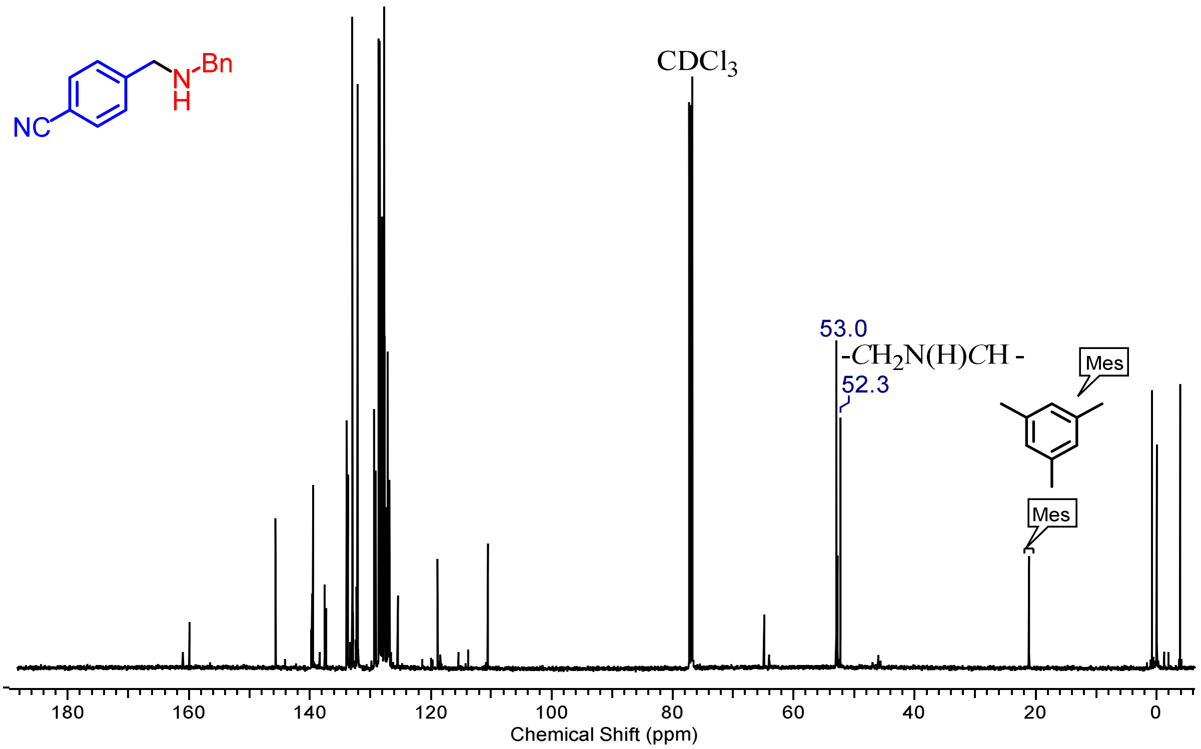
NC
N
H
Bn
CDCl3
53.0
-CH2N(H)CH -
52.3
Mes
Mes
180
160
140
120
100
80
60
40
20
0
Chemical Shift (ppm)

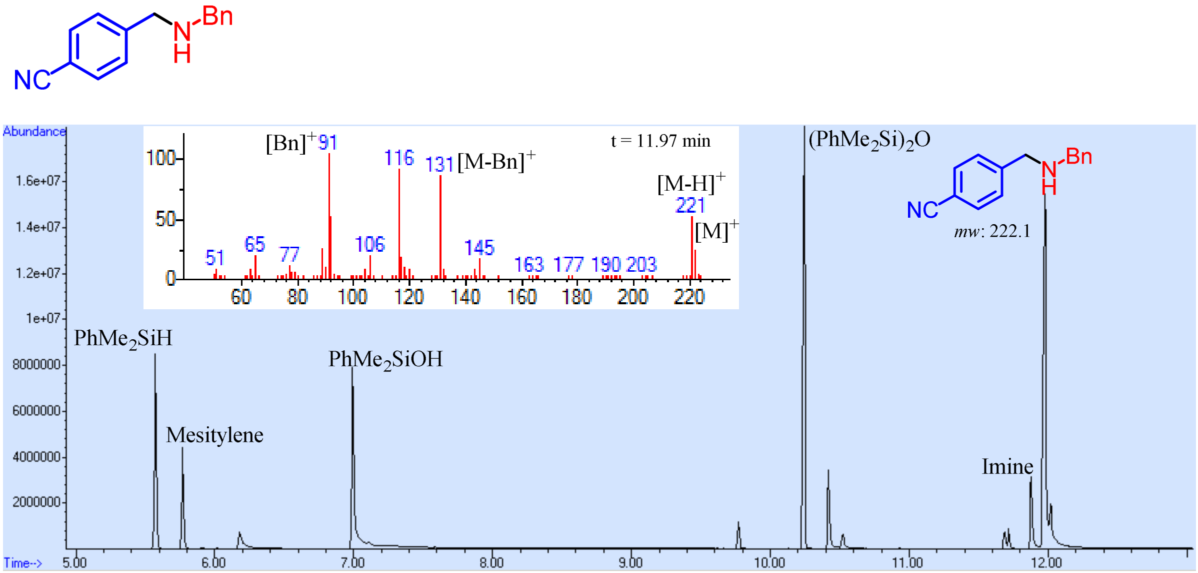
NC
N
H
Bn
Abundance
1.6e+07
1.4e+07
1.2e+07
1e+07
8000000
6000000
4000000
2000000
Time-->
5.00
6.00
7.00
8.00
9.00
10.00
11.00
12.00
[Bn]+ 91
116
131 [M-Bn]+
t = 11.97 min
[M-H]+
221
[M]+
51
65
77
106
145
163
177
190
203
100
50
0
60
80
100
120
140
160
180
200
220
(PhMe2Si)2O
mw: 222.1
PhMe2SiH
Mesitylene
PhMe2SiOH
Imine

N-benzyl-1-(4-nitrophenyl)methanamine (1h)

In situ – ^{1}H-NMR spectra

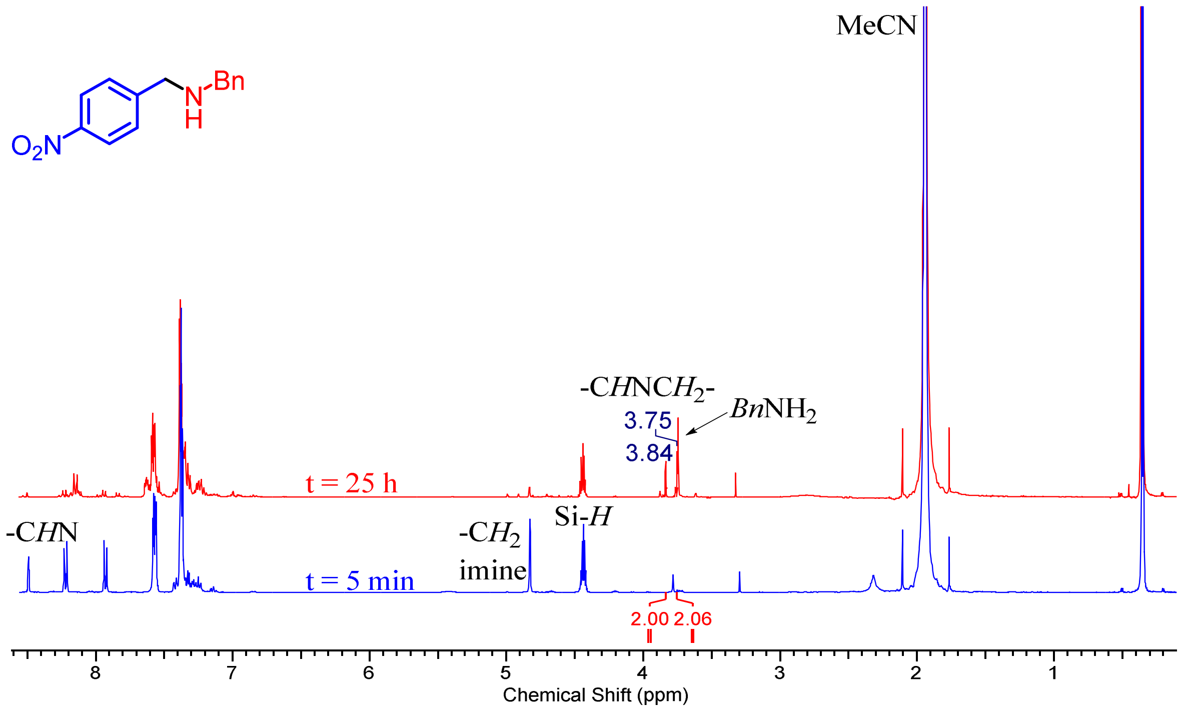

Crude - ^{1}H and ^{13}C{^{1}H}-NMR spectra and GC-MS analysis

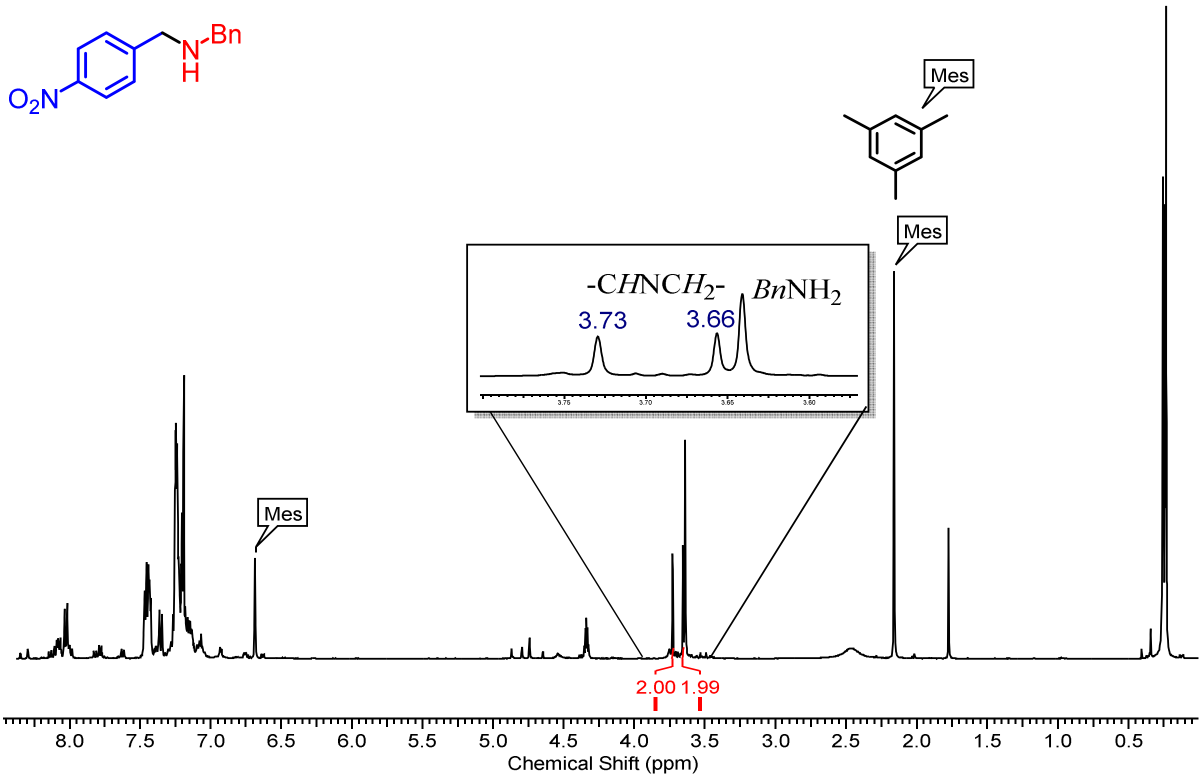

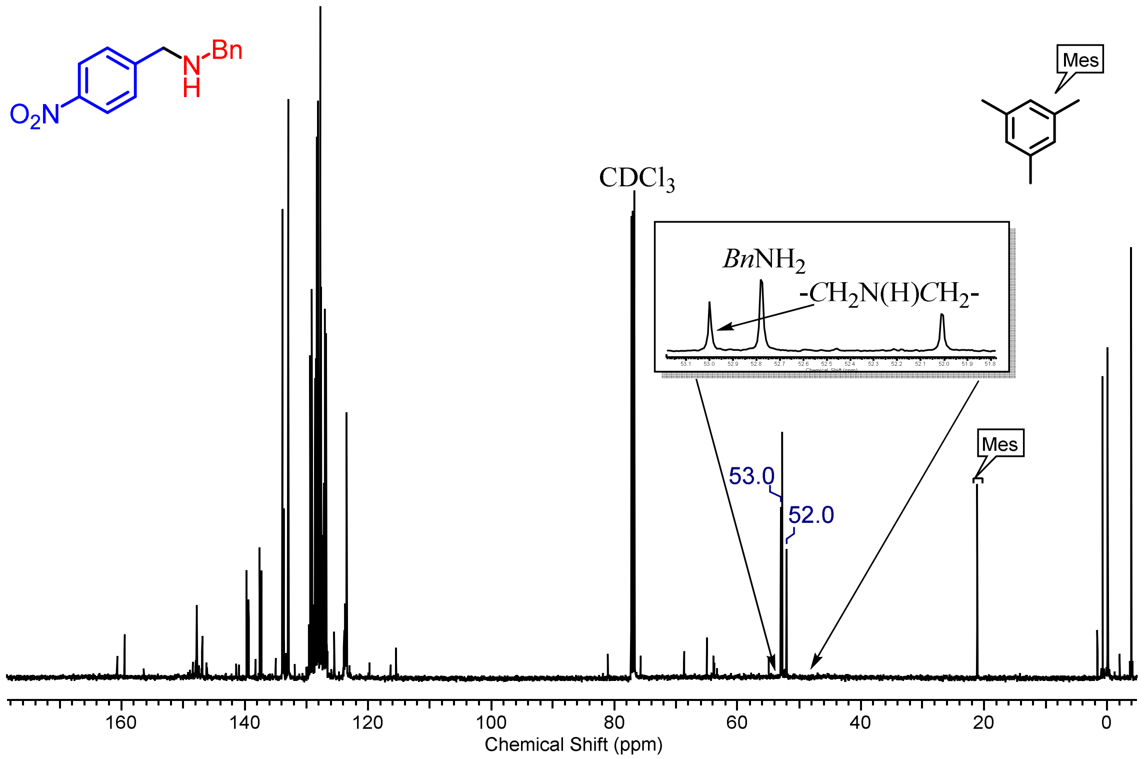

O2N
N
H
Bn
Mes
CDCl3
BnNH2
-CH2N(H)CH2-
53.0
52.0
Mes
160
140
120
100
80
60
40
20
0
Chemical Shift (ppm)

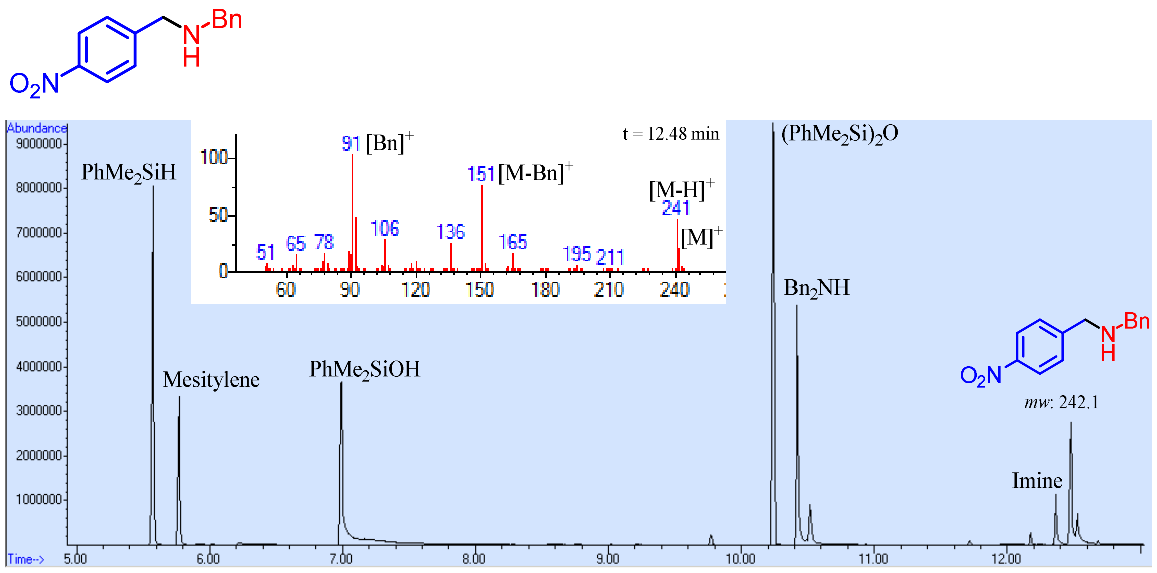

O2N
N
H
Bn
Abundance
9000000
8000000
7000000
6000000
5000000
4000000
3000000
2000000
1000000
PhMe2SiH
Mesitylene
PhMe2SiOH
(PhMe2Si)2O
Bn2NH
Imine
mw: 242.1
t = 12.48 min
91 [Bn]+
151 [M-Bn]+
[M-H]+
241
[M]+
51
65
78
106
136
165
195
211
100
50
0
60
90
120
150
180
210
240
Time-->
5.00
6.00
7.00
8.00
9.00
10.00
11.00
12.00

N-benzyl-1-(4-ethynylphenyl)methanamine (1i)

In situ – ^{1}H-NMR spectra

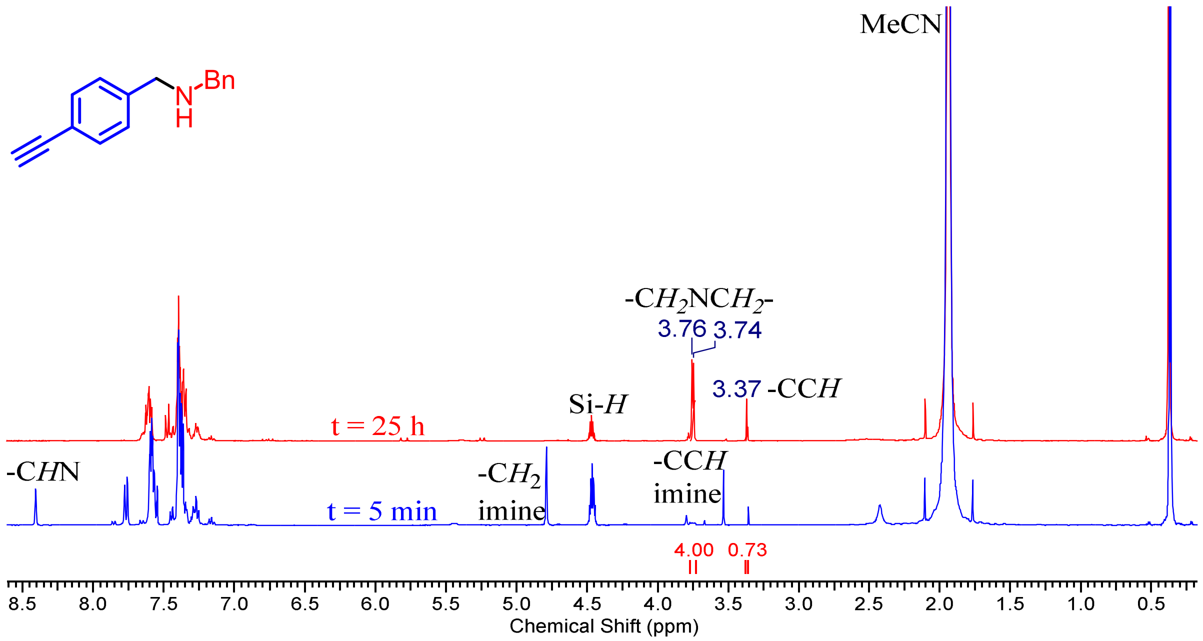

Crude - ^{1}H and ^{13}C{^{1}H}-NMR spectra and GC-MS analysis

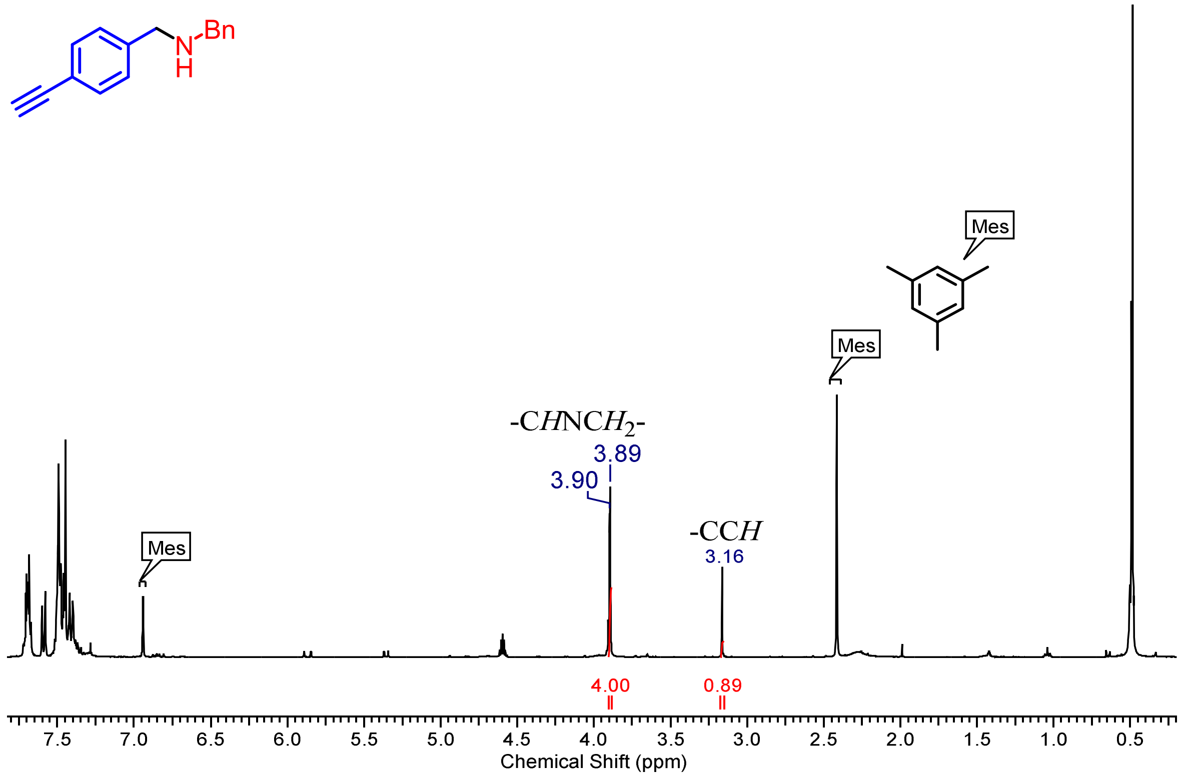

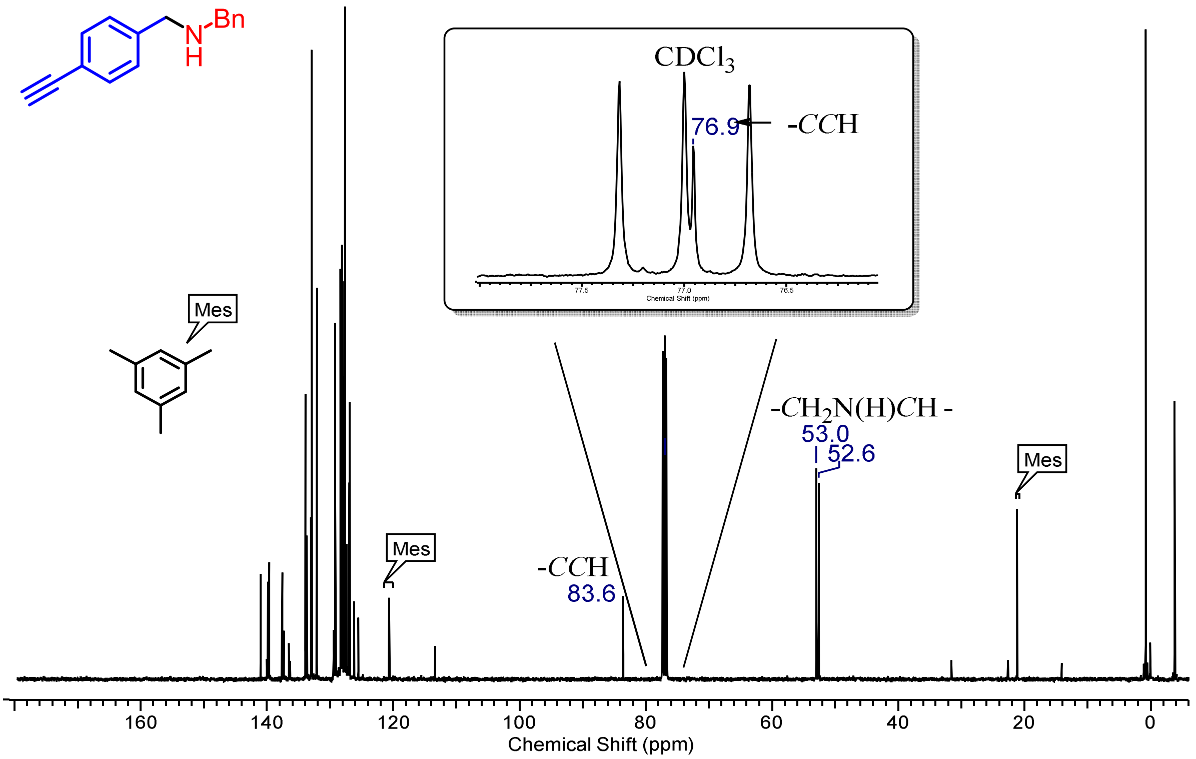
Bn
N
H
Mes
CDCl3
76.9
-CCH
-CCH
83.6
-CH2N(H)CH -
53.0
52.6
Mes
Mes
160
140
120
100
80
60
40
20
0
Chemical Shift (ppm)

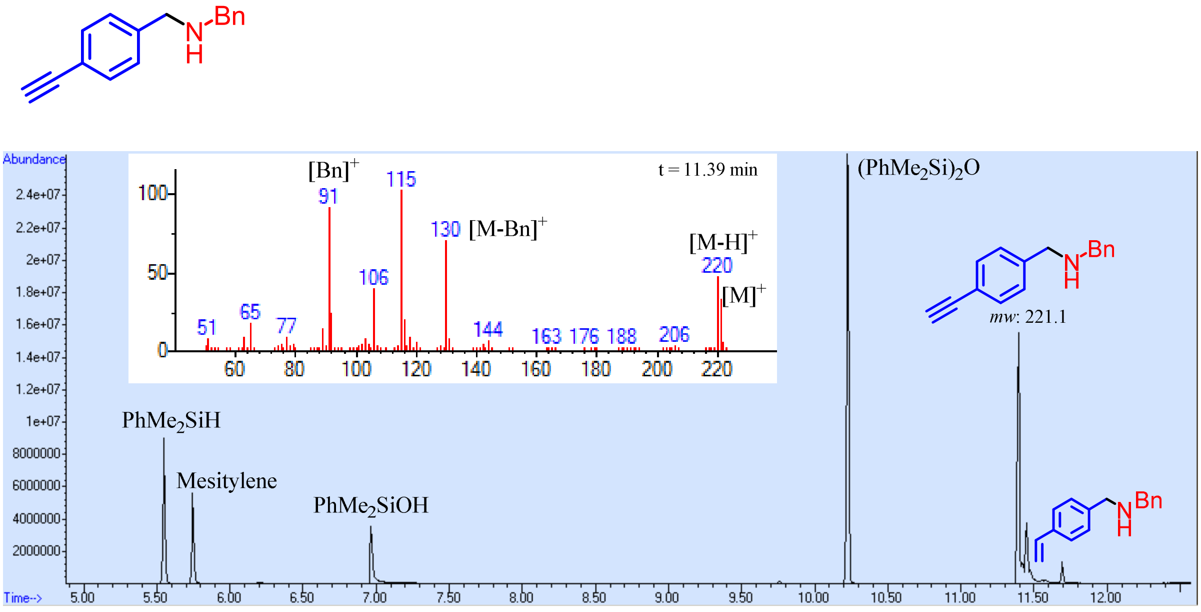
Bn
N
H
Abundance
t = 11.39 min
[Bn]+
91
115
130 [M-Bn]+
106
[M-H]+
220
[M]+
51
65
77
144
163 176 188
206
(PhMe2Si)2O
mw: 221.1
PhMe2SiH
Mesitylene
PhMe2SiOH
Time-->

N-benzyl-1-phenylethan-1-amine (1j)

In situ – ^{1}H-NMR spectra

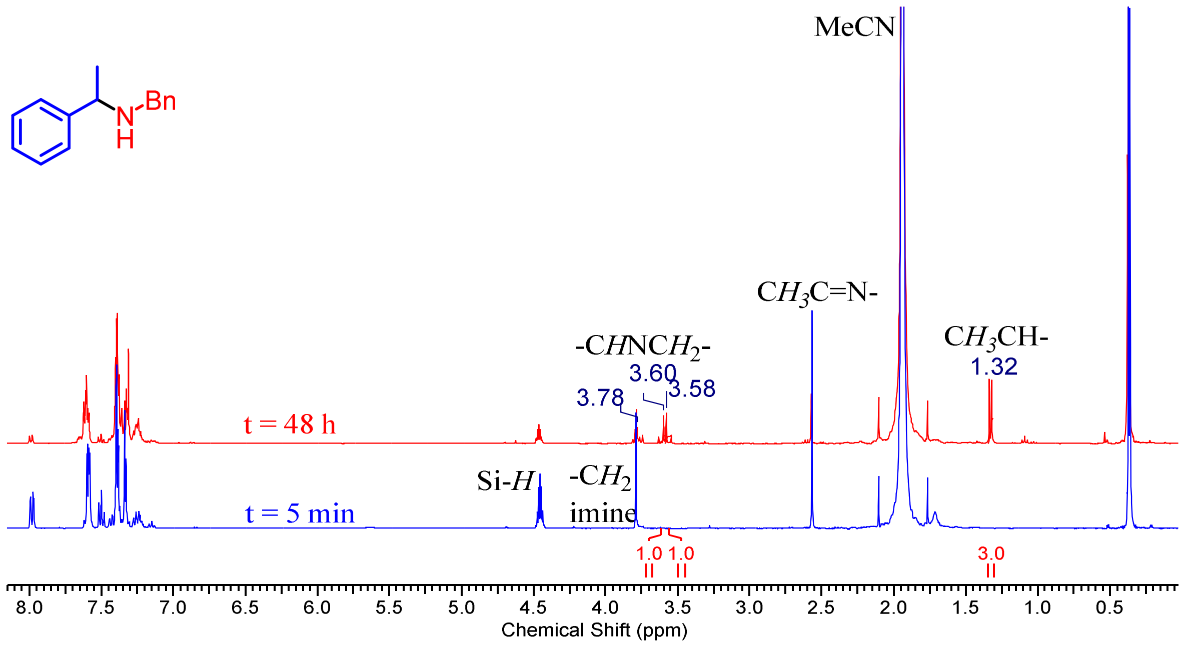

Crude - ^{1}H and ^{13}C{^{1}H}-NMR spectra and GC-MS analysis

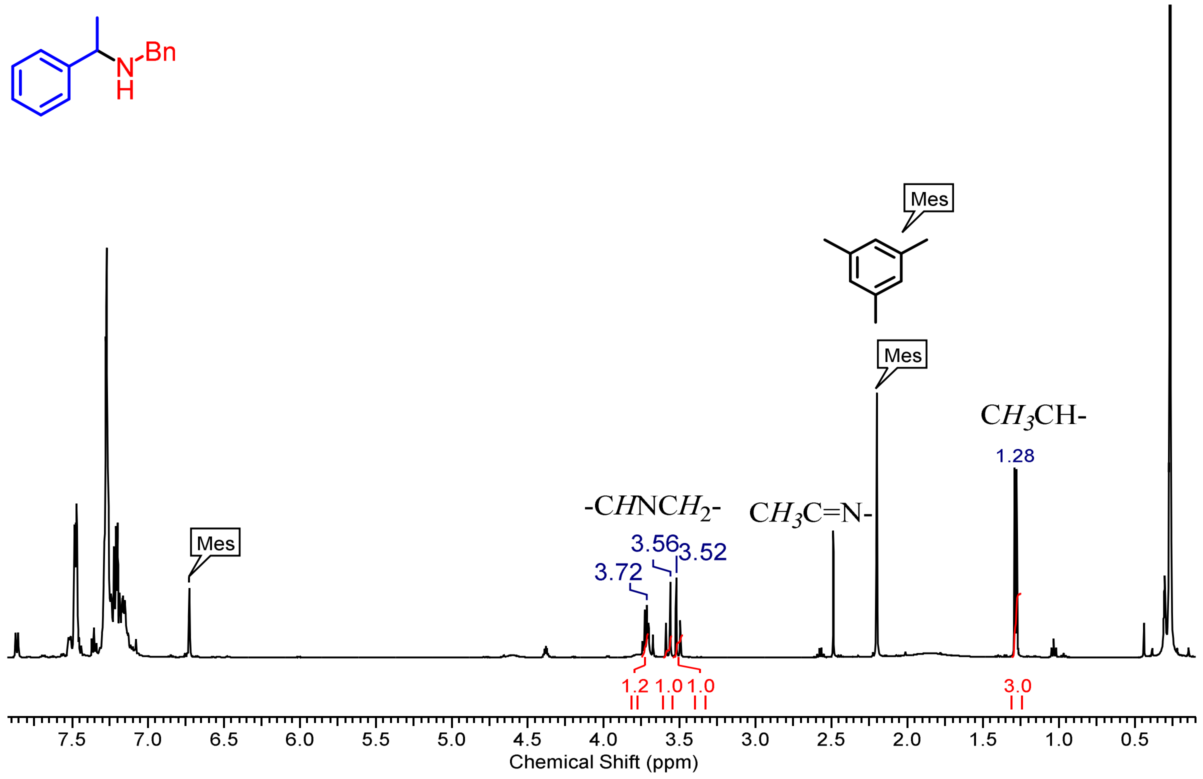

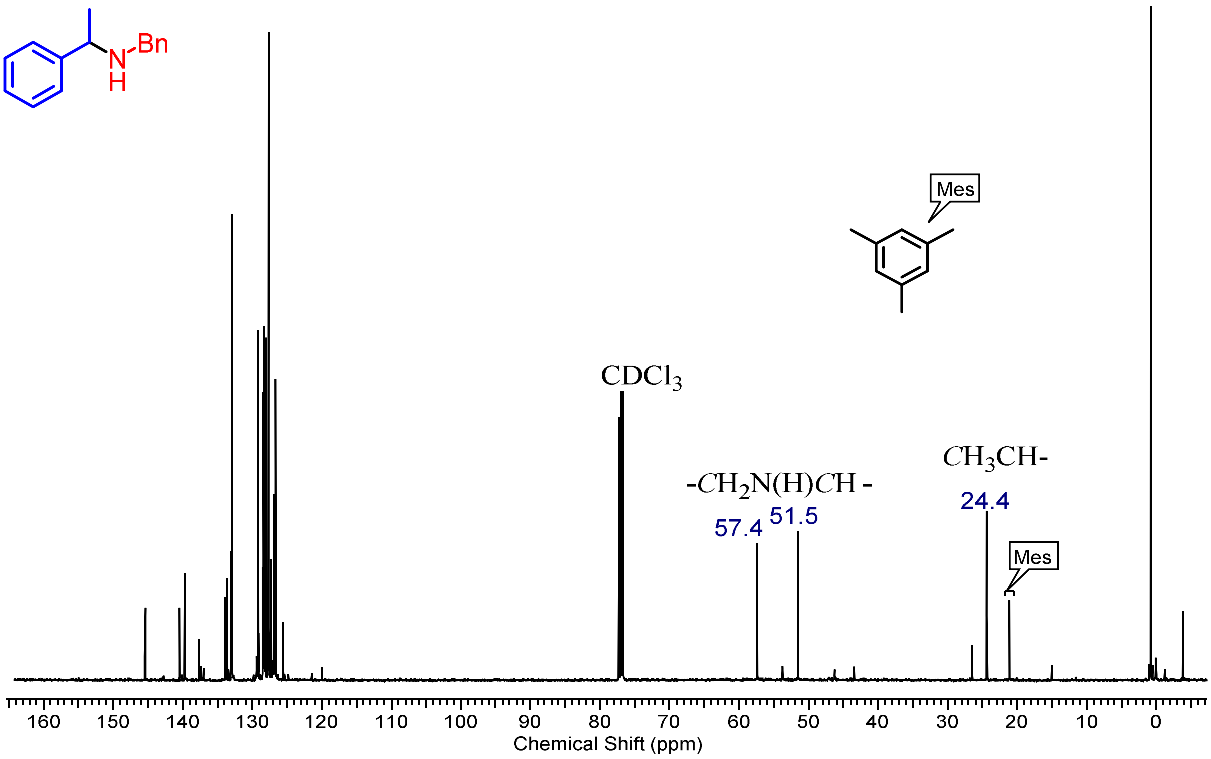

N
H
Bn
Mes
CDCl3
-CH2N(H)CH -
57.4
51.5
CH3CH-
24.4
Mes
160 150 140 130 120 110 100 90 80 70 60 50 40 30 20 10 0
Chemical Shift (ppm)

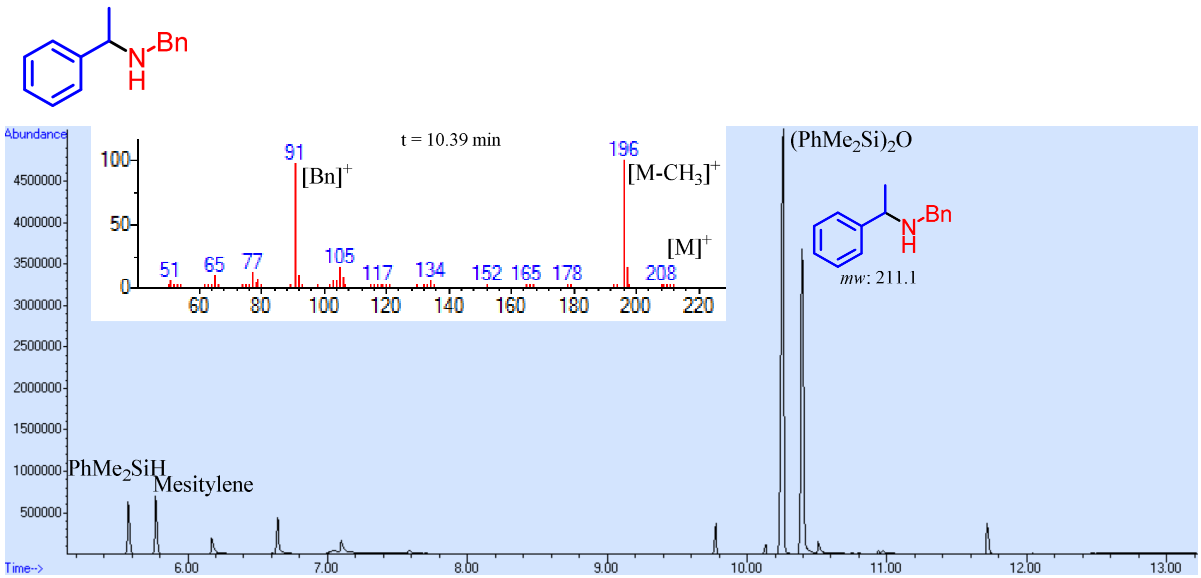

N
H
Bn
Abundance
t = 10.39 min
100
50
0
51
65
77
91
[Bn]+
105
117
134
152
165
178
196
[M-CH3]+
[M]+
208
60 80 100 120 140 160 180 200 220
(PhMe2Si)2O
N
H
Bn
mw: 211.1
4500000
4000000
3500000
3000000
2500000
2000000
1500000
1000000
500000
PhMe2SiH
Mesitylene
Time-->
6.00 7.00 8.00 9.00 10.00 11.00 12.00 13.00

N-benzylpropan-2-amine (1k)

In situ – ^{1}H-NMR spectra

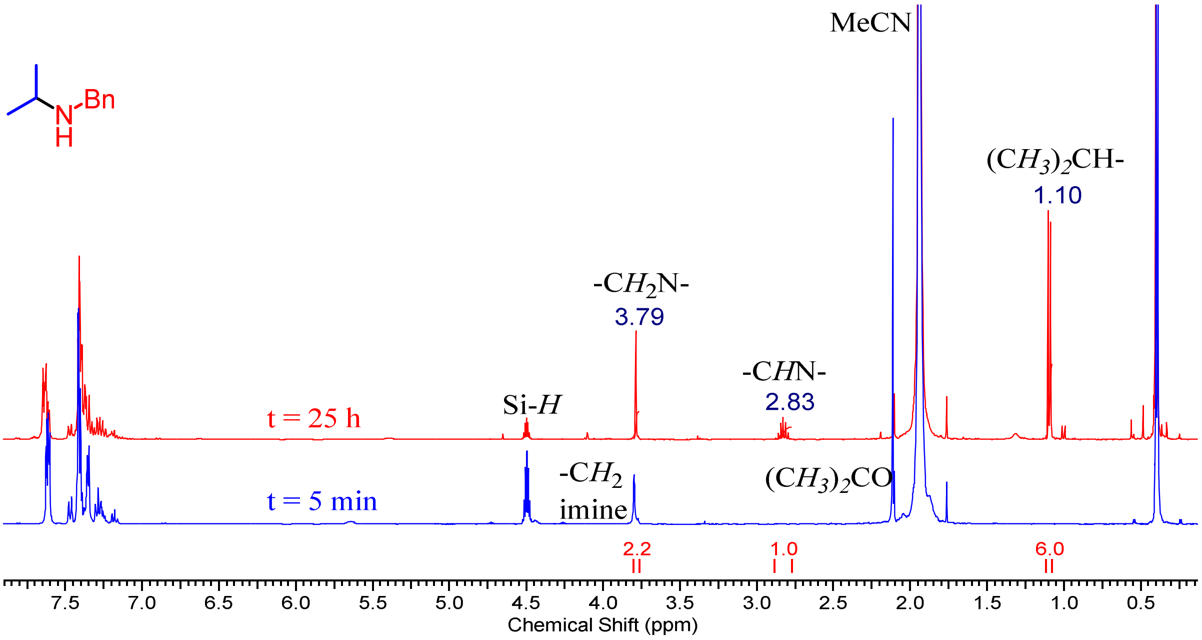

Crude - ^{1}H and ^{13}C{^{1}H}-NMR spectra and GC-MS analysis

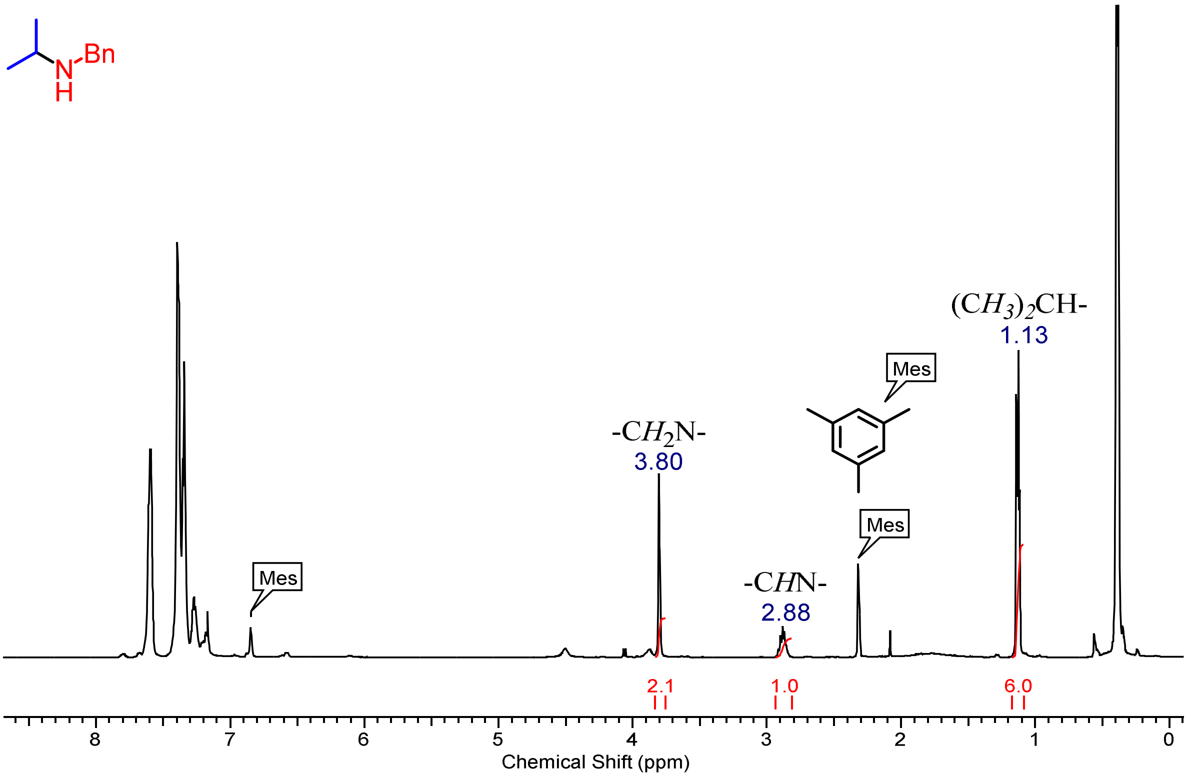

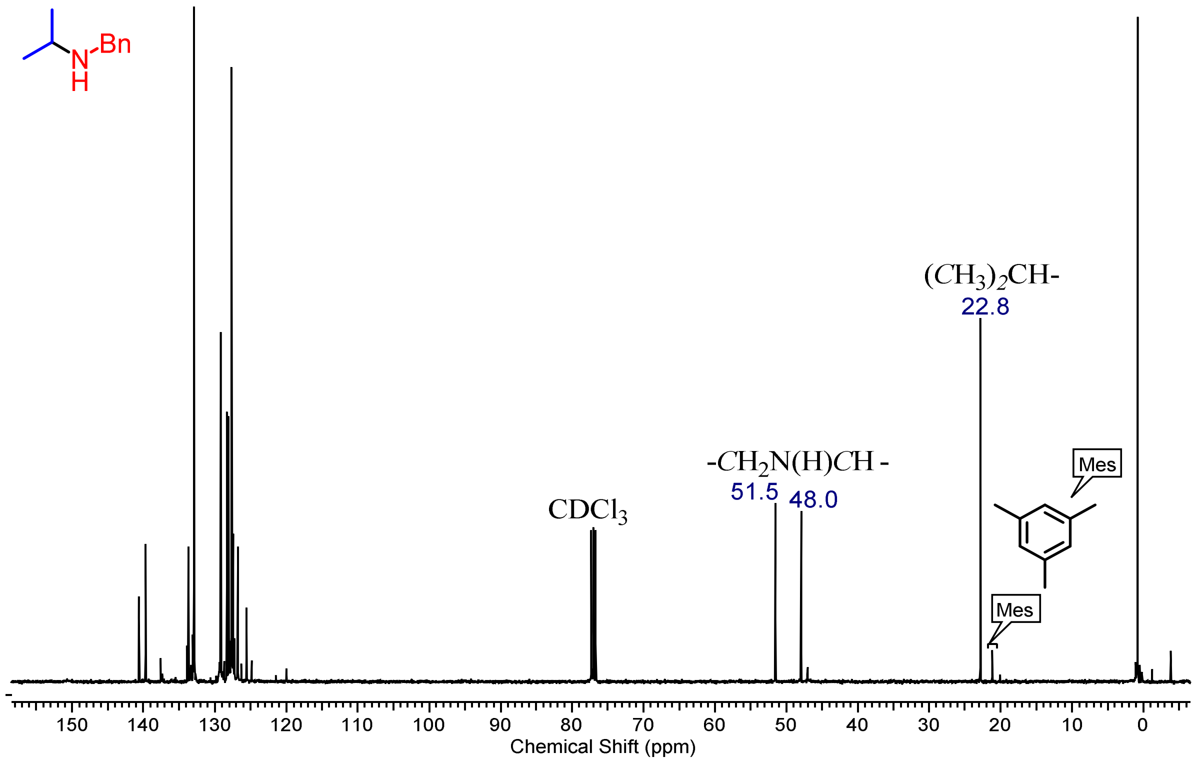

Bn
N
H
(CH3)2CH-
22.8
-CH2N(H)CH -
51.5
48.0
CDCl3
Mes
Mes
150
140
130
120
110
100
90
80
70
60
50
40
30
20
10
0
Chemical Shift (ppm)

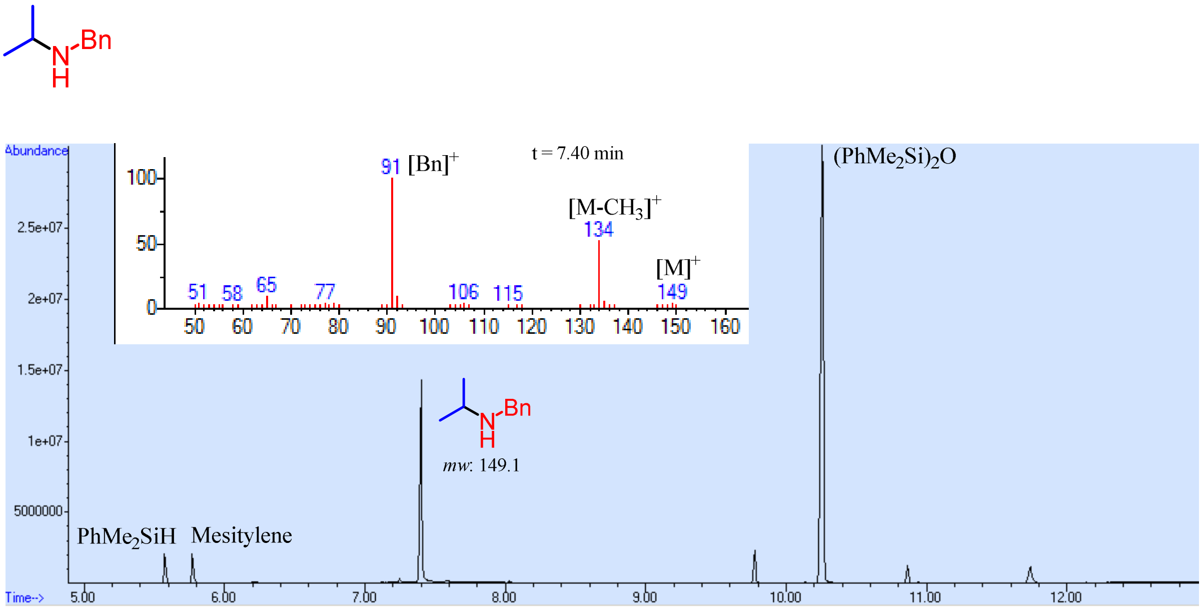

Bn
N
H
Abundance
2.5e+07
2e+07
1.5e+07
1e+07
5000000
Time-->
5.00
6.00
7.00
8.00
9.00
10.00
11.00
12.00
PhMe2SiH
Mesitylene
(PhMe2Si)2O
mw: 149.1
t = 7.40 min
91 [Bn]+
[M-CH3]+
134
[M]+
149
51
58
65
77
106
115
100
50
0

4-(1-(cyclohex-1-en-1-yl)ethyl)morpholine (1l)

***In situ* – ^{1}H-NMR spectra**

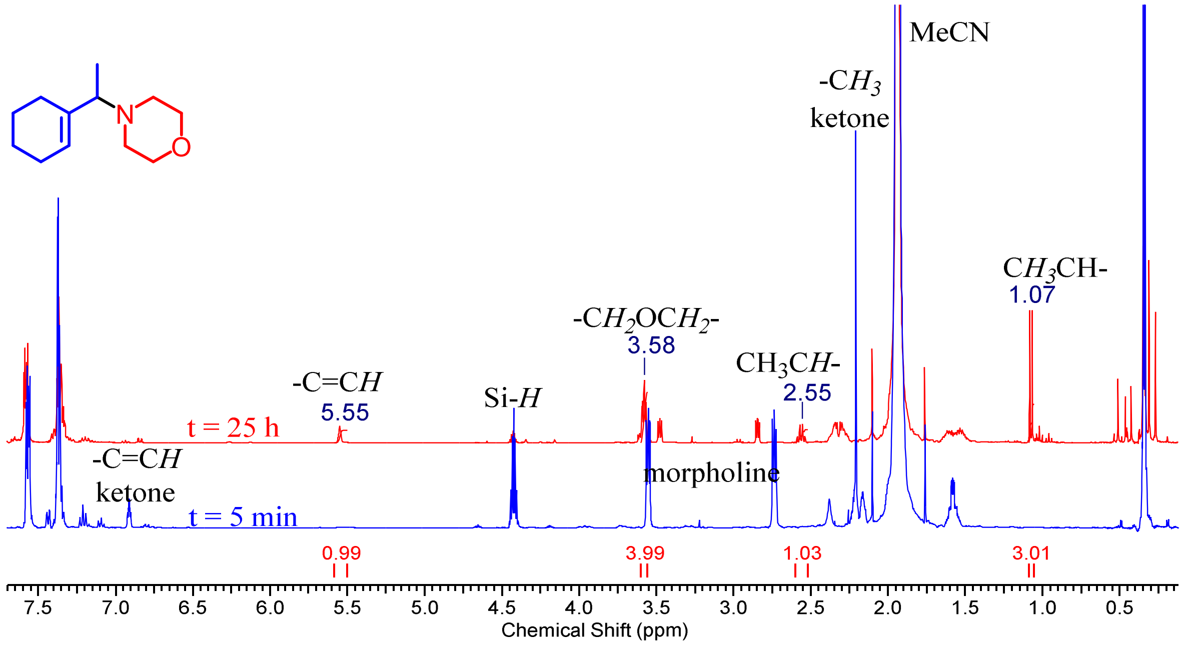

Crude - ^{1}H and ^{13}C{^{1}H}-NMR spectra and GC-MS analysis

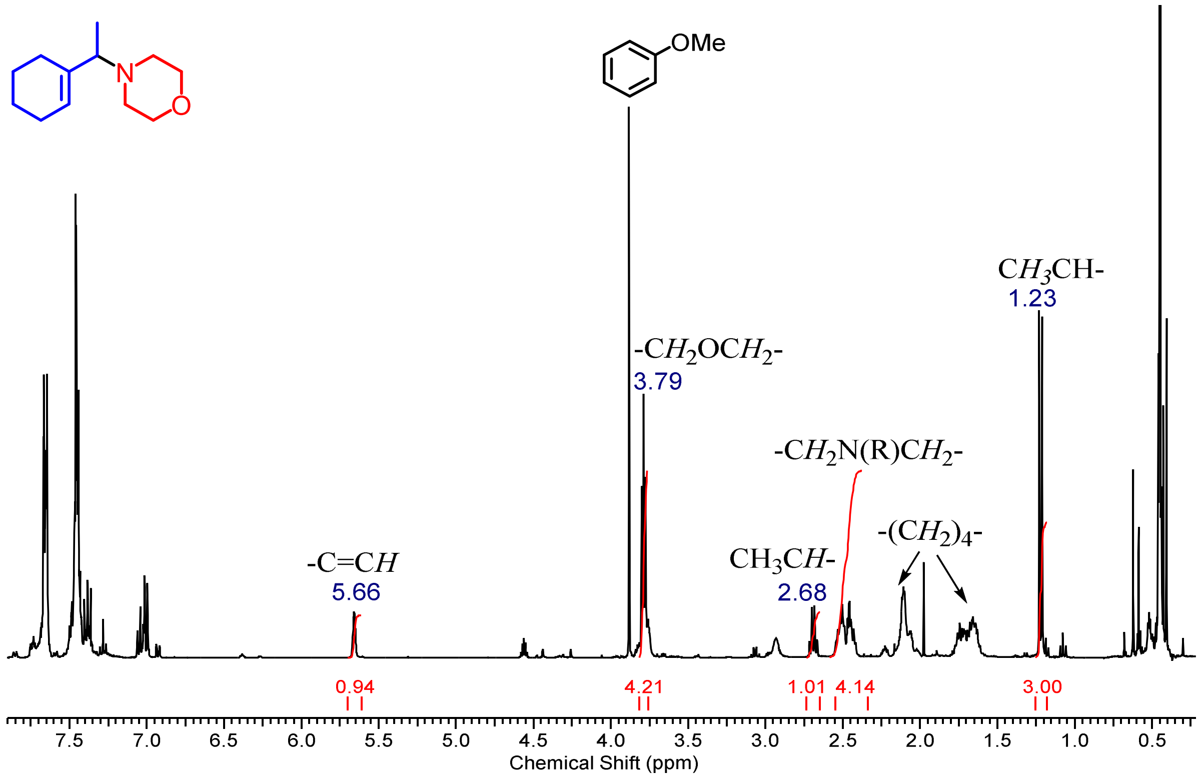

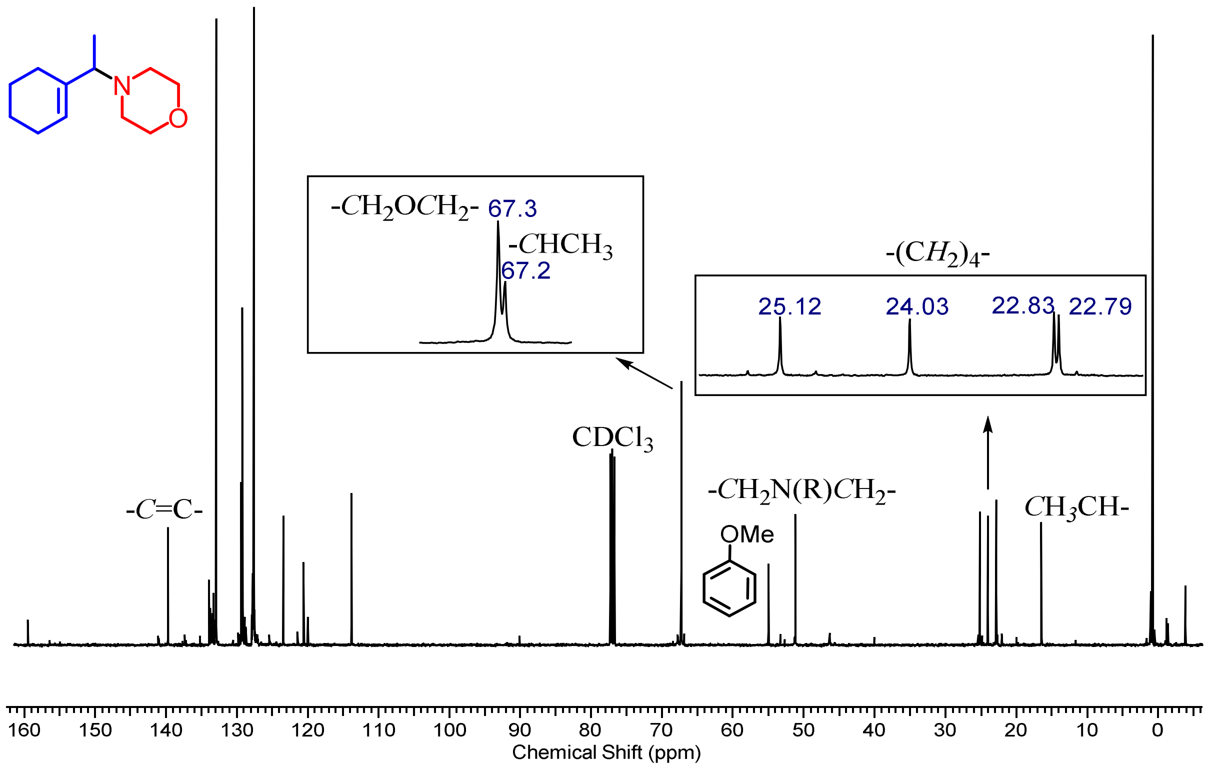
-CH_2OCH_2- 67.3
-$CHCH_3$
67.2
-$(CH_2)_4$-
25.12
24.03
22.83
22.79
CDCl$_3$
-C=C-
-$CH_2N(R)CH_2$-
OMe
CH_3CH-
160 150 140 130 120 110 100 90 80 70 60 50 40 30 20 10 0
Chemical Shift (ppm)

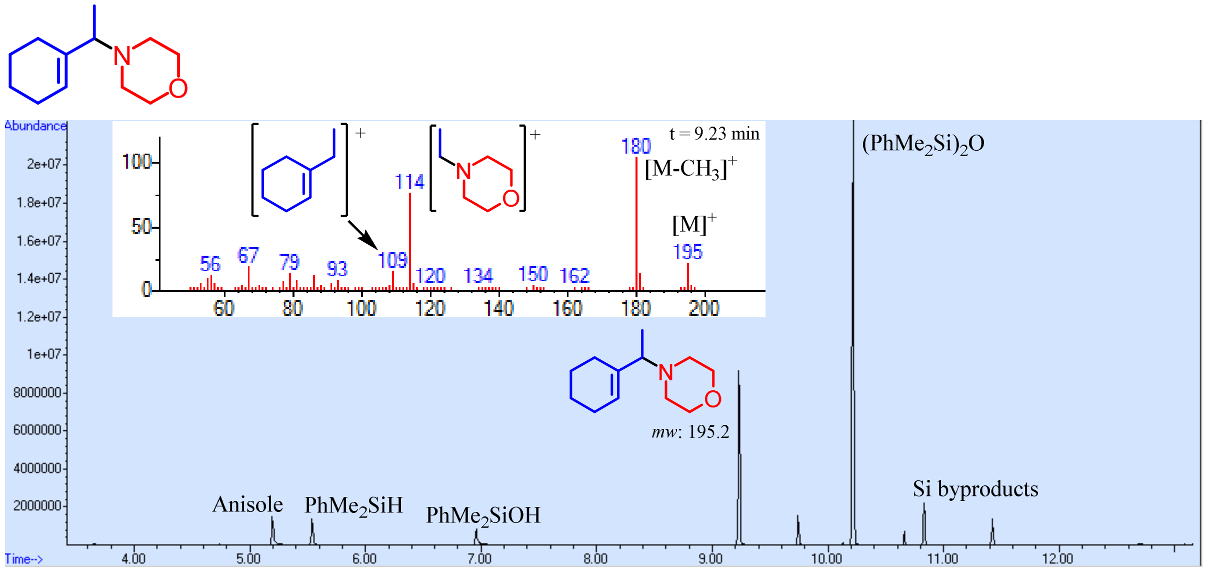
Abundance
t = 9.23 min
180
[M-CH$_3$]$^+$
114
[M]$^+$
195
56 67 79 93 109 120 134 150 162
(PhMe$_2$Si)$_2$O
mw: 195.2
Anisole
PhMe$_2$SiH
PhMe$_2$SiOH
Si byproducts
Time-->
4.00 5.00 6.00 7.00 8.00 9.00 10.00 11.00 12.00

N-benzyl-1-(m-tolyl)methanamine (1m)

In situ – ^{1}H-NMR spectra

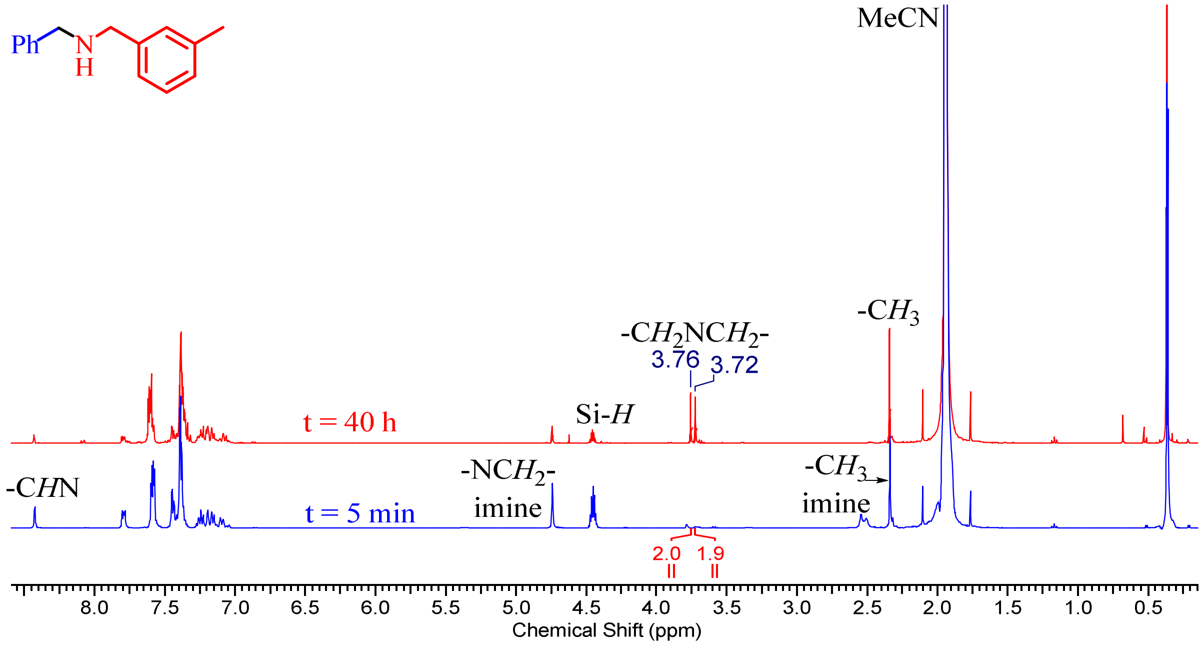

Crude - ^{1}H and ^{13}C{^{1}H}-NMR spectra and GC-MS analysis

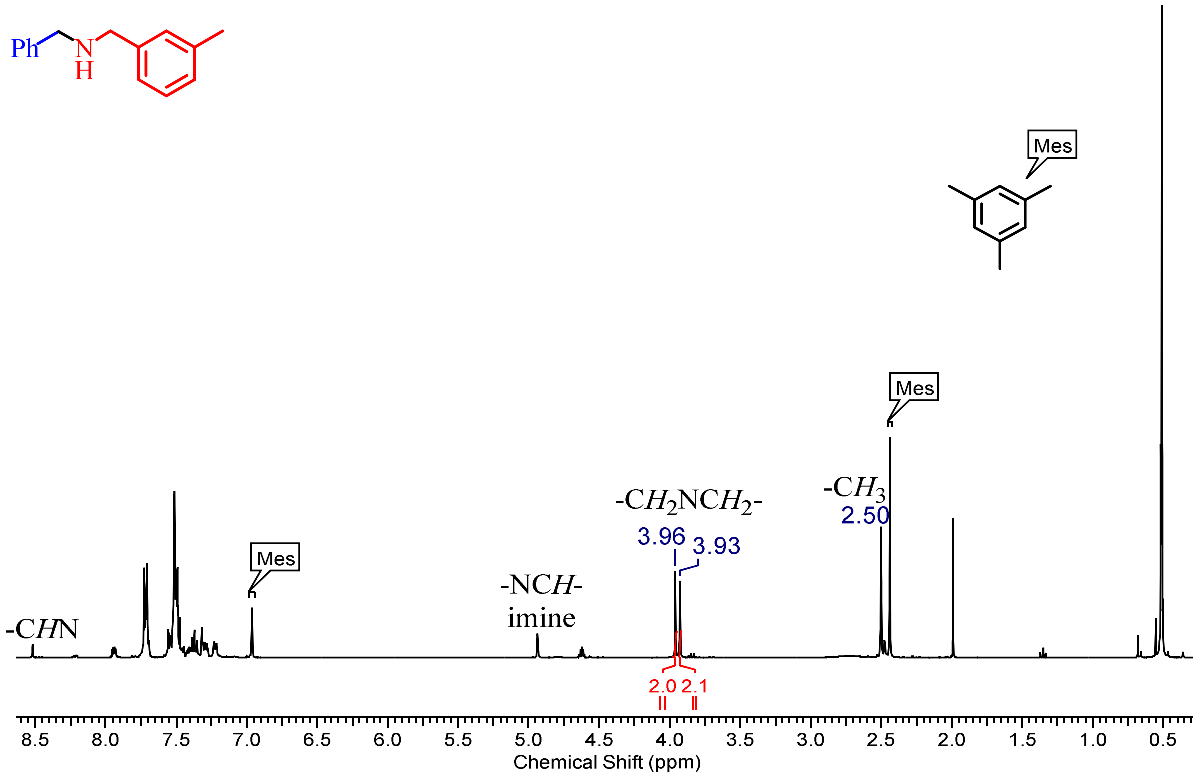

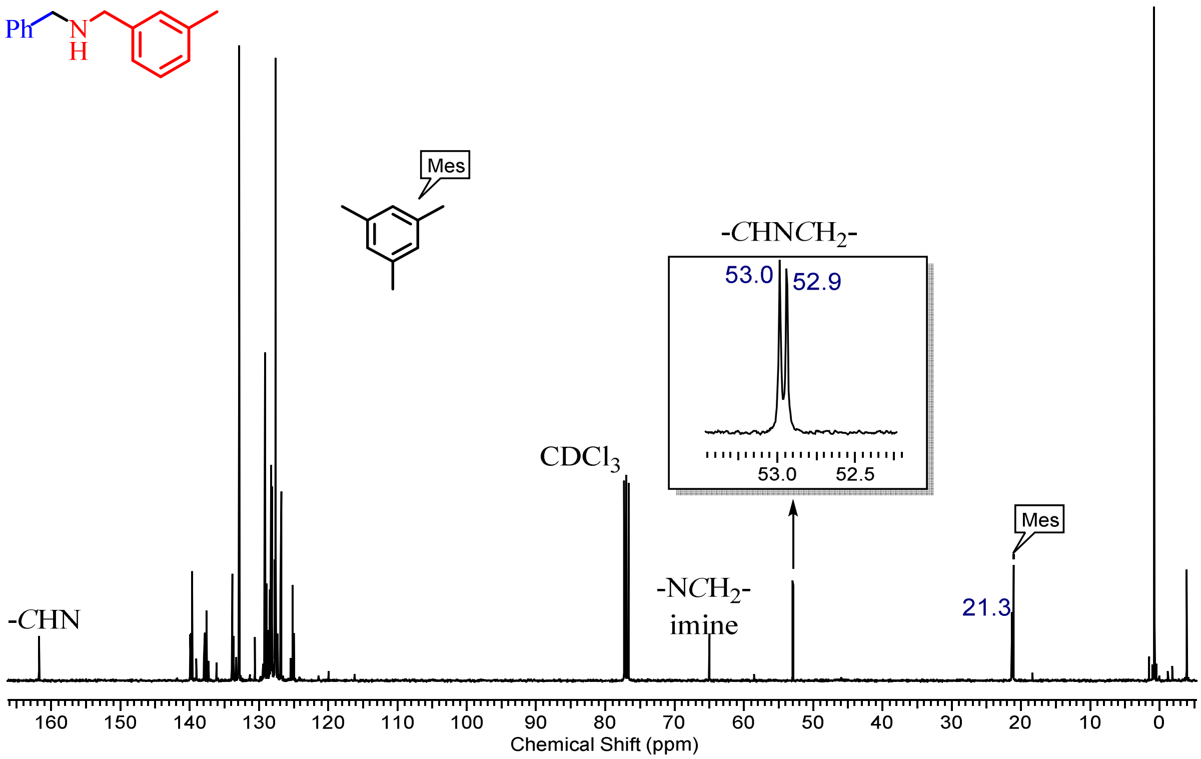
Ph
N
H
Mes
-CHNCH2-
53.0
52.9
53.0
52.5
CDCl3
-NCH2-
imine
-CHN
21.3
Mes
160 150 140 130 120 110 100 90 80 70 60 50 40 30 20 10 0
Chemical Shift (ppm)

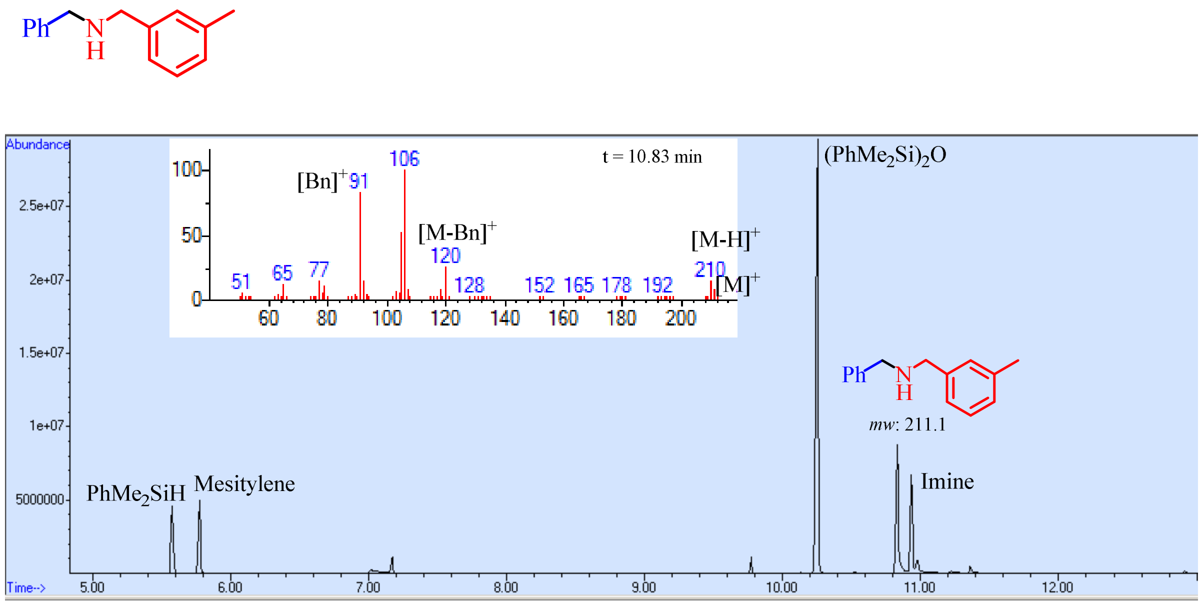
Ph
N
H
Abundance
2.5e+07
2e+07
1.5e+07
1e+07
5000000
Time-->
5.00 6.00 7.00 8.00 9.00 10.00 11.00 12.00
t = 10.83 min
100
50
0
[Bn]+ 91
106
[M-Bn]+
120
[M-H]+
210
[M]+
51 65 77 128 152 165 178 192
60 80 100 120 140 160 180 200
(PhMe2Si)2O
mw: 211.1
Imine
PhMe2SiH
Mesitylene

N-benzyl-1-(4-fluorophenyl)methanamine (1n)

***In situ* – ^{1}H-NMR spectra**

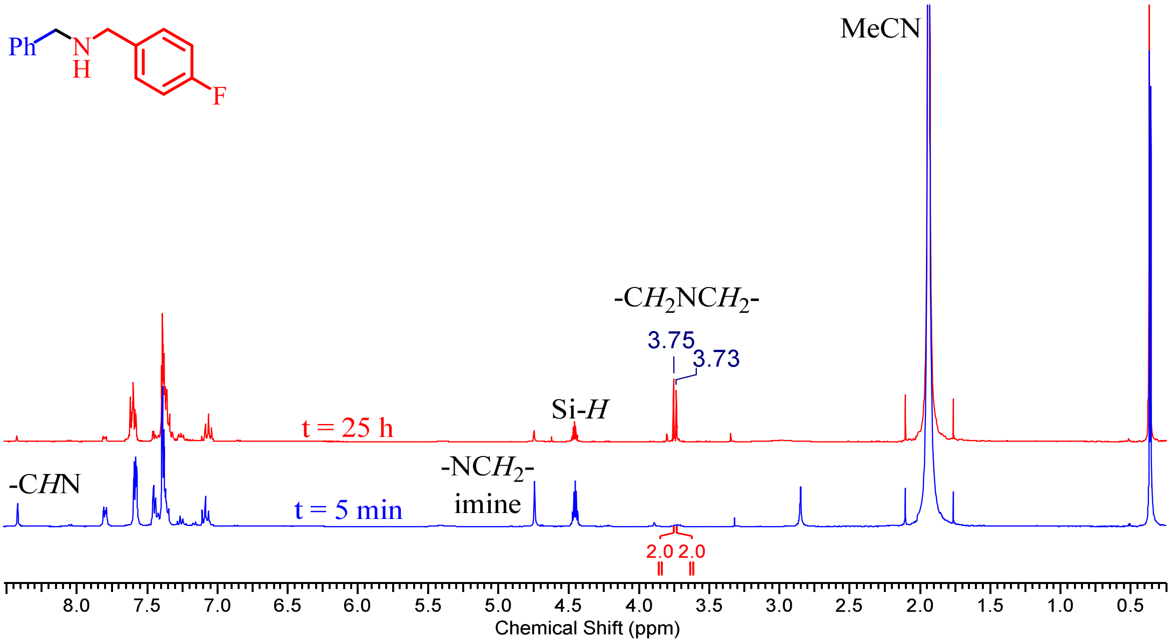

Crude - ^{1}H and ^{13}C{^{1}H}-NMR spectra and GC-MS analysis

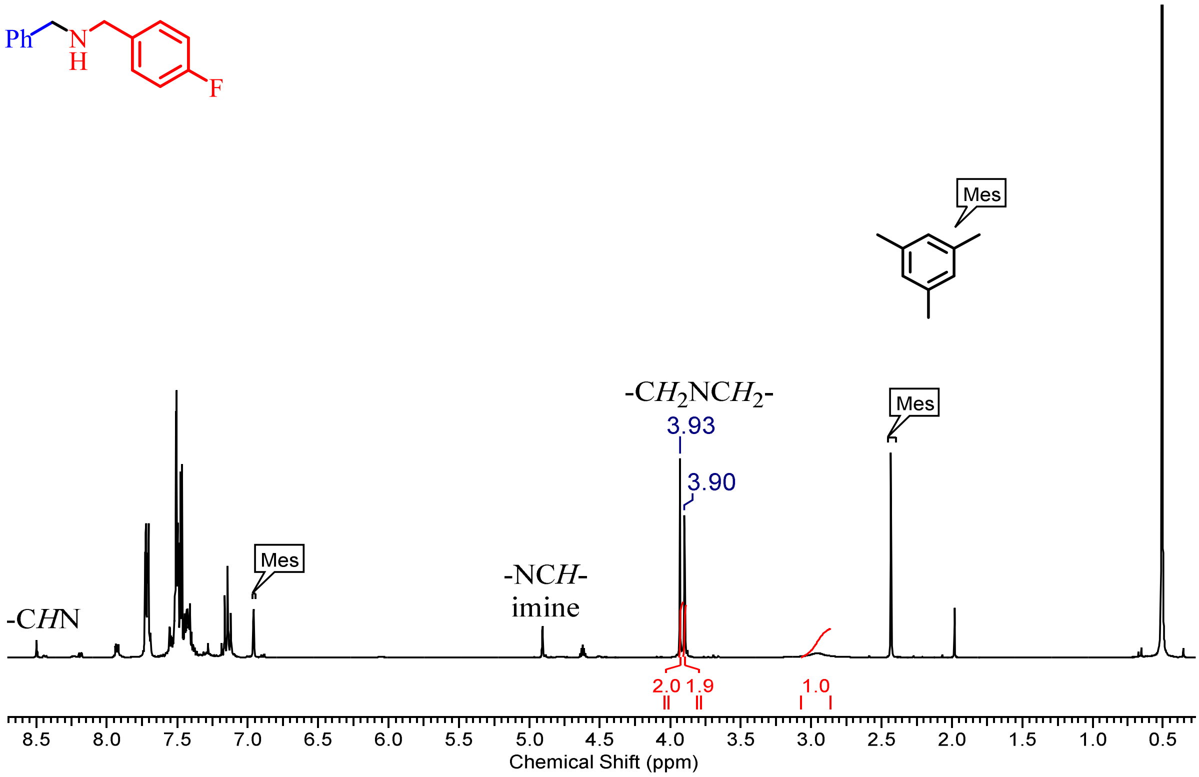

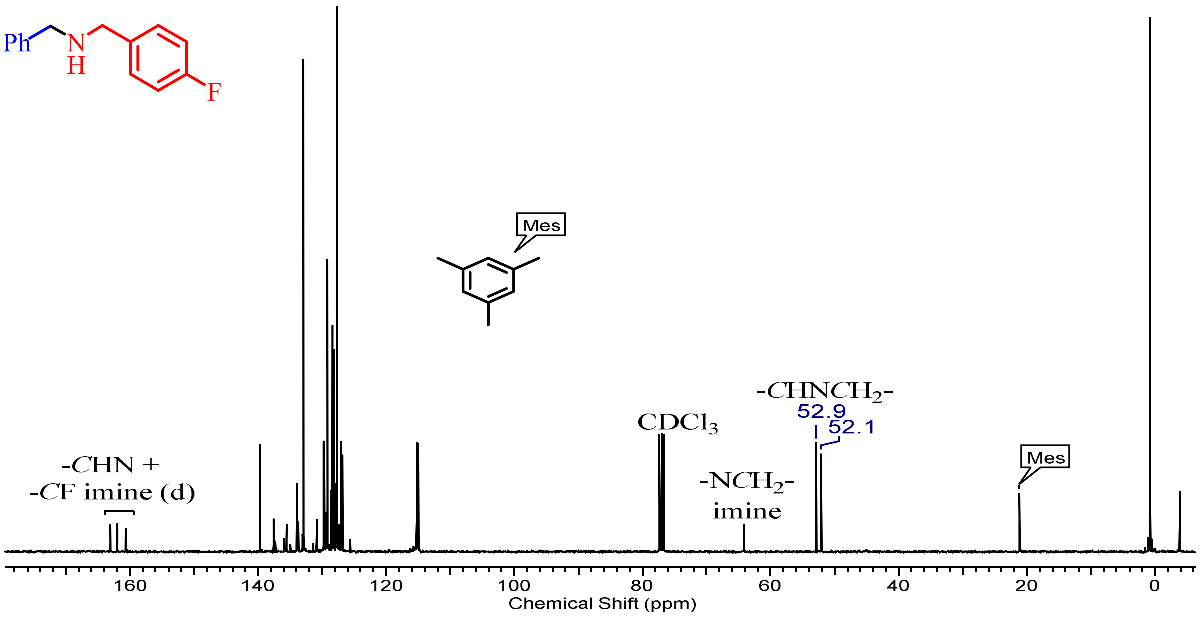
Ph
N
H
F
Mes
-CHNCH2-
52.9
52.1
CDCl3
-NCH2-
imine
Mes
-CHN +
-CF imine (d)
160
140
120
100
80
60
40
20
0
Chemical Shift (ppm)

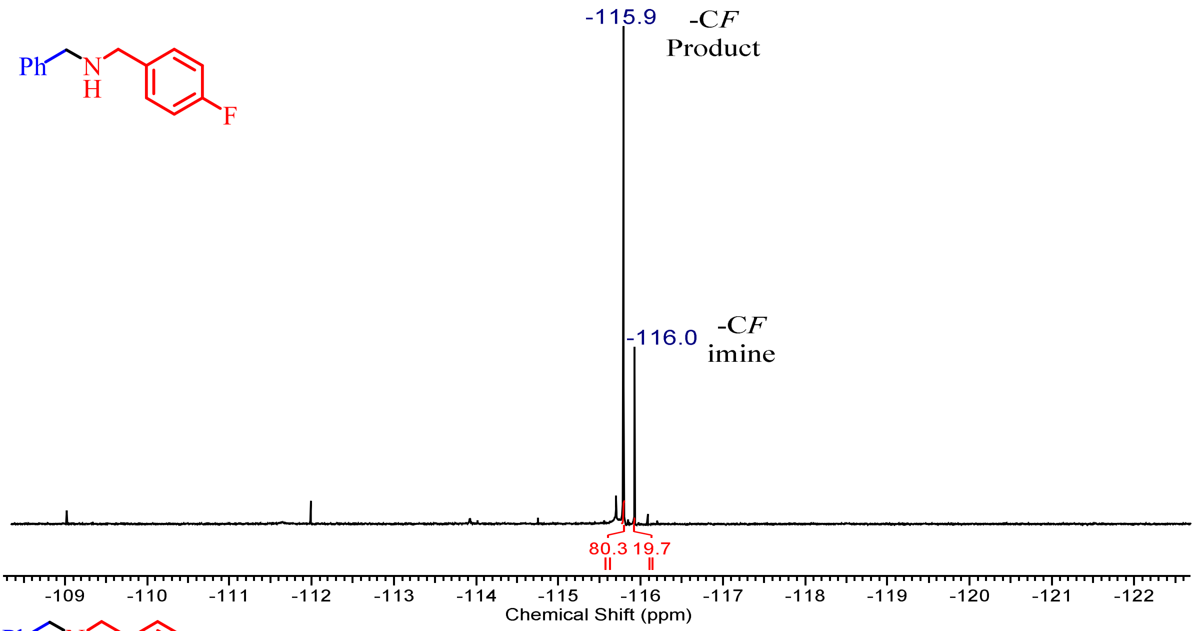
-115.9
-CF
Product
Ph
N
H
F
-116.0
-CF
imine
80.3
19.7
-109
-110
-111
-112
-113
-114
-115
-116
-117
-118
-119
-120
-121
-122
Chemical Shift (ppm)

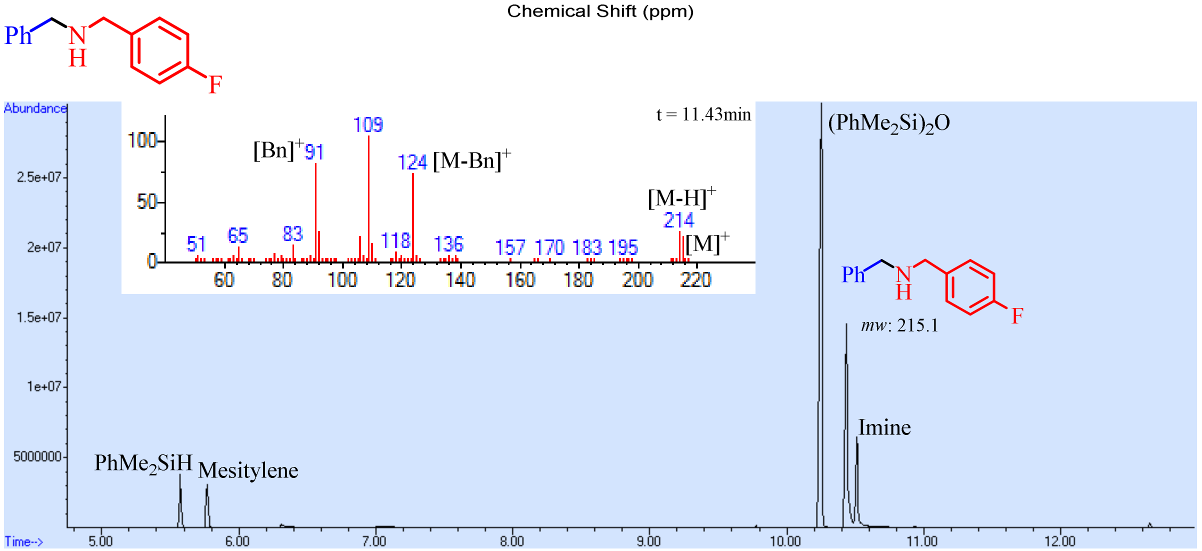
Ph
N
H
F
Abundance
t = 11.43min
109
[Bn]+ 91
124 [M-Bn]+
[M-H]+
214
[M]+
51
65
83
118
136
157 170 183 195
(PhMe2Si)2O
mw: 215.1
Imine
PhMe2SiH
Mesitylene
Time-->

N-benzylbutan-1-amine (1o)

In situ **– ^{1}H-NMR spectra**

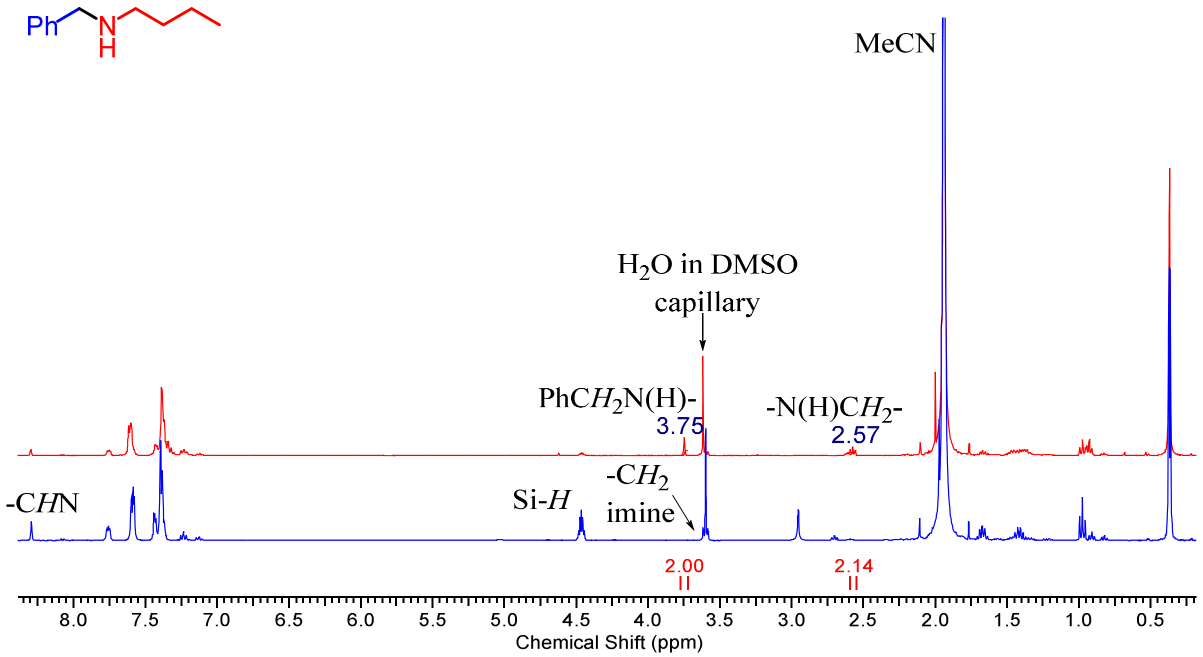

Crude - ^{1}H and ^{13}C{^{1}H} NMR spectra and GC-MS analysis

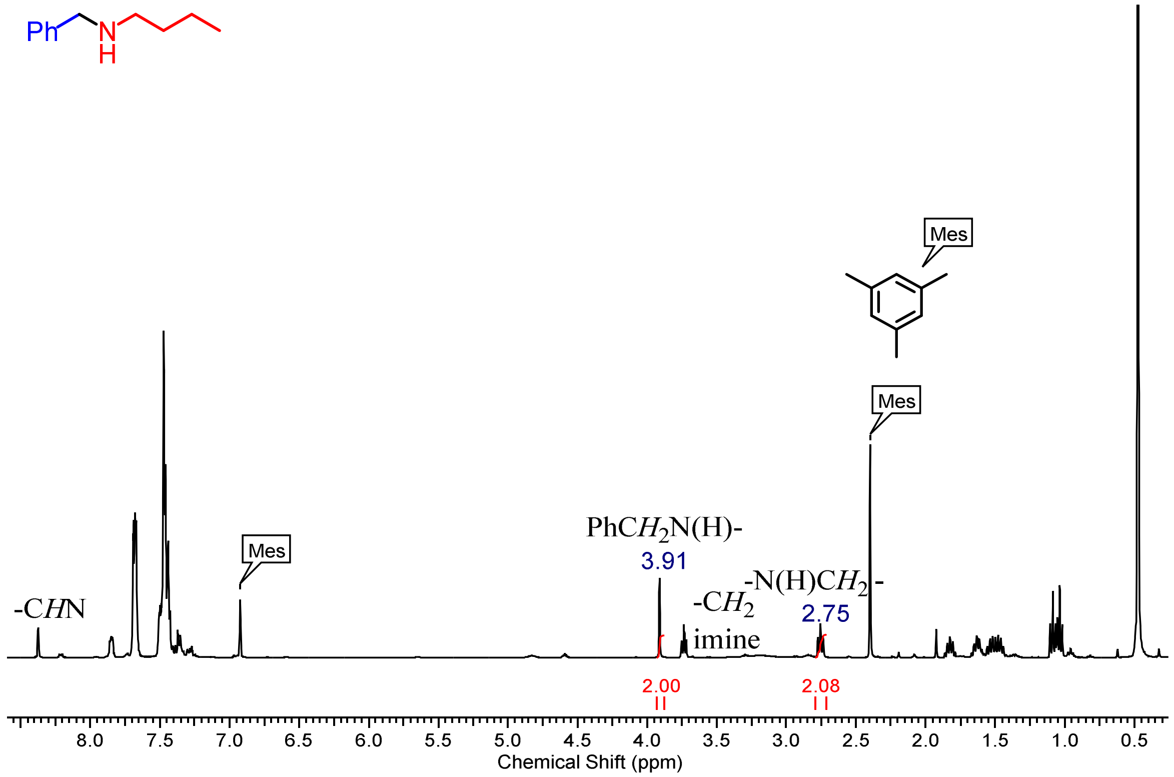

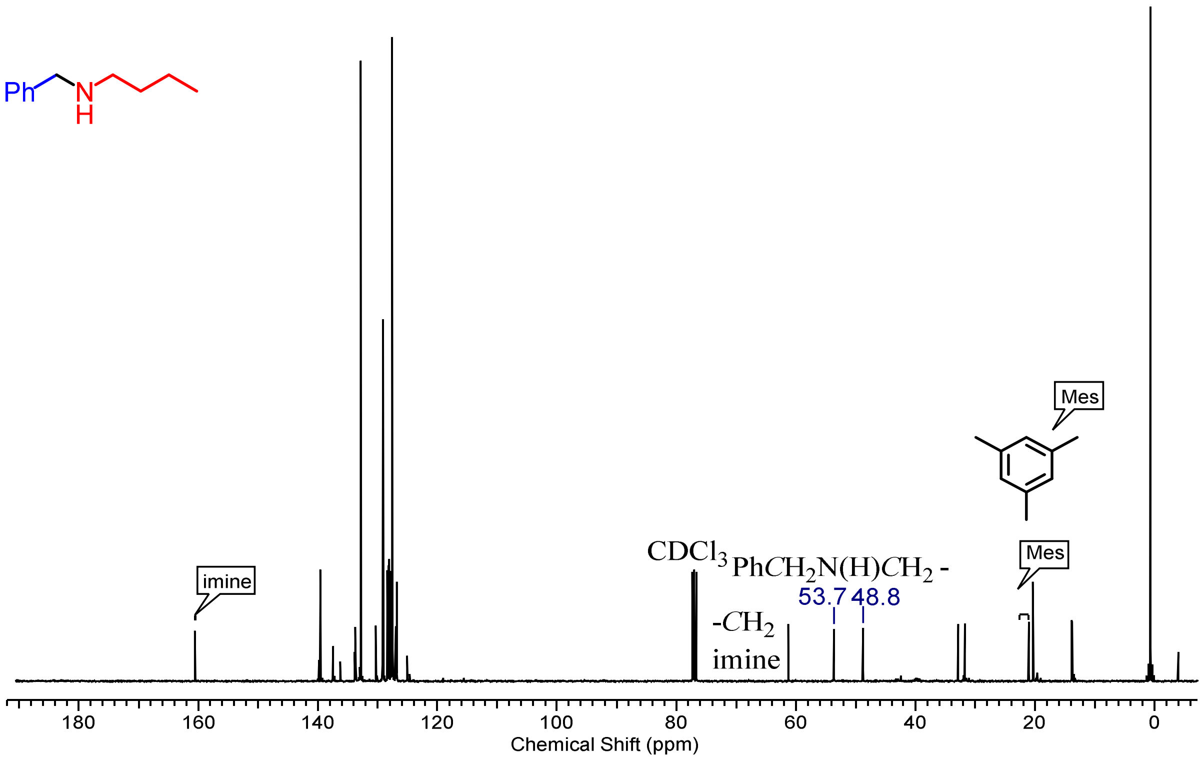

Ph
N
H
imine
CDCl3
PhCH2N(H)CH2 -
53.7 48.8
-CH2
imine
Mes
Mes
Chemical Shift (ppm)

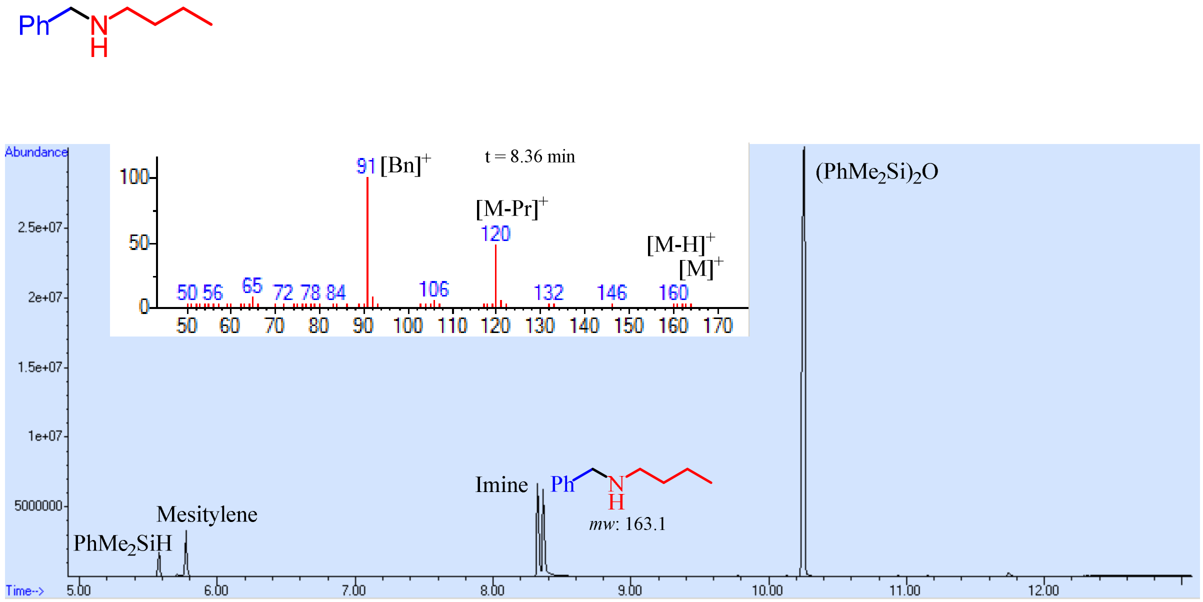

Ph
N
H
Abundance
t = 8.36 min
91 [Bn]+
[M-Pr]+
120
[M-H]+
[M]+
(PhMe2Si)2O
Mesitylene
PhMe2SiH
Imine
mw: 163.1
Time-->

***N*-benzylpropan-2-amine (1p)**

***In situ* – ¹H-NMR spectra**

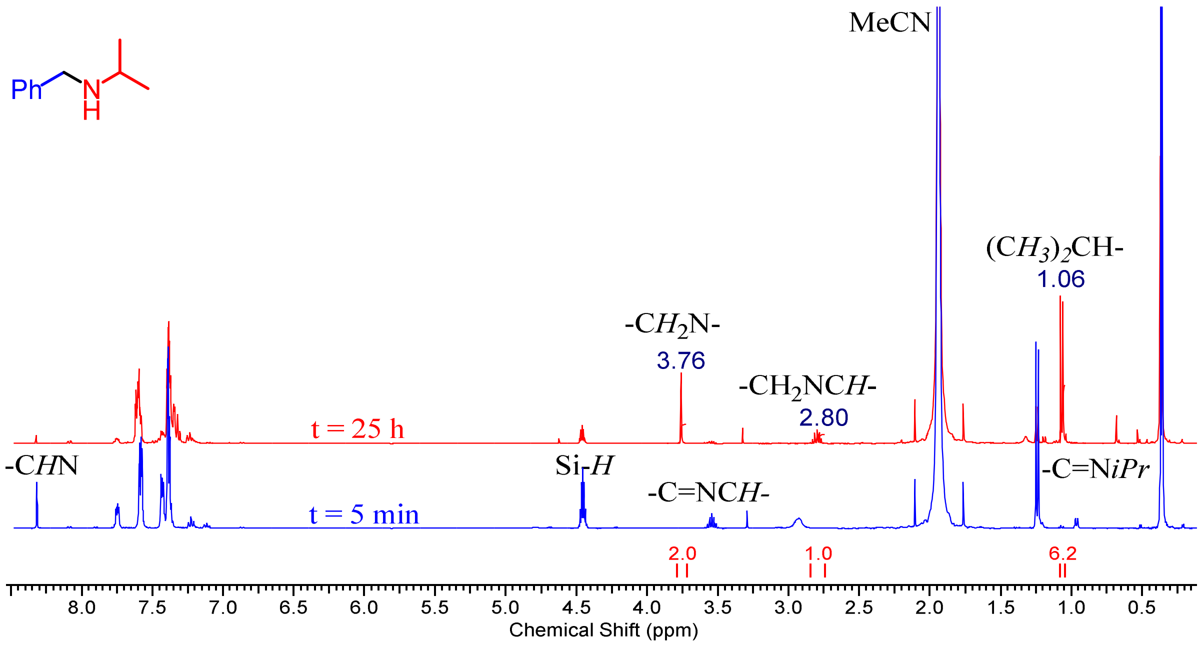

Crude - ¹H and ¹³C{¹H}-NMR spectra and GC-MS analysis

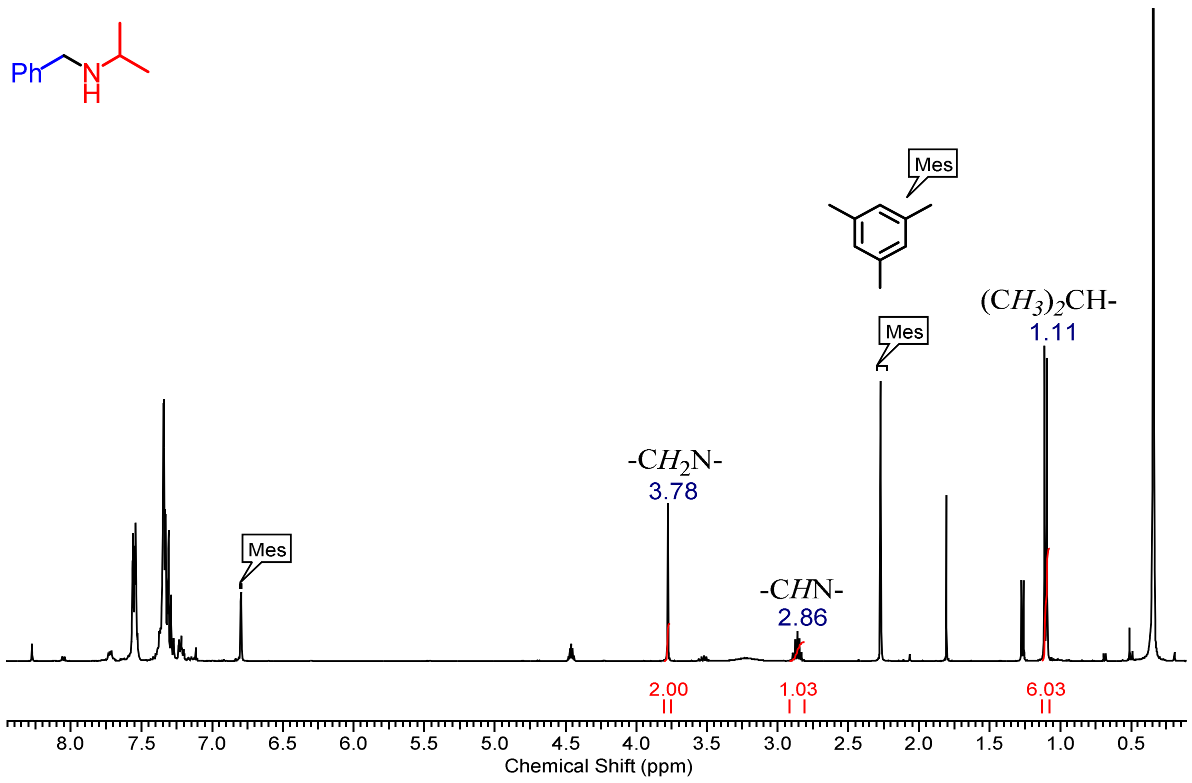

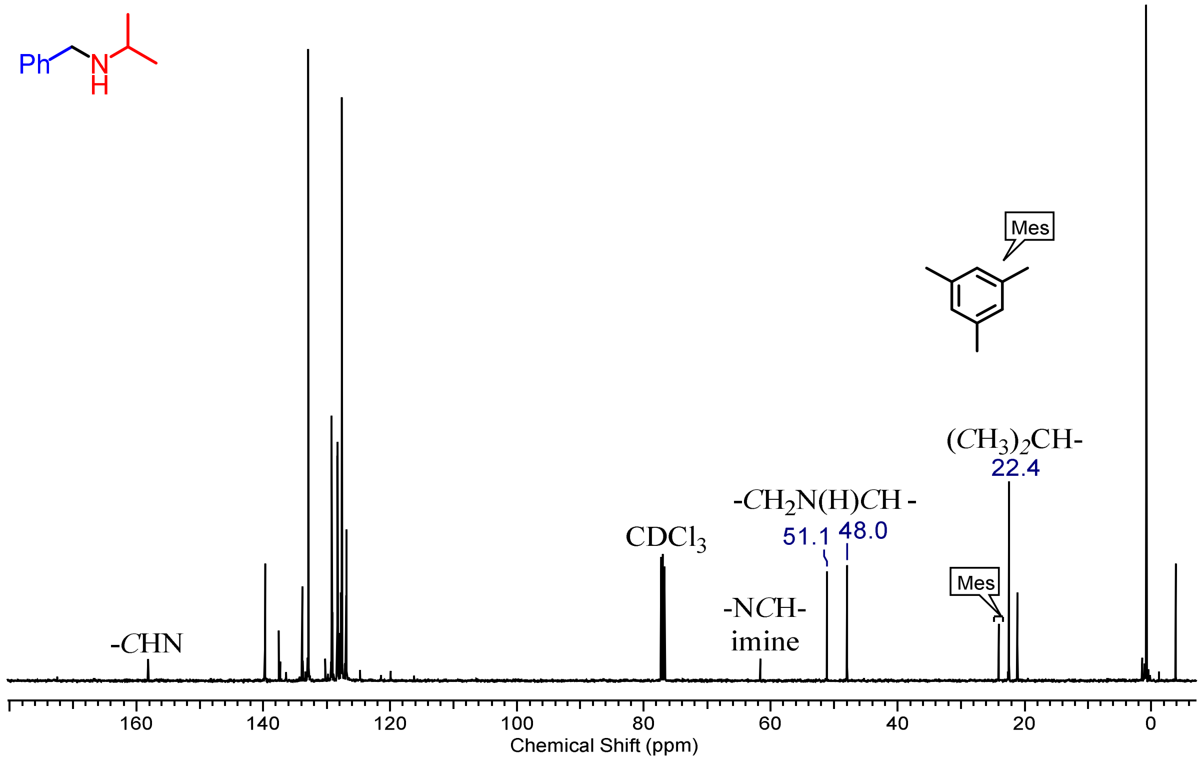
Ph
N
H
Mes
(CH3)2CH-
22.4
-CH2N(H)CH -
51.1 48.0
CDCl3
-NCH-
imine
-CHN
160 140 120 100 80 60 40 20 0
Chemical Shift (ppm)

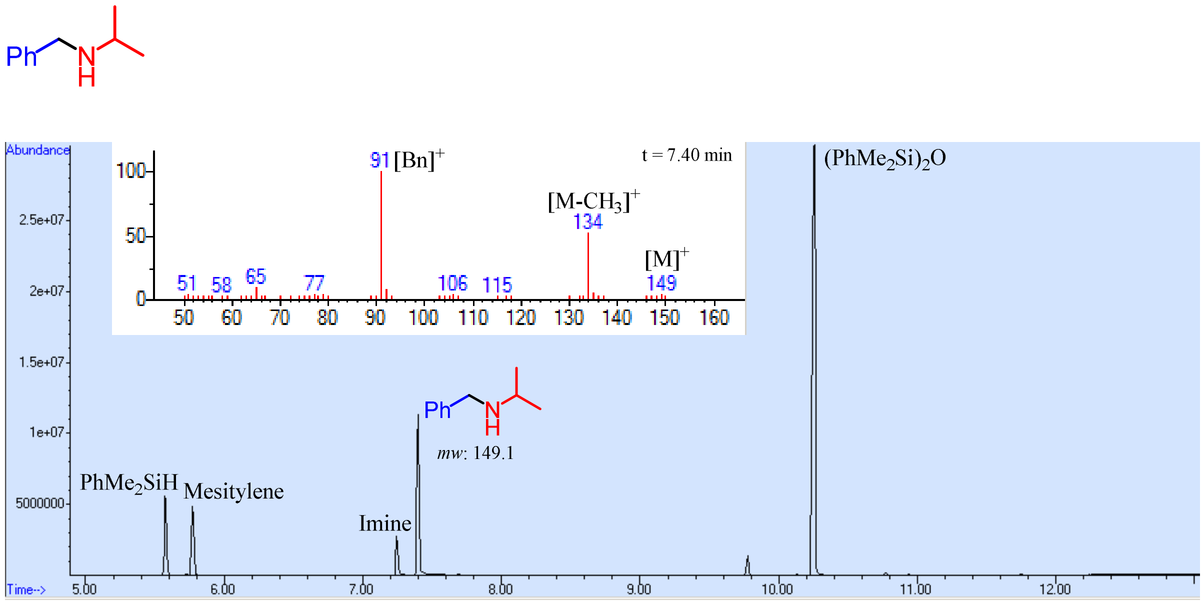
Ph
N
H
Abundance
t = 7.40 min
91 [Bn]+
[M-CH3]+
134
[M]+
149
51 58 65 77 106 115
(PhMe2Si)2O
PhMe2SiH
Mesitylene
Imine
mw: 149.1
Time-->

***N*-benzylcyclohexanamine (1q)**

***In situ* – ^{1}H-NMR spectra**

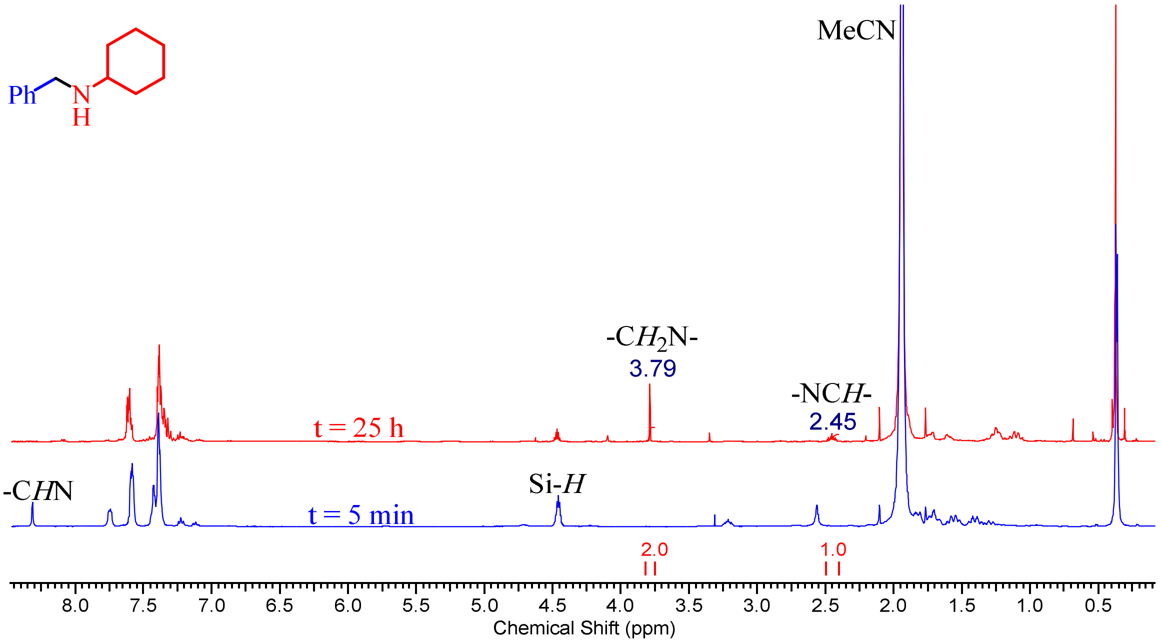

Crude - ^{1}H and ^{13}C{^{1}H}-NMR spectra and GC-MS analysis

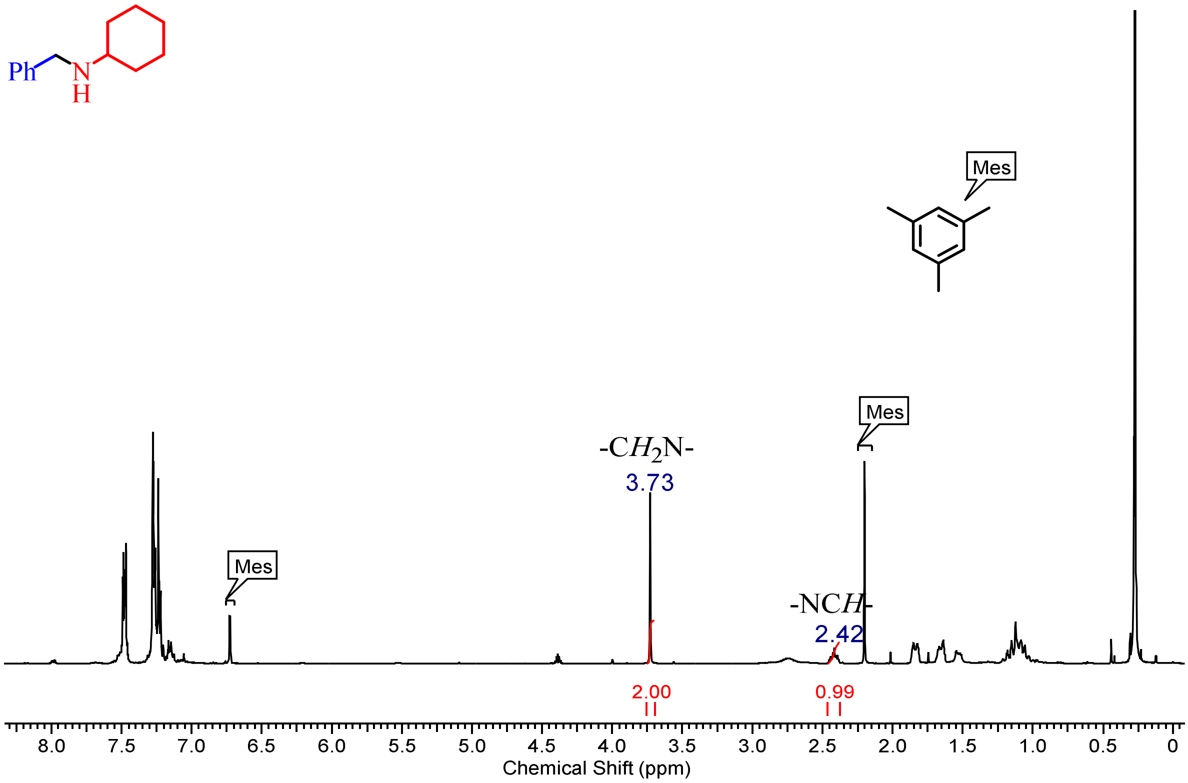

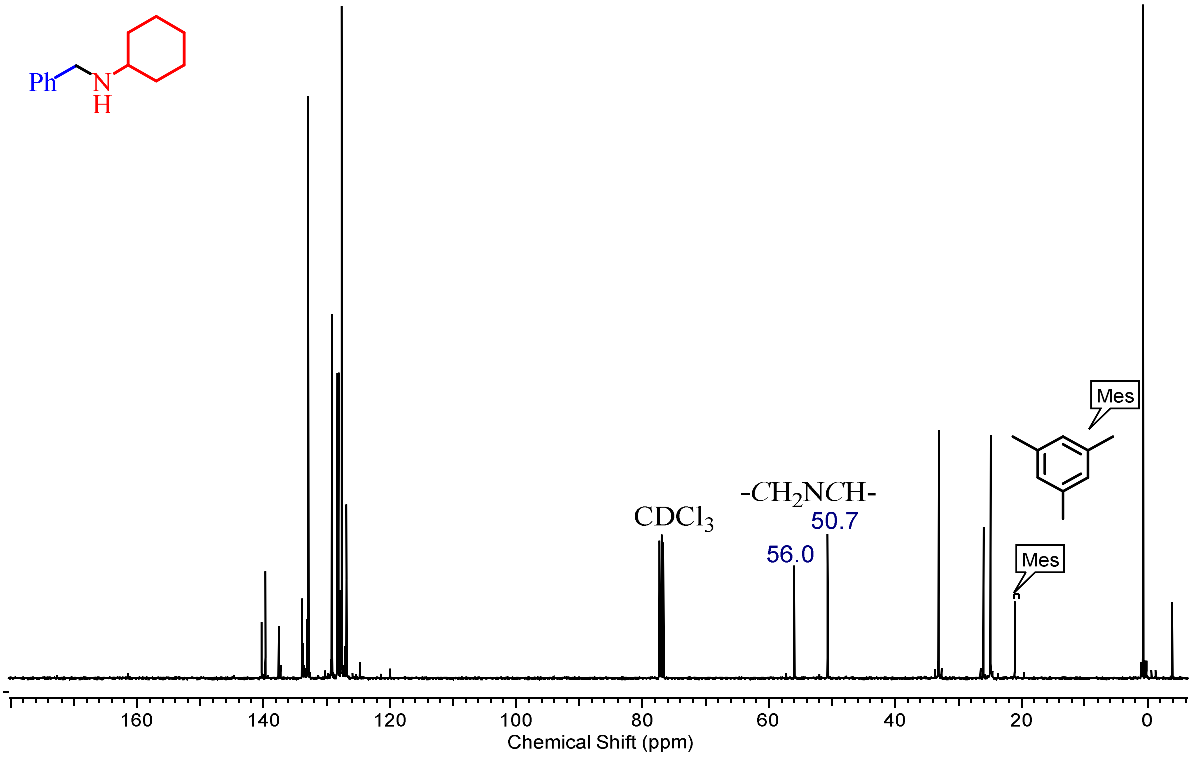
Ph
N
H
CDCl3
-CH2NCH-
50.7
56.0
Mes
Mes
160
140
120
100
80
60
40
20
0
Chemical Shift (ppm)

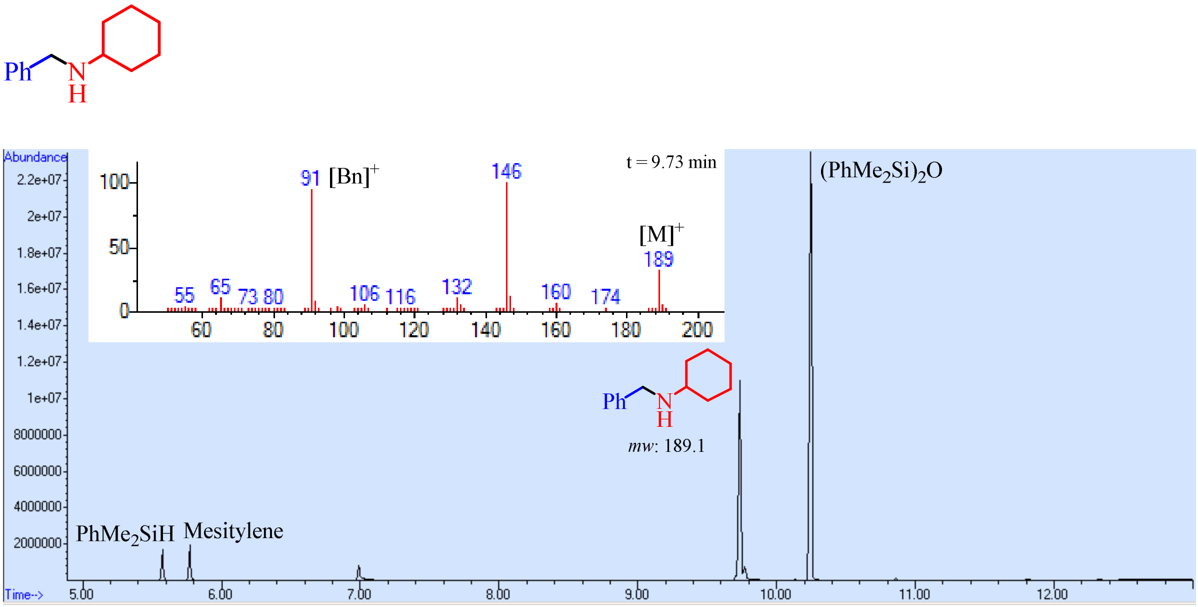
Ph
N
H
Abundance
t = 9.73 min
91 [Bn]+
146
[M]+
189
55
65
73 80
106 116
132
160
174
(PhMe2Si)2O
mw: 189.1
PhMe2SiH
Mesitylene
Time-->

***N*-benzyl-2-methylpropan-2-amine (1r)**

***In situ* – ^{1}H-NMR spectra**

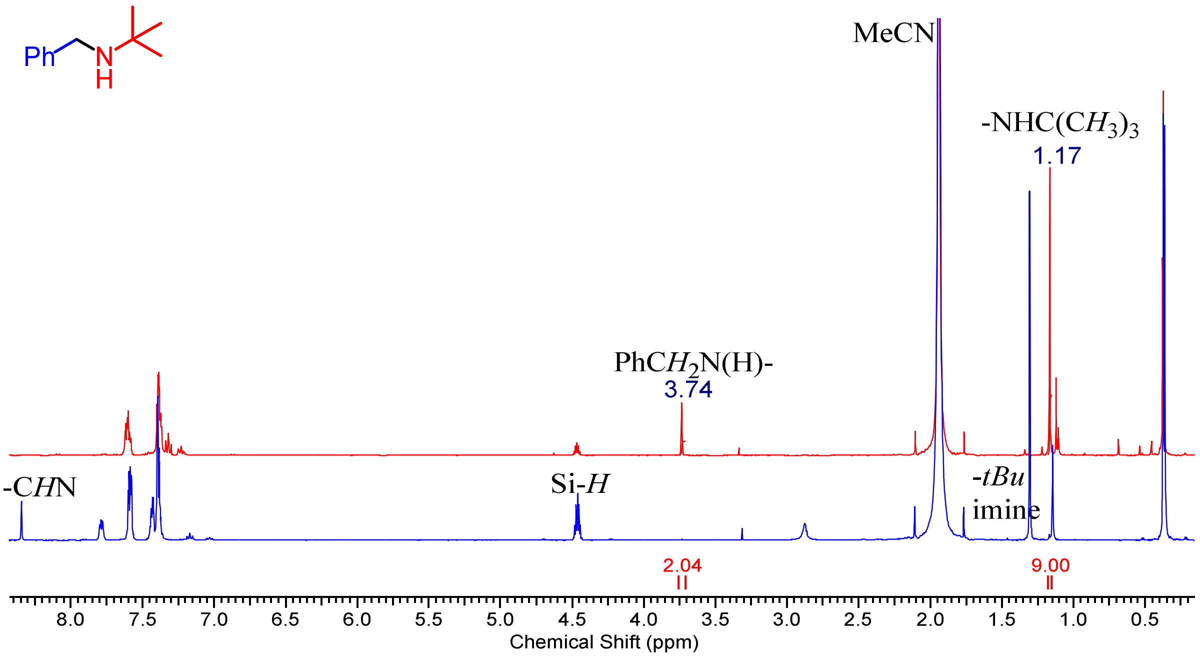

Crude - ^{1}H and ^{13}C{^{1}H} NMR spectra and GC-MS analysis

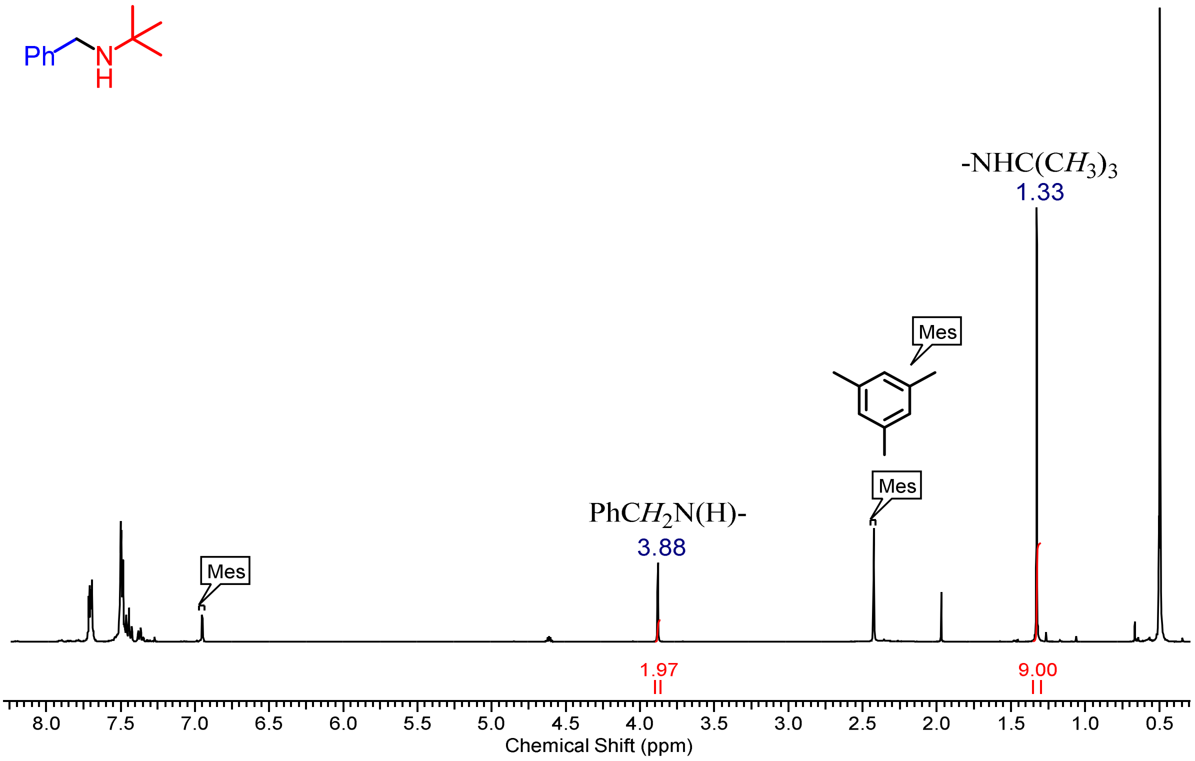

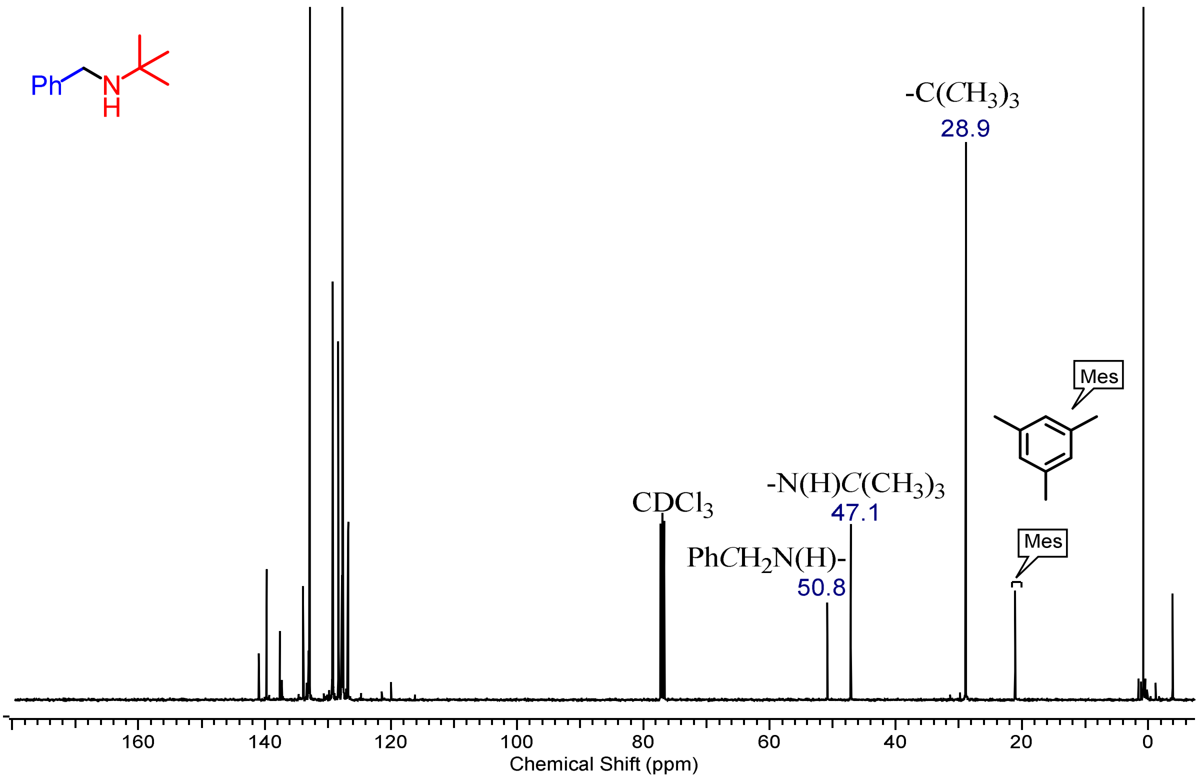
Ph
N
H
-C(CH3)3
28.9
Mes
-N(H)C(CH3)3
47.1
CDCl3
PhCH2N(H)-
50.8
Mes
160
140
120
100
80
60
40
20
0
Chemical Shift (ppm)

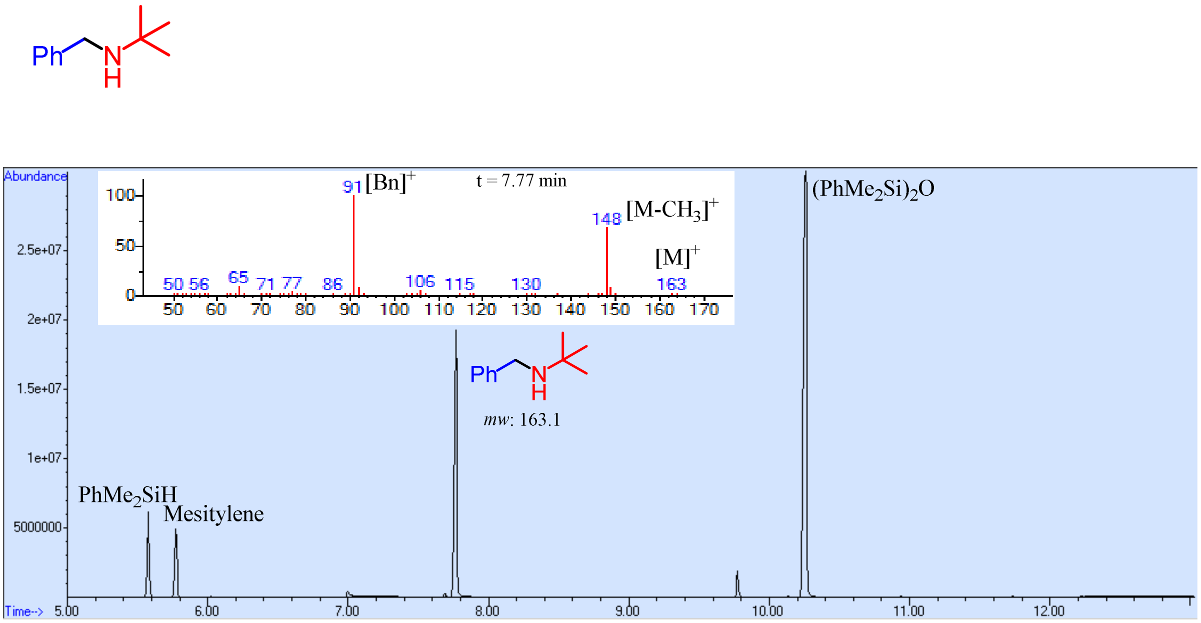
Ph
N
H
Abundance
91 [Bn]+
t = 7.77 min
148 [M-CH3]+
[M]+
163
50 56 65 71 77 86 106 115 130
(PhMe2Si)2O
mw: 163.1
PhMe2SiH
Mesitylene
Time-->

***N*-benzyl-*N*-methylpropan-2-amine (1s)**

***In situ* – ^{1}H-NMR spectra**

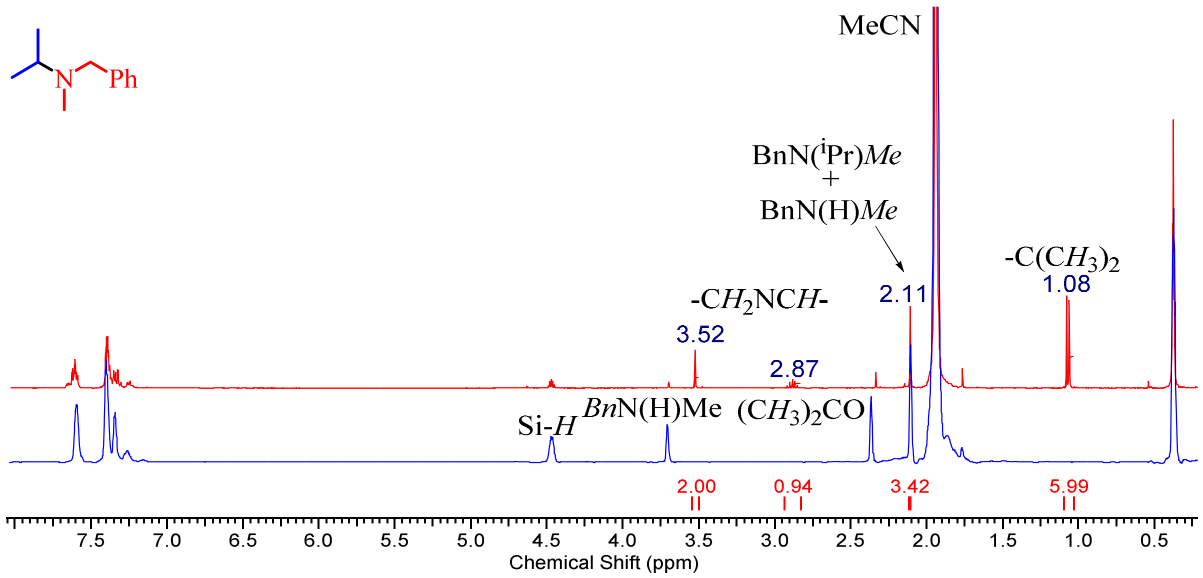

Crude - ^{1}H and ^{13}C{^{1}H}-NMR spectra and GC-MS analysis

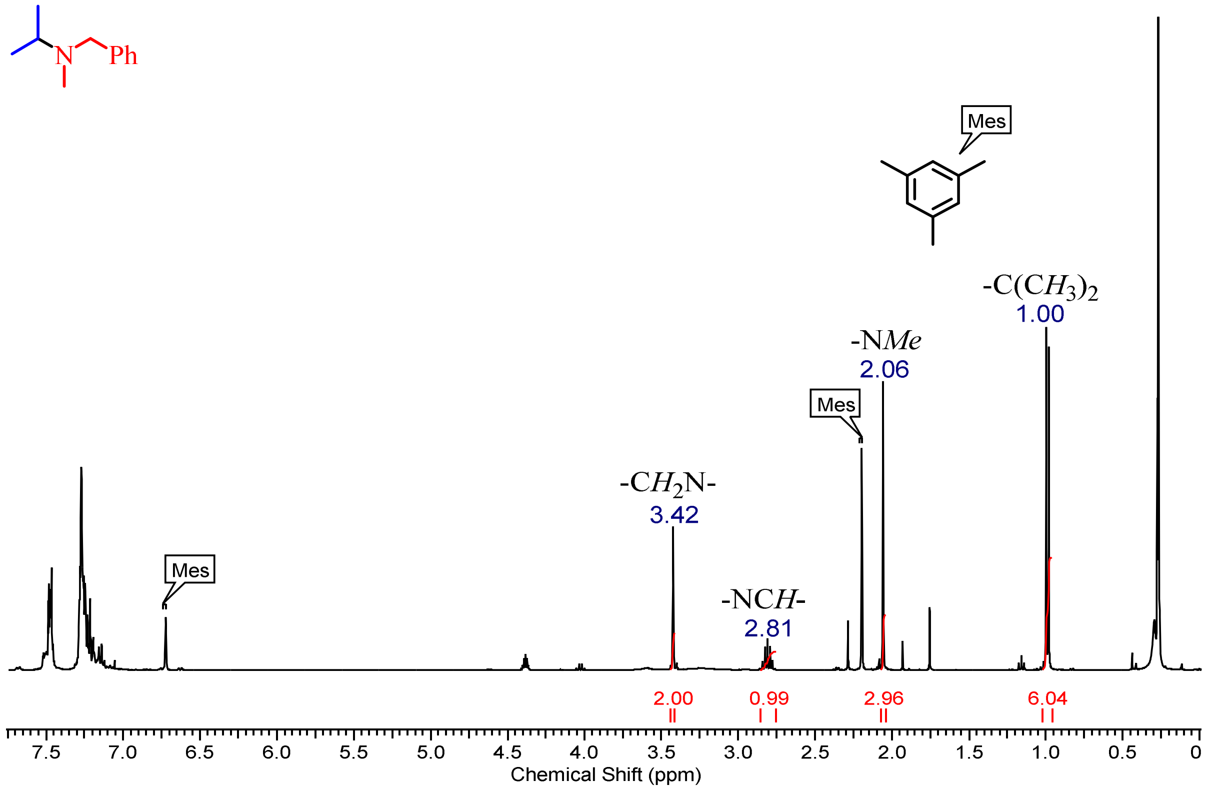

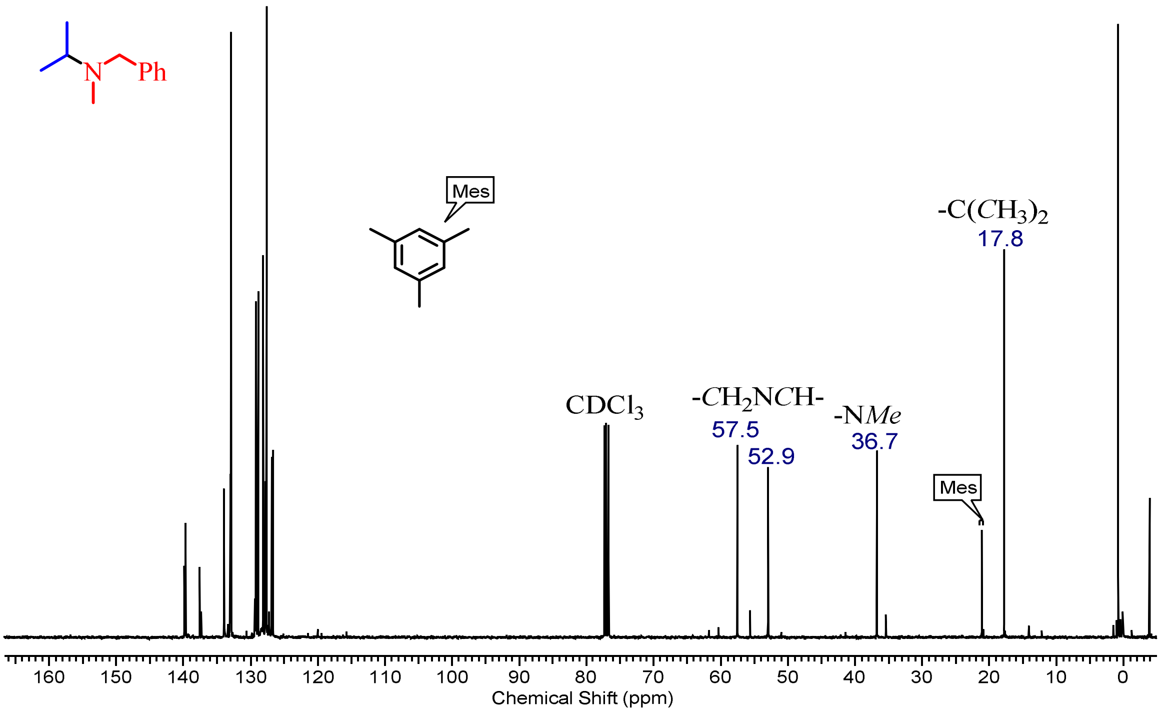
N
Ph
Mes
$CDCl_3$
$-CH_2NCH-$
57.5
52.9
-NMe
36.7
Mes
$-C(CH_3)_2$
17.8
160
150
140
130
120
110
100
90
80
70
60
50
40
30
20
10
0
Chemical Shift (ppm)

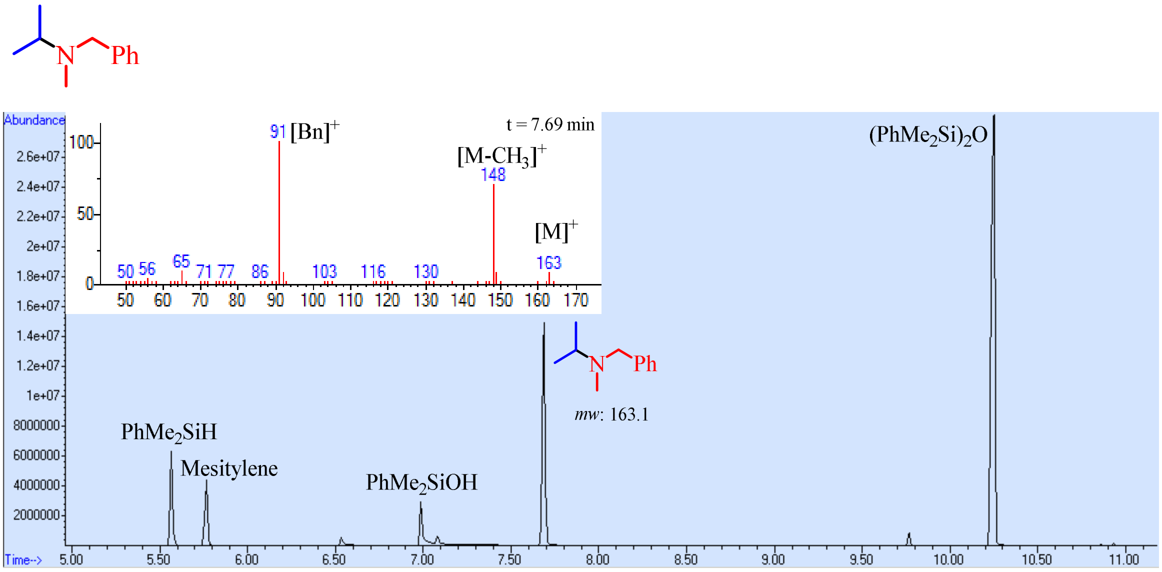
N
Ph
Abundance
t = 7.69 min
91 $[Bn]^+$
$[M-CH_3]^+$
148
$[M]^+$
163
50 56 65 71 77 86 103 116 130
$(PhMe_2Si)_2O$
$PhMe_2SiH$
Mesitylene
$PhMe_2SiOH$
mw: 163.1
Time-->

N-benzyl-1-adamantylamine

In situ – ^{1}H-NMR spectra (small scale)

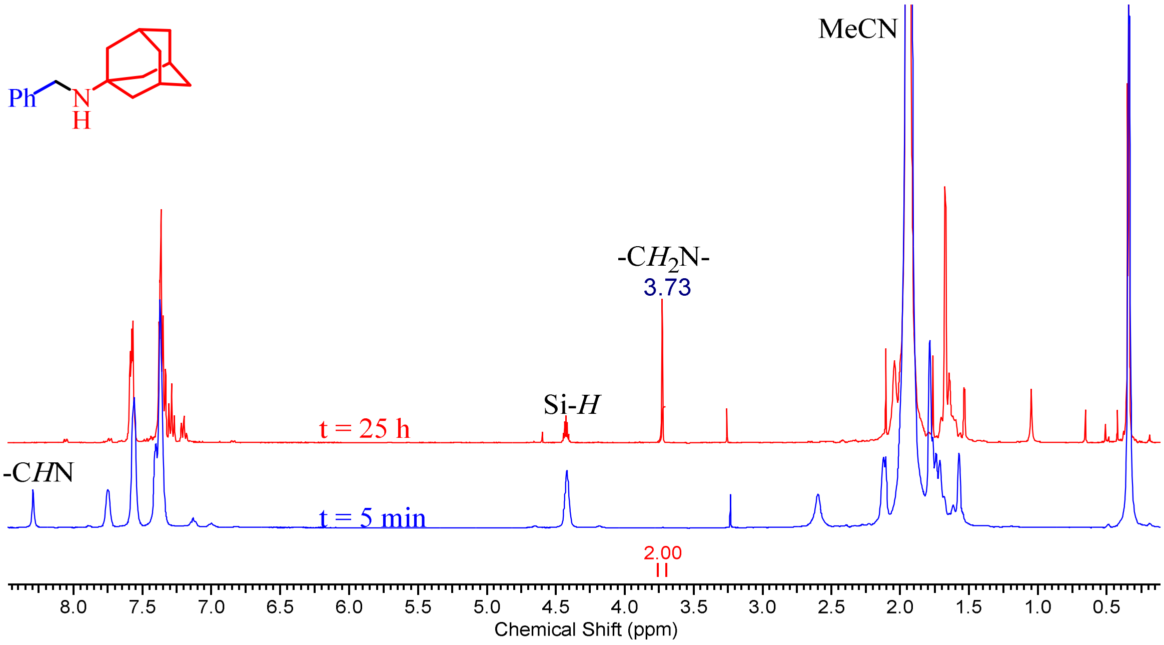

GC-MS analysis (small scale)

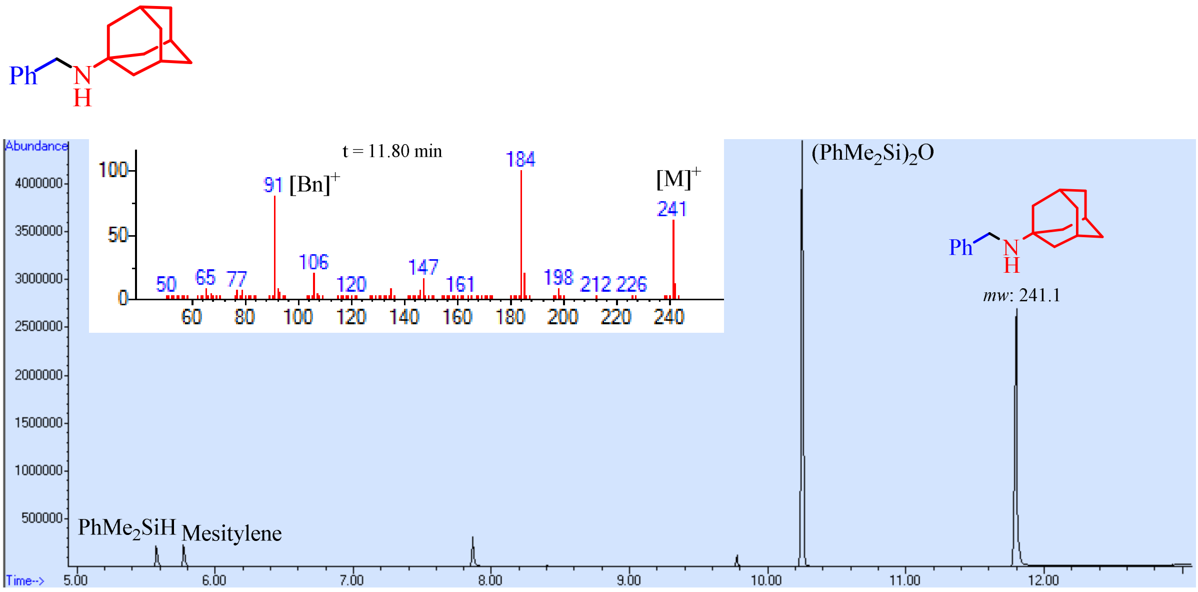

^{1}H and ^{13}C{^{1}H}-NMR spectra of isolated product (gram-scale)

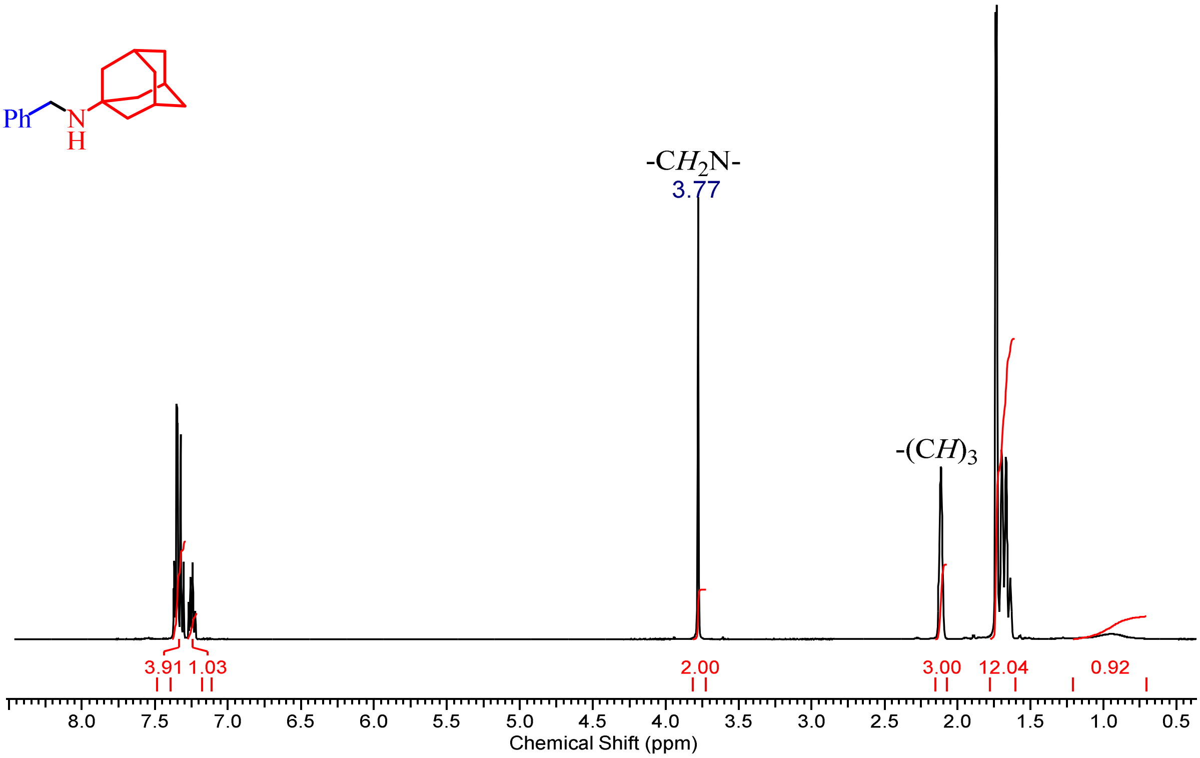

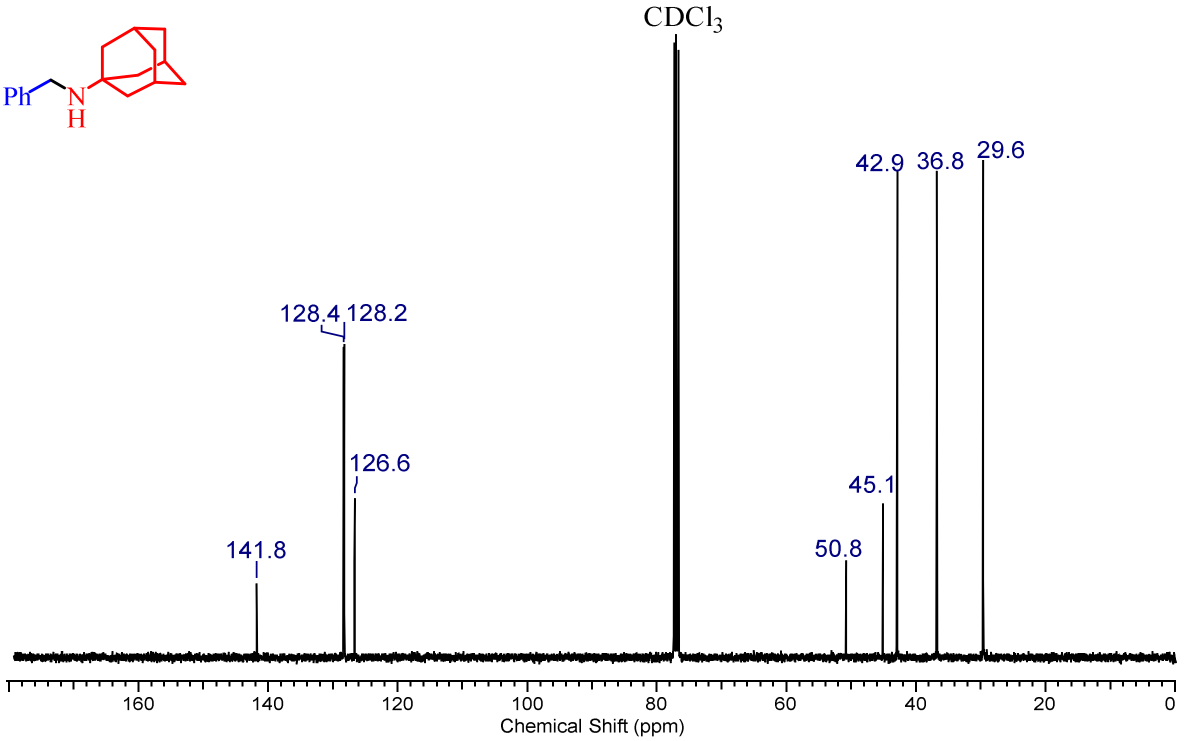

Piribedil

^{1}H and ^{13}C{^{1}H}-NMR spectra of isolated product

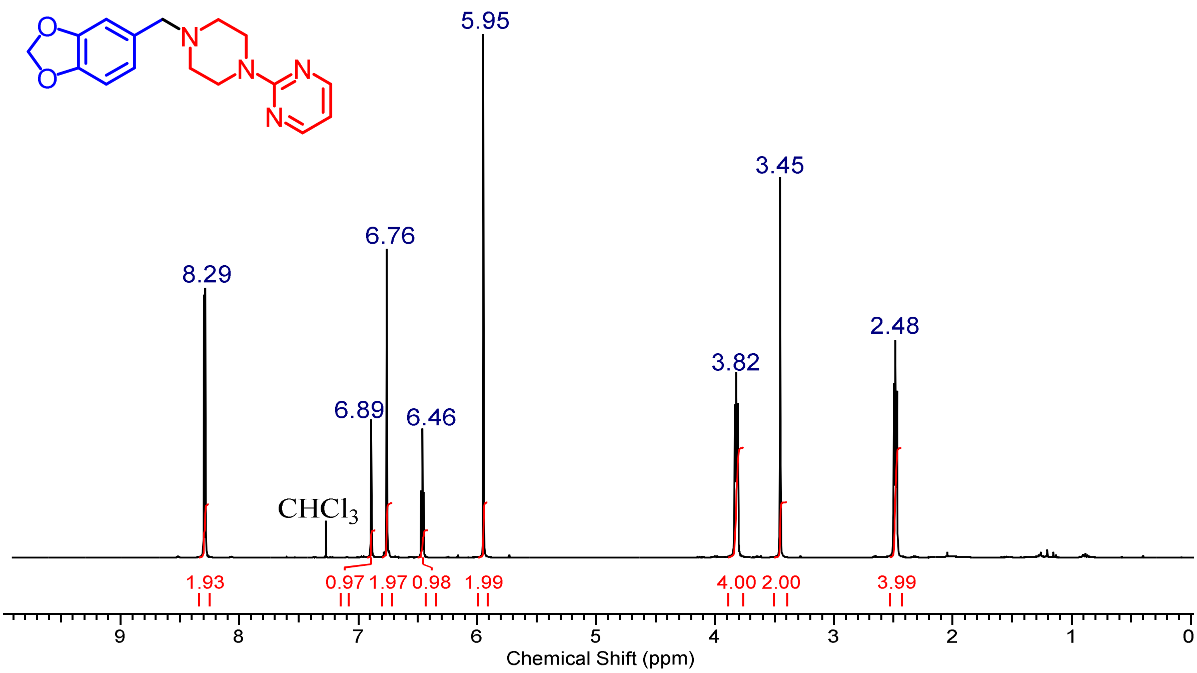

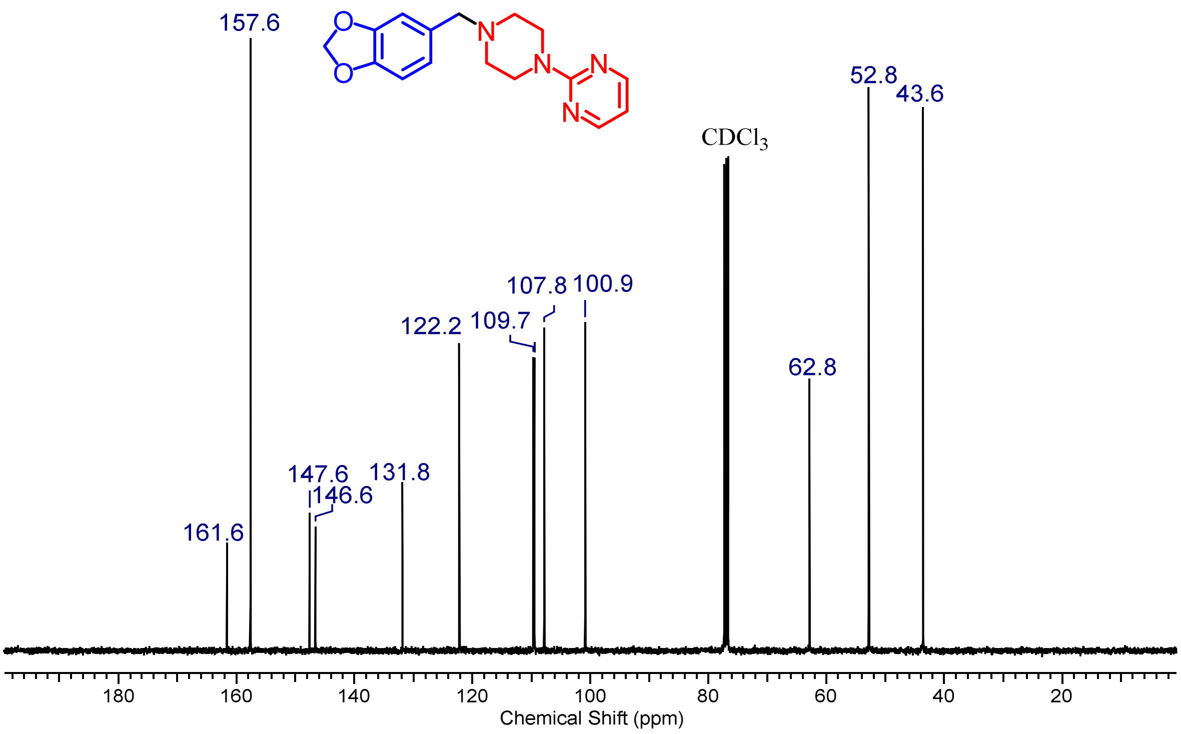

GC-MS analysis

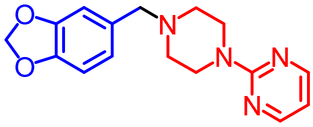

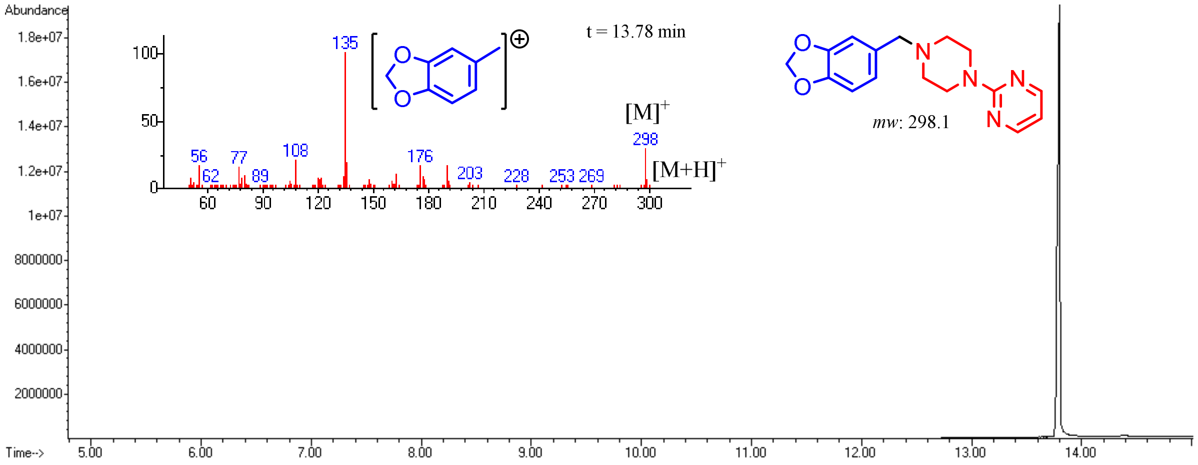

Supplement: Supplementary file 1 — Supplementary [file CHEM-23-2217-s001.pdf]
